# Supplementary material for: Discovery and chemical optimisation of a potent, Bi-cyclic antimicrobial inhibitor of Escherichia coli PBP3
Source: Commun Biol. 2025 May 28;8:819. doi: 10.1038/s42003-025-08246-x (PMC12120022; doi:10.1038/s42003-025-08246-x)

Supplementary Information for: Discovery and chemical optimisation of a Potent, Bi-cyclic Antimicrobial Inhibitor of Escherichia coli PBP3

Table S-1: Characterization of hits from phage display selections against *Ec*PBP3………………………………………………………………………………… S2

Figure S-1: Bocillin FL- and coomassie-stained gel of peptides binding to PBPs extracted from the bacterial inner membrane………………………………………… S3
Table S-2: Binding affinity to *Ec*PBP3 for peptide 2 when fluorescently labelled at the C (Tracer 1) or N (Tracer 2) terminus……………………………………………….. S3

Figure S-2: Schematic of fluorescently-labelled peptides………………………….. S4

Table S-3 Statistics of crystalised complex of *Ec*PBP3 and peptide 2………………… S5

Figure S-3: Comparison of *Ec*PBP3 in complex with peptide 2 as compared to various previously reported apo structures of *Ec*PBP3………………………………………. S6

Figure S-4. Demonstration of the tight-fitting nature of the interaction of the Bicycle molecule and protein…..……………………………………………………………… S7

Figure S-5: Two stereo views of the electron density on the Bicycle molecule……………………………………………………………………………………… S8

Figure S-6 View of the active site of *Ec*PBP3 complexed with peptide 2………….. S9

Figure S-7 Two views comparing the hydrogen bonds formed in the complex of reacted piperacillin and PBP3 and those formed in the complex of peptide 2 with *Ec*PBP3……………………………………………………………………………….. S10

Table S-4: MIC and binding evaluation of ala scan peptides…………………… S11

Table S-5: Binding affinity for EcPBP3 and activity against hyperporinated *E. coli* for peptides in the peptide 2 series……………………………………………….. S12
Figure S-8: Tolerance for amino acid substitution in Peptide 2 as characterized by binding of EcPBP3………………………………………………………………………… S14
Figure S-9: Non-natural substitutions screened in natural peptide 2…………………. S15

Table S-6: MIC evaluation of peptides and conjugates against *E. coli*………… S15

Figure S-10: Cytotoxicity of peptide 2 and conjugate 1 against human cells…………………………………………………………………………………………… S16

Table S-7: Plasma stability (mouse) of conjugates with DRAMP18563 variants……………………………………………………………………………………. S17
Table S-8: Blood stability (mouse) for SAR peptides……………………………………. S17
Table S-9: Blood stability (mouse) for SAR conjugates………………………………… S17

Table S-10: EcPBP3-targeted bicyclic peptide-vector conjugates had promising spectrum of activity against related strains of the Enterobacterales…………………… S18
Table S-11: Sequence homology (%) of the gene product of *ftsI* from relevant organisms of the Enterobacterales, plus *A. baumannii* and *P. aeruginosa*………………………………………………………………………………… S29
Peptide sequences…………………………………………………………………….. S20
Peptide QC data……………………………………………………………………..... S28

Table S-1: Characterization of hits from phage display selections against EcPBP3. Sequence motifs are highlighted in bold. NB, non-binder (compounds tested at 100 μM top concentration); NT, not tested; N/A, not applicable.

Peptide	Sequence	Copies in sequencing output	IC50 (nM) by fluorescence polarisation with Bocillin FL as tracer, Geometric mean (Lower 95% CI of geo. Mean; Upper 95% CI of geo. Mean, n=number of values)	*Ki* (nM) by fluorescence polarisation with tracer **1**, Geometric mean (Lower 95% CI of geo. Mean; Upper 95% CI of geo. Mean, n=number of values)	K_d (nM) by SPR	MIC (μg mL^{-1}) *E. coli* GKCW102 (EcPore)	MIC (μg mL^{-1}) *E. coli* GKCW102 (EcPore)
1	ACAADRLWCLAKNDWCA	43	NB (n=2)	NT	NT		
2	ACSFPKCPWVEGCA	7	602 (587;618, n=2)	<9 (n=46)	5.23	>64, >64, >64	0.5, 1, 2
3	ACAKTPEWLCGFNAYCA	6	NB (n=2)	NT	NT		
4	ACRGESSFCLMFPELCA	5	NB (n=2)	NT	NT		
5	ACRTFGCWWEGCA	2	1550 (469;2630, n=2)	NT	369	>64, >64, >64	32, 32, 64
6	ACADTIYSLTCVPYPCA	2	NB (n=2)	NT	NT		
7	ACATEFL**YPL**CW**W**DNCA	6	NB (n=2)	NT	NT		
8	ACEVV**YPL**CY**W**SDCA	1	NB (n=2)	NT	NT		
9	ACYK**YPGL**CLEFTNMCA	1	NB (n=2)	NT	NT		
10	AC**YPGL**PPELCSPSFCA	1	NB (n=2)	NT	NT		
11	ACPYA**YPGL**CLEFKSCA	1	NB (n=2)	NT	NT		
12	ACPERLCA**LGLP**T**L**RCA	1	NB (n=2)	NT	NT		
13	ACLENCYYVPYYGYACA	1	3800 (3000;4600, n=2)	NT	NT		
14	ACIRERCWDLKENDWCA	1	NB (n=2)	NT	NT		
15	ACARPVVLCYWPEDCA	1	NB (n=2)	NT	NT		
	Carbenicillin		NT	NT	NT	4, 8, 8	0.5, 1, 1

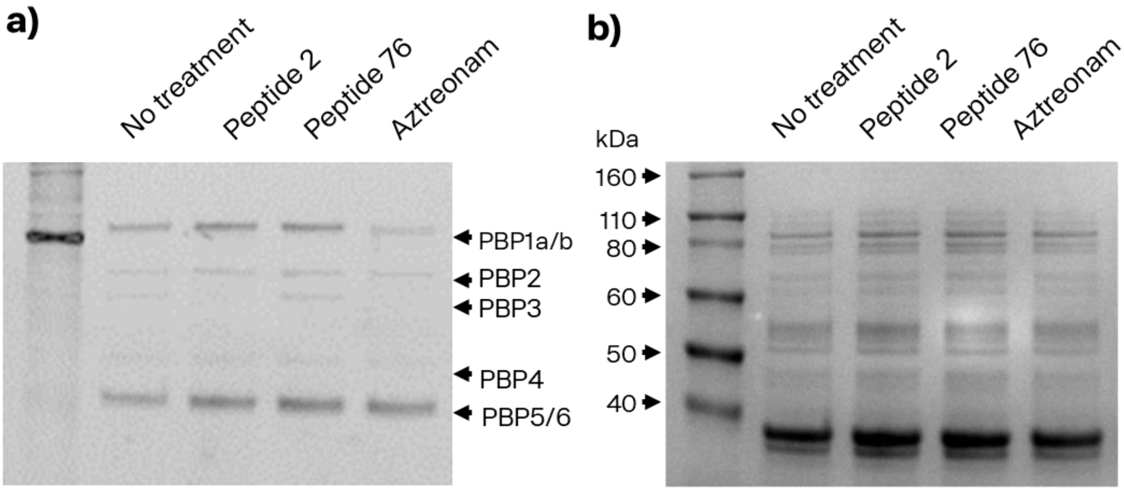

Figure S-1: Bocillin FL- and Coomassie-stained gel of peptides binding to PBPs extracted from the bacterial inner membrane. PBPs were extracted from the bacterial inner membrane, incubated with the indicated compounds (Peptides at 3 µM, aztreonam at 10 µM) for 15 minutes then stained with Bocillin FL (10 µg mL^{-1}) for 10 minutes before being washed and imaged (a) for fluorescence when excited at 455-485 nM and (b) then stained with Coomassie and imaged in brightfield. Aztreonam was used as a positive control. Ladder is Novex Sharp Pre-Stained Protein Standard (Invitrogen), the 80 kDa band of which exhibits fluorescence when excited at 455-485 nM.

Table S-2: Binding affinity to EcPBP3 for peptide 2 when fluorescently labelled at the C (Tracer 1) or N (Tracer 2) terminus.

Compound	N-terminus	C-terminus	K_d by fluorescence direct binding, Geomean (Lower 95% CI of geo. Mean; Upper 95% CI of geo. Mean, n=number of values) (nM)	K_d by SPR (nM)
Tracer 1	H	Sar_6K[Fl]	2110 (1830;2380, n=2)	NT
Tracer 2	[Fl]$GSar_5$	NH_2	6.86 (5.12;8.60, n=47)	28.1

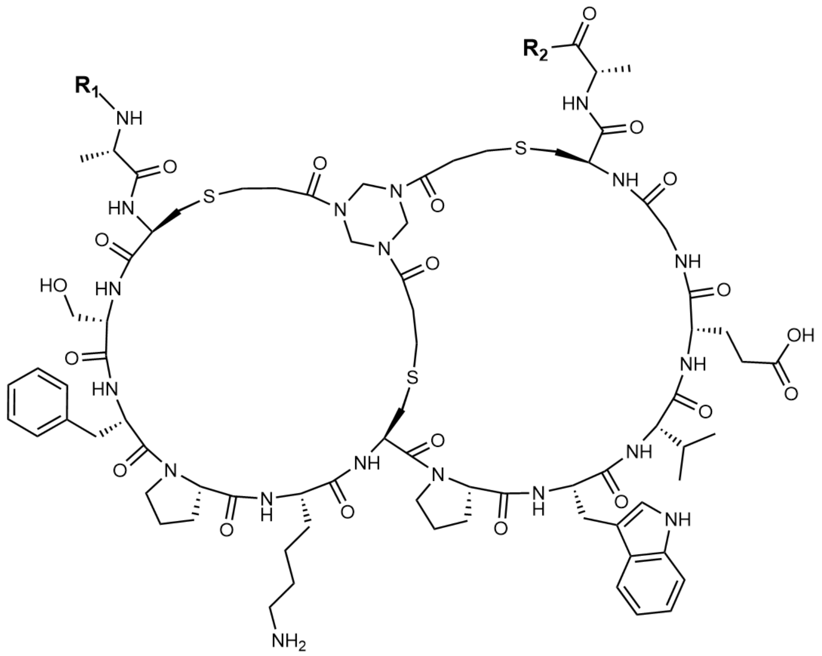

	R_1	R_2
Tracer 1	H	Sar_6K[Fl]
Tracer 2	[Fl]GSar_5	NH_2

Figure S-2: Schematic of fluorescently-labelled peptides. Labelled peptides were used as tracers in fluorescence polarisation assays. Sar, sarcosine; Fl, fluorescein.

Table S-3 Statistics of crystalised complex of EcPBP3 and peptide 2.

Data collection and processing statistics	
Synchrotron, Beam line	SLS, PXIII
Date of data collection	23rd Apr 2020
Wavelength (Å)	1.000
Detector type	Pilatus 2M
Transmission (%)	100
Temperature (K)	100
Exposure time (s)	0.1
Oscillation range per frame (˚)	0.2
Overall rotation (˚)	360
Resolution range (Å)	45.04 – 1.52
Number of observed reflections	618393
Number of unique reflections	50525
Multiplicity (overall and last shell)	12.2 (7.1)
Completeness (%) (overall and last shell)	99.6 (93.0)
R_{pim} (%) (overall and last shell)	0.6 (42.5)
Mean I/sigma (overall and last shell)	23.8 (1.7)
Space group	$C\ 222_1$
Unit cell parameters (Å); (˚)	97.89, 152.18, 43.67; 90, 90, 90
Refinement statistics	
Refinement program	Refmac5
Resolution range (Å)	45.08 – 1.52
Number of reflections (working; test)	50510; 2525
R_{factor} (%)	18.2
R_{free} (%)	21.6
Number of protein atoms modeled	2838
Number of water atoms modeled	261
Number of ligand atoms modeled	24
RMSD Bond lengths (Å)	0.015
RMSD Bond angles (˚)	1.88
Mean overall protein B value (Å^2)	26.38
Mean water B value (Å^2)	35.91
Mean ligand B value (Å^2)	30.40
Ramachandran plot favored (%)	98.49
Ramachandran plot allowed (%)	1.21
Ramachandran plot outlier region (%)	0.3

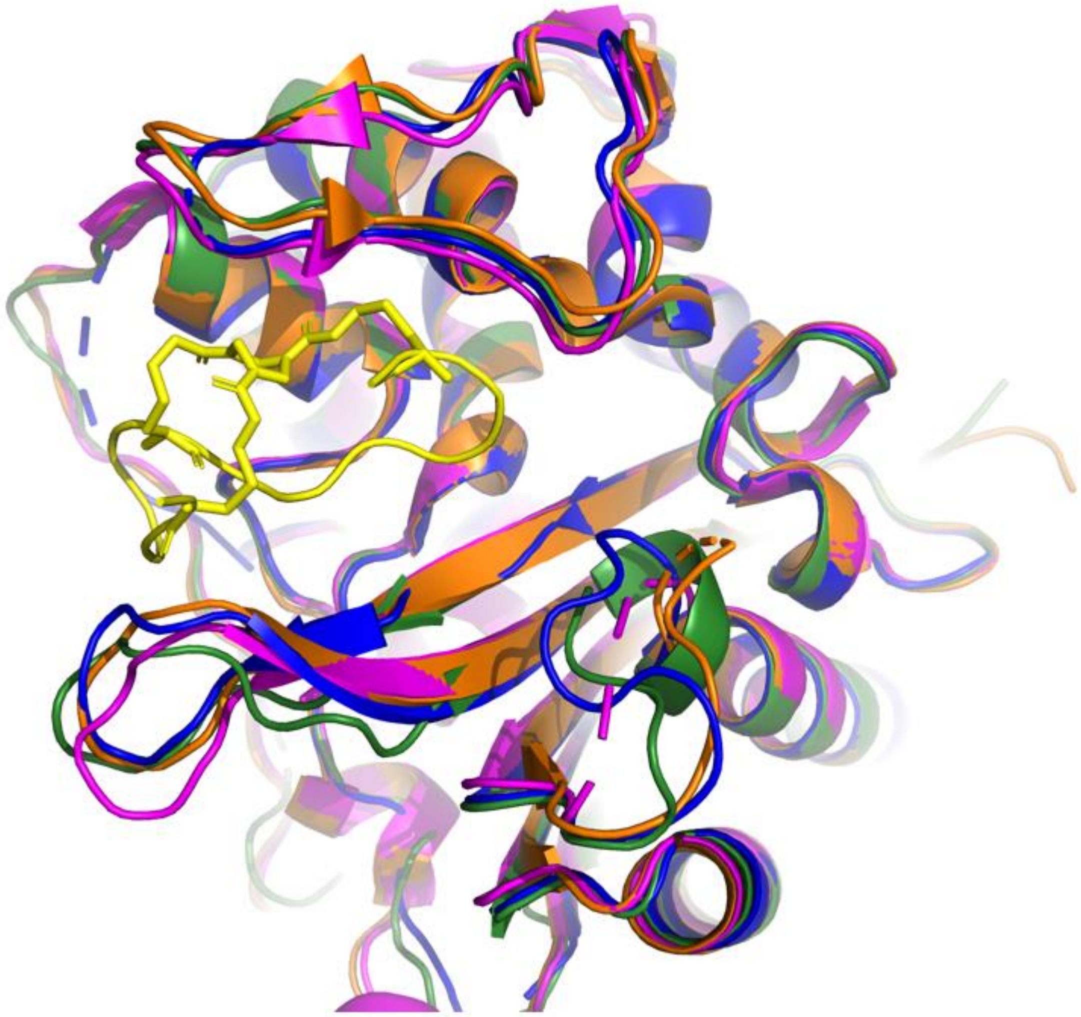

Figure S-3: Comparison of EcPBP3 in complex with peptide 2 (PDB code: 8RTZ) as compared to various previously reported apo structures of EcPBP3. PDB codes: 4BJP (Pink), 7ONO (Orange), 6HZQ (Blue).[1–3] Despite the presence of the large ligand (yellow), few changes in the backbone structure of the protein were observed, with only flexible loop regions such as the β3-β4 loop and the β5-α11 varying significantly between the structures.[3] Root mean squared deviation (RMSD) of 0.357 Å (calculated by Pymol, aligning *apo* EcPBP3 (PDB code: 6HZQ) and the EcPBP3:peptide complex).

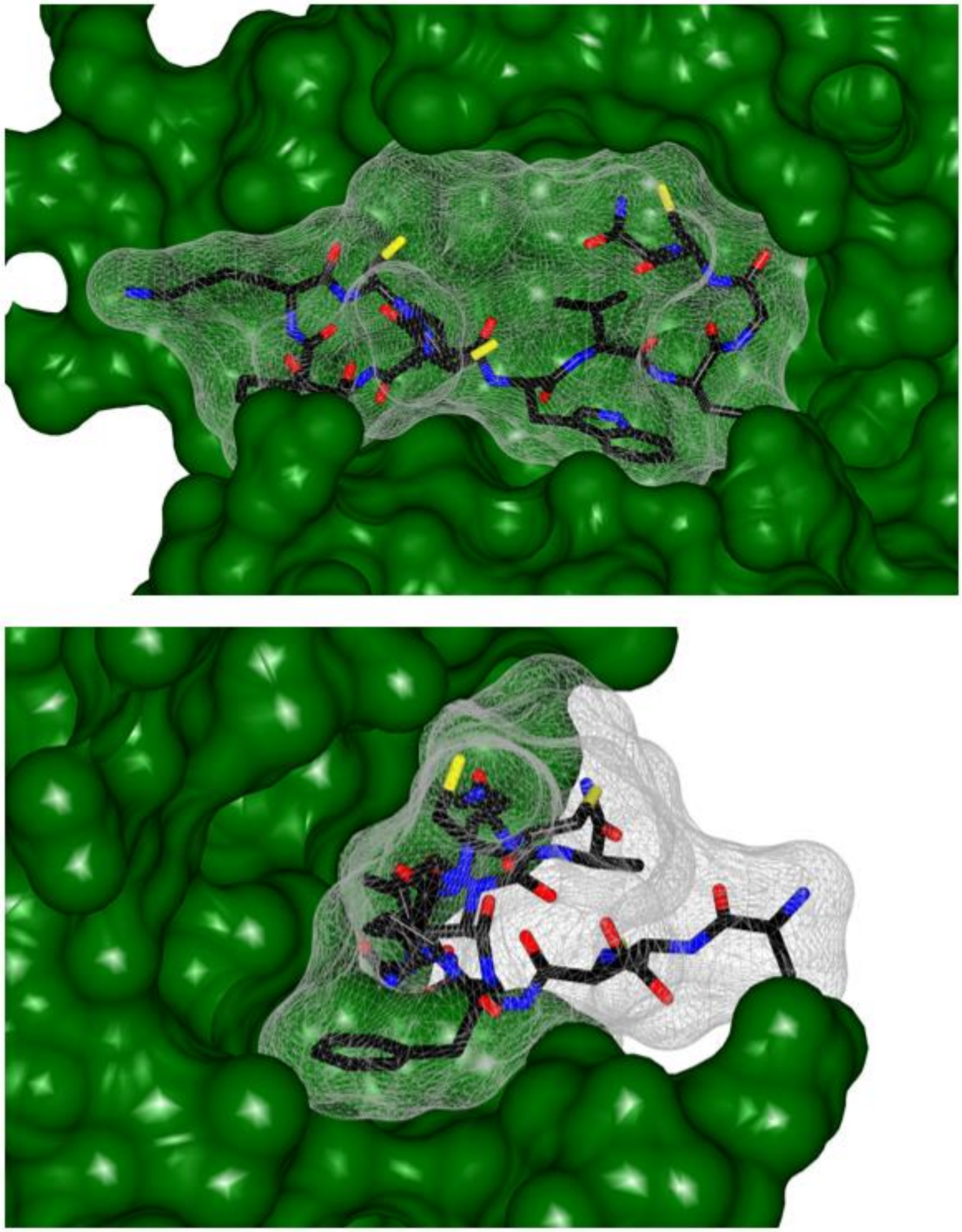

Figure S-4. Demonstration of the tight-fitting nature of the interaction of the Bicycle molecule and protein. Two views of the surface of the peptide:protein interaction. The peptide (peptide **2**) is shown in black and its space filling representation shown in grey mesh. The protein is represented in green surface. Figure generated in CCP4mg (v2.10.11).

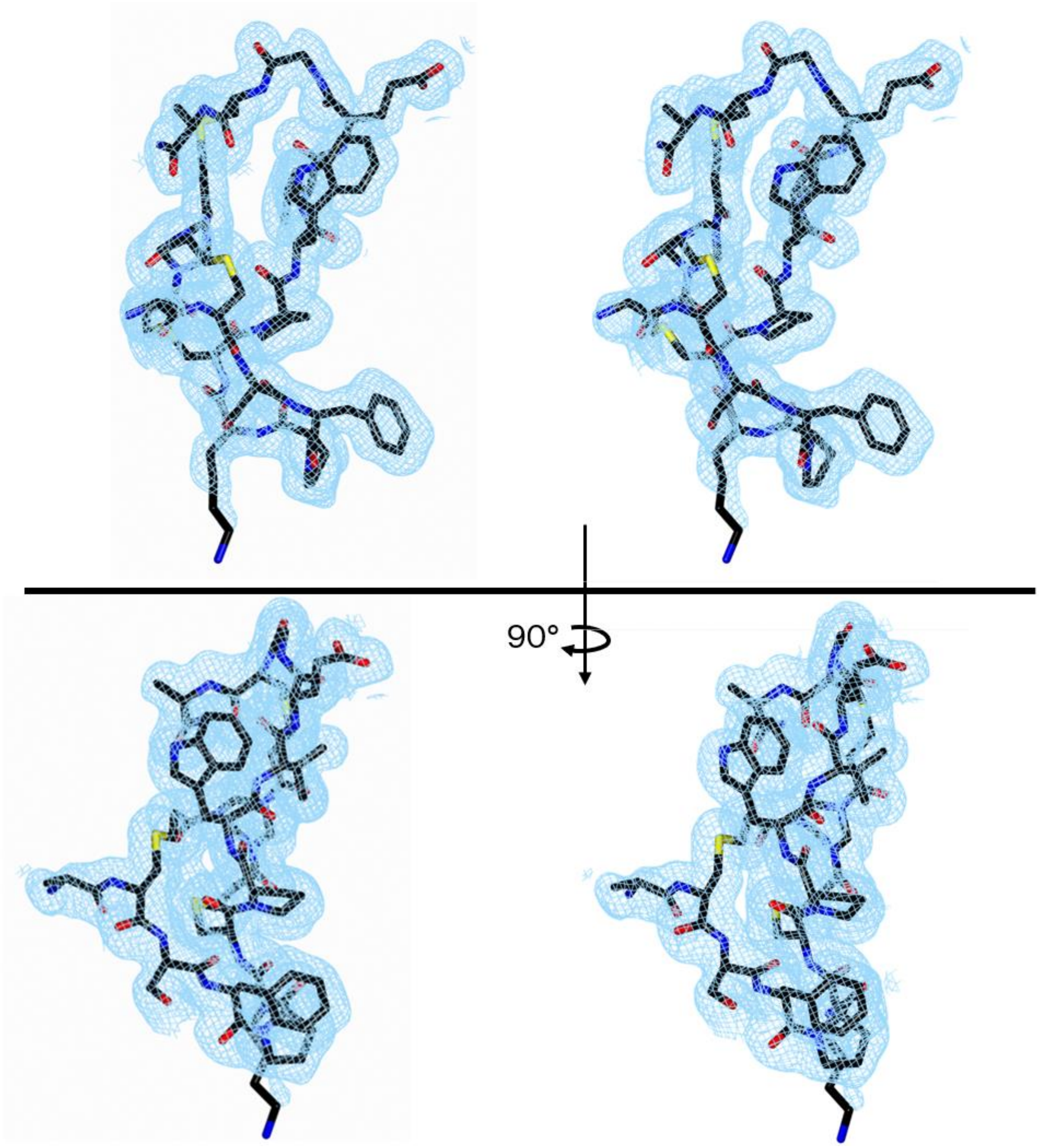

Figure S-5: Two stereo views of the electron density on the Bicycle molecule. Electron density is contoured at 1 σ. Composite Omit maps generated with PHENIX. [4] Figure generated in CCP4mg (v2.10.11).

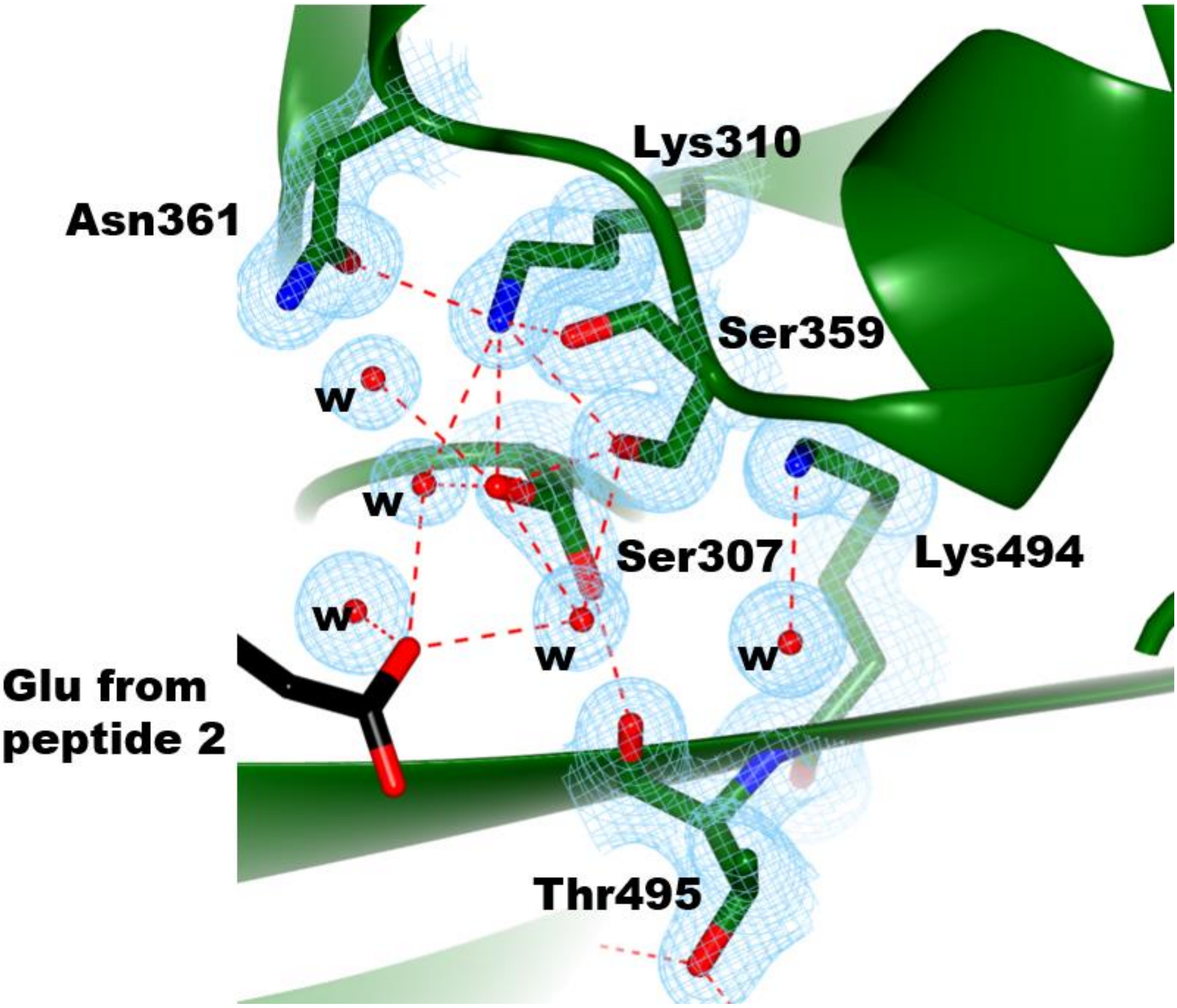

Figure S-6 View of the active site of EcPBP3 complexed with peptide 2. Side chains and electron densities are shown for conserved residues of the active site (green): Ser307 and Lys 310 (from the **S**xx**K** motif); Ser 359 and Asn361 (from the SxN motif); Lys494 and Thr495 from the **K**(S/**T**)G motif as well as several waters in the active site (indicated with a **w**). The glutamate residue (Glu8) from peptide 2 (black) is found adjacent (5.3 Å from the Glutamate Oε1 atom to Ser307 Cα atom) to the active site, but only interacts with active site residues via bridging waters. Density (light blue mesh, Composite Omit maps generated with PHENIX) for all active site residues is well defined. The catalytic serine (Ser307) exists in two alternate conformations (each with refined with 50% occupancy). Hydrogen bonds (red dashed lines) are shown between residues and key water molecules in the active site. Figure generated in CCP4mg (v2.10.11).

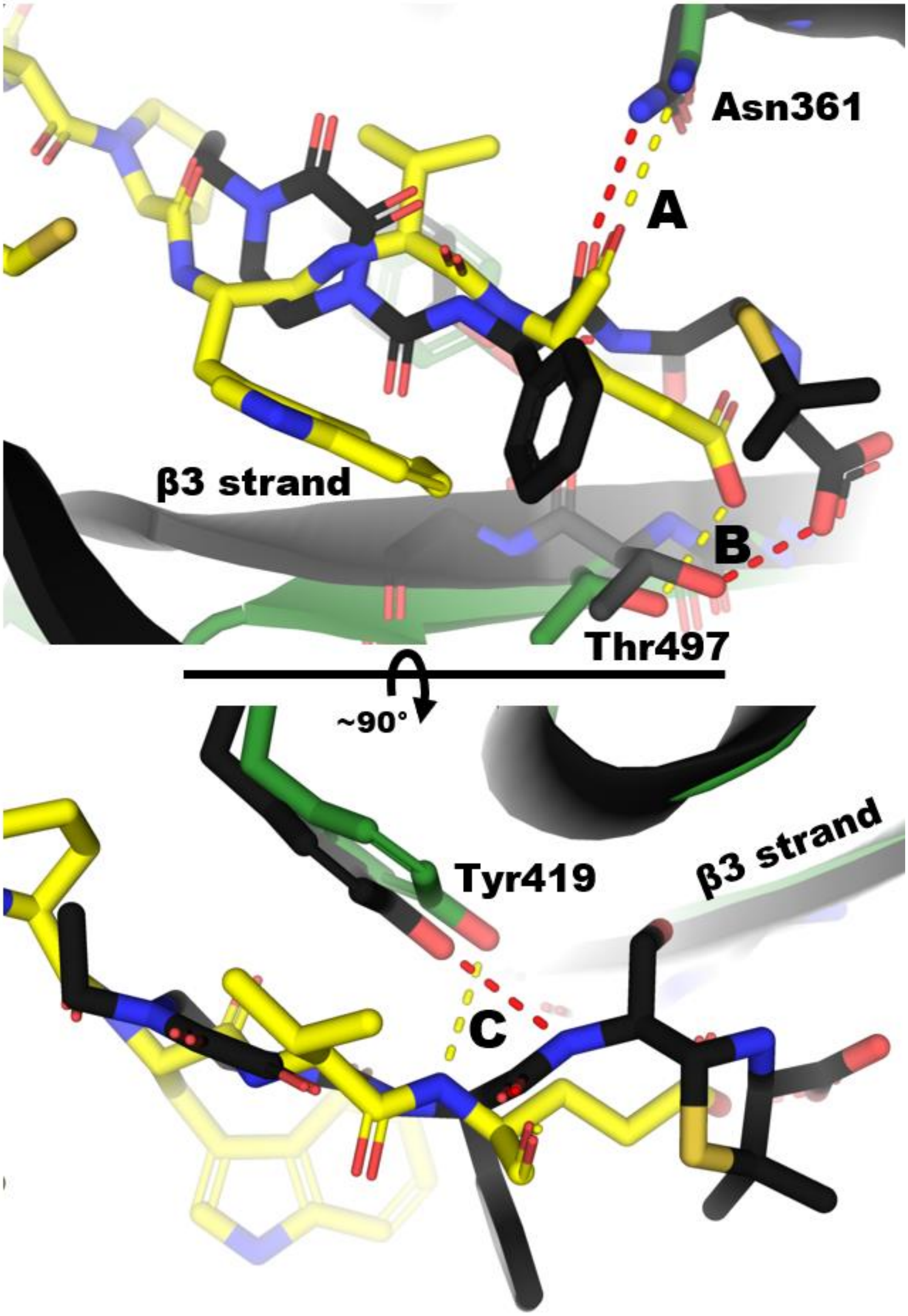

Figure S-7 Two views comparing the hydrogen bonds formed in the complex of reacted piperacillin and PBP3 (red dashes, from PDB code: 6I1I) and those formed in the complex of peptide 2 with EcPBP3 (yellow dashes). Three hydrogen bonds are conserved in the two binding interactions, labelled '**A**', '**B**' and '**C**'. Hydrogen bond '**A**' is formed between Asn361 and a peptide carboxyl in both molecules. Asn361 is in one of the conserved active site motifs of the PBPs (the Sx**N** motif) and this bond is seen in many other β-lactam:PBP structures. [3] Hydrogen bond '**B**' is formed between acidic groups on both molecules and Thr497. In β-lactams, this acidic group is highly conserved. Hydrogen bond '**C**' is formed between Tyr419's OH and a backbone amide nitrogen. The conservation of these hydrogen bonds in the two ligands is evidence of a shared binding mode.

Table S-4: MIC and binding evaluation of ala scan peptides. MICs were generated in caMHB medium. MIC data are given for n-3 biological replicates - values for peptide 2 are representative of range across >8 replicates in separate assays. Alanine substitutions are marked in bold.

Peptide	Sequence	*Ki* (nM) by fluorescence polarisation with BCY12820 as tracer, Geometric mean (Lower 95% CI of geo. Mean; Upper 95% CI of geo. Mean, n=number of values)	K_d (nM) by SPR	MIC (µg mL^{-1}) *E. coli* GKCW102 (EcPore)
2	ACSFPKCPWVEGCA	<9 (n=46)	5.23	0.5, 1, 2
16	AC**A**FPKCPWVEGCA	<9 (n=3)	6.44	0.5, 2, 2
17	ACS**A**PKCPWVEGCA	331, >5000 (n=2)	NB[1]	>64, >64, 256
18	ACSF**A**KCPWVEGCA	76.9 (68.9;85.0, n=3)	178	4, 8, 8
19	ACSFP**A**CPWVEGCA	<9 (n=2)	12.4	0.5, 0.5, 1
20	ACSFPKC**A**WVEGCA	294, >5000 (n=2)	1380	64, 64, 128
21	ACSFPKCP**A**VEGCA	>5000 (n=2)	NB[1]	>64, >64, >256
22	ACSFPKCPW**A**EGCA	>5000 (n=2)	669	32, 32, 32
23	ACSFPKCPWV**A**GCA	9.08 (4.64;13.5, n=3)	6.71	0.25, 0.5, 0.5
24	ACSFPKCPWVE**A**CA	25.5 (18.2;32.7, n=3)	24.4	0.5, 1, 2

[1] No binding observed at 2500 nM

Table S-5: Binding affinity for EcPBP3 and activity against hyperporinated *E. coli* for peptides in the peptide 2 series. Substituted amino acids are marked in bold.

Peptide no.	Sequence	*Ki* (nM) with BCY12820 as tracer, Geometric mean (Lower 95% CI of geo. Mean; Upper 95% CI of geo. Mean, n=number of values)	K_d (nM) by SPR	MIC (µg mL^{-1}) *E. coli* GKCW102 (EcPore)
25	ACSFP**[hArg]**CPWVEGCA	<9 (n=7)	2.85	0.5, 0.5, 0.5
26	AC**G**FP**[HArg]**CPWVEGCA[CONH2]	17.1 (1.44;32.7; n=2)	NT	2, 2, 4
27	AC**L**FP**[HArg]**CPWVEGCA[CONH2]	<9 (n=2)	3.36	0.25, 0.25, 0.25
28	AC**F**FP**[HArg]**CPWVEGCA[CONH2]	<9 (n=2)	1.12	0.125, 0.125, 0.25
29	AC**N**FP**[HArg]**CPWVEGCA[CONH2]	<9 (n=2)	NT	0.25, 1, 1
30	AC**Q**FP**[HArg]**CPWVEGCA[CONH2]	<9 (n=2)	NT	1, 1, 1
31	AC**E**FP**[HArg]**CPWVEGCA[CONH2]	<9 (n=2)	NT	0.25, 0.25, 0.5
32	ACS**H**P**[HArg]**CPWVEGCA[CONH2]	115 (70.1;161, n=3)	NT	32, 64, 64
33	ACSF**G[HArg]**CPWVEGCA[CONH2]	53.5 (51.1;55.8, n=2)	NT	4, 8, 8
34	ACSF**Y[HArg]**CPWVEGCA[CONH2]	27.1 (11.4;42.8, n=2)	NT	4, 4, 4
35	ACSF**S[HArg]**CPWVEGCA[CONH2]	53.4 (32.7;74.1, n=2)	NT	8, 16, 16
36	ACSF**T[HArg]**CPWVEGCA[CONH2]	75.0 (55.0;95.0, n=2)	NT	8, 8, 16
37	ACSF**H[HArg]**CPWVEGCA[CONH2]	31.5 (13.3;49.7, n=2)	NT	4, 4, 4
38	ACSF**D[HArg]**CPWVEGCA[CONH2]	167 (29.7;304, n=2)	NT	32, 64, 64
39	ACSFP**T**CPWVEGCA[CONH2]	<9, 21.6 (n=2)	NT	0.5, 0.5, 1
40	ACSFP**H**CPWVEGCA[CONH2]	<9, 19.4 (n=2)	NT	0.5, 0.5 ,0.5
41	ACSFP**[HArg]**C**G**WVEGCA[CONH2]	>5000 (n=2)	NT	NT
42	ACSFP**[HArg]**CP**Y**VEGCA[CONH2]	209 (114;305, n=4)	NT	NT
43	ACSFP**[HArg]**CP**F**VEGCA[CONH2]	252, >5000 (n=2)	NT	NT
44	ACSFP**[HArg]**CP**H**VEGCA[CONH2]	>5000 (n=2)	NT	NT
45	ACSFP**[HArg]**CPW**I**EGCA[CONH2]	<9 (n=2)	NT	0.5,0.5,2
46	ACSFP**[HArg]**CPW**T**EGCA[CONH2]	15.5 (11.5;19.5, n=2)	NT	4, 4, 4
47	ACSFP**[HArg]**CPWV**P**GCA[CONH2]	18.0 (11.7;24.3, n=2)	NT	4, 8, 16

Table S-5 continued

Peptide no.	Sequence	*Ki* (nM) with BCY12820 as tracer, Geometric mean (Lower 95% CI of geo. Mean; Upper 95% CI of geo. Mean, n=number of values)	K_d (nM) by SPR	MIC (µg mL^{-1}) *E. coli* GKCW102 (EcPore)
48	ACSFP**[HArg]**CPWV**L**GCA[CONH2]	49.7 (29.2;70.2, n=2)	NT	16, 16, 64
49	ACSFP**[HArg]**CPWV**F**GCA[CONH2]	<9, 23.4 (n=2)	NT	4, 8, 16
50	ACSFP**[HArg]**CPWV**Q**GCA[CONH2]	<9, 32.7 (n=2)	NT	2, 8, 8
51	ACSFP**[HArg]**CPWVE**Y**CA[CONH2]	<9 (n=2)	1.83	0.125, 0.25, 0.5
52	ACSFP**[HArg]**CPWVE**N**CA[CONH2]	<9 (n=2)	NT	1, 2, 4
53	ACSFP**[HArg]**CPWVE**H**CA[CONH2]	<9 (n=2)	NT	1, 1, 2
54	ACS**[5FPhe]**PKCPWVEGCA[CONH2]	NT	701	64, 64, >64
55	ACS**[3FPhe]**PKCPWVEGCA[CONH2]	NT	16.8	1, 1, 1
56	ACS**[2FPhe]**PKCPWVEGCA[CONH2]	NT	56.6	0.5, 2, 2
57	ACS**[2MePhe]**PKCPWVEGCA[CONH2]	NT	22.3	0.5, 1, 1
58	ACS**[4MePhe]**PKCPWVEGCA[CONH2]	NT	5.06	0.5, 1, 1
59	ACS**[4Pal]**PKCPWVEGCA[CONH2]	NT	121	1, 2, 4,
60	ACS**[2Pal]**PKCPWVEGCA[CONH2]	NT	122	16, 32, 64
61	ACS**[HPhe]**PKCPWVEGCA[CONH2]	NT	232	16, 16, 64
62	ACSFPKCP**[3QuinAla]**VEGCA[CONH2]	NT	27	0.5, 0.5, 2
63	ACSFPKCP**[5MeoTrp]**VEGCA[CONH2]	NT	175	4, 4, 16
64	ACSFPKCP**[4MeoTrp]**VEGCA[CONH2]	NT	NT	64, >64, >64
65	ACSFPKCP**[6ClTrp]**VEGCA[CONH2]	NT	0.269	0.0625, 0.0625, 0.125
66	ACSFPKCP**[6FTrp]**VEGCA[CONH2]	NT	0.489	0.125, 0.125, 0.25
67	ACSFPKCPW**[tBuGly]**EGCA[CONH2]	NT	574	8, 16, >16
68	ACSFPKCPW**[Cbg]**EGCA[CONH2]	NT	187	4, 8, 8
69	ACSFPKCPW**[dA]**EGCA[CONH2]	NT	NB[1]	>16, >16, >16

[1] No binding observed at 2500 nM

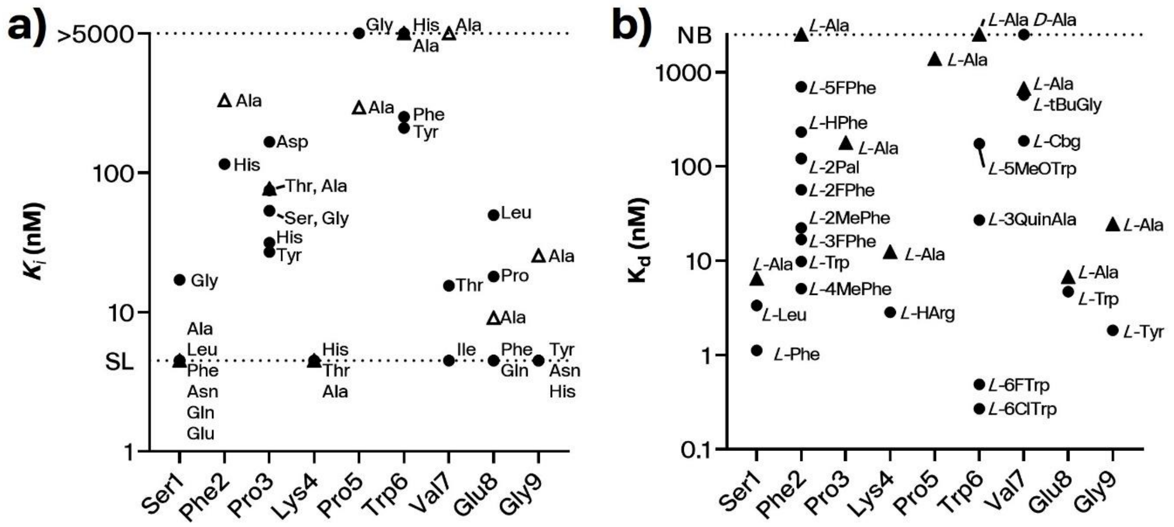

Figure S-8: Tolerance for amino acid substitution in Peptide 2 as characterized by binding of *Ec*PBP3. Single residue library selections, alanine scanning and medicinal chemistry were used to evaluate the tolerance for substitution at each position, and rationalised using structural biology. Binding of peptides to *Ec*PBP3 was evaluated in a) FP competition assays using tracer **1** and b) SPR assays. Points represent the geometric mean of at least 2 replicates. Triangles, alanine scan peptides; circles, all other substitutions. SL, sensitivity limit of assay.

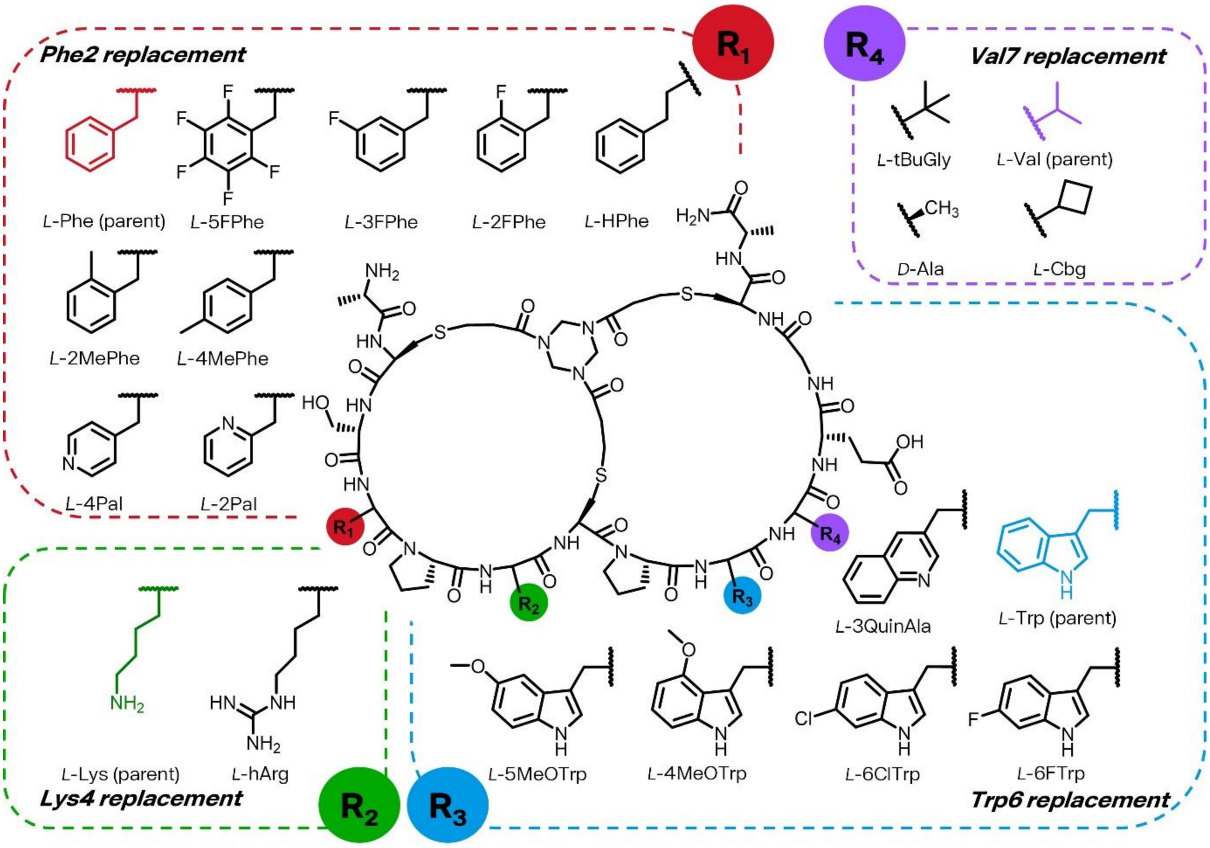

Figure S-9: Non-natural substitutions screened in natural peptide 2. 5FPhe, pentafluoro-phenylalanine; 3FPhe, 3-fluoro-phenylalanine; 2FPhe, 2-fluoro-phenylalanine; HPhe, homophenylalanine; 2MePhe, 2-methyl-phenylalanine; 4MePhe, 4-methyl-phenylalanine; 4Pal, 4-pyridylalanine; 2Pal, 2-pyridylalanine; hArg, homoarginine; 3QuinAla, 3-Quinoyl-L-alanine; 5MeOTrp, 5-methoxytryptophan; 4MeOTrp, 4-methoxytryptophan; 6ClTrp, 6-chlorotryptophan; 6FlTrp, 6-fluorotryptophan; tBuGly, tert-butyl-leucine; Cbg, cyclobutylglycine.

Table S-6: MIC evaluation of peptides and conjugates against *E. coli*. MICs were determined in caMHB for hyperporinated strains, and in MHB for *E. coli* ATCC 25922.

Compound	MIC (µg mL^{-1})			*Ki* (nM) by fluorescence polarisation with tracer 1, Geometric mean (Lower 95% CI of geo. Mean; Upper 95% CI of geo. Mean, n=number of values)
	E. coli GKCW101 (WT)	*E. coli* GKCW102 (EcPore)	*E. coli* ATCC 25922	
Peptide 2	>128, >128, >128	0.5, 1, 2	>16, >16, >16	<9 (n=46)
Conjugate 2	4, 4, 8	0.5, 1, 1	1, 2, 2	27.7nM (12.5, 42.9) n=16
Conjugate 3	>64, >64, >64	16, 16, 16	64, 64, >64	NT

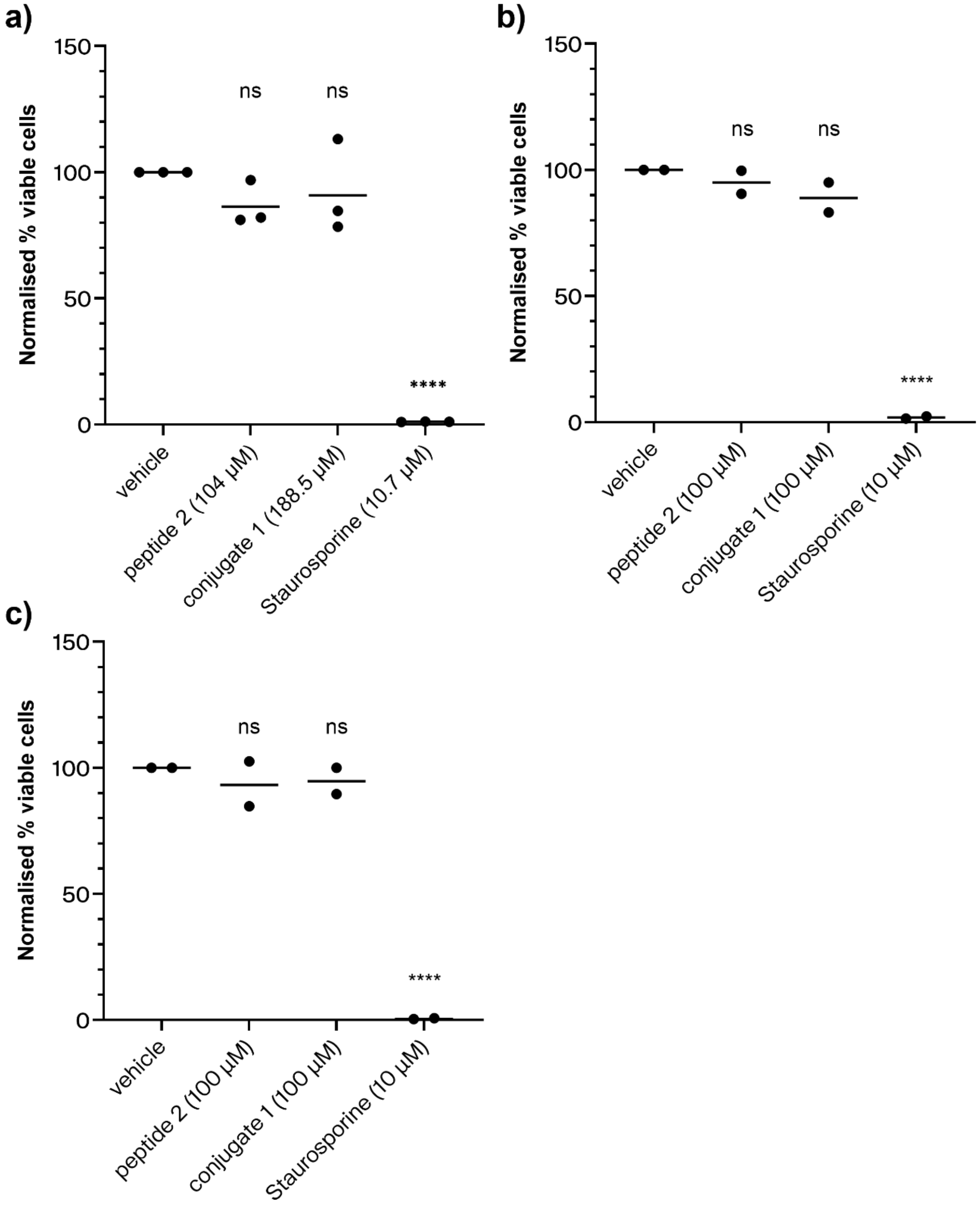

Figure S-10: Cytotoxicity of peptide 2 and conjugate 1 against human cells. Compounds were tested against a) HT1080 cells, b) A549 cells and c) HepG2 cells. Each point represents a technical replicate, derived from n≥2 biological replicates. Data were analyzed using an unpaired t-test (two-tailed) to compare compounds with the vehicle control. ns, no significant difference; ****, $P < 0.0001$.

Table S-7: Plasma stability (mouse) of conjugates with DRAMP18563 variants

Conjugate	DRAMP variant	Plasma stability ($t_{1/2}$), h
1	L-amino acids	0.3
2	Retroinverso	5.7
8	D-amino acids	5.3

Table S-8: Blood stability (mouse) for SAR peptides. Results are the average of two technical replicates. Data were generated with EDTA as the anticoagulant. MICs were generated in caMHB medium.

Peptide	Sequence	Blood stability ($t_{1/2}$), h	MIC (µg mL^{-1}) *E. coli* GKCW102
2	ACSFPKCPWVEGCA	NT	0.5, 1, 2
25	ACSFP[HArg]CPWVEGCA	0.2	0.25, 0.5, 0.5
70	ACSFP[HArg]CPWVEGC	0.3	0.125, 0.125, 0.25
71	CSFP[HArg]CPWVEGCA	5.7	0.125, 0.25, 0.5
72	CSFP[HArg]CPWVEGC	3.0	0.5, 1, 1
73	[-Ac]ACSFP[HArg]CPWVEGCA	14.1	0.5, 1, 2
74	[-Ac]CSFP[HArg]CPWVEGCA	17.7	0.25, 0.5, 1
75	[-Ac]ACSFP[HArg]CP[6ClTrp]VEGCA	11.4	0.0625, 0.125, 0.125

Table S-9: Blood stability (mouse) for SAR conjugates. Results are the average of two technical replicates. Data were generated with heparin as the anticoagulant. MICs were generated in MHB medium. * indicates the position of conjugation in each case.

Conjugate	Sequence	Blood stability ($t_{1/2}$), h	MIC (µg mL^{-1}) *E. coli* ATCC25922
2	ACSFPKCPWVEGCA*	6.9	0.5, 1, 2
4	ACSFP[HArg]CP[6ClTrp]VEGCA*	8.8	0.5, 2, 2
5	[-Ac]ACSFP[HArg]CP[6ClTrp]VEGCA*	19.9	2, 4, 4
6	[-Ac]CSFP[HArg]CP[6ClTrp]VEGCA*	4.4	0.5, 1, 2
7	[-Ac]CSFP[HArg]CPWVEGCA*	2.8	1, 1, 4

Table S-10: *Ec*PBP3-targeted bicyclic peptide-vector conjugates had promising spectrum of activity against related strains of the Enterobacterales. Antimicrobial activity of Conjugate 1 and Conjugate 2 in MIC assays against strains of the Enterobacterales order (*E. coli*, *C. freundii, K. pneumoniae, E. cloacae, P. mirabilis*) as well as two additional high-priority Gram negative pathogens, *A. baumannii* and *P. aeruginosa*. MICs were generated in caMHB medium, and number of replicates are given in brackets.

Organism	% identity of *ftsI* gene product as compared to *E. coli ftsI*	Strain	MIC (µg mL^{-1})			
			Conjugate 1	Conjugate 2	Meropenem	Levofloxacin
Escherichia coli	100	ATCC 25922	2,4 (2)	2,8 (2)	0.016,0.03 (2)	0.016,0.016, 0.03 (3)
		ATCC BAA-2469	32,32 (2)	8,8 (2)	>2,>2 (2)	>2,>2,>2 (3)
		NCTC 13441	4,4 (2)	4,8 (2)	0.016,0.03 (2)	>2,>2,>2 (3)
		UTI89	4,4 (2)	8 (1)	0.016,0.016 (2)	0.03,0.125 (2)
Citrobacter freundii	96	AR0116	8,8 (2)	8 (1)	>2,>2 (2)	>2,>2 (2)
		B121	4,4 (2)	8 (1)	0.016,0.03 (2)	0.25,0.25 (2)
Klebsiella pneumoniae	94	ATCC 43816	4,4 (2)	8,16 (2)	0.03,0.03 (2)	0.06,0.06,0.06 (3)
		ATCC 700603	4,4 (2)	16 (1)	0.03,0.03 (2)	0.5,1 (2)
		B454	4,8 (2)	16,32 (2)	0.03,0.03 (2)	>2,>2,>2 (3)
		KPNIH1	8,8 (2)	32,>64 (2)	>2,>2 (2)	>2,>2,>2 (3)
Enterobacter cloacae	94	AR0032	2,4 (2)	4 (1)	0.25,0.5 (2)	0.5,0.5 (2)
		AR0038	2,2 (2)	8 (1)	>2,>2 (2)	>2,>2 (2)
		B173	4,4 (2)	16 (1)	0.03,0.03 (2)	0.03,0.03 (2)
		KPC114	4,4 (2)	8 (1)	>2,>2 (2)	0.5,0.5 (2)
Proteus mirabilis	75	AR159	>64, >128 (2)	>64 (1)	>2,>2 (2)	2,2 (2)
		ATCC 43071	>64, >128 (2)	>64 (1)	0.06,0.125 (2)	0.06,0.06 (2)
Pseudomonas aeruginosa	45	ATCC 27853	>64, >128 (2)	>64, >64 (2)	0.5,0.5 (2)	1,1,1 (3)
		NCTC 13437	>64, >128 (2)	>64 (1)	>2,>2 (2)	2,2 (2)
Acinetobacter baumannii	40	AR0275	>64, >128 (2)	>64 (1)	>2,>2 (2)	2,2 (2)
		NCTC 12156	>64, >128 (2)	>64 (1)	1,1 (2)	0.25,0.5 (2)

Table S-11: Sequence homology (%) of the gene product of *ftsI* from relevant organisms of the Enterobacterales, plus *A. baumannii* and *P. aeruginosa*. Sequences are labelled with gene, organism and the NCBI accession number for the protein sequence.

	FTSI_ECOLI	FTSI_CFREUNDII	FTSI_KPNEU	FTSI_ECLOACAE	FTSI_PMIRABILIS	FTSI_PAERU	FTSI_ABAUM
FTSI_ECOLI _WP_000642196.1	100	96	94	94	75	45	40
FTSI_CFREUNDII _WP_088902745.1	96	100	93	95	74	46	39
FTSI_KPNEUMONIAE _WP_002888559.1	94	93	100	96	75	46	40
FTSI_ECLOACAE _CP033466.1	94	95	96	100	75	45	40
FTSI_PMIRABILIS _EEI48461.1	75	74	75	75	100	43	39
FTSI_PAERUGINOSA _UTQ31465.1	45	46	46	45	43	100	39
FTSI_ABAUMANNII _AEK25671.1	40	39	40	40	39	39	100

Peptide sequences

Tracers	
Tracer	Sequence
1	[-Fl]G[Sar5]ACSFPKCPWVEGCA[CONH2]
2	ACSFPKCPWVEGCA[Sar6][KFl][CONH2]

Conjugates		
Conjugate	Peptide sequence	Vector sequence
1	ACSFPKCPWVEGCA[K(PYA)]	KSLRRVWRSWR[CAzal]
2	ACSFPKCPWVEGCA[K(PYA)]	[CAzal][dR][dW][dS][dR][dW][dV][dR][dR][dL][dS][dK][CONH2]
3	[dA][dC][dS][dF][dP][dK][dC][dP][dW][dV][dE]G[dC][dA][K(PYA)]	[CAzal][dR][dW][dS][dR][dW][dV][dR][dR][dL][dS][dK]
4	ACSFP[HArg]CP[6ClTrp]VEGCA[K(PYA)]	[CAzal][dR][dW][dS][dR][dW][dV][dR][dR][dL][dS][dK]
5	[-Ac]ACSFP[HArg]CP[6ClTrp]VEGCA[K(PYA)]	[CAzal][dR][dW][dS][dR][dW][dV][dR][dR][dL][dS][dK]
6	[-Ac]CSFP[HArg]CP[6ClTrp]VEGCA[K(PYA)]	[CAzal][dR][dW][dS][dR][dW][dV][dR][dR][dL][dS][dK]
7	[-Ac]CSFP[HArg]CPWVEGCA[K(PYA)]	[CAzal][dR][dW][dS][dR][dW][dV][dR][dR][dL][dS][dK]
8	ACSFPKCPWVEGCA[K(PYA)]	[dK][dS][dL][dR][dR][dV][dW][dR][dS][dW][dR][CAzal]

Peptides	
Peptide	Sequence
1	ACAADRLWCLAKNDWCA[CONH2]
2	ACSFPKCPWVEGCA[CONH2]
3	ACAKTPEWLCGFNAYCA[CONH2]
4	ACRGESSFCLMFPELCA[CONH2]
5	ACRTFGCWWEGCA[CONH2]
6	ACADTIYSLTCVPYPCA[CONH2]
7	ACATEFLYPLCWWDNCA[CONH2]
8	ACEVVYPLCYWSDCA[CONH2]
9	ACYKYPGLCLEFTNMCA[CONH2]

10	ACYPGLPPELCSPSFCA[CONH2]
11	ACPYAYPGLCLEFKSCA[CONH2]
12	ACPERLCALGLPTLRCA[CONH2]
13	ACLENCYYVPYYGYACA[CONH2]
14	ACIRERCWDLKENDWCA[CONH2]
15	ACARPVVLCYWPEDCA[CONH2]
16	ACAFPKCPWVEGCA[CONH2]
17	ACSAPKCPWVEGCA[CONH2]
18	ACSFAKCPWVEGCA[CONH2]
19	ACSFPACPWVEGCA[CONH2]
20	ACSFPKCAWVEGCA[CONH2]
21	ACSFPKCPAVEGCA[CONH2]
22	ACSFPKCPWAEGCA[CONH2]
23	ACSFPKCPWVAGCA[CONH2]
24	ACSFPKCPWVEACA[CONH2]
25	ACSFP[HArg]CPWVEGCA[CONH2]
26	ACGFP[HArg]CPWVEGCA[CONH2]
27	ACLFP[HArg]CPWVEGCA[CONH2]
28	ACFFP[HArg]CPWVEGCA[CONH2]
29	ACNFP[HArg]CPWVEGCA[CONH2]
30	ACQFP[HArg]CPWVEGCA[CONH2]
31	ACEFP[HArg]CPWVEGCA[CONH2]
32	ACSHP[HArg]CPWVEGCA[CONH2]
33	ACSFG[HArg]CPWVEGCA[CONH2]
34	ACSFY[HArg]CPWVEGCA[CONH2]
35	ACSFS[HArg]CPWVEGCA[CONH2]
36	ACSFT[HArg]CPWVEGCA[CONH2]
37	ACSFH[HArg]CPWVEGCA[CONH2]
38	ACSFD[HArg]CPWVEGCA[CONH2]
39	ACSFPTCPWVEGCA[CONH2]

40	ACSFPHCPWVEGCA[CONH2]
41	ACSFP[HArg]CGWVEGCA[CONH2]
42	ACSFP[HArg]CPYVEGCA[CONH2]
43	ACSFP[HArg]CPFVEGCA[CONH2]
44	ACSFP[HArg]CPHVEGCA[CONH2]
45	ACSFP[HArg]CPWIEGCA[CONH2]
46	ACSFP[HArg]CPWTEGCA[CONH2]
47	ACSFP[HArg]CPWVPGCA[CONH2]
48	ACSFP[HArg]CPWVLGCA[CONH2]
49	ACSFP[HArg]CPWVFGCA[CONH2]
50	ACSFP[HArg]CPWVQGCA[CONH2]
51	ACSFP[HArg]CPWVEYCA[CONH2]
52	ACSFP[HArg]CPWVENCA[CONH2]
53	ACSFP[HArg]CPWVEHCA[CONH2]
54	ACS[5FPhe]PKCPWVEGCA[CONH2]
55	ACS[3FPhe]PKCPWVEGCA[CONH2]
56	ACS[2FPhe]PKCPWVEGCA[CONH2]
57	ACS[2MePhe]PKCPWVEGCA[CONH2]
58	ACS[4MePhe]PKCPWVEGCA[CONH2]
59	ACS[4Pal]PKCPWVEGCA[CONH2]
60	ACS[2Pal]PKCPWVEGCA[CONH2]
61	ACS[HPhe]PKCPWVEGCA[CONH2]
62	ACSFPKCP[3QuinAla]VEGCA[CONH2]
63	ACSFPKCP[5MeoTrp]VEGCA[CONH2]
64	ACSFPKCP[4MeoTrp]VEGCA[CONH2]
65	ACSFPKCP[6ClTrp]VEGCA[CONH2]
66	ACSFPKCP[6FTrp]VEGCA[CONH2]
67	ACSFPKCPW[tBuGly]EGCA[CONH2]
68	ACSFPKCPW[Cbg]EGCA[CONH2]
69	ACSFPKCPW[dA]EGCA[CONH2]

70	ACSFP[HArg]CPWVEGC[CONH2]
71	CSFP[HArg]CPWVEGCA[CONH2]
72	CSFP[HArg]CPWVEGC[CONH2]
73	[-Ac]ACSFP[HArg]CPWVEGCA[CONH2]
74	[-Ac]CSFP[HArg]CPWVEGCA[CONH2]
75	[-Ac]ACSFP[HArg]CP[6ClTrp]VEGCA[CONH2]
76	[dA][dC][dS][dF][dP][dK][dC][dP][dW][dV][dE]G[dC][dA][CONH2]

References

(1) Sauvage, E.; Derouaux, A.; Fraipont, C.; Joris, M.; Herman, R.; Rocaboy, M.; Schloesser, M.; Dumas, J.; Kerff, F.; Nguyen-Disteche, M. Crystal Structure of Penicillin-Binding Protein 3 (PBP3) from Escherichia Coli. *PloS one* **2014**, *9* (5), e98042.

(2) Freischem, S.; Grimm, I.; López-Pérez, A.; Willbold, D.; Klenke, B.; Vuong, C.; Dingley, A. J.; Weiergräber, O. H. Interaction Mode of the Novel Monobactam AIC499 Targeting Penicillin Binding Protein 3 of Gram-Negative Bacteria. *Biomolecules* **2021**, *11* (07), 1057.

(3) Bellini, D.; Koekemoer, L.; Newman, H.; Dowson, C. G. Novel and Improved Crystal Structures of H. Influenzae, E. Coli and P. Aeruginosa Penicillin-Binding Protein 3 (PBP3) and N. Gonorrhoeae PBP2: Toward a Better Understanding of β-Lactam Target-Mediated Resistance. *Journal of molecular biology* **2019**, *431* (18), 3501–3519.

(4) Liebschner, D.; Afonine, P. V.; Baker, M. L.; Bunkóczi, G.; Chen, V. B.; Croll, T. I.; Hintze, B.; Hung, L.-W.; Jain, S.; McCoy, A. J. Macromolecular Structure Determination Using X-Rays, Neutrons and Electrons: Recent Developments in Phenix. *Acta Crystallographica Section D: Structural Biology* **2019**, *75* (10), 861–877.

Peptide QC data

Peptide 1

Data file: D:\Chemstation\1\Data\Peptides\4800-4899_BCY00012129_01 4840S1Cy1P1F2 Z (1) 2019-04-19 15-04-59.D

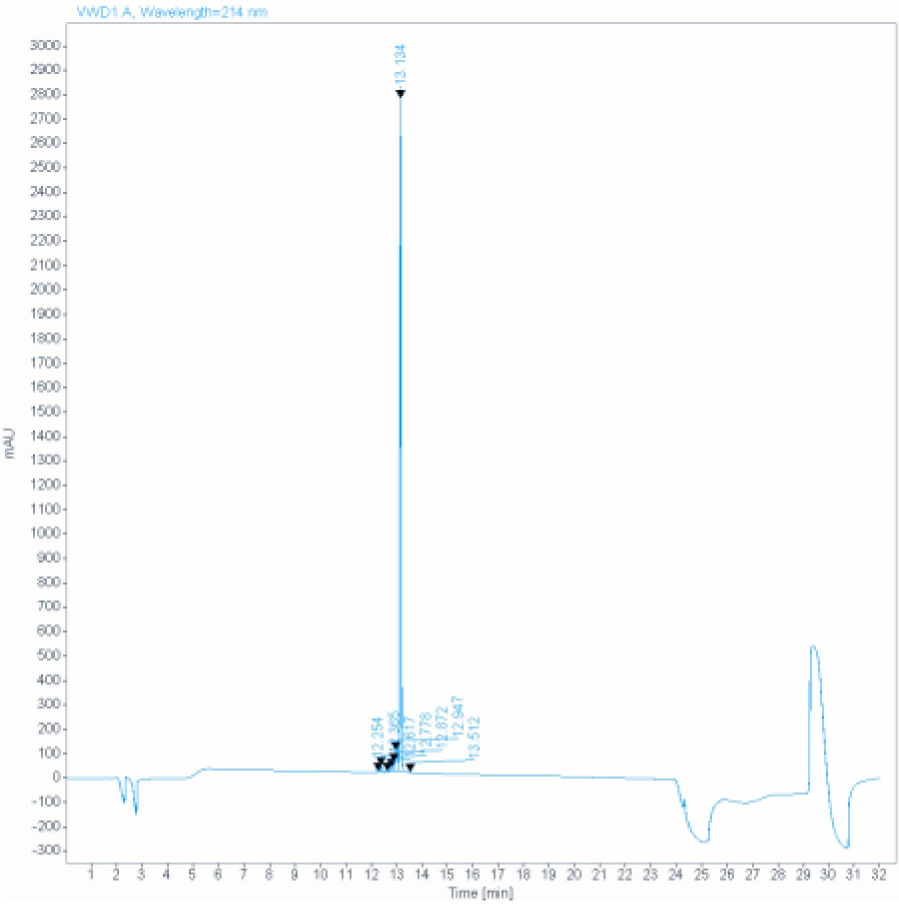

Signal: VWD1 A, Wavelength=214 nm

RT [min]	Type	Width [min]	Area	Height	Area%	Name
12.254	BV	0.0419	16.1391	5.7258	0.1683	
12.365	VB	0.0458	85.3088	28.6163	0.8894	
12.617	BB	0.0478	9.9602	2.9970	0.1038	
12.778	BV E	0.0537	66.6822	18.2018	0.6952	
12.872	VV E	0.0723	191.8802	39.4078	2.0005	
12.947	VV E	0.0816	541.4348	87.0055	5.6450	
13.134	VB R	0.0492	8669.7451	2757.6914	90.3903	
13.512	BB	0.0430	10.3085	3.8196	0.1075	
		Sum	9591.4589			

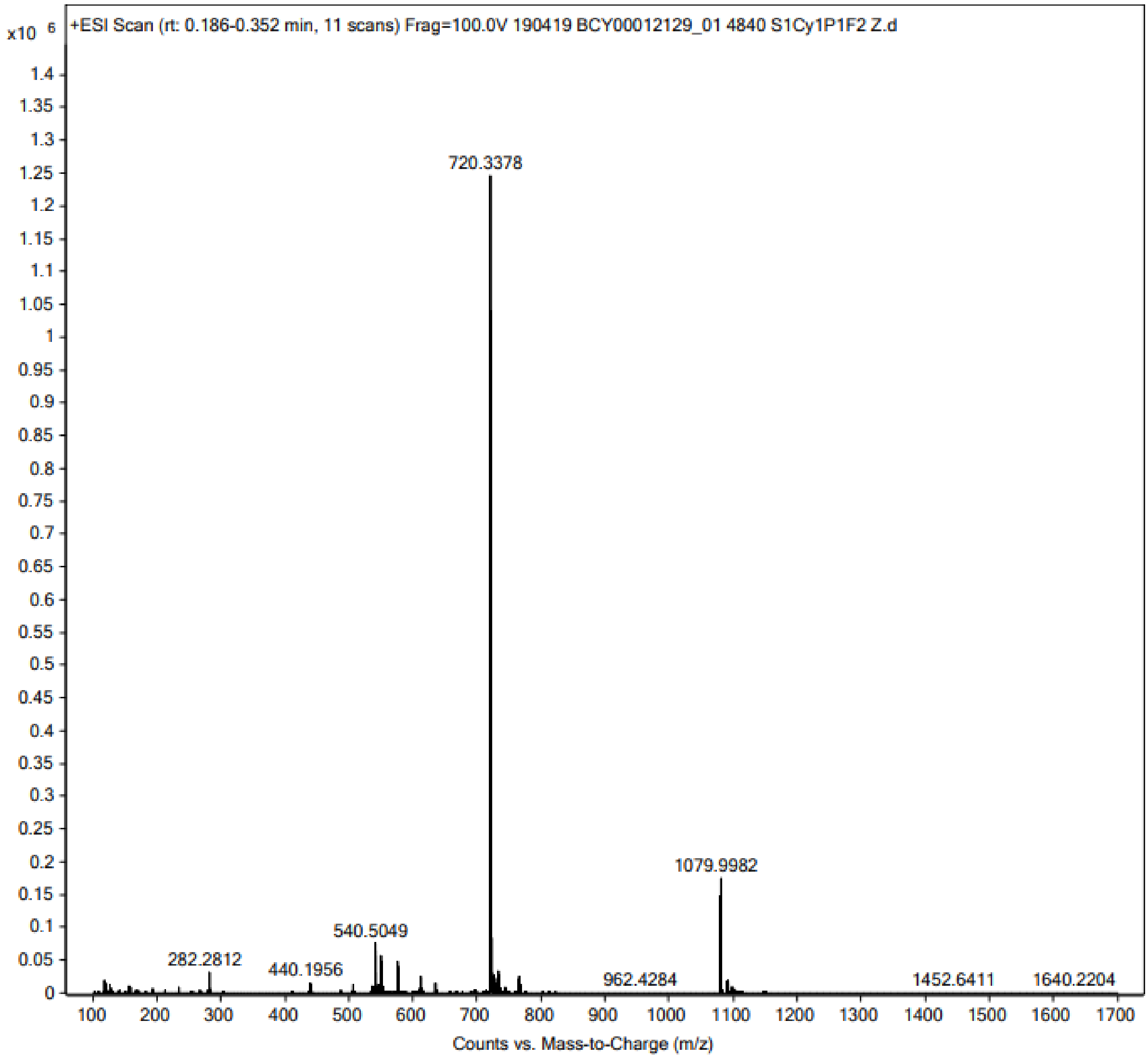

x10 6
+ESI Scan (rt: 0.186-0.352 min, 11 scans) Frag=100.0V 190419 BCY00012129_01 4840 S1Cy1P1F2 Z.d
720.3378
1079.9982
540.5049
282.2812
440.1956
962.4284
1452.6411
1640.2204
Counts vs. Mass-to-Charge (m/z)

Peptide 2

Data file: D:\Chemstation\1\Data\Peptides_facon\7000-7999_BCY00012130_05_7557_A_zHPLC 16-07-49.D

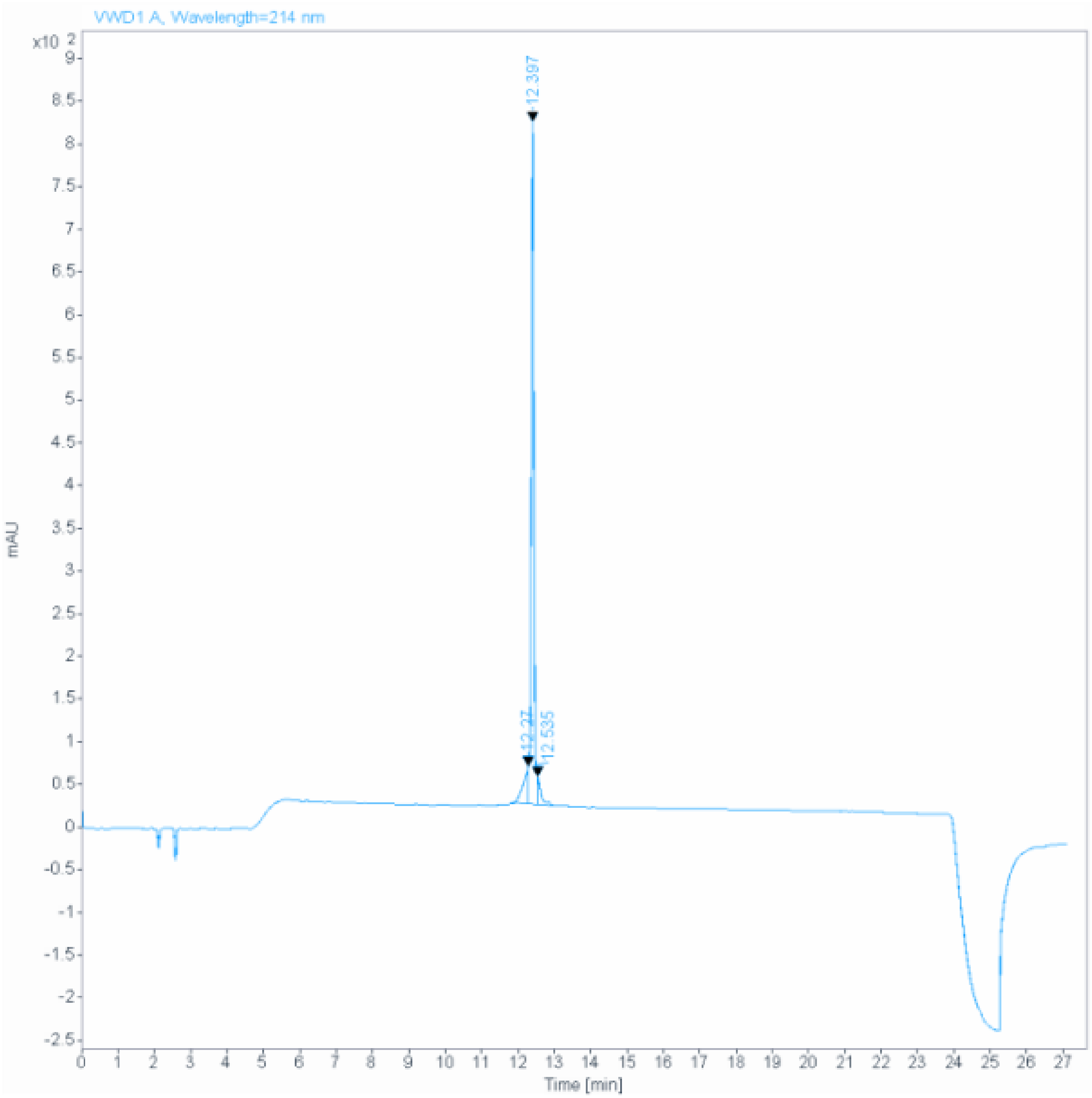

Signal: VWD1 A, Wavelength=214 nm

RT [min]	Type	Width [min]	Area	Height	Area%	Name
12.270	MF	0.1590	424.4482	44.4815	8.3924	
12.397	MF	0.0919	4412.6670	799.9210	87.2493	
12.535	FM	0.1100	220.4254	33.3902	4.3584	
		Sum	5057.5407			

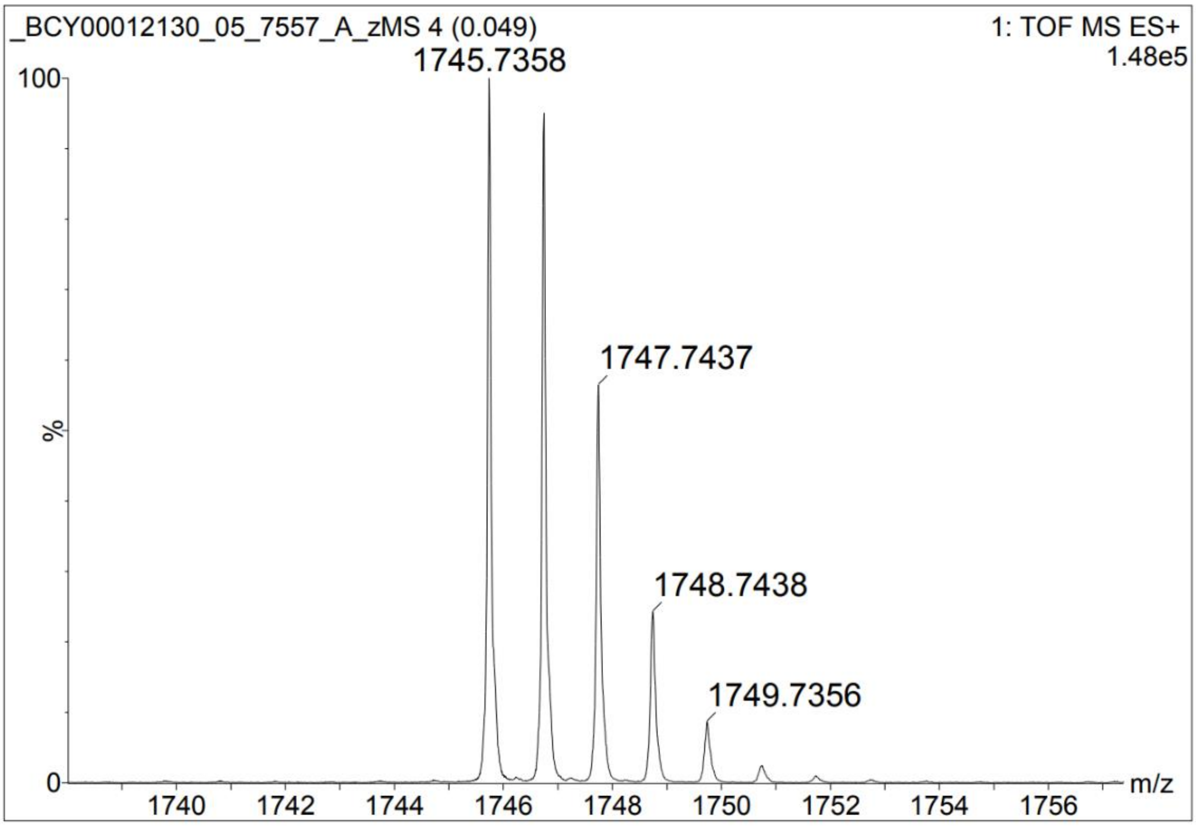

_BCY00012130_05_7557_A_zMS 4 (0.049)
1: TOF MS ES+
1.48e5
1745.7358
1747.7437
1748.7438
1749.7356
100
%
0
1740
1742
1744
1746
1748
1750
1752
1754
1756
m/z

Peptide 3

Data file: C:\Chem32\1\Data\Peptides\4800-4899_BCY00012131_01 4867 S1Cy1P1F1Z 2019-05-20 18-18-37.D

Sample name: _BCY00012131_01 4867 S1Cy1P1F1Z

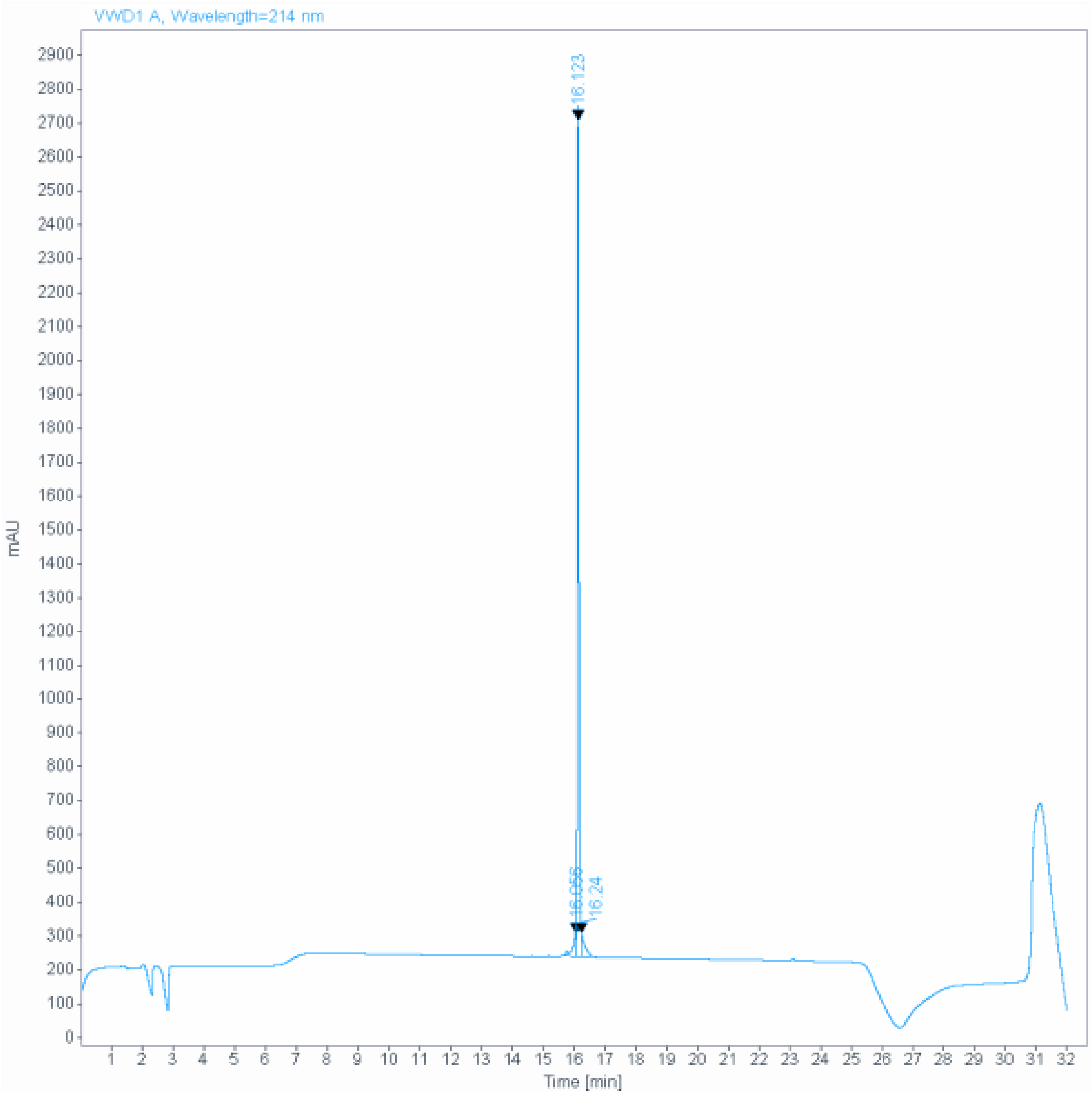

Signal: VWD1 A, Wavelength=214 nm

RT [min]	Type	Width [min]	Area	Height	Area%	Name
16.056	MF	0.1077	447.4658	69.2520	4.6339	
16.123	MF	0.0587	8704.0625	2472.5249	90.1375	
16.240	FM	0.1221	504.8945	68.8973	5.2286	
		Sum	9656.4228			

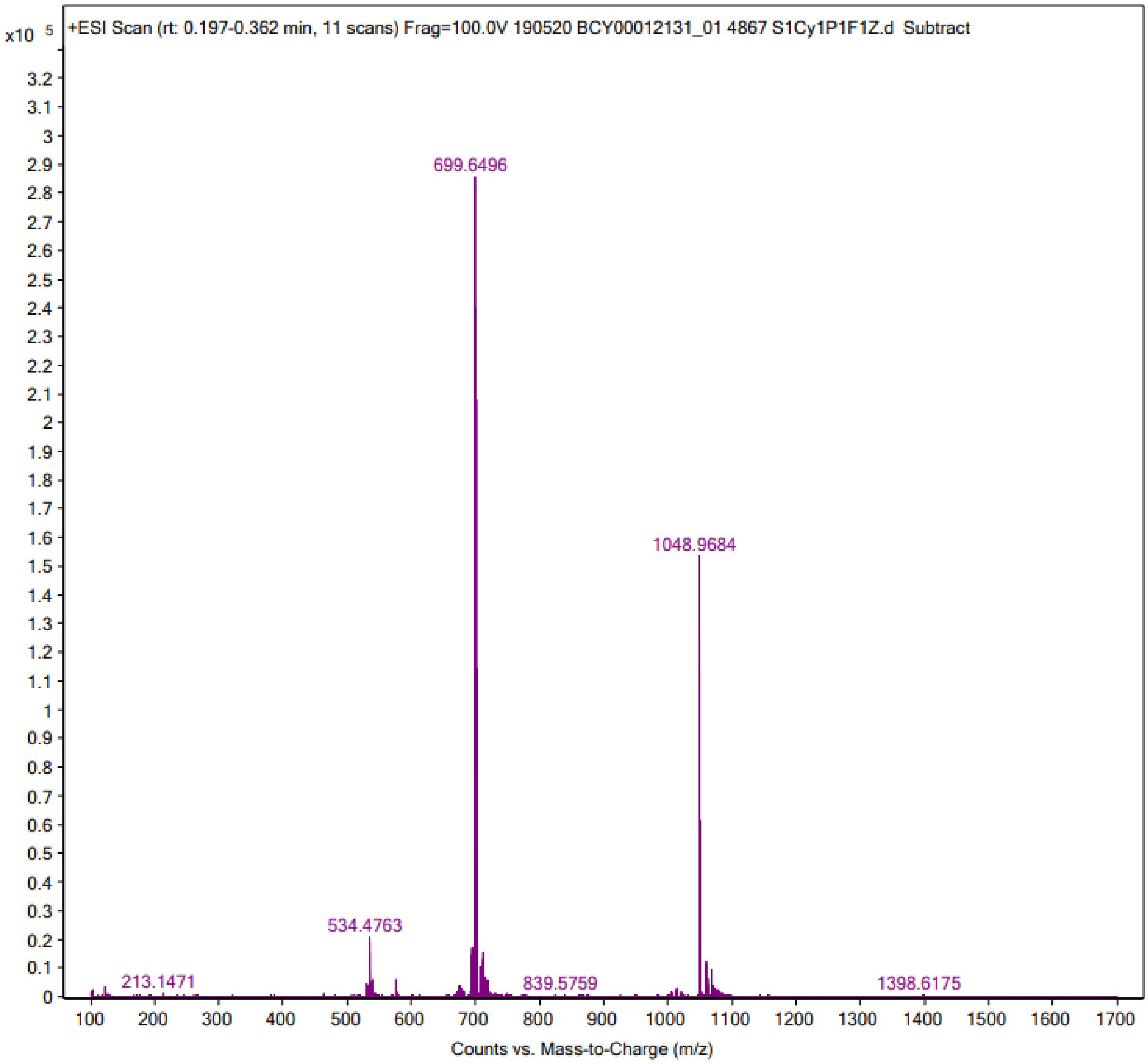

x10 5
+ESI Scan (rt: 0.197-0.362 min, 11 scans) Frag=100.0V 190520 BCY00012131_01 4867 S1Cy1P1F1Z.d Subtract
699.6496
1048.9684
534.4763
213.1471
839.5759
1398.6175
Counts vs. Mass-to-Charge (m/z)

Peptide 4

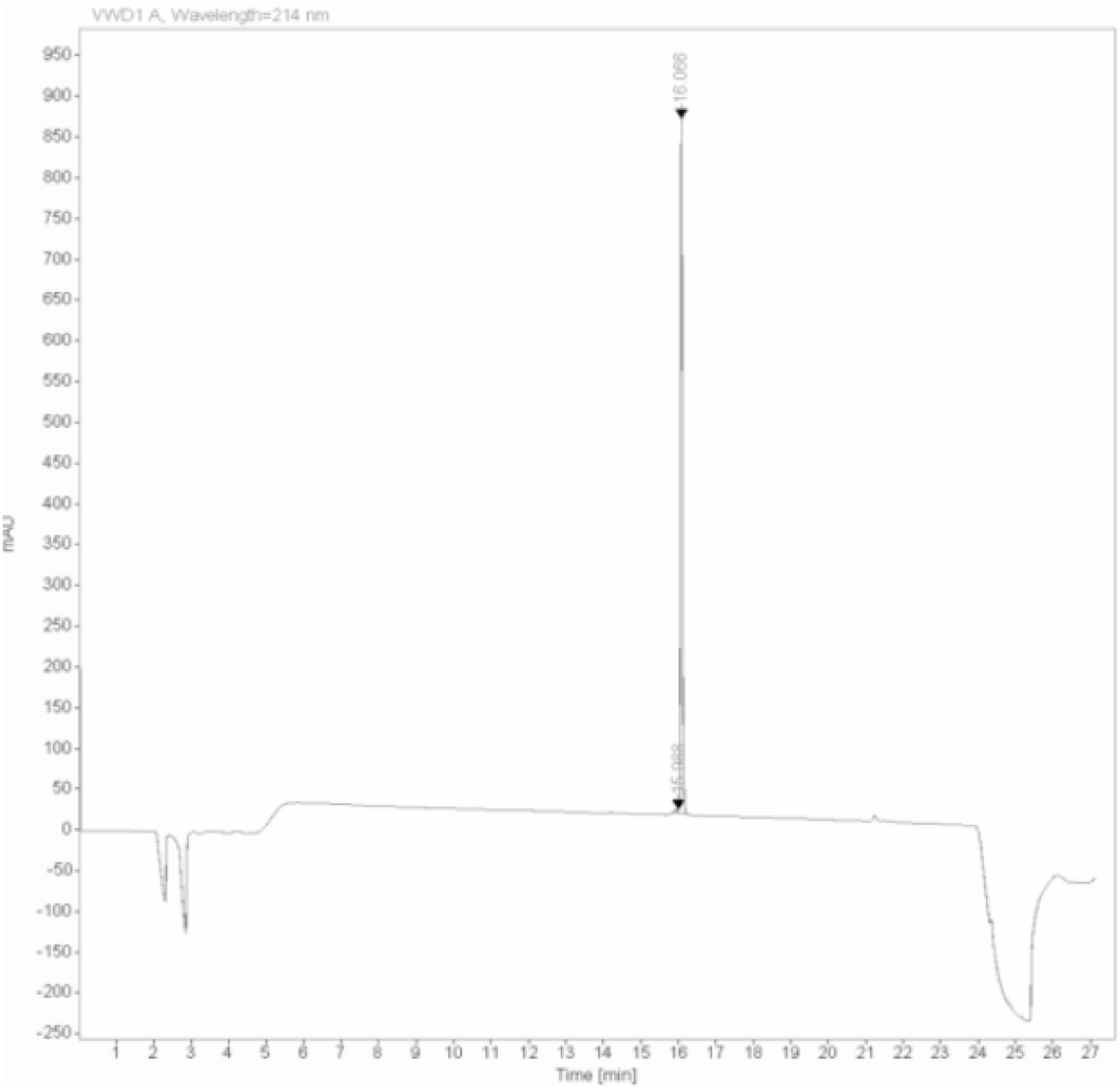

Signal: VWD1 A, Wavelength=214 nm

RT [min]	Type	Width [min]	Area	Height	Area%	Name
15.988	MF	0.0900	29.1654	5.4000	0.9969	
16.066	FM	0.0566	2896.3469	853.4639	99.0031	
		Sum	2925.5123			

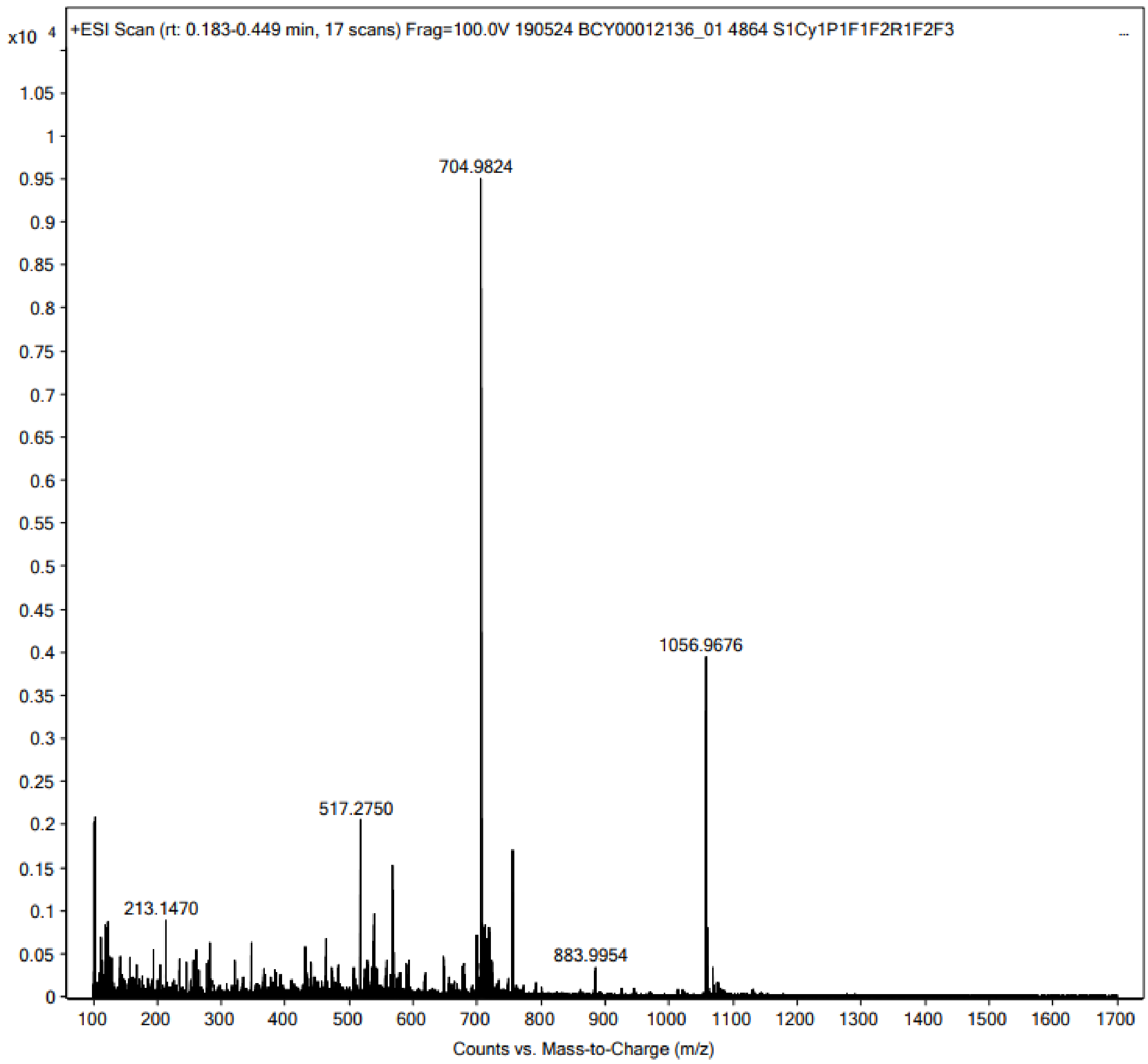

x10 4
+ESI Scan (rt: 0.183-0.449 min, 17 scans) Frag=100.0V 190524 BCY00012136_01 4864 S1Cy1P1F1F2R1F2F3
704.9824
1056.9676
517.2750
213.1470
883.9954
Counts vs. Mass-to-Charge (m/z)

Peptide 5

Data file: C:\Chem32\1\Data\Peptides\4800-4899_BCY00012132_01 4868 S1Cy1P1F1Z 2019-05-20 18-18-50.D

Sample name: _BCY00012132_01 4868 S1Cy1P1F1Z

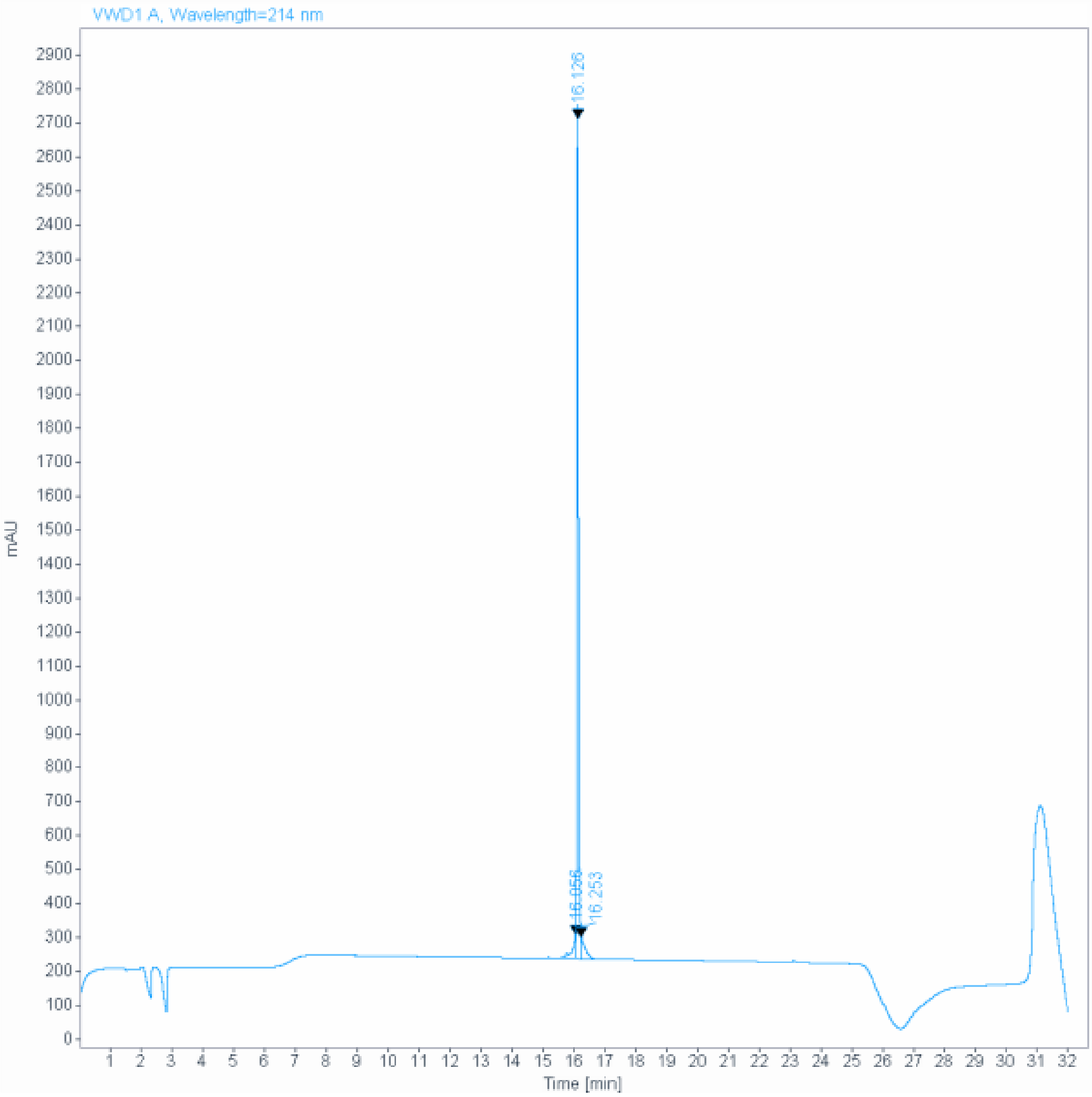

Signal: VWD1 A, Wavelength=214 nm

RT [min]	Type	Width [min]	Area	Height	Area%	Name
16.056	MF	0.1088	448.8487	68.7475	4.6234	
16.126	MF	0.0591	8772.6650	2475.7566	90.3627	
16.253	FM	0.1280	486.7630	63.3937	5.0139	
		Sum	9708.2768			

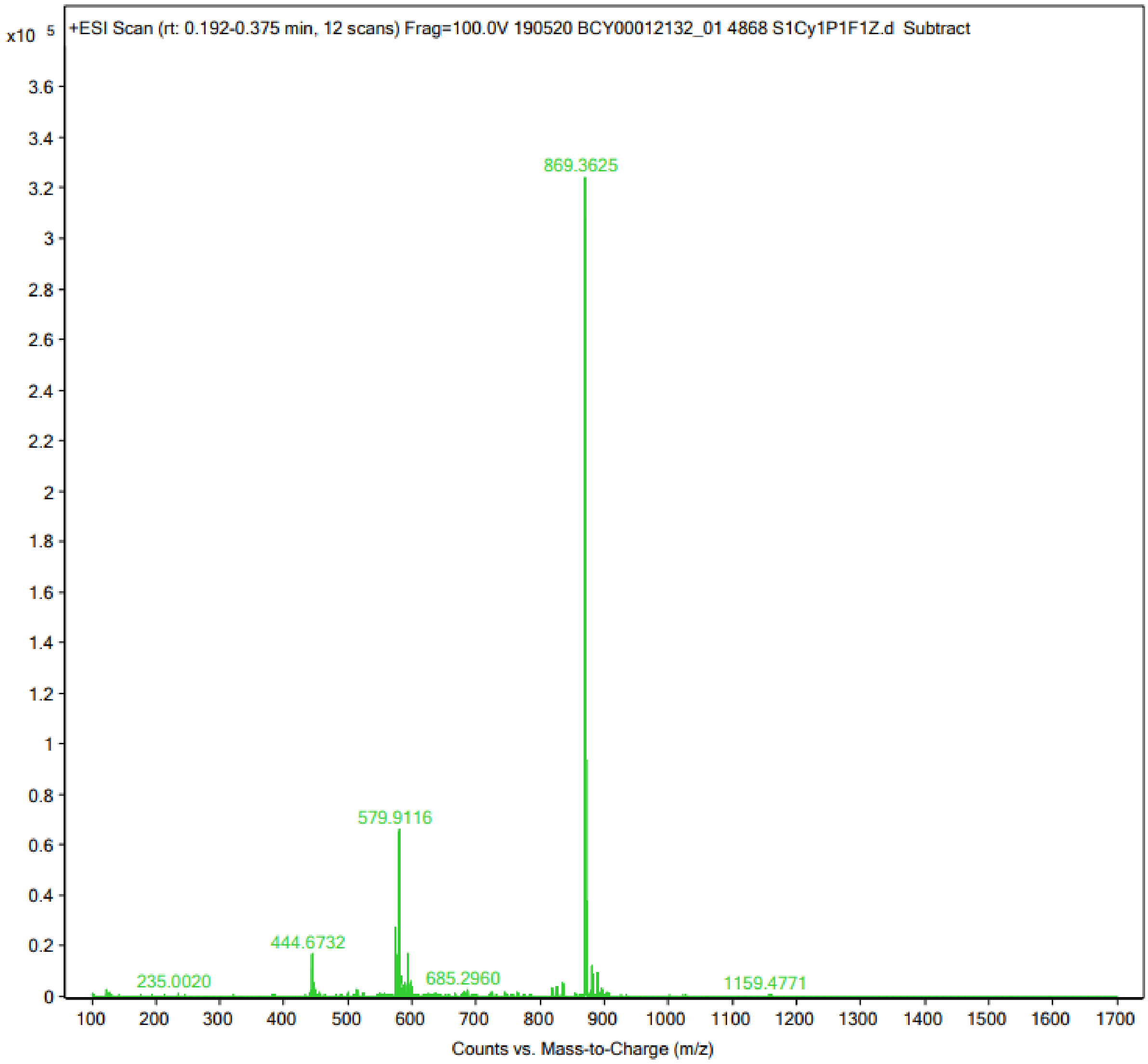

x10 5
+ESI Scan (rt: 0.192-0.375 min, 12 scans) Frag=100.0V 190520 BCY00012132_01 4868 S1Cy1P1F1Z.d Subtract
869.3625
579.9116
444.6732
235.0020
685.2960
1159.4771
Counts vs. Mass-to-Charge (m/z)

Peptide 6

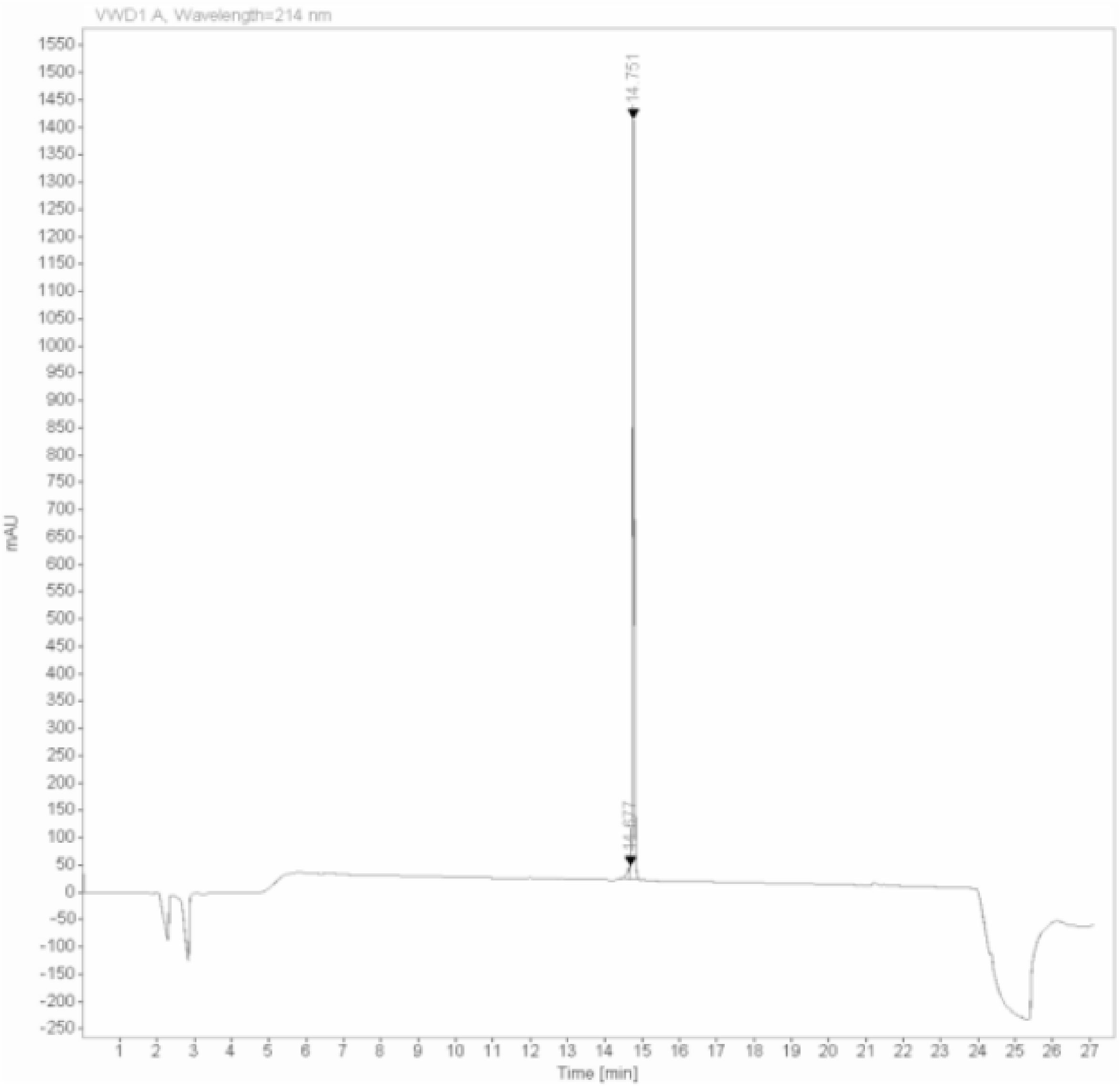

Signal: VWD1 A, Wavelength=214 nm

RT [min]	Type	Width [min]	Area	Height	Area%	Name
14.677	MF	0.0886	130.1320	24.4681	2.6617	
14.751	FM	0.0569	4758.9902	1392.9545	97.3383	
		Sum	4889.1222			

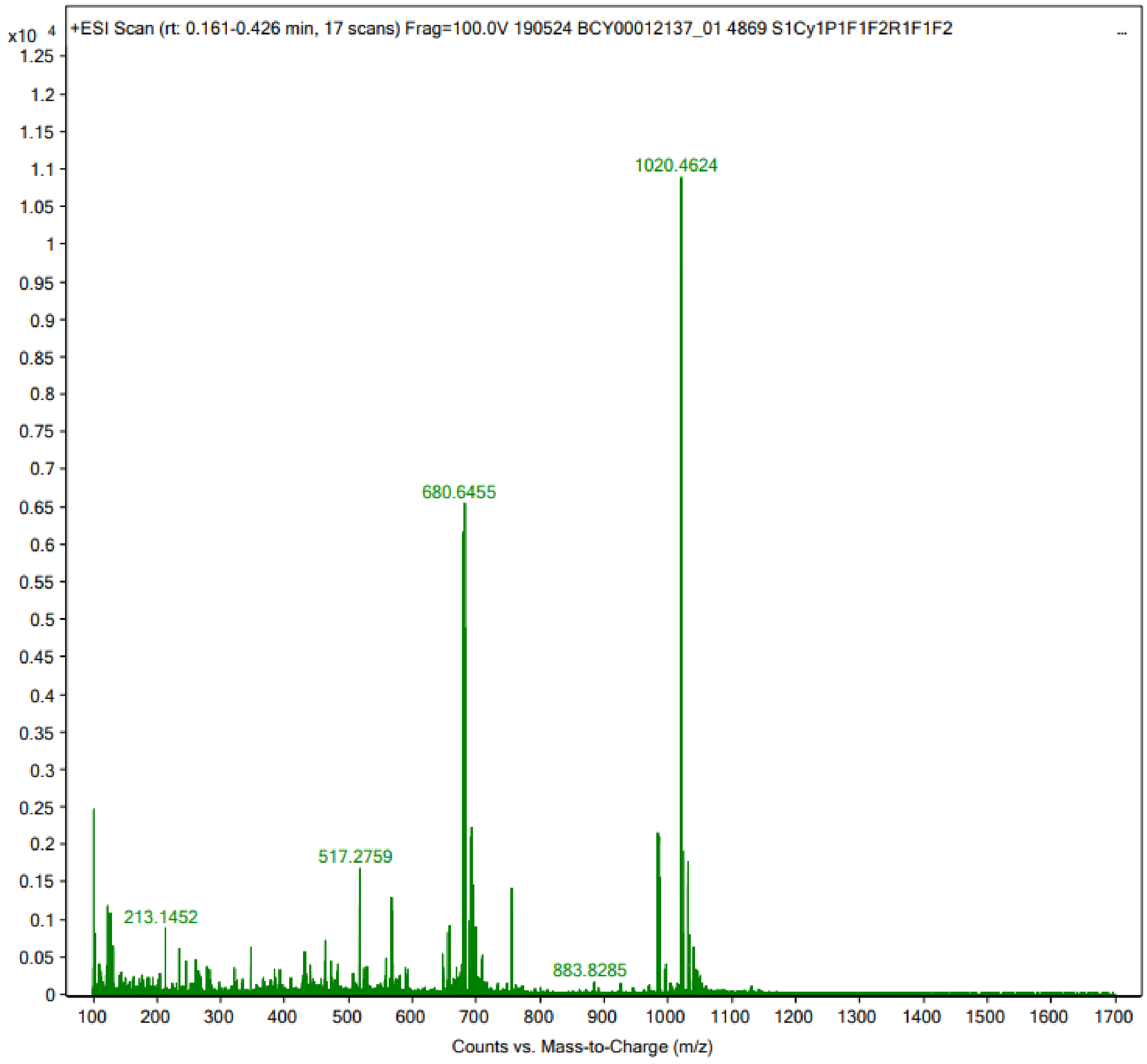

x10 4
+ESI Scan (rt: 0.161-0.426 min, 17 scans) Frag=100.0V 190524 BCY00012137_01 4869 S1Cy1P1F1F2R1F1F2
1020.4624
680.6455
517.2759
213.1452
883.8285
Counts vs. Mass-to-Charge (m/z)

Peptide 7

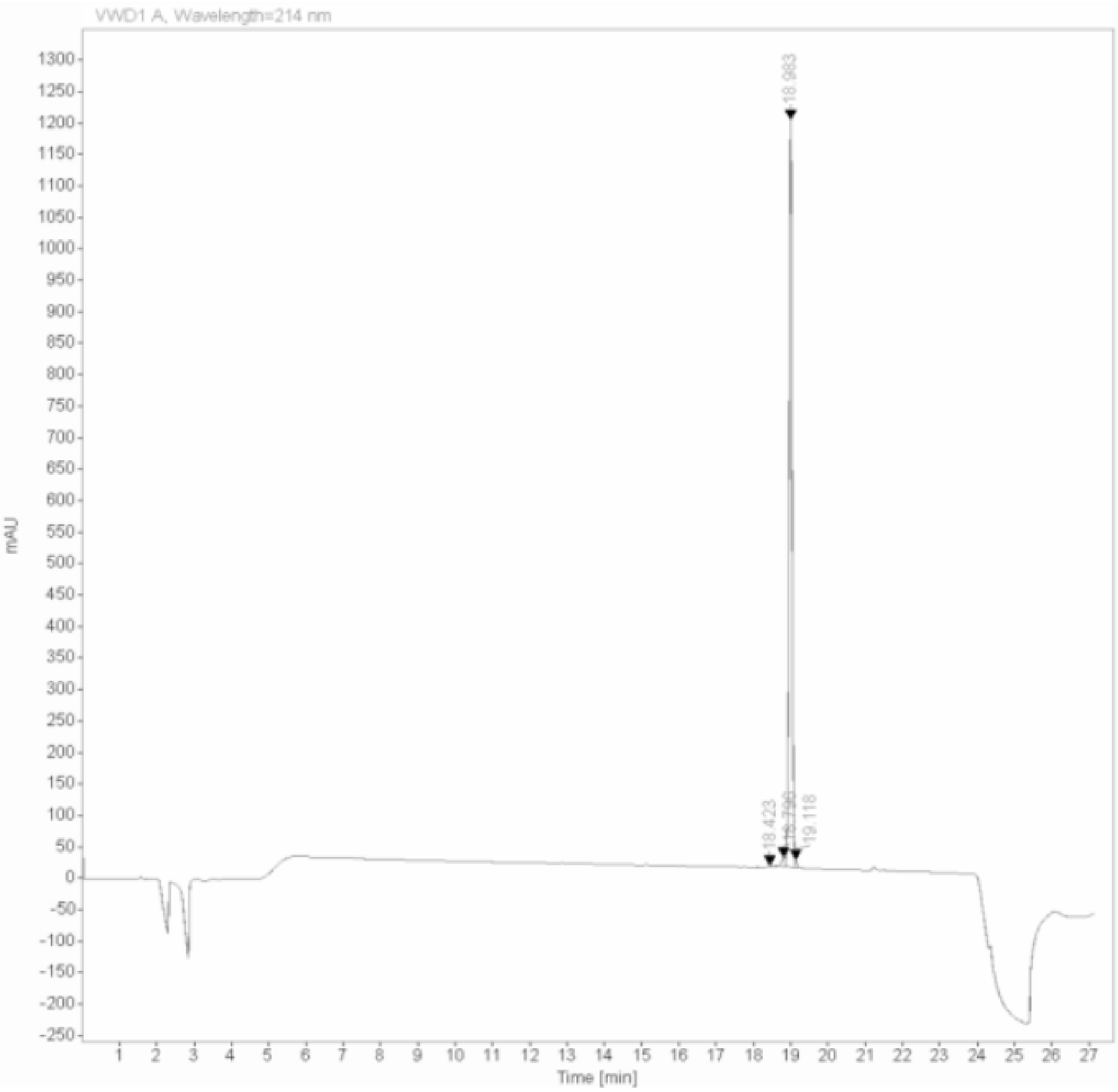

Signal: VWD1 A, Wavelength=214 nm

RT [min]	Type	Width [min]	Area	Height	Area%	Name
18.423	MM	0.0547	8.2297	2.5094	0.1208	
18.796	MF	0.1131	105.5613	15.5575	1.5490	
18.983	MF	0.0934	6663.8960	1188.6801	97.7828	
19.118	FM	0.0474	37.3108	13.1298	0.5475	
		Sum	6814.9978			

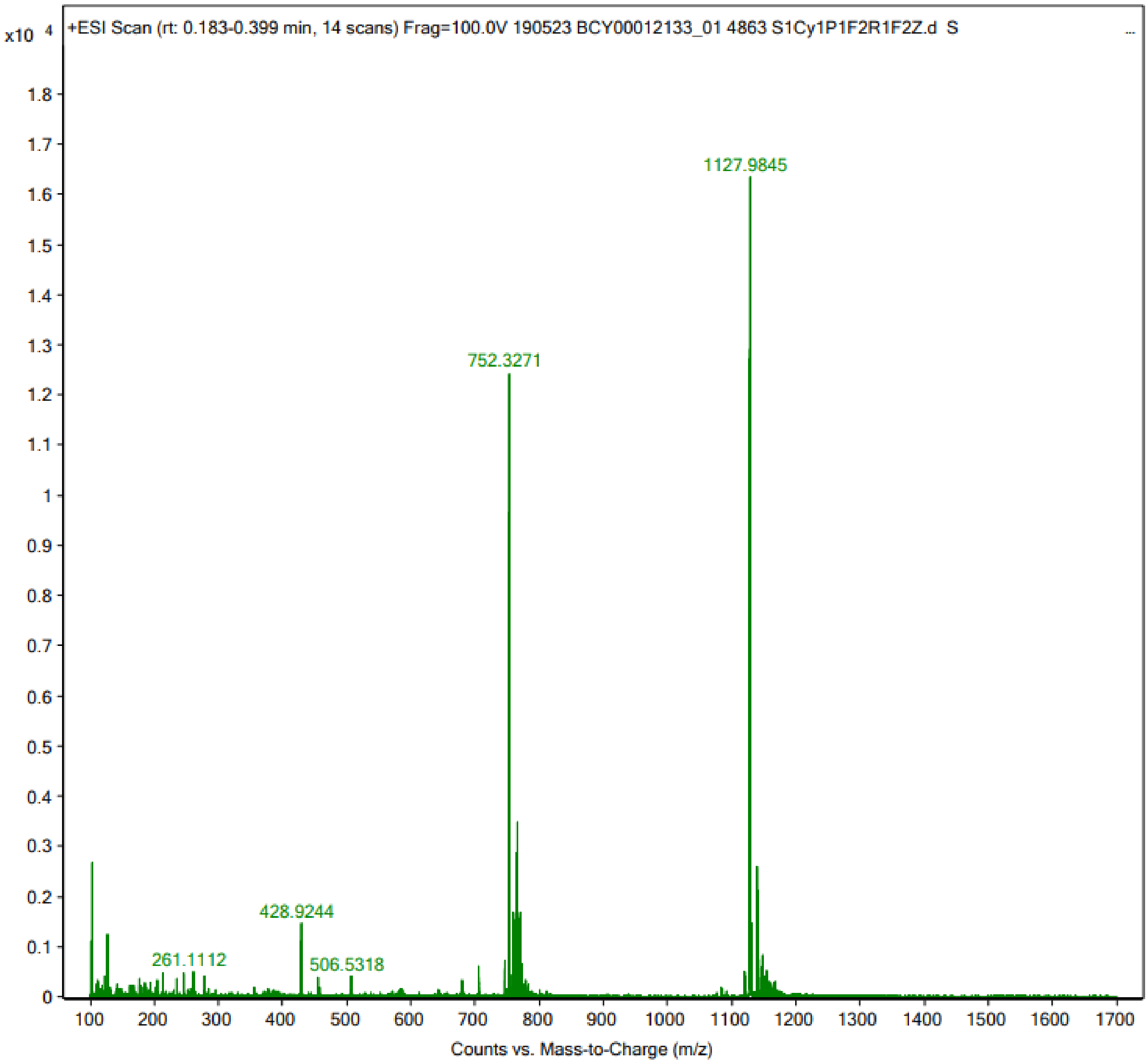

x10 4
+ESI Scan (rt: 0.183-0.399 min, 14 scans) Frag=100.0V 190523 BCY00012133_01 4863 S1Cy1P1F2R1F2Z.d S
1127.9845
752.3271
428.9244
261.1112
506.5318
Counts vs. Mass-to-Charge (m/z)

Peptide 8

Data file: Y:\Cunegonde\Chem32\1\Data\Peptides\4800-4899_BCY-00012125_01 4843 S1Cy1P1F1Z 2019-05-03 18-08-28.D

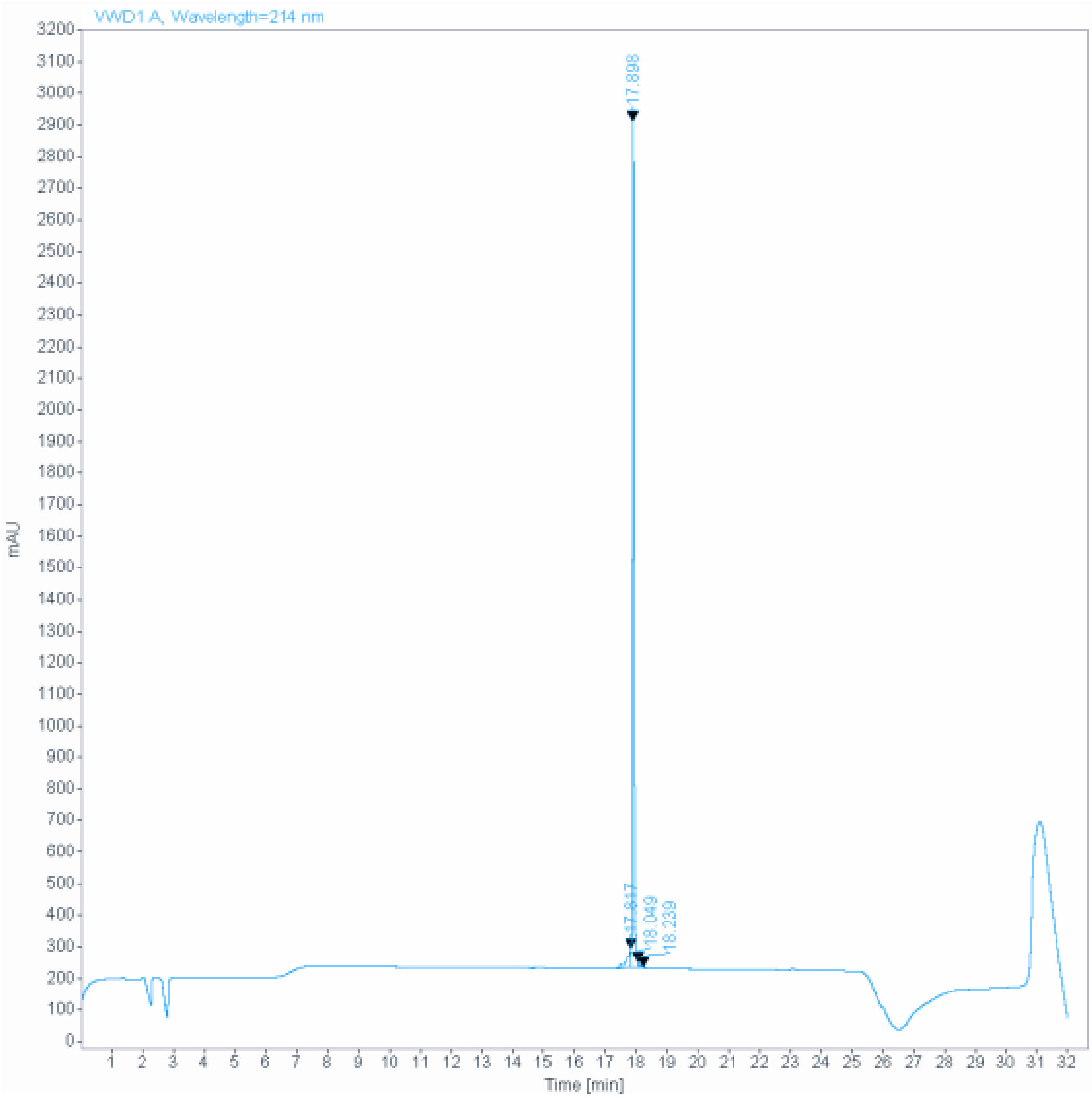

Signal: VWD1 A, Wavelength=214 nm

RT [min]	Type	Width [min]	Area	Height	Area%	Name
17.817	MF	0.1405	490.2820	58.1721	4.9663	
17.898	MF	0.0579	9314.9258	2680.0886	94.3553	
18.049	FM	0.0592	62.0055	17.4606	0.6281	
18.239	MM	0.0497	4.9650	1.6666	0.0503	
		Sum	9872.1783			

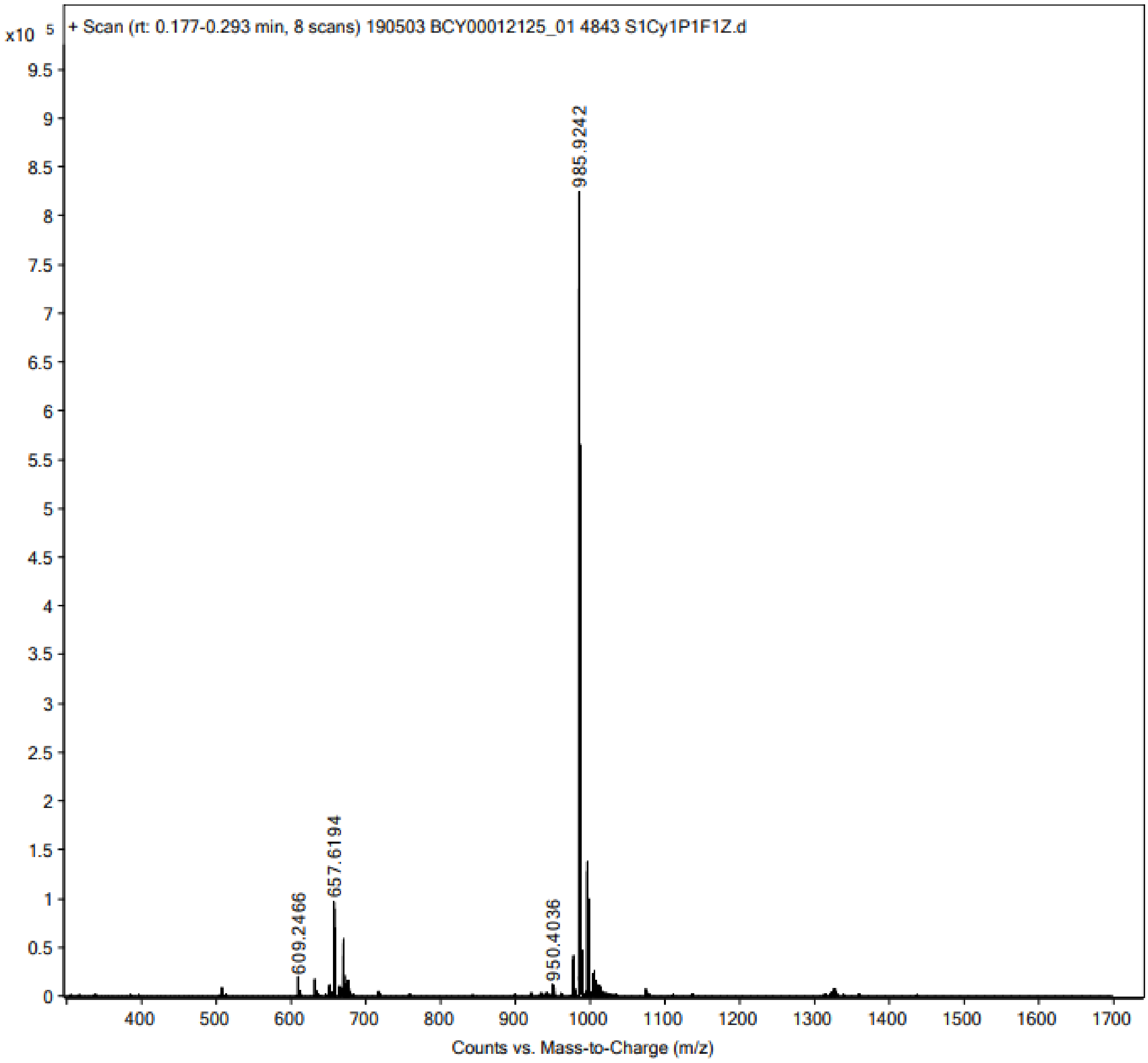

x10 5
+ Scan (rt: 0.177-0.293 min, 8 scans) 190503 BCY00012125_01 4843 S1Cy1P1F1Z.d
985.9242
657.6194
609.2466
950.4036
Counts vs. Mass-to-Charge (m/z)

Peptide 9

Data file: Y:\Cunegonde\Chem32\1\Data\Peptides\4800-4899_BCY-00012124_01 4842 S1Cy1P1F1Z 2019-05-03 18-07-57.D

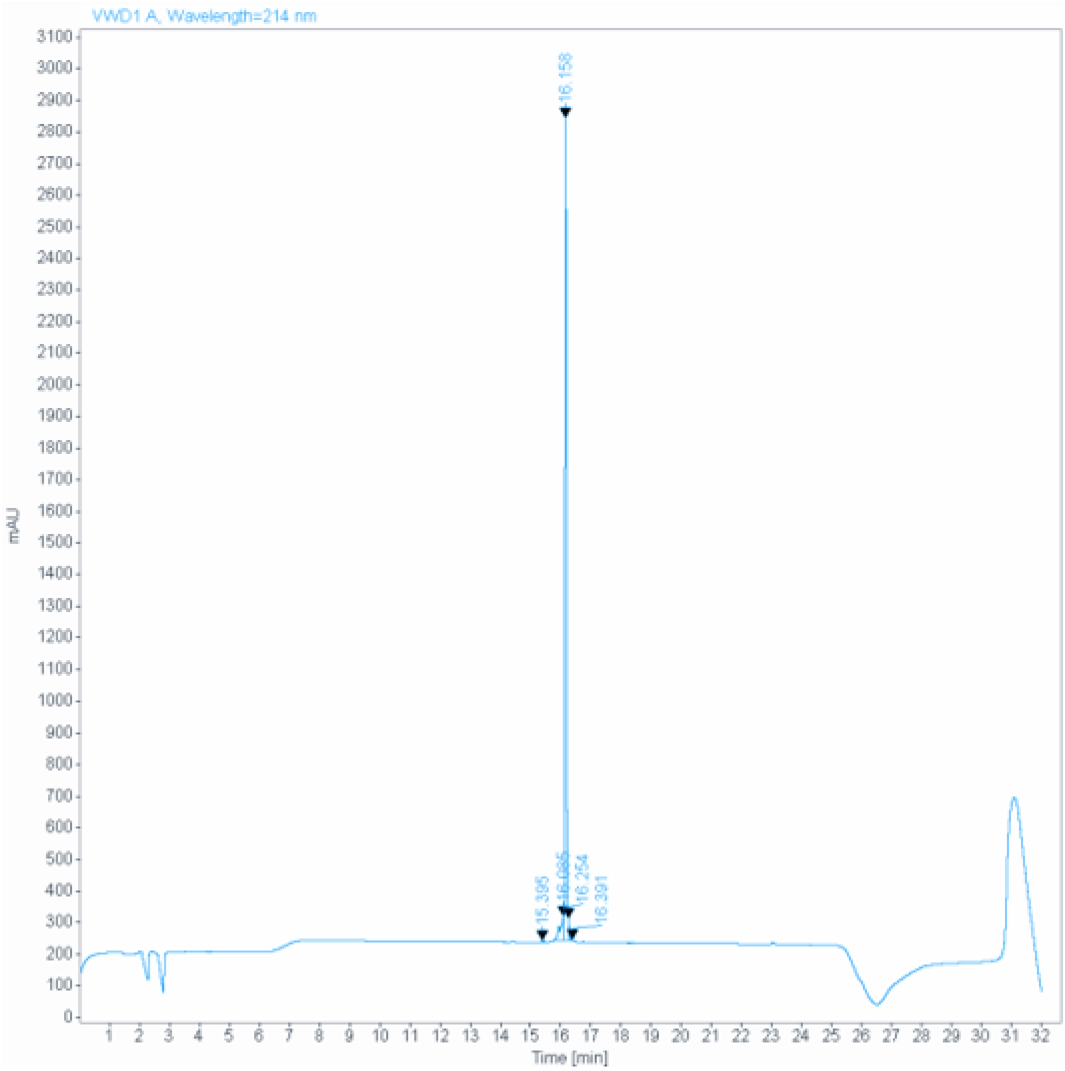

Signal: VWD1 A, Wavelength=214 nm

RT [min]	Type	Width [min]	Area	Height	Area%	Name
15.395	VV R	0.0632	15.6337	3.7458	0.1671	
16.085	MF	0.1321	591.7968	74.6883	6.3262	
16.158	MF	0.0553	8624.3271	2601.4370	92.1927	
16.254	FM	0.0269	109.8465	68.1846	1.1742	
16.391	MM	0.0430	13.0673	5.0638	0.1397	
		Sum	9354.6715			

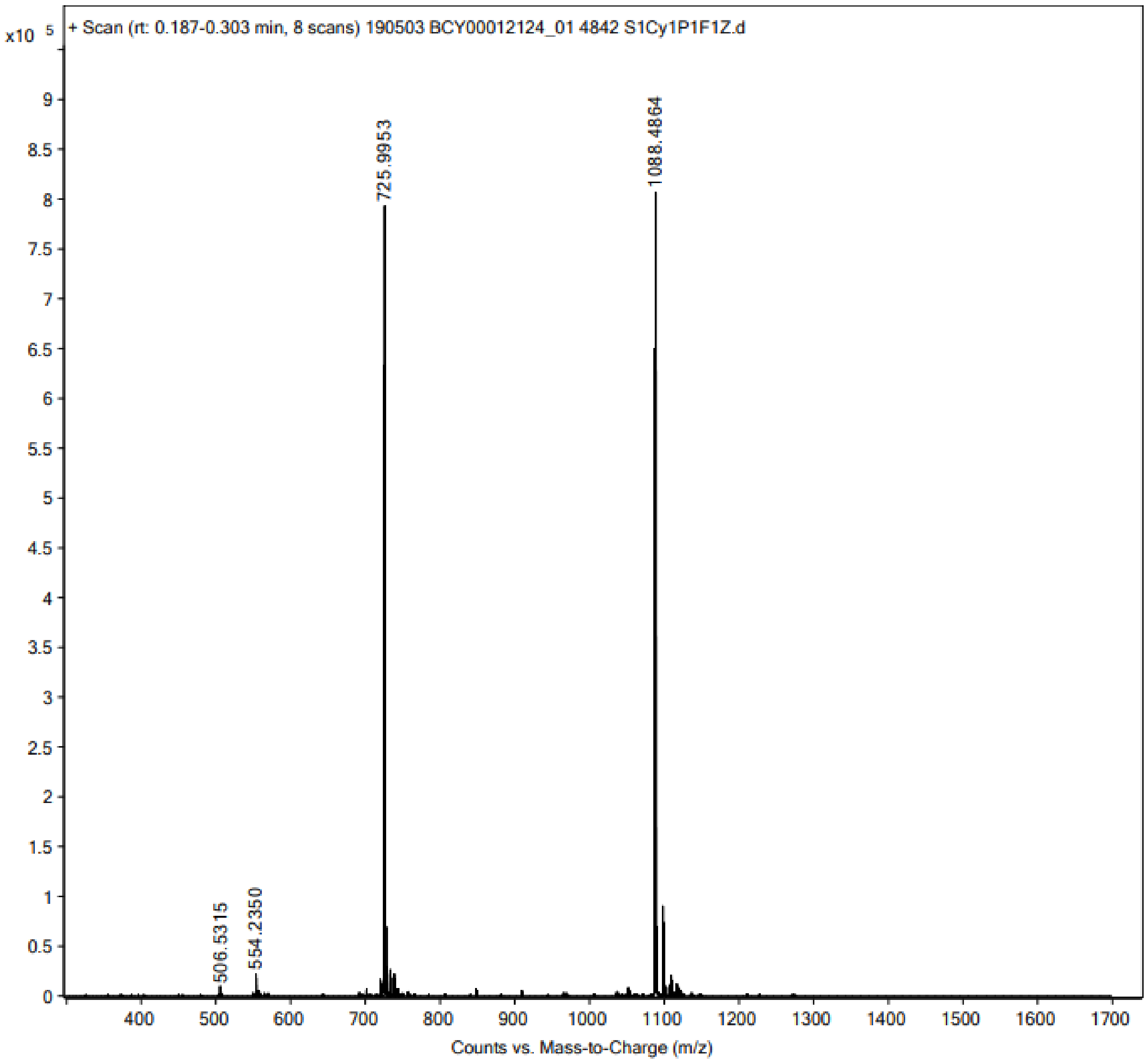
+ Scan (rt: 0.187-0.303 min, 8 scans) 190503 BCY00012124_01 4842 S1Cy1P1F1Z.d
x10 5
725.9953
1088.4864
506.5315
554.2350
Counts vs. Mass-to-Charge (m/z)

Peptide 10

Data file: C:\Chem32\1\Data\Peptides\4800-4899_BCY00012135_01 4865 S1Cy1P1F1F2Z 2019-05-20 18-18-18.D

Sample name: _BCY00012135_01 4865 S1Cy1P1F1F2Z

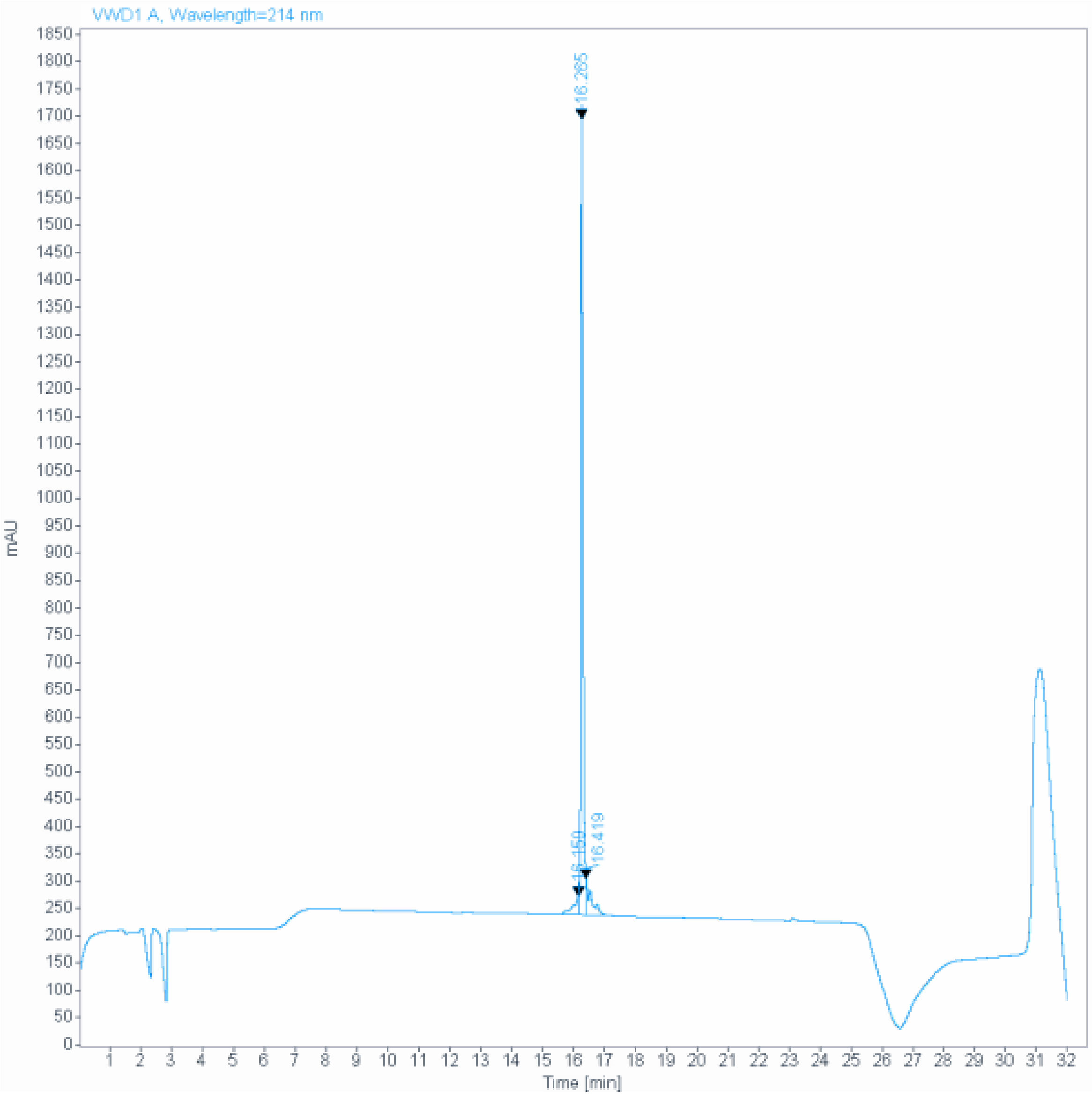

Signal: VWD1 A, Wavelength=214 nm

RT [min]	Type	Width [min]	Area	Height	Area%	Name
16.159	MF	0.1862	373.6817	33.4513	4.7803	
16.265	MF	0.0773	6764.0708	1458.1731	86.5283	
16.419	FM	0.1727	679.4210	65.5497	8.6914	
		Sum	7817.1735			

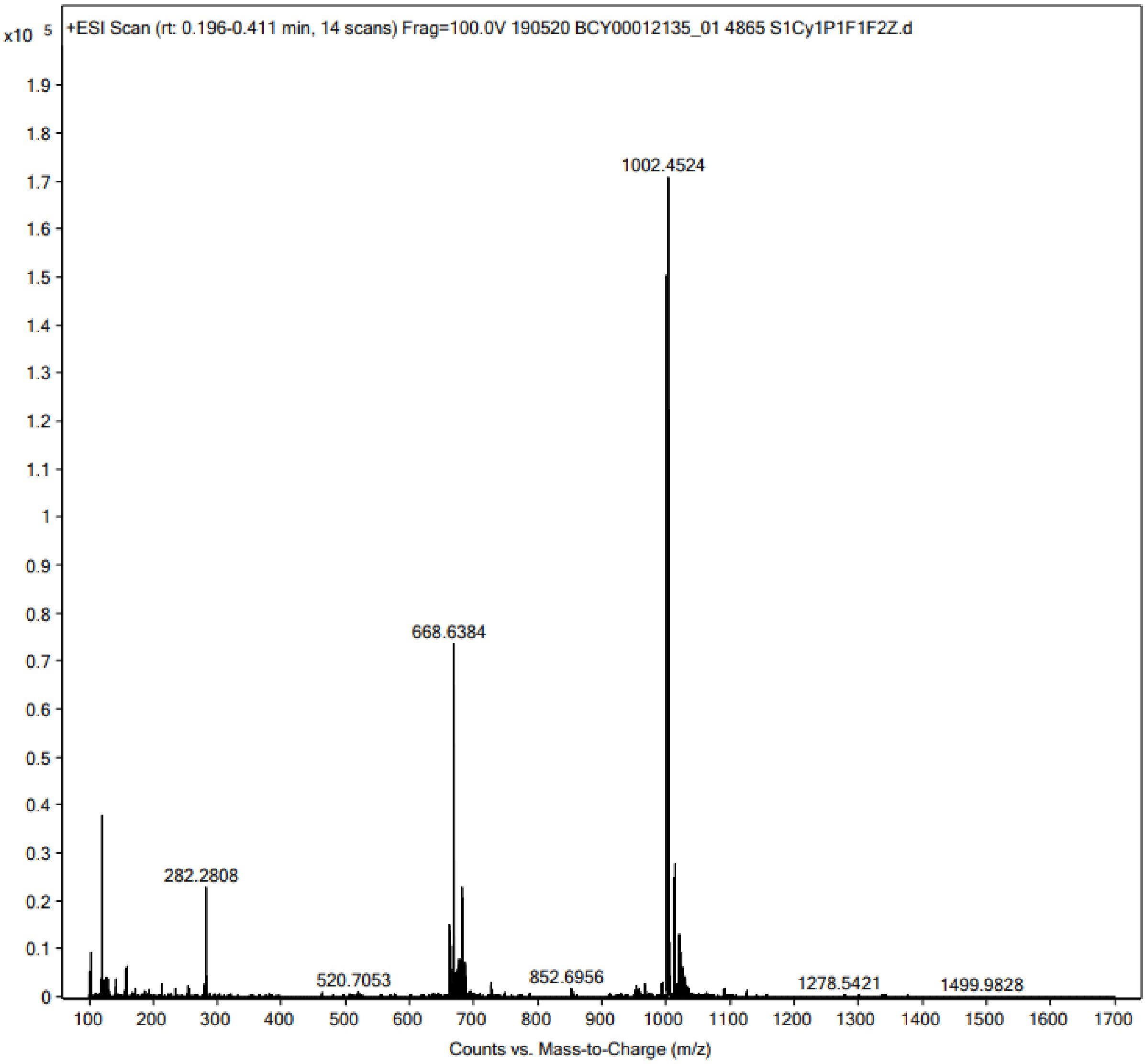
x10 5
+ESI Scan (rt: 0.196-0.411 min, 14 scans) Frag=100.0V 190520 BCY00012135_01 4865 S1Cy1P1F1F2Z.d
1.9
1.8
1.7
1.6
1.5
1.4
1.3
1.2
1.1
1
0.9
0.8
0.7
0.6
0.5
0.4
0.3
0.2
0.1
0
1002.4524
668.6384
282.2808
520.7053
852.6956
1278.5421
1499.9828
100
200
300
400
500
600
700
800
900
1000
1100
1200
1300
1400
1500
1600
1700
Counts vs. Mass-to-Charge (m/z)

Peptide 11

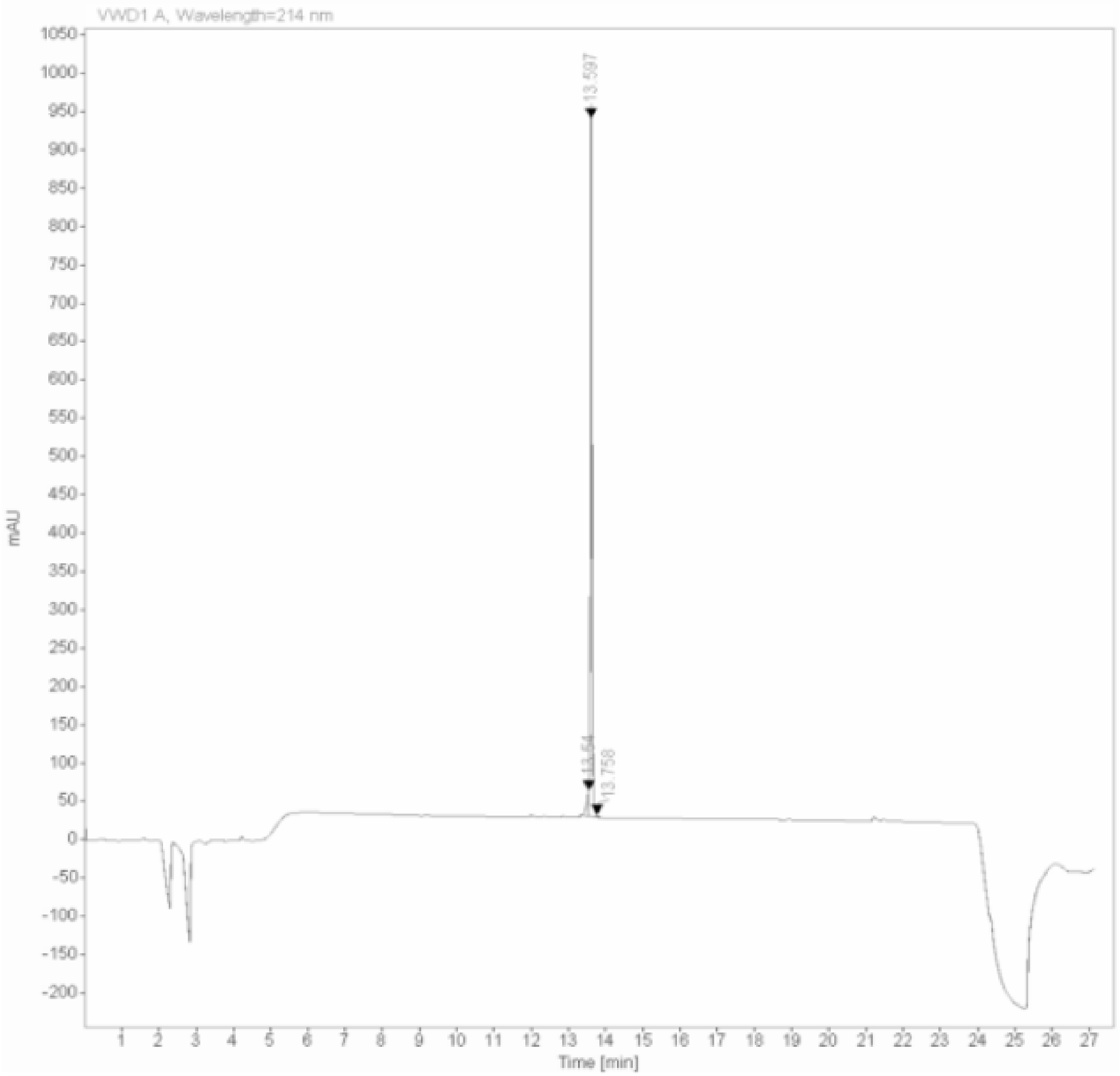

Signal: VWD1 A, Wavelength=214 nm

RT [min]	Type	Width [min]	Area	Height	Area%	Name
13.540	MF	0.0685	143.5043	34.9315	4.8870	
13.597	FM	0.0507	2773.6589	912.5562	94.4570	
13.758	FM	0.0673	19.2617	4.7713	0.6560	
		Sum	2936.4249			

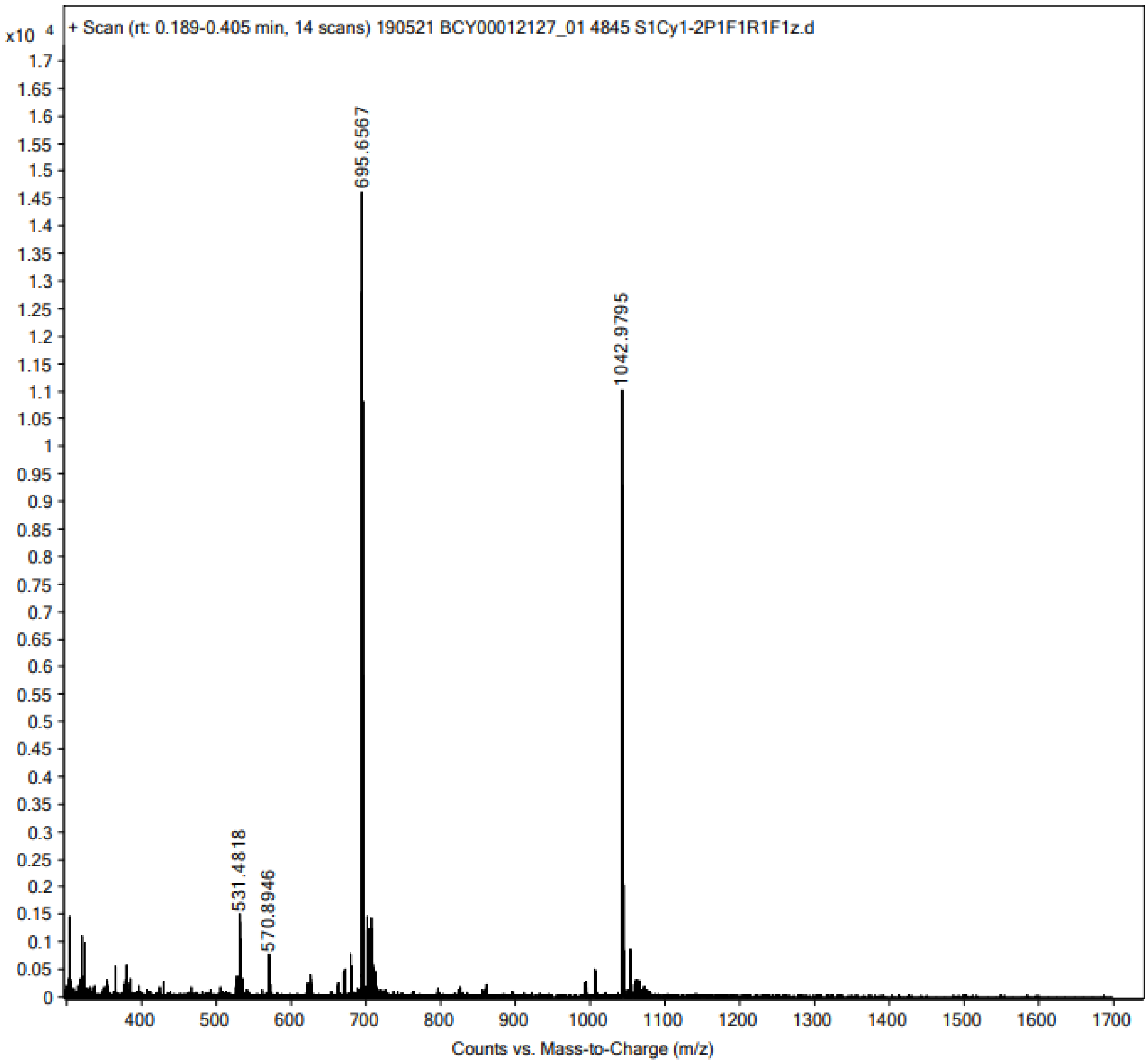
+ Scan (rt: 0.189-0.405 min, 14 scans) 190521 BCY00012127_01 4845 S1Cy1-2P1F1R1F1z.d
x10 4
695.6567
1042.9795
531.4818
570.8946
Counts vs. Mass-to-Charge (m/z)

Peptide 12

Data file: C:\Chem32\1\Data\Peptides\4800-4899_BCY00012134_01 4861 S1Cy1P1F1Z 2019-05-20 18-17-43.D

Sample name: _BCY00012134_01 4861 S1Cy1P1F1Z

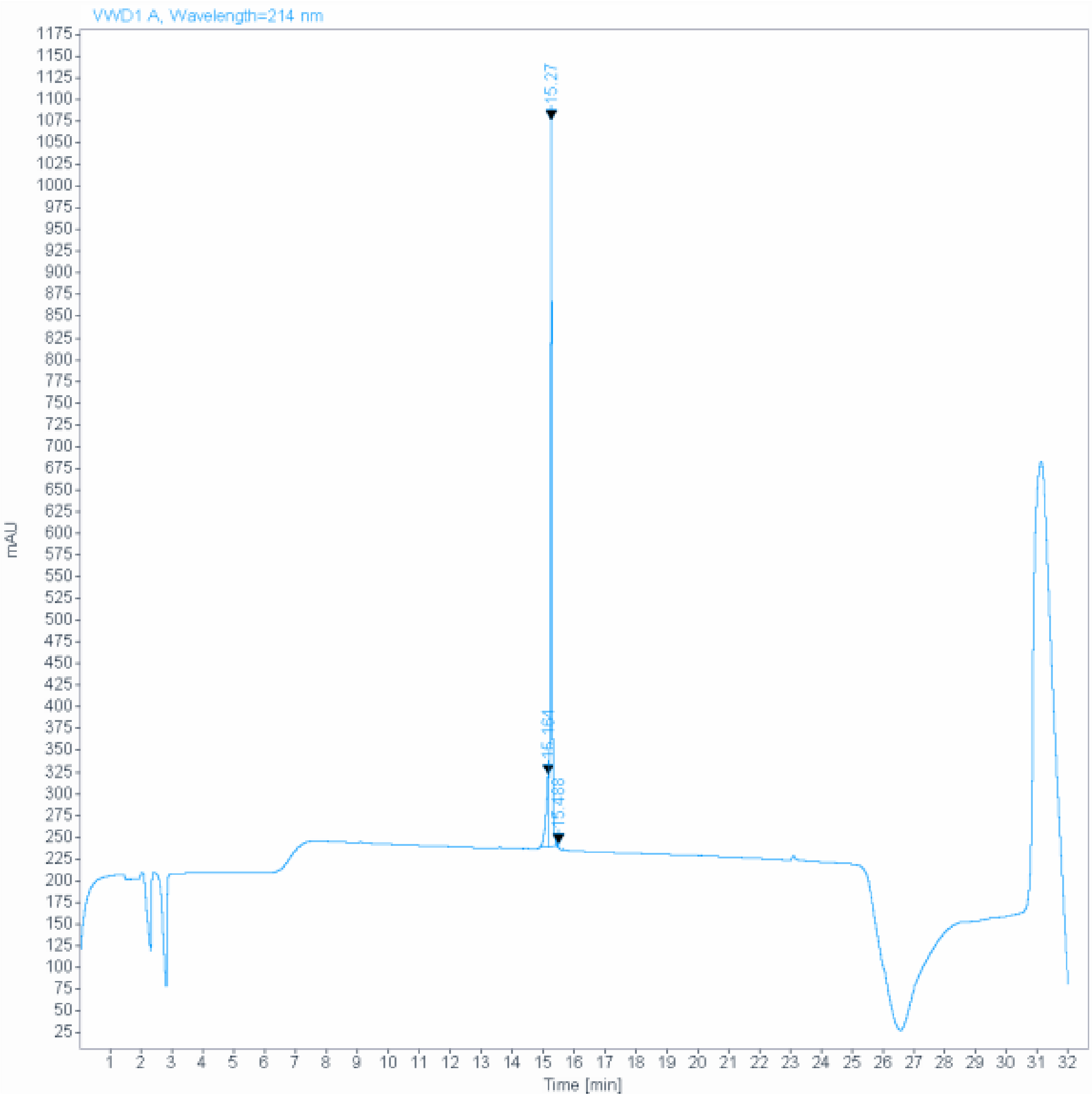

Signal: VWD1 A, Wavelength=214 nm

RT [min]	Type	Width [min]	Area	Height	Area%	Name
15.164	MF	0.1023	511.0786	83.2671	14.0753	
15.270	FM	0.0618	3108.8955	838.0609	85.6204	
15.488	MM	0.0458	11.0467	4.0182	0.3042	
		Sum	3631.0207			

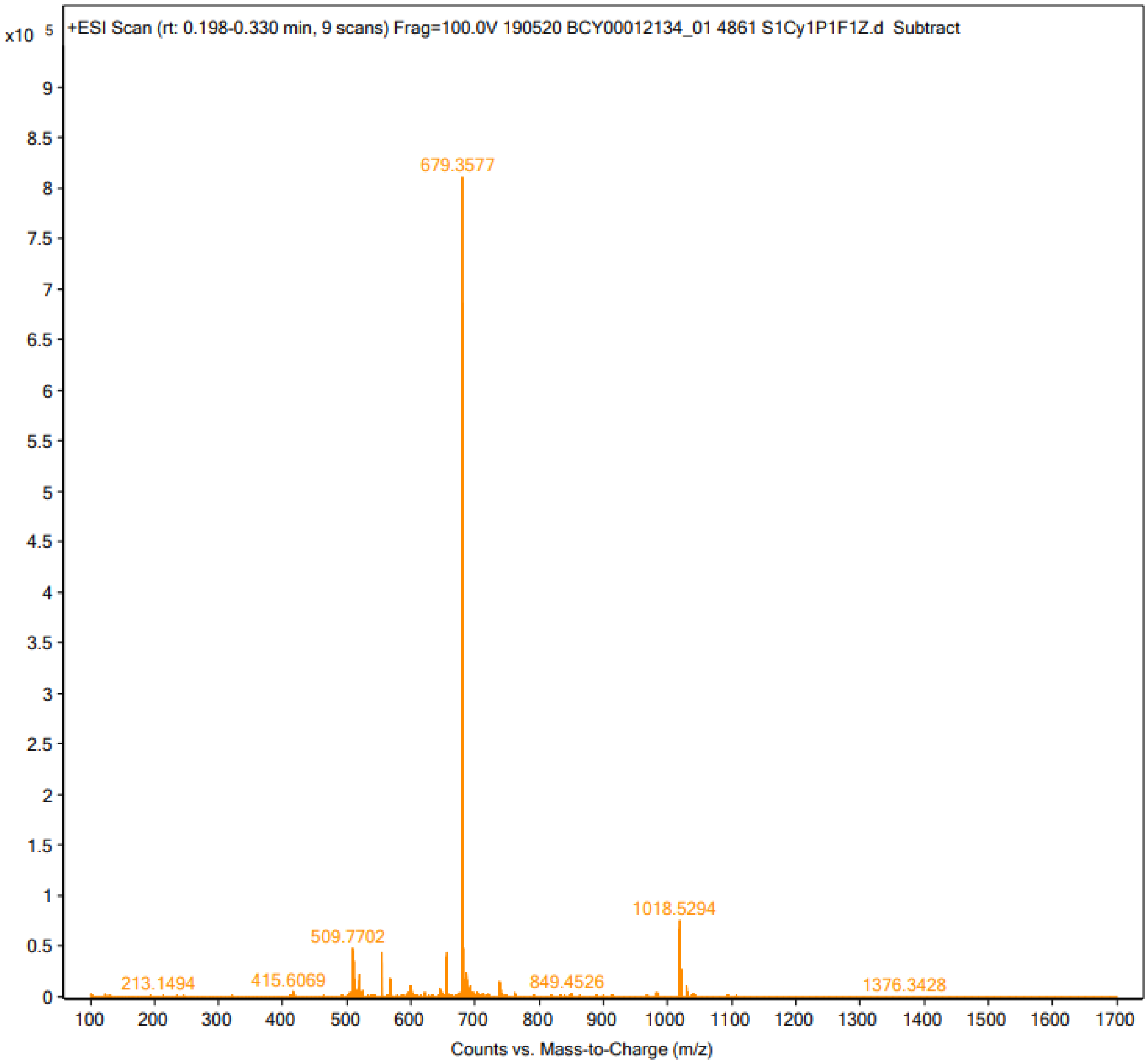
x10 5
+ESI Scan (rt: 0.198-0.330 min, 9 scans) Frag=100.0V 190520 BCY00012134_01 4861 S1Cy1P1F1Z.d Subtract
679.3577
1018.5294
509.7702
213.1494
415.6069
849.4526
1376.3428
Counts vs. Mass-to-Charge (m/z)

Peptide 13

Data file: Y:\Cunegonde\Chem32\1\Data\Peptides\4800-4899_BCY00012123_01 4841 S1Cy1P1F1z 2019-05-02 17-25-45.D

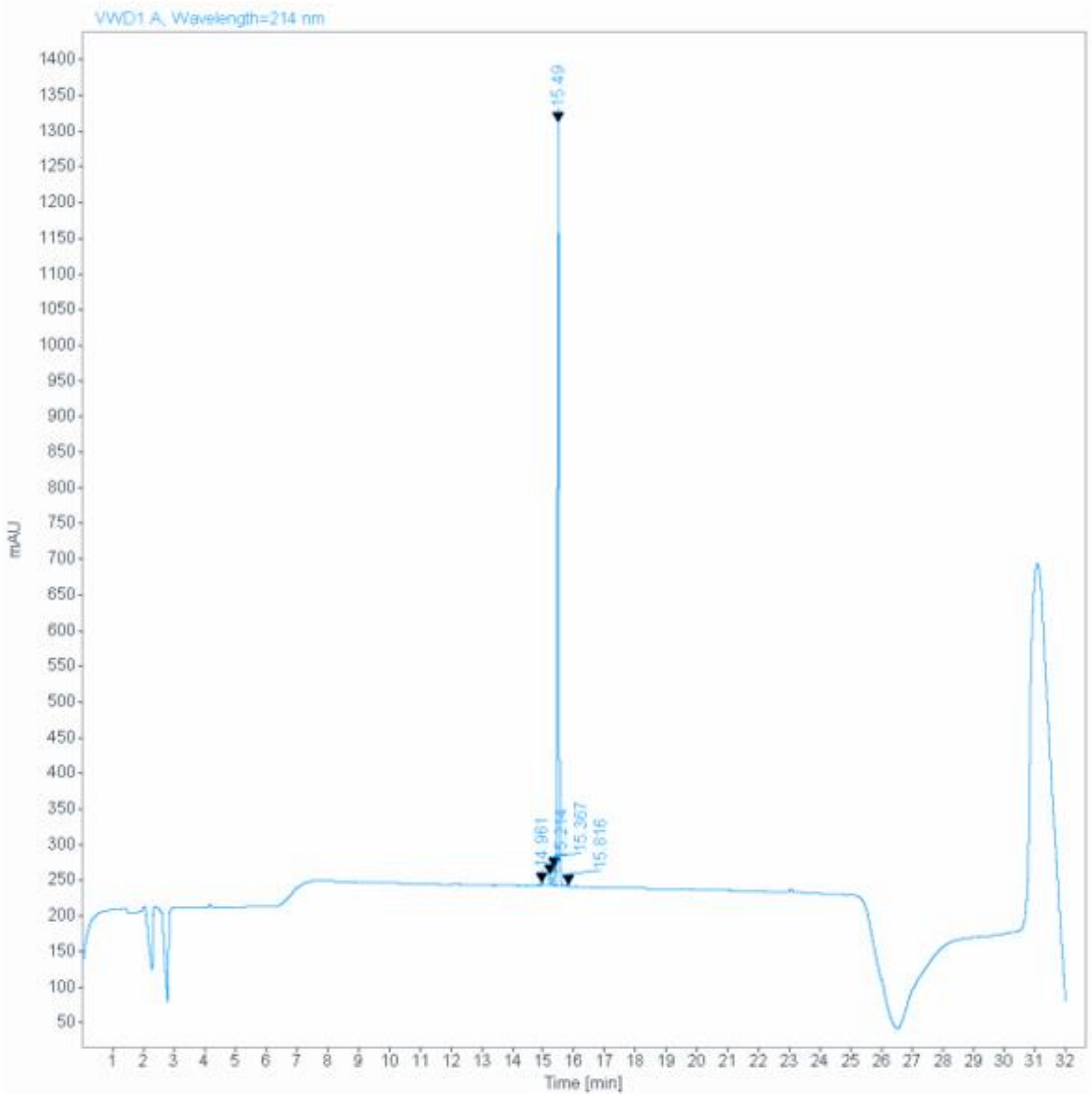

Signal: VWD1 A, Wavelength=214 nm

RT [min]	Type	Width [min]	Area	Height	Area%	Name
14.961	VB	0.0630	13.6901	3.1022	0.3796	
15.214	BV E	0.0557	55.0858	15.0598	1.5273	
15.367	VV E	0.0784	126.9883	25.1406	3.5208	
15.490	VB R	0.0486	3402.8162	1070.3527	94.3445	
15.816	VB	0.0464	8.2186	2.7497	0.2279	
		Sum	3606.7990			

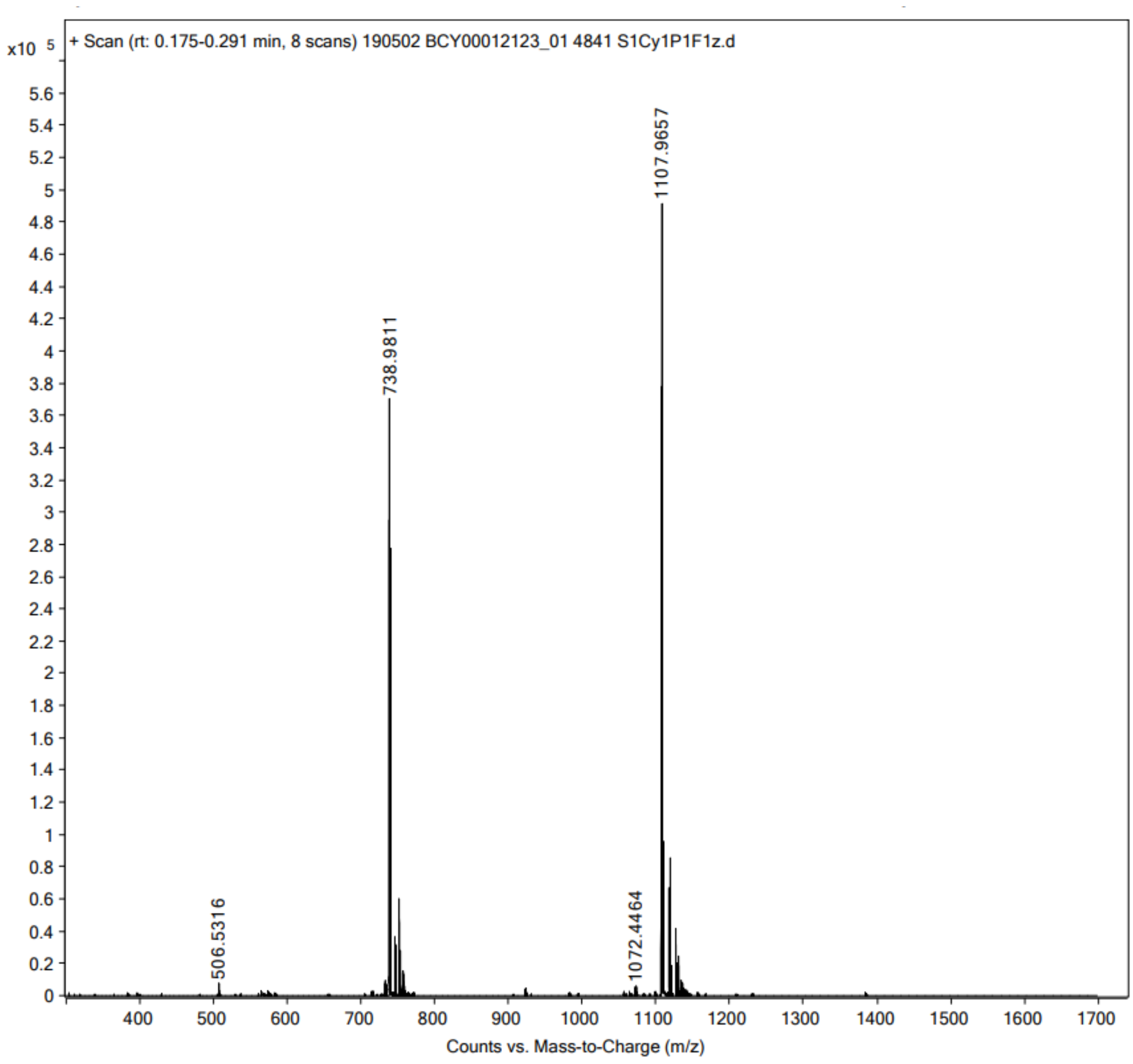
x10 5
+ Scan (rt: 0.175-0.291 min, 8 scans) 190502 BCY00012123_01 4841 S1Cy1P1F1z.d
1107.9657
738.9811
506.5316
1072.4464
Counts vs. Mass-to-Charge (m/z)

Peptide 14

Data file: D:\Chemstation\1\Data\Peptides\4800-4899_BCY00012126_01 4844 S1Cy1P1F1z 2019-05-16 15-45-06.D

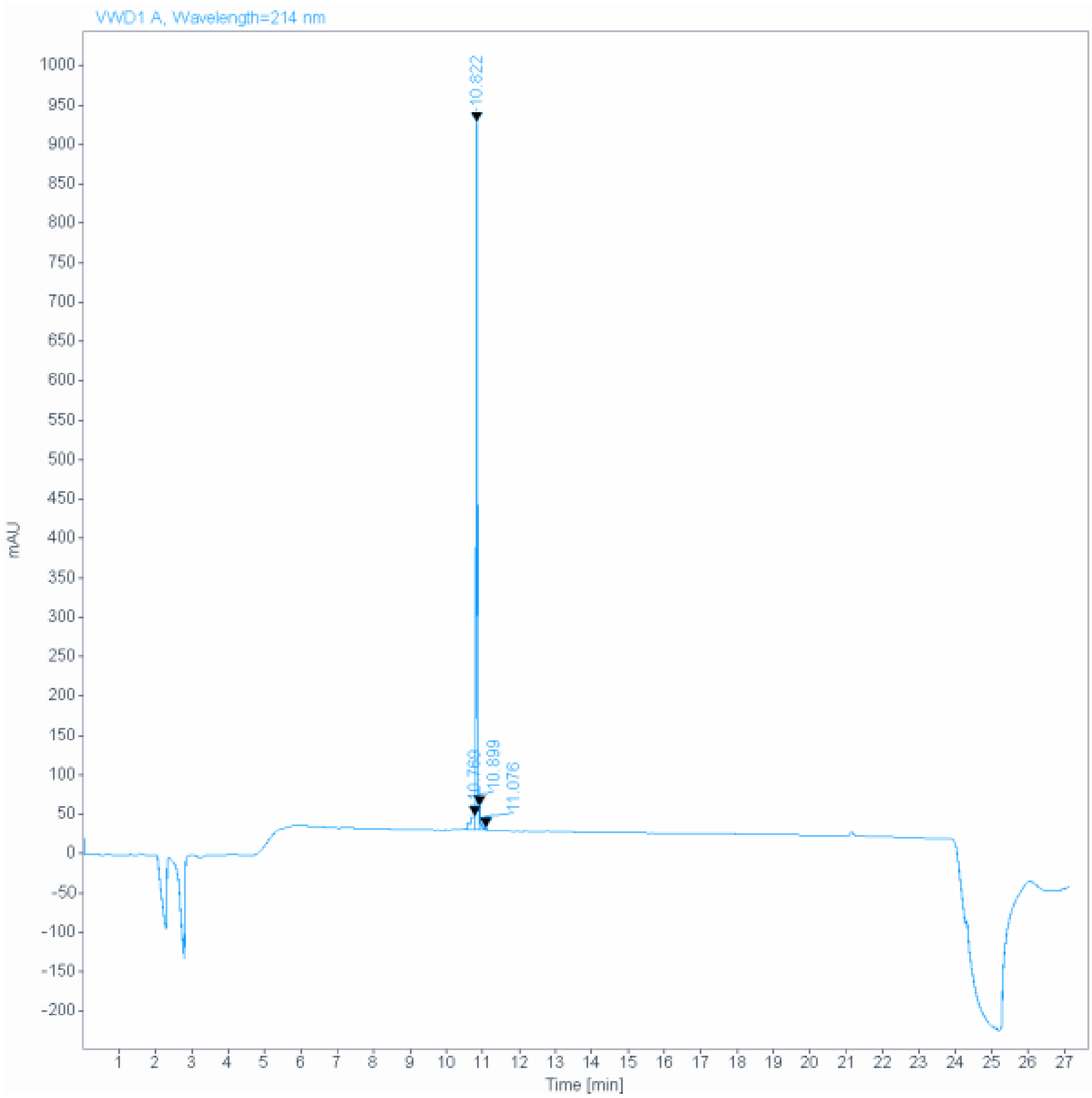

Signal: VWD1 A, Wavelength=214 nm

RT [min]	Type	Width [min]	Area	Height	Area%	Name
10.769	MF	0.1517	158.2035	17.3859	5.6179	
10.822	FM	0.0477	2572.5610	898.4066	91.3524	
10.899	FM	0.0397	71.8466	30.1981	2.5513	
11.076	MM	0.0654	13.4723	3.4346	0.4784	
		Sum	2816.0835			

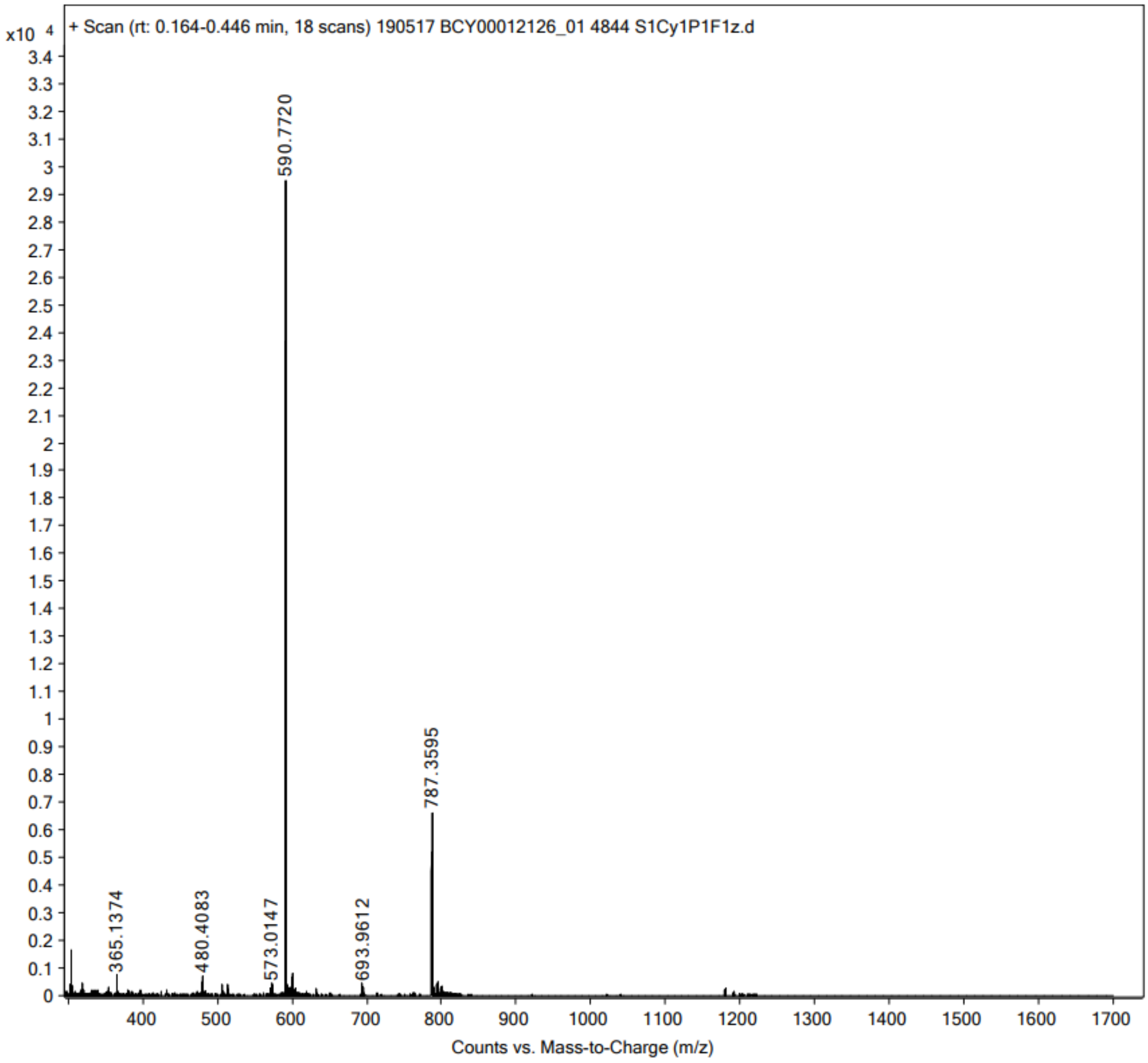

+ Scan (rt: 0.164-0.446 min, 18 scans) 190517 BCY00012126_01 4844 S1Cy1P1F1z.d
x10 4
590.7720
787.3595
365.1374
480.4083
573.0147
693.9612
Counts vs. Mass-to-Charge (m/z)

Peptide 15

Data file: C:\Chem32\1\Data\Peptides\4800-4899_BCY00012128_01 4862 S1Cy1P1F1F2Z 2019-05-20 18-18-03.D

Sample name: _BCY00012128_01 4862 S1Cy1P1F1F2Z

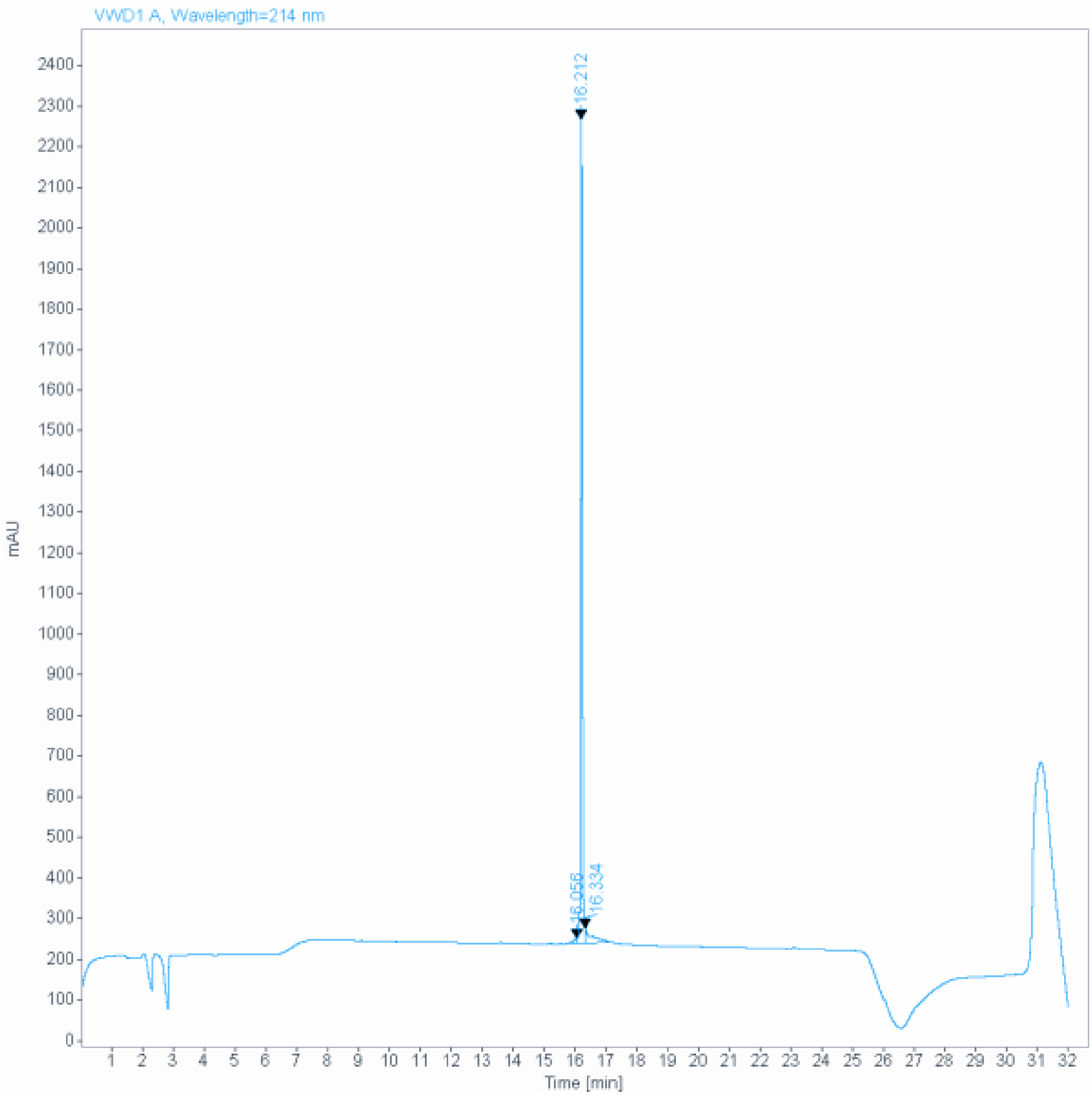

Signal: VWD1 A, Wavelength=214 nm

RT [min]	Type	Width [min]	Area	Height	Area%	Name
16.056	MF	0.1189	76.1543	10.6751	0.9295	
16.212	MF	0.0626	7619.6328	2027.8024	92.9980	
16.334	FM	0.2442	497.5469	33.9556	6.0726	
		Sum	8193.3340			

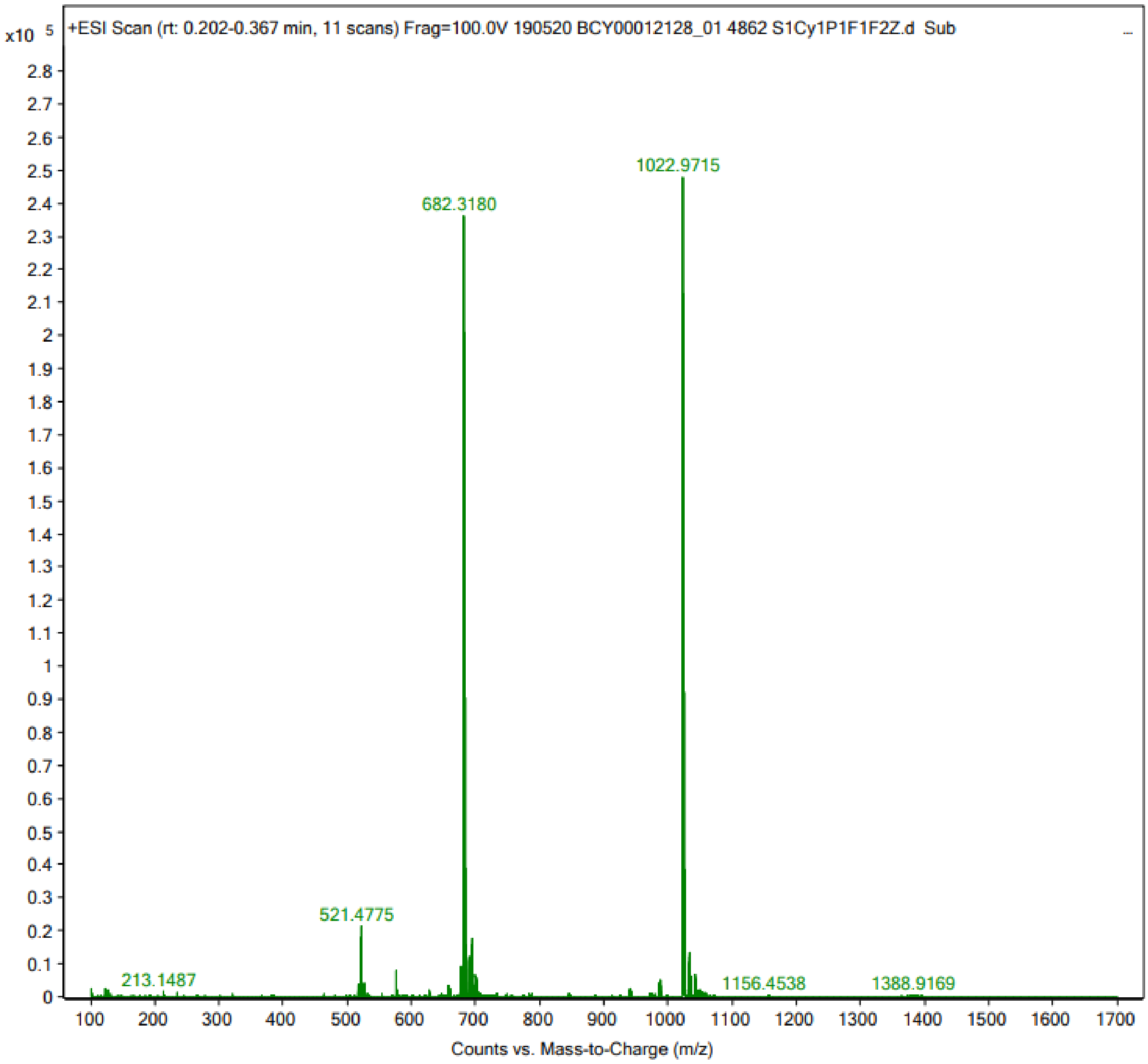

x10 5
+ESI Scan (rt: 0.202-0.367 min, 11 scans) Frag=100.0V 190520 BCY00012128_01 4862 S1Cy1P1F1F2Z.d Sub
1022.9715
682.3180
521.4775
213.1487
1156.4538
1388.9169
Counts vs. Mass-to-Charge (m/z)

Peptide 16

Sample name:	(101-08-00) Ala1 (TATA) QC
Instrument:	1260_1
Injection date:	11/4/2019 2:52:52 PM
Acq. method:	0595B_AB_Poroshell120_15.5min.M

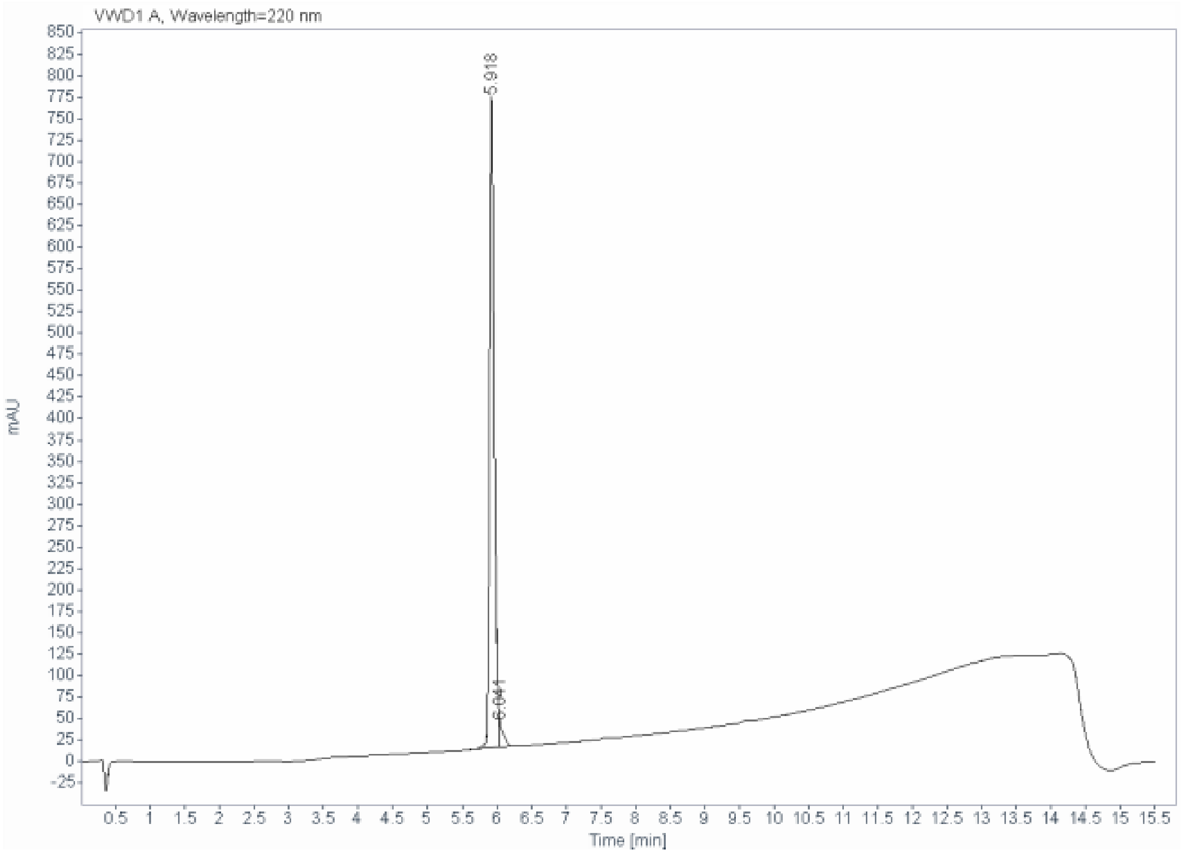

Signal: VWD1 A, Wavelength=220 nm

RT [min]	Type	Width [min]	Area	Height	Area%
5.918	BF	0.0724	3666.1338	757.6926	97.2293
6.041	VB	0.0617	104.4730	28.2120	2.7707
		Sum	3770.6067		

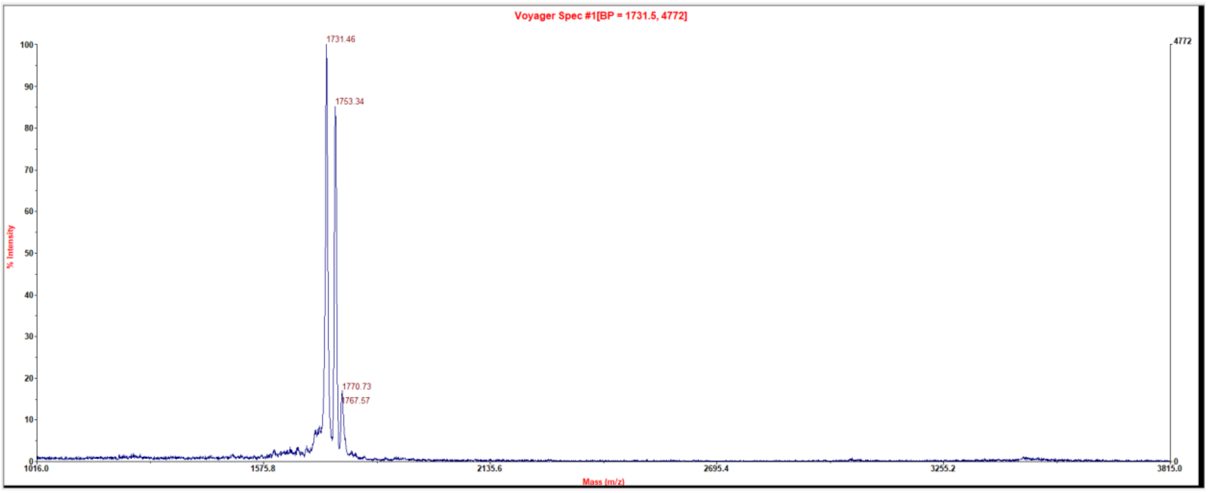

Peptide 17

**Sample name:**	(101-08-00) Ala2 (TATA) QC
**Instrument:**	1260_1
**Injection date:**	11/4/2019 3:09:26 PM
**Acq. method:**	0595B_AB_Poroshell120_15.5min.M

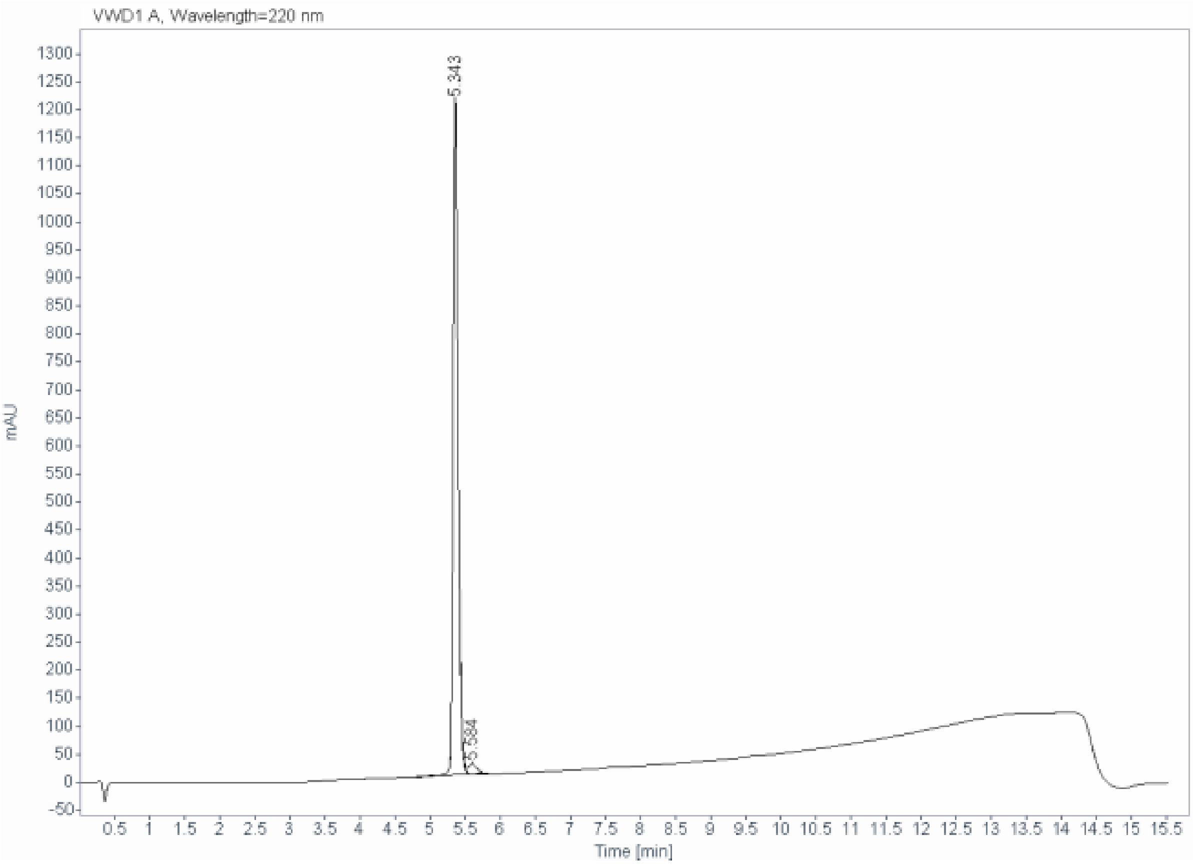

Signal: VWD1 A, Wavelength=220 nm

RT [min]	Type	Width [min]	Area	Height	Area%
5.343	BV R	0.0744	5935.8984	1206.6854	97.1012
5.584	VB E	0.1295	177.2058	18.7091	2.8988
		Sum	6113.1042		

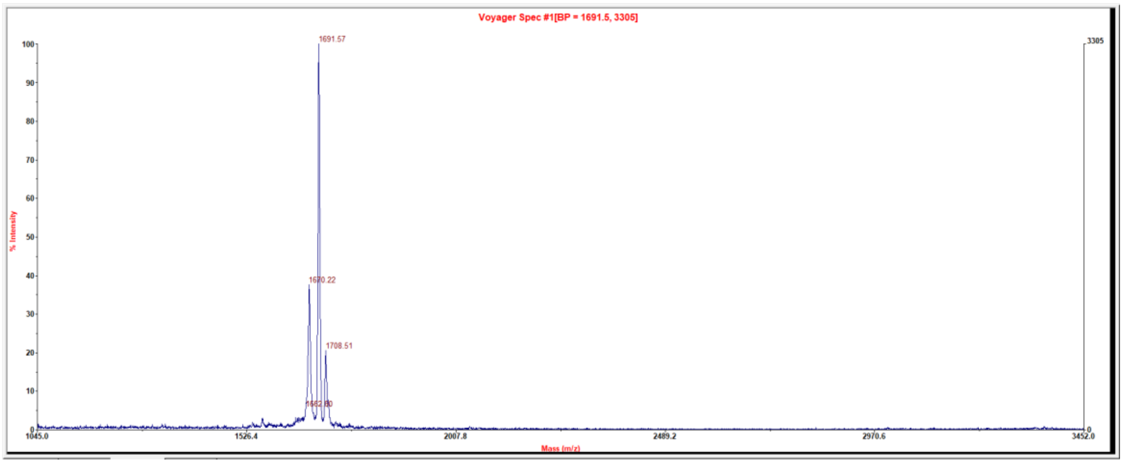

Peptide 18

**Sample name:**	(101-08-00) Ala3 (TATA) QC
**Instrument:**	1260_1
**Injection date:**	11/4/2019 3:25:45 PM
**Acq. method:**	0595B_AB_Poroshell120_15.5min.M

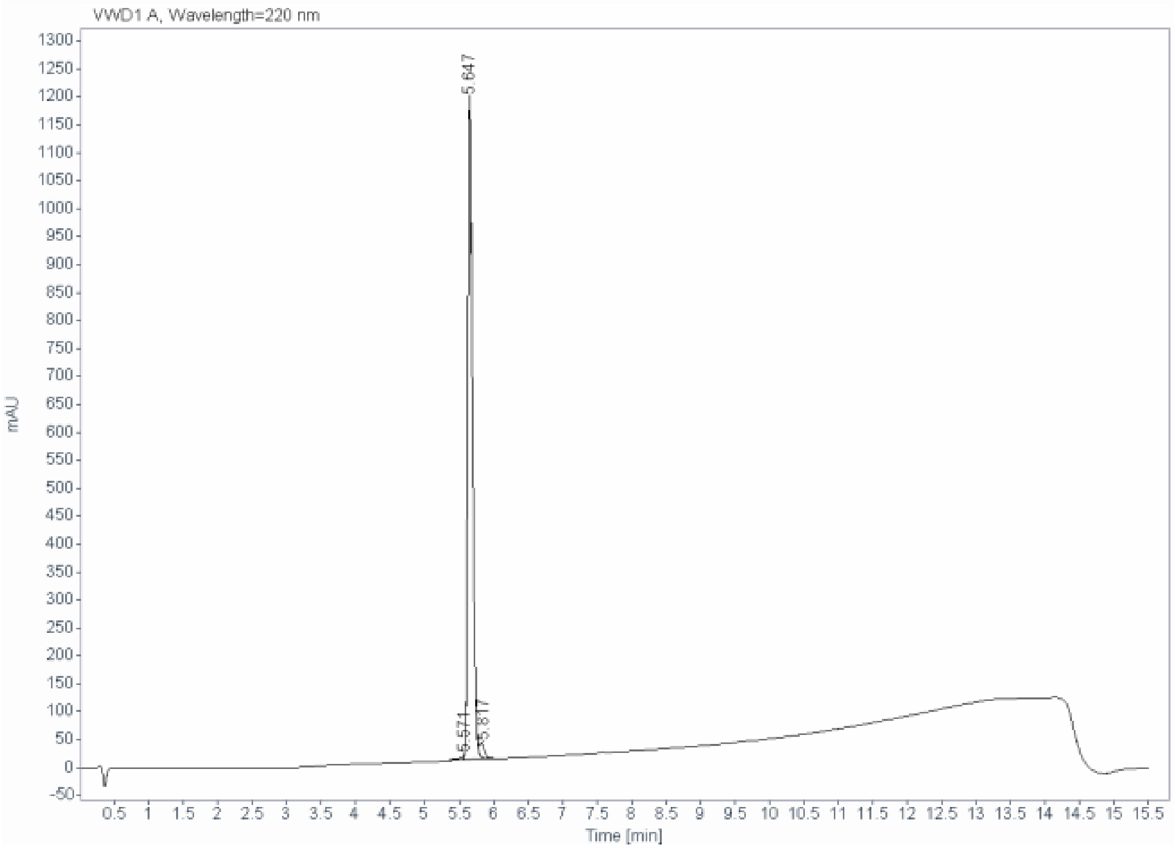

Signal: VWD1 A, Wavelength=220 nm

RT [min]	Type	Width [min]	Area	Height	Area%
5.571	BV	0.1024	52.6781	8.5738	0.9037
5.647	VV R	0.0716	5646.2817	1185.5745	96.8662
5.817	VB E	0.0786	129.9880	24.6271	2.2300
		Sum	5828.9479		

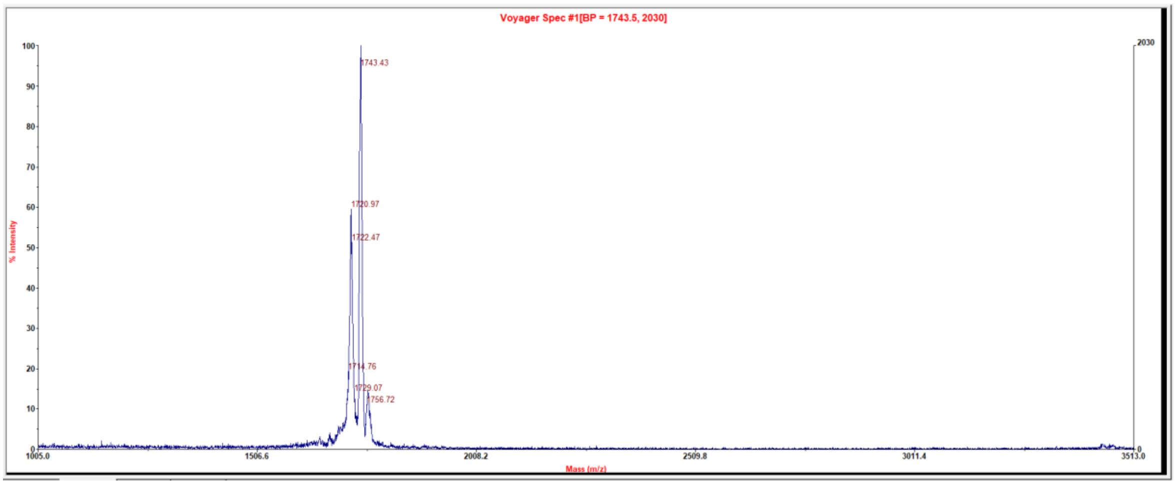

Peptide 19

Sample name: (101-08-00) Ala4 (TATA) QC
Instrument: 1260_1

Injection date: 11/4/2019 3:42:20 PM
Acq. method: 0595B_AB_Poroshell120_15.5min.M

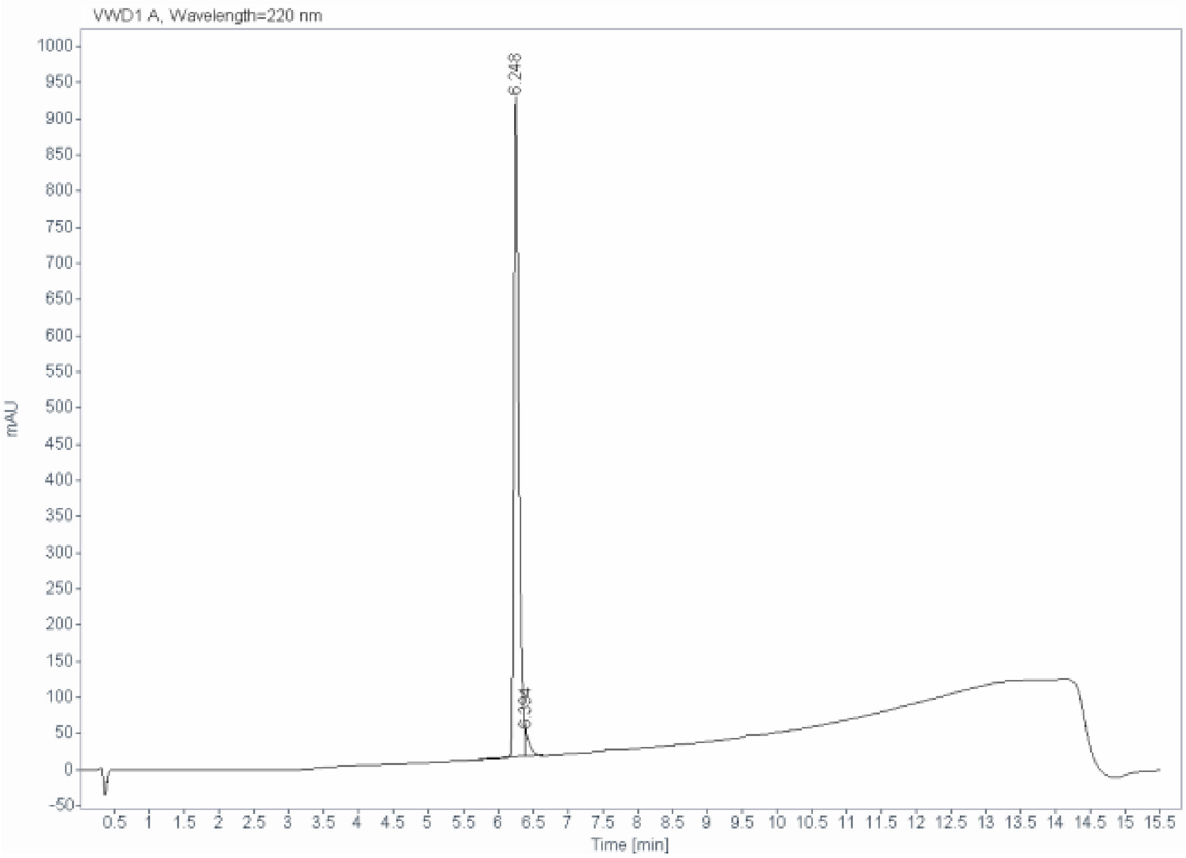

Signal: VWD1 A, Wavelength=220 nm

RT [min]	Type	Width [min]	Area	Height	Area%
6.248	BF	0.0717	4504.1519	910.2987	97.1590
6.394	VB	0.0654	131.7032	33.5718	2.8410
		Sum	4635.8550		

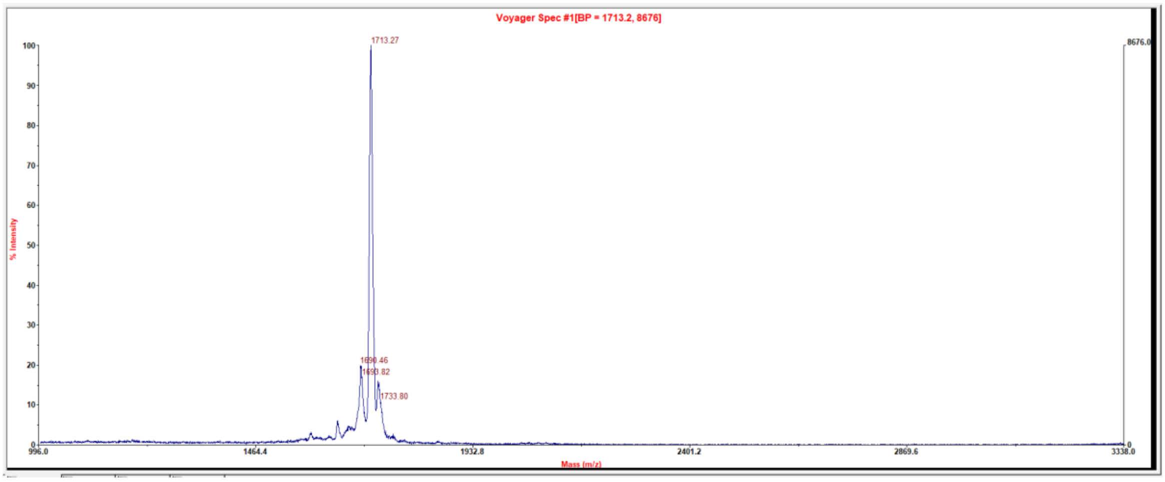

Peptide 20

Sample name: (101-08-00) Ala5 (TATA) QC2
Instrument: 1260_1
Injection date: 11/4/2019 4:14:59 PM
Acq. method: 0595B_AB_Poroshell1 20_15.5min.M

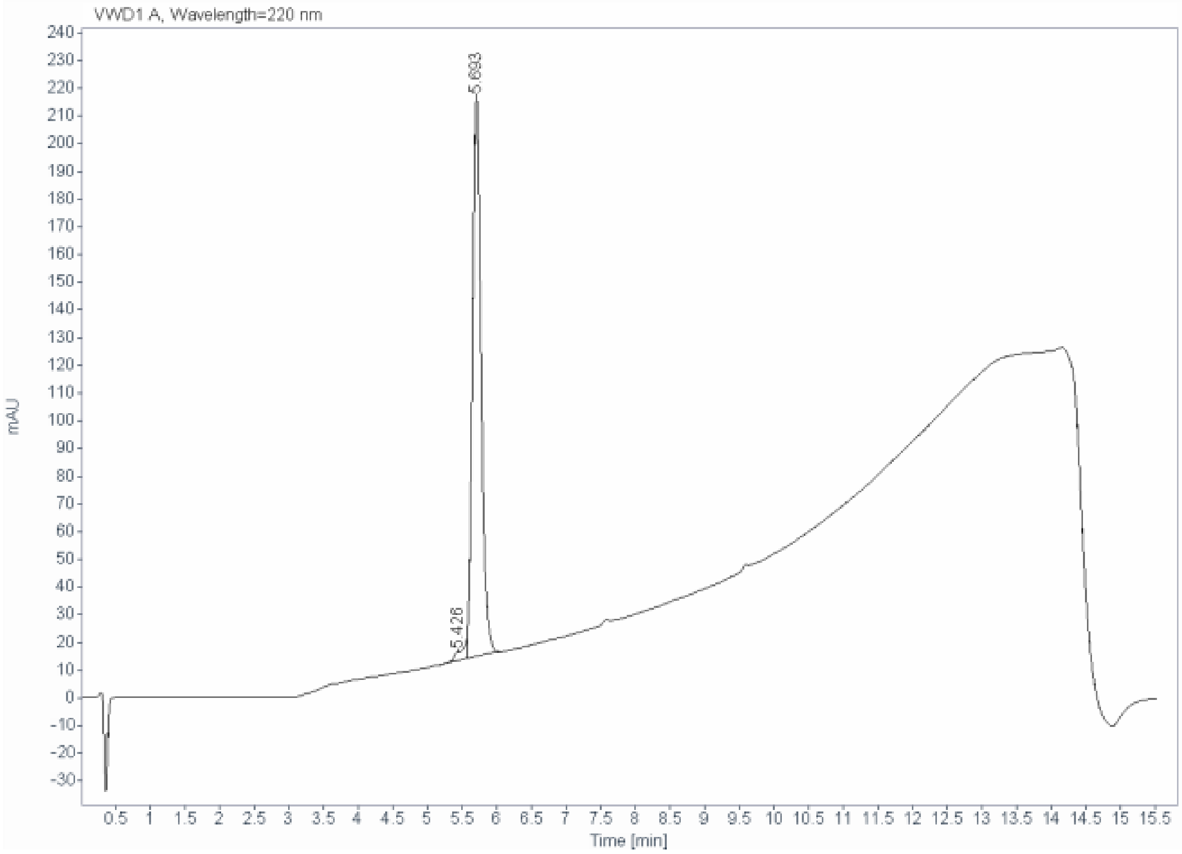

Signal: VWD1 A, Wavelength=220 nm

RT [min]	Type	Width [min]	Area	Height	Area%
5.426	BV	0.1952	50.1846	3.2858	2.7684
5.693	VB	0.1358	1762.5721	201.9507	97.2316
		Sum	1812.7568		

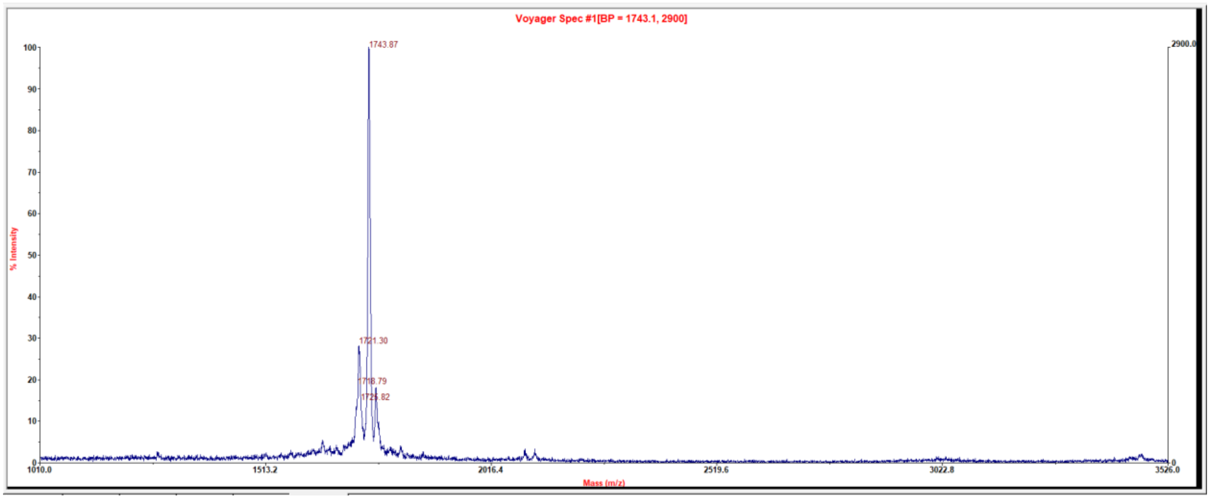

Peptide 21

Sample name:	(101-08-00) Ala6 (TATA) QC2
Instrument:	1260_1
Injection date:	11/4/2019 4:55:17 PM
Acq. method:	0595B_AB_Poroshell120_15.5min.M

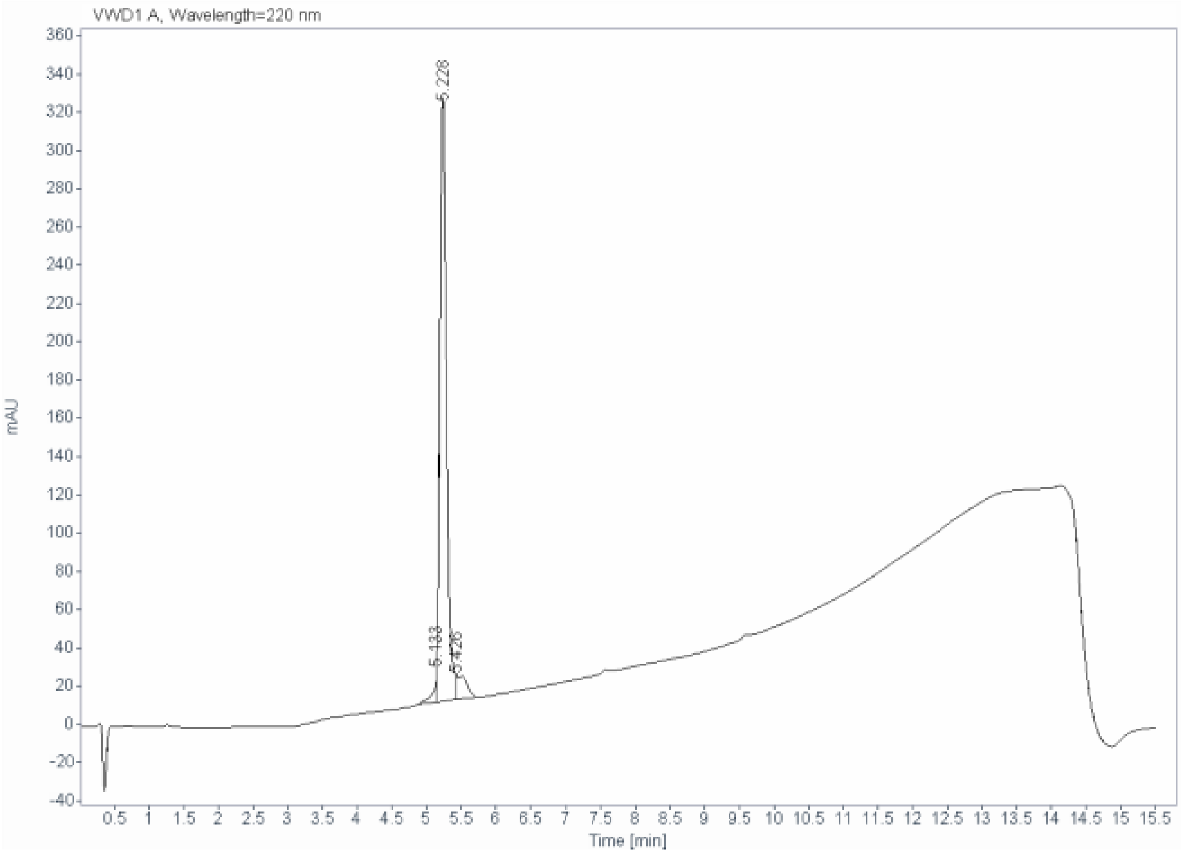

Signal: VWD1 A, Wavelength=220 nm

RT [min]	Type	Width [min]	Area	Height	Area%
5.133	BV	0.0480	52.2967	18.1773	2.3051
5.228	VF	0.1159	2094.3215	291.1203	92.3112
5.426	VB	0.1706	122.1438	11.9302	5.3837
		Sum	2268.7620		

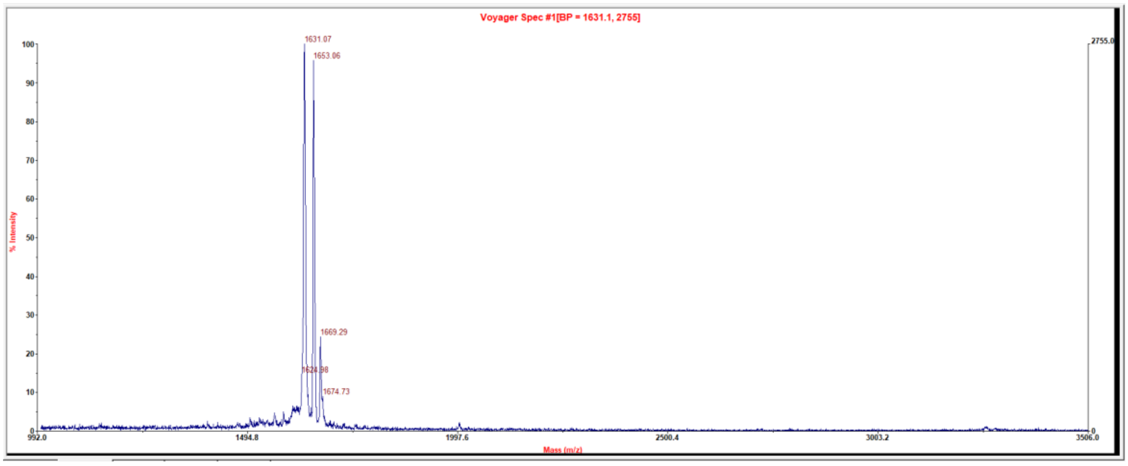

Peptide 22

**Sample name:**	(101-08-00) Ala7 (TATA) QC2
**Instrument:**	1260_1
**Injection date:**	11/4/2019 5:11:36 PM
**Acq. method:**	0595B_AB_Poroshell120_15.5min.M

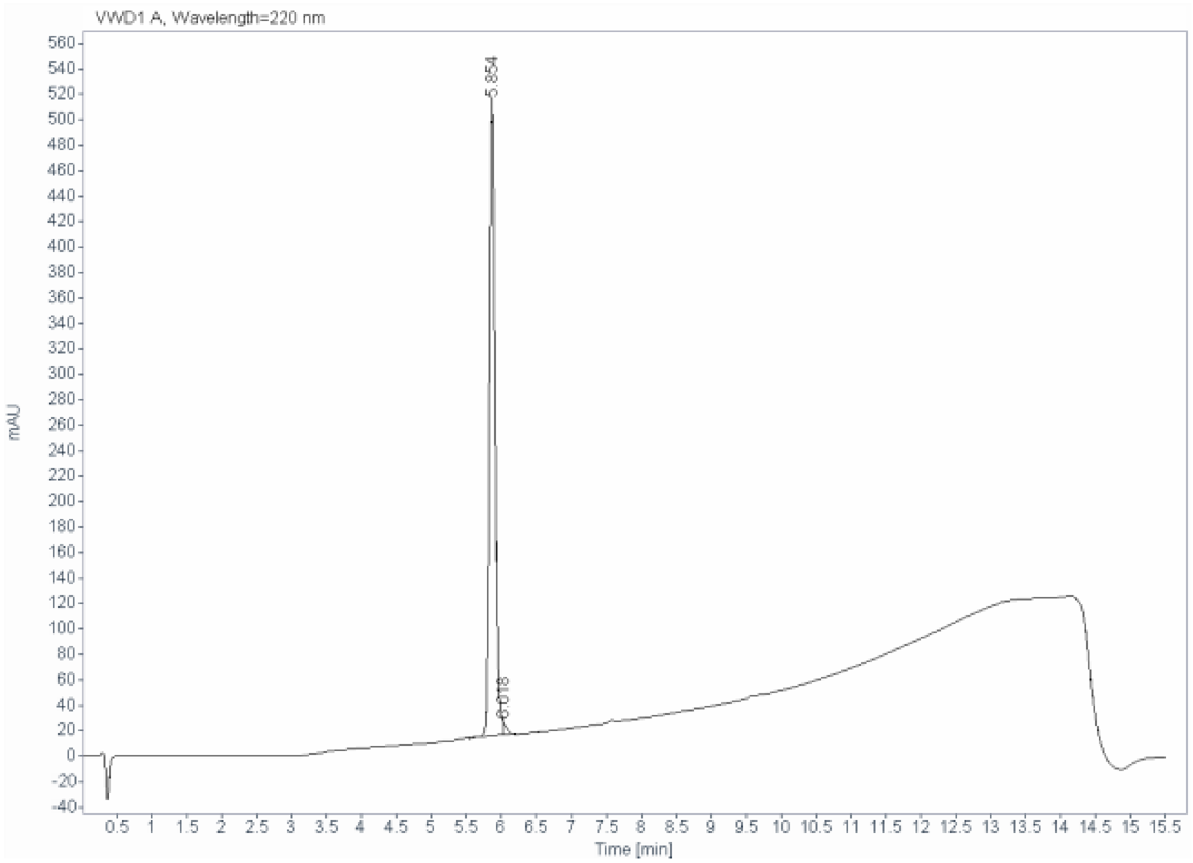

Signal: VWD1 A, Wavelength=220 nm

RT [min]	Type	Width [min]	Area	Height	Area%
5.854	BF	0.0871	2826.5938	498.8858	98.7241
6.018	VB	0.0584	36.5300	10.4305	1.2759
		Sum	2863.1238		

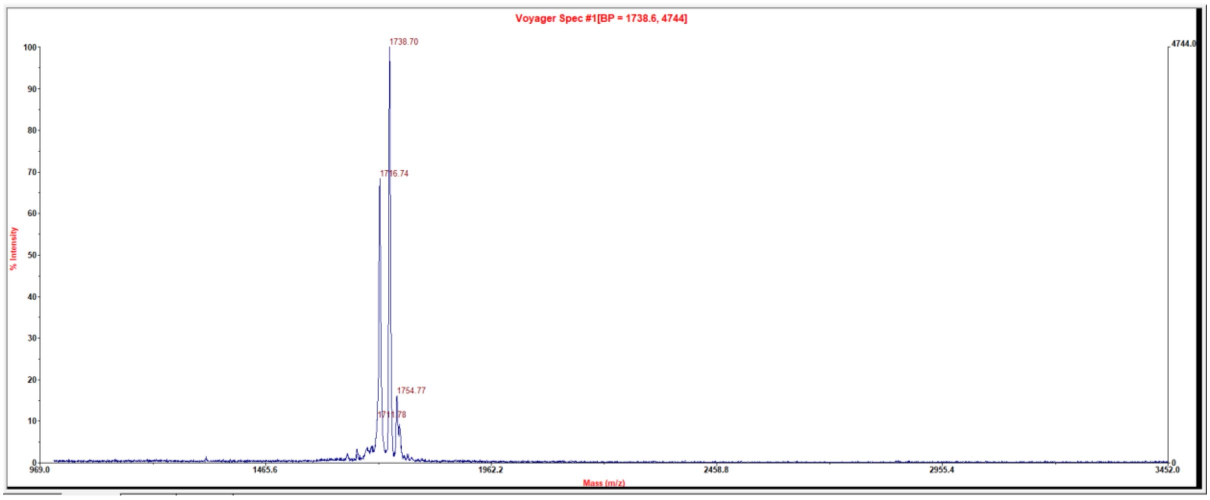

Peptide 23

**Sample name:**	(101-08-00) Ala8 (TATA) QC
**Instrument:**	1260_2
**Injection date:**	11/4/2019 5:15:56 PM
**Acq. method:**	0595B_AB_Poroshell120_15.5min.M

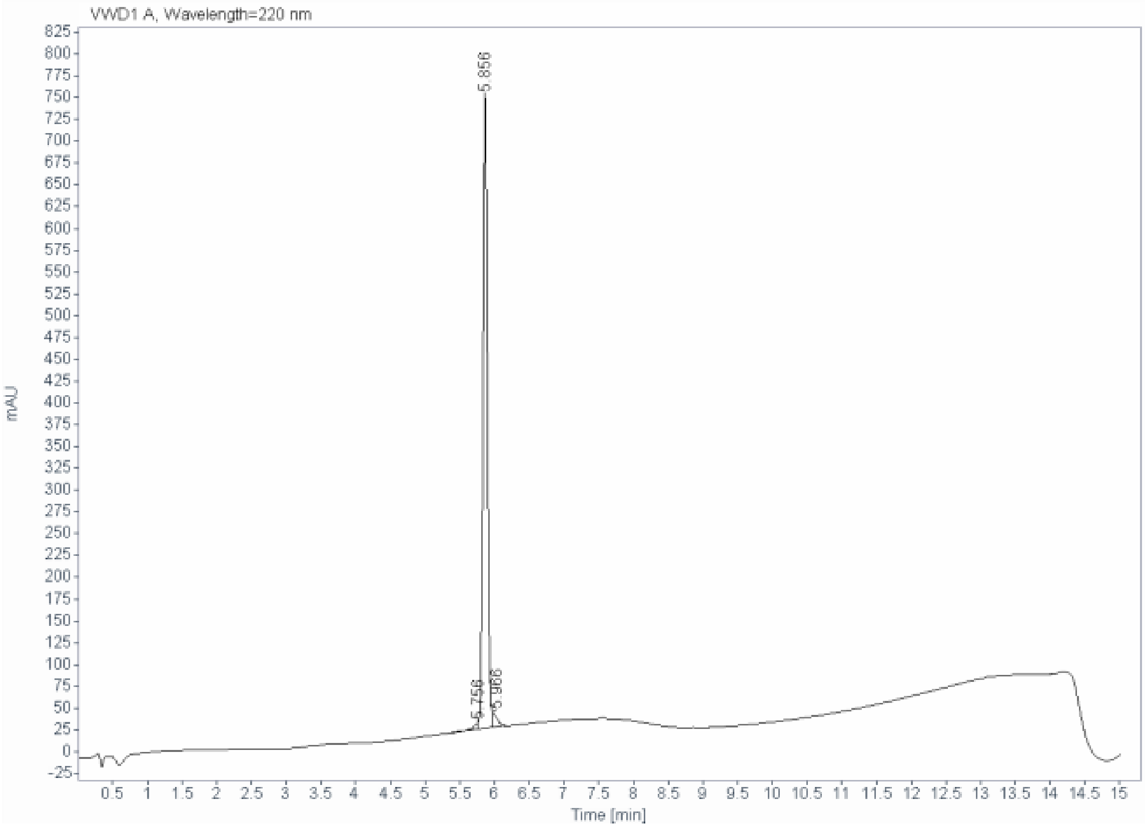

Signal: VWD1 A, Wavelength=220 nm

RT [min]	Type	Width [min]	Area	Height	Area%
5.756	BV	0.0858	39.7773	7.7236	1.2113
5.856	VF	0.0678	3165.6328	727.2114	96.3973
5.966	VB	0.0731	78.5320	17.9154	2.3914
		Sum	3283.9422		

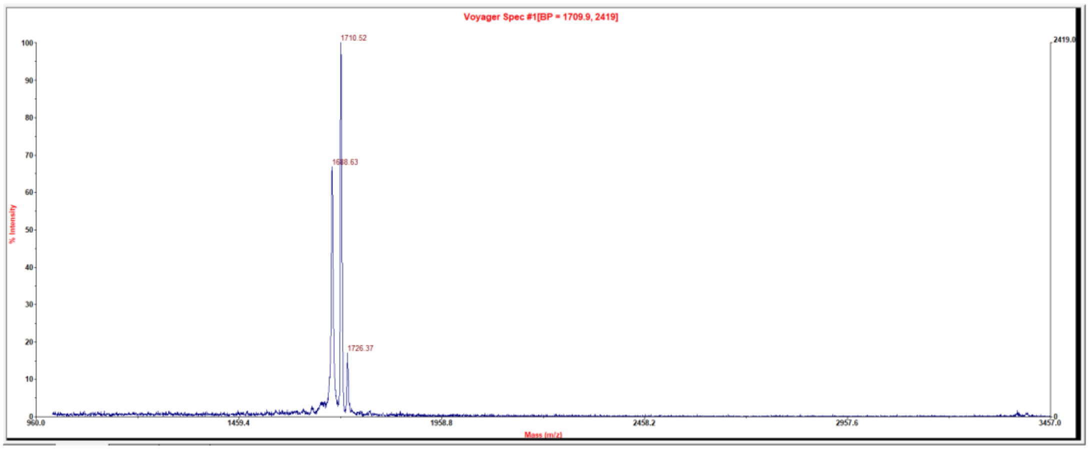

Peptide 24

Sample name:	(101-08-00) Ala9 (TATA) QC
Instrument:	1260_1
Injection date:	11/4/2019 5:29:28 PM
Acq. method:	0595B_AB_Poroshell120_15.5min.M

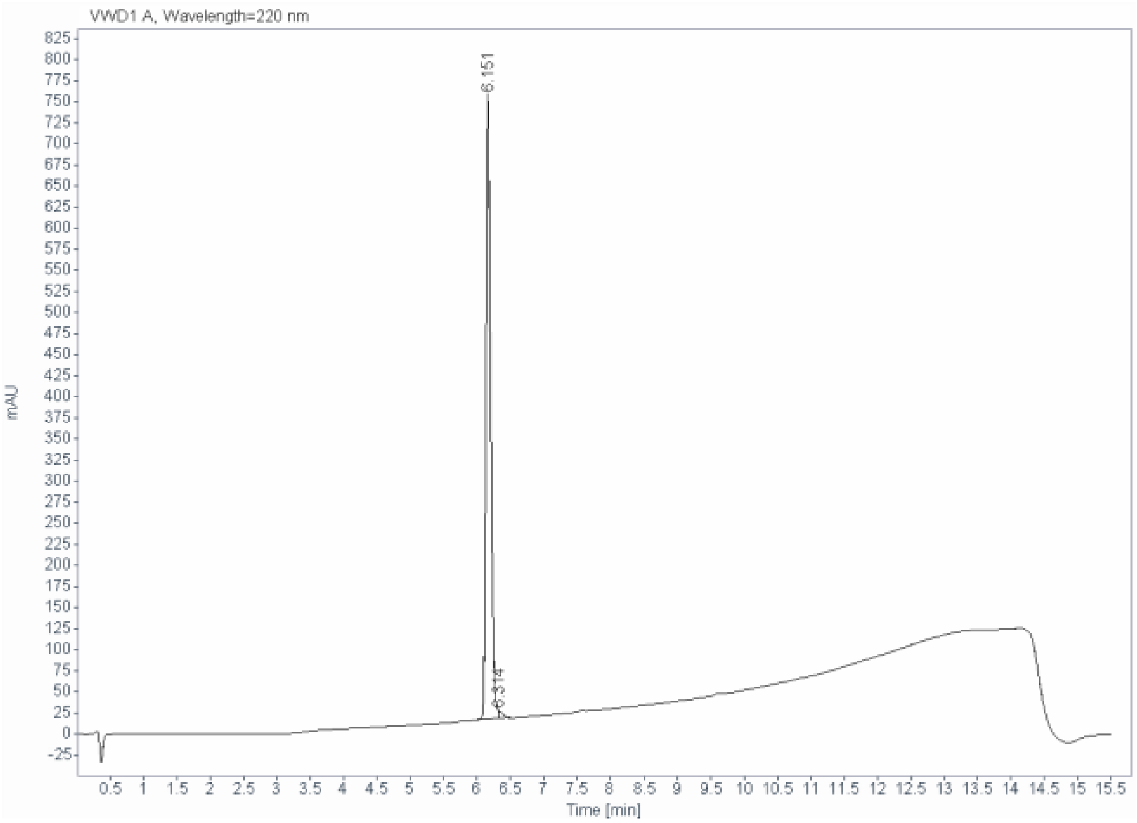

Signal: VWD1 A, Wavelength=220 nm

RT [min]	Type	Width [min]	Area	Height	Area%
6.151	VF R	0.0730	3612.3682	740.0170	98.9778
6.314	VB	0.0715	37.3083	8.6952	1.0222
		Sum	3649.6765		

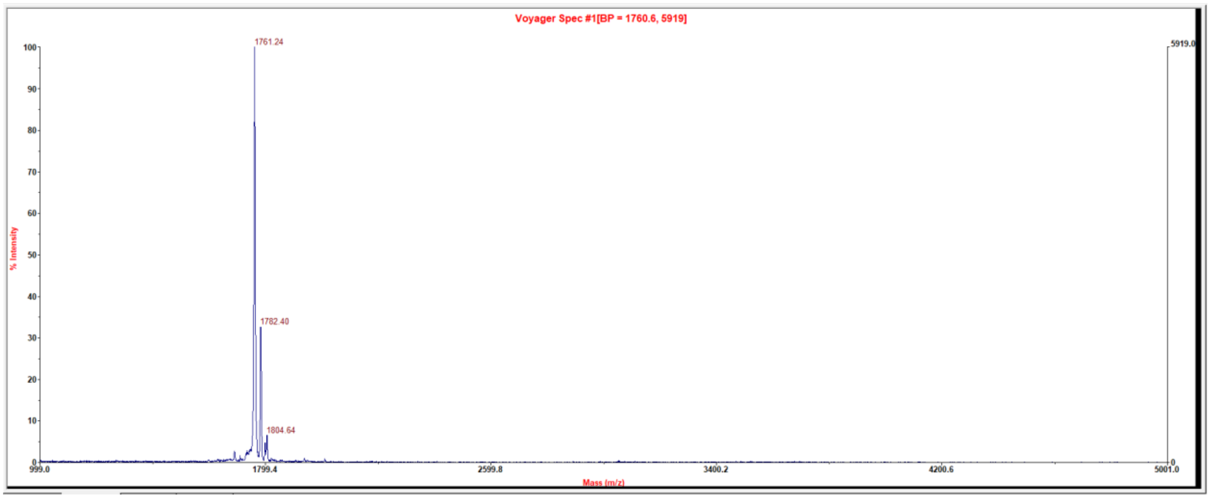

Peptide 25

**Sample name:**	(101-08-00)HArg4 (TATA) QC
**Instrument:**	1260_1
**Injection date:**	10/10/2019 12:34:59 PM
**Acq. method:**	0595B_AB_Poroshell1 20_15.5min.M

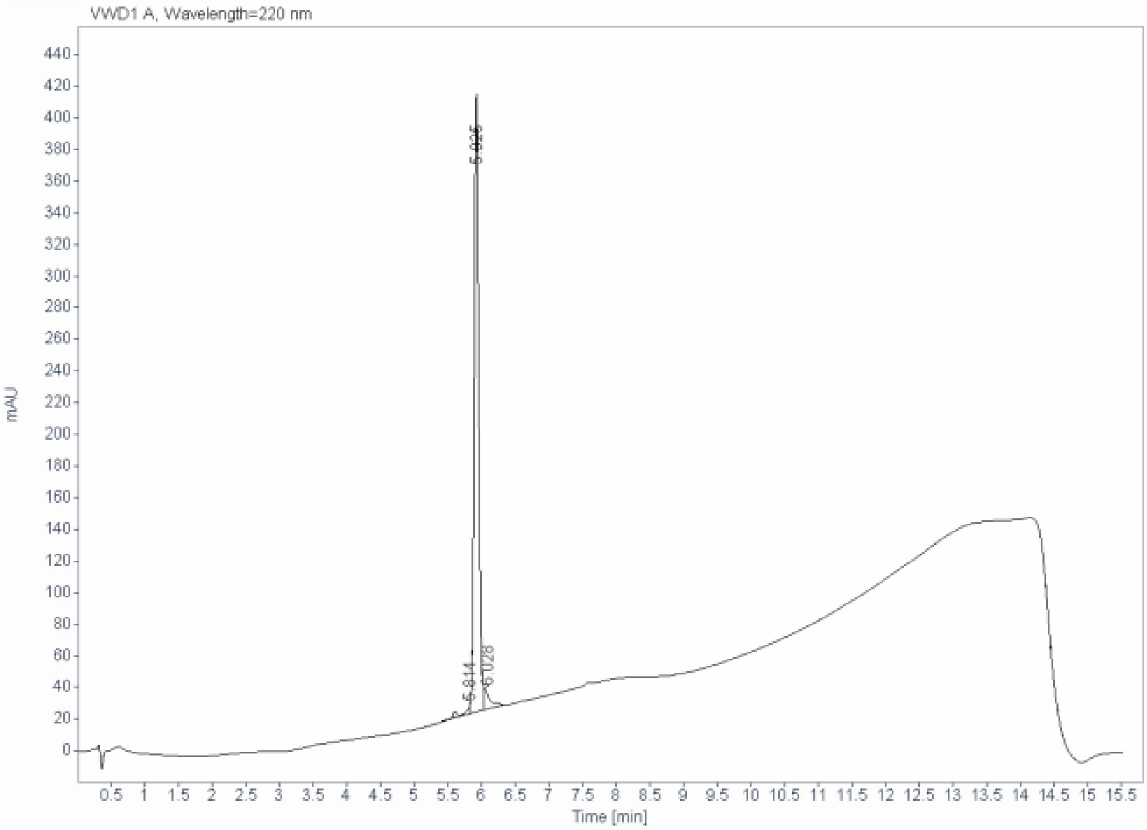

Signal: VWD1 A, Wavelength=220 nm

RT [min]	Type	Width [min]	Area	Height	Area%
5.814	BV	0.0328	44.0170	22.3527	2.2270
5.925	VV	0.1205	1848.8356	255.6557	93.5394
6.028	VB	0.0746	83.6783	18.6860	4.2336
		Sum	1976.5309		

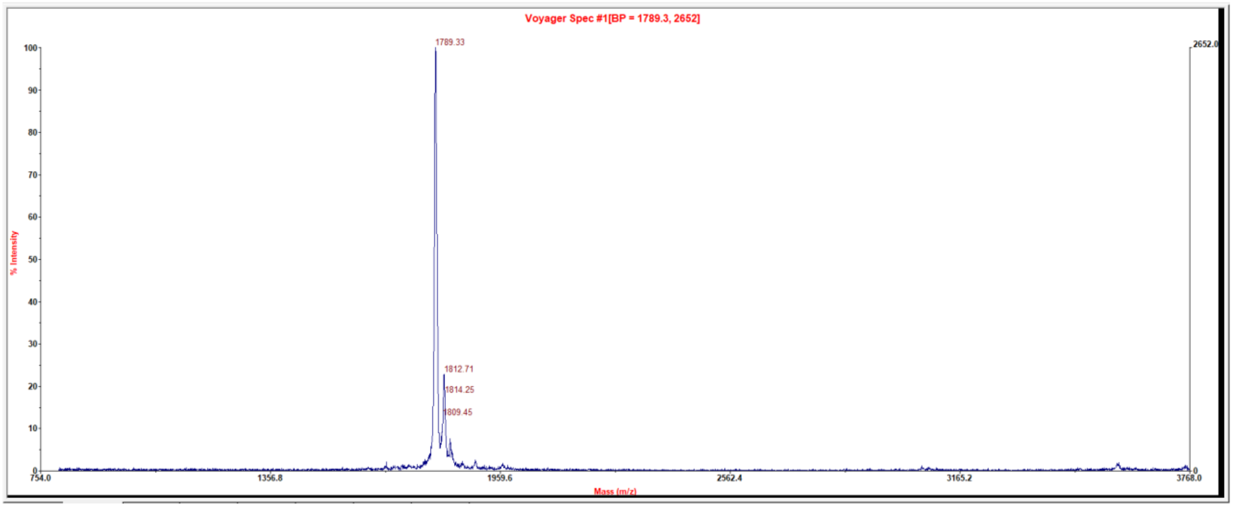

Peptide 26

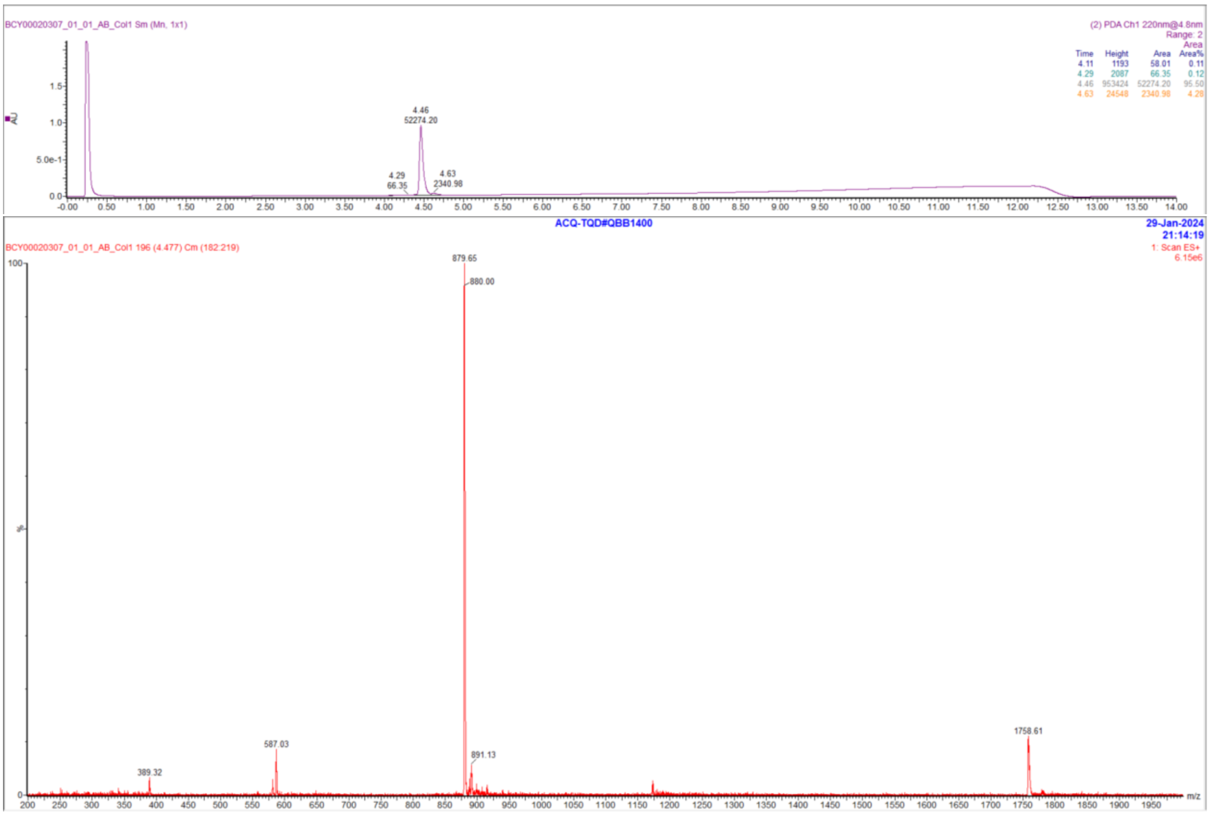

Peptide 27

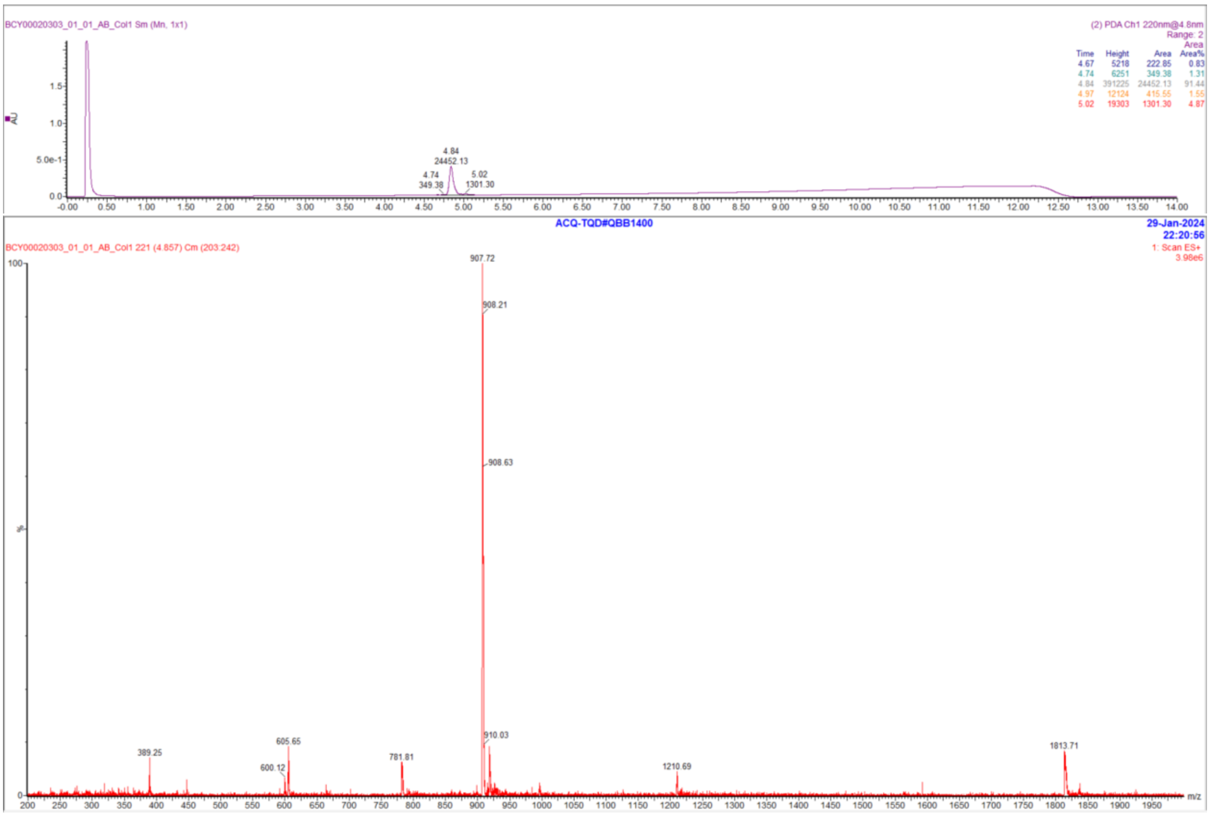

Peptide 28

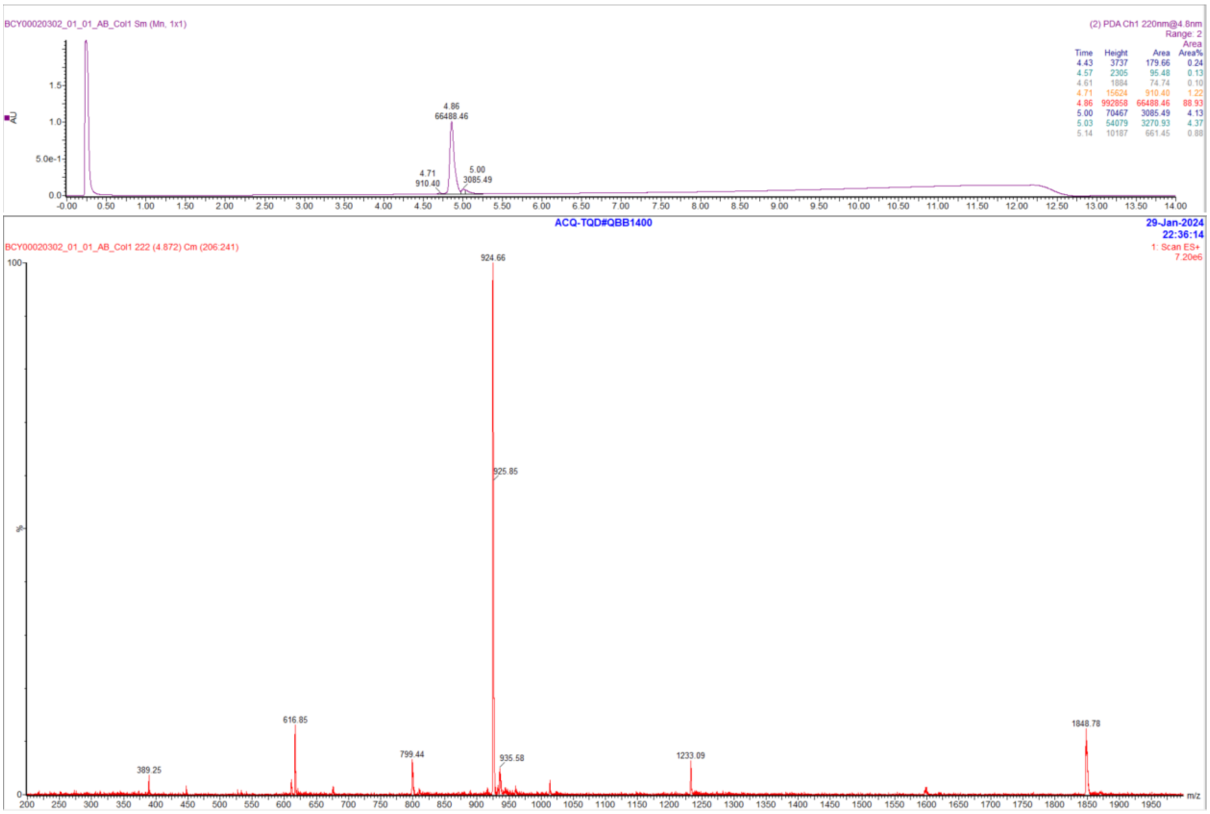

Peptide 29

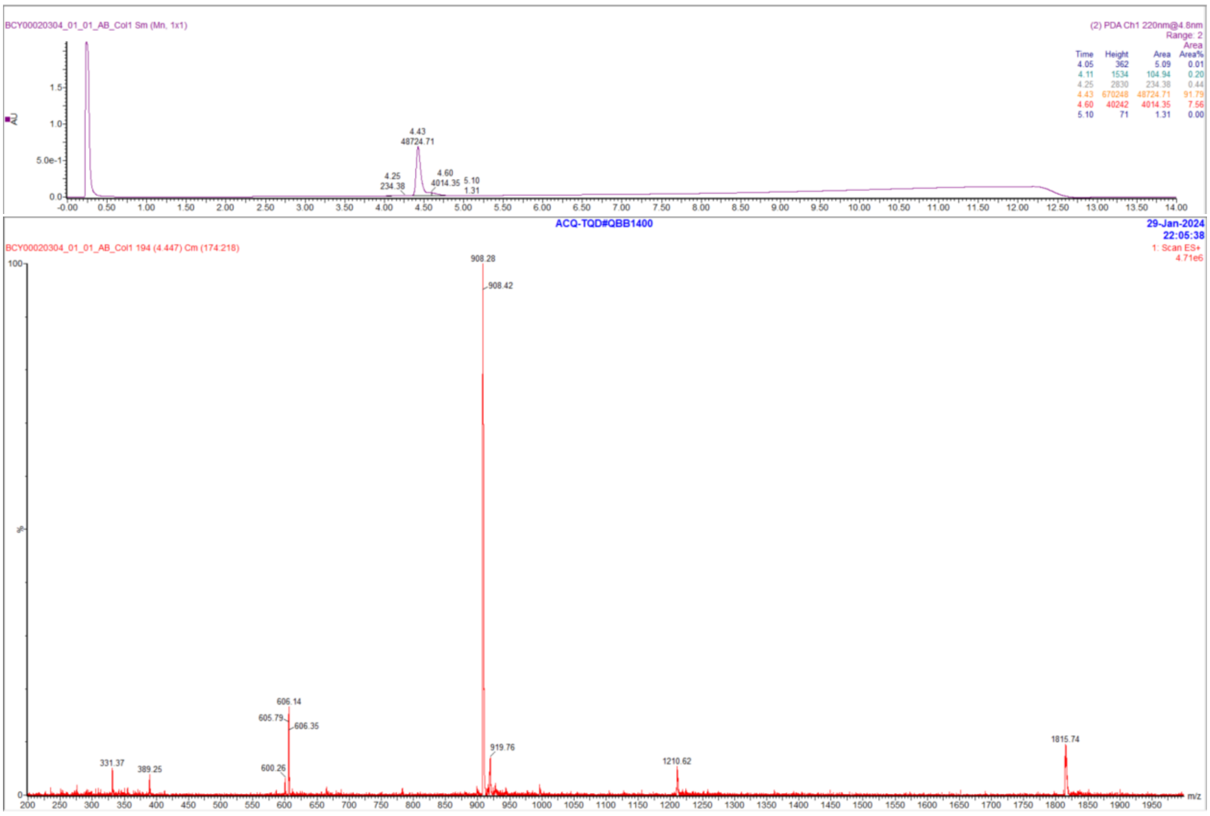

Peptide 30

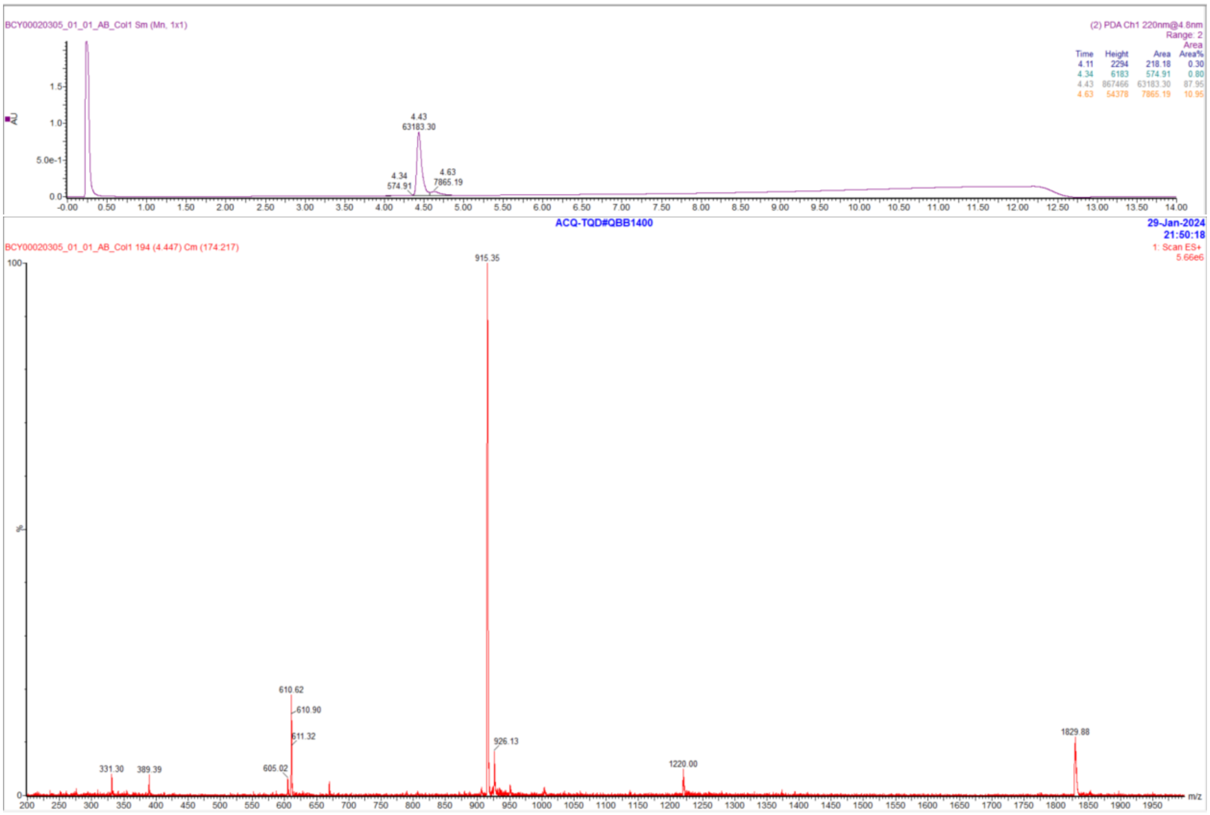

Peptide 31

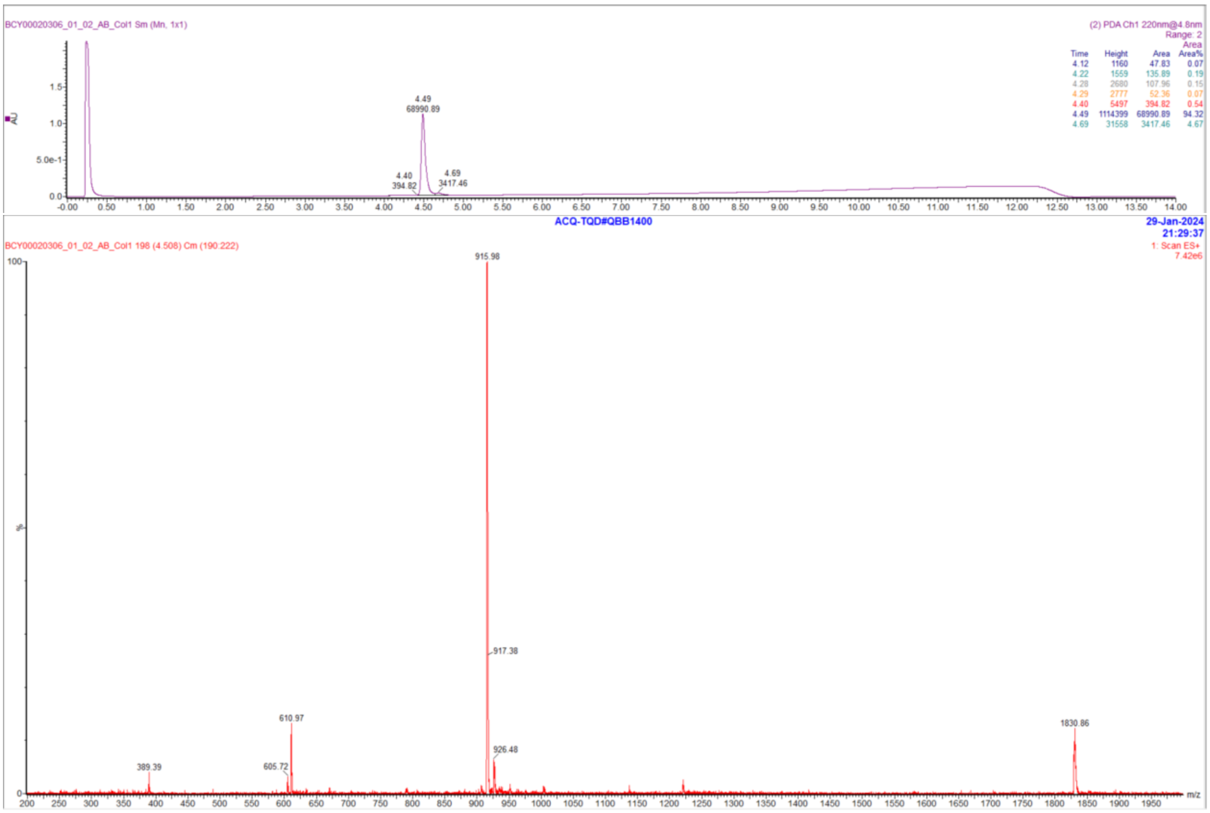

Peptide 32

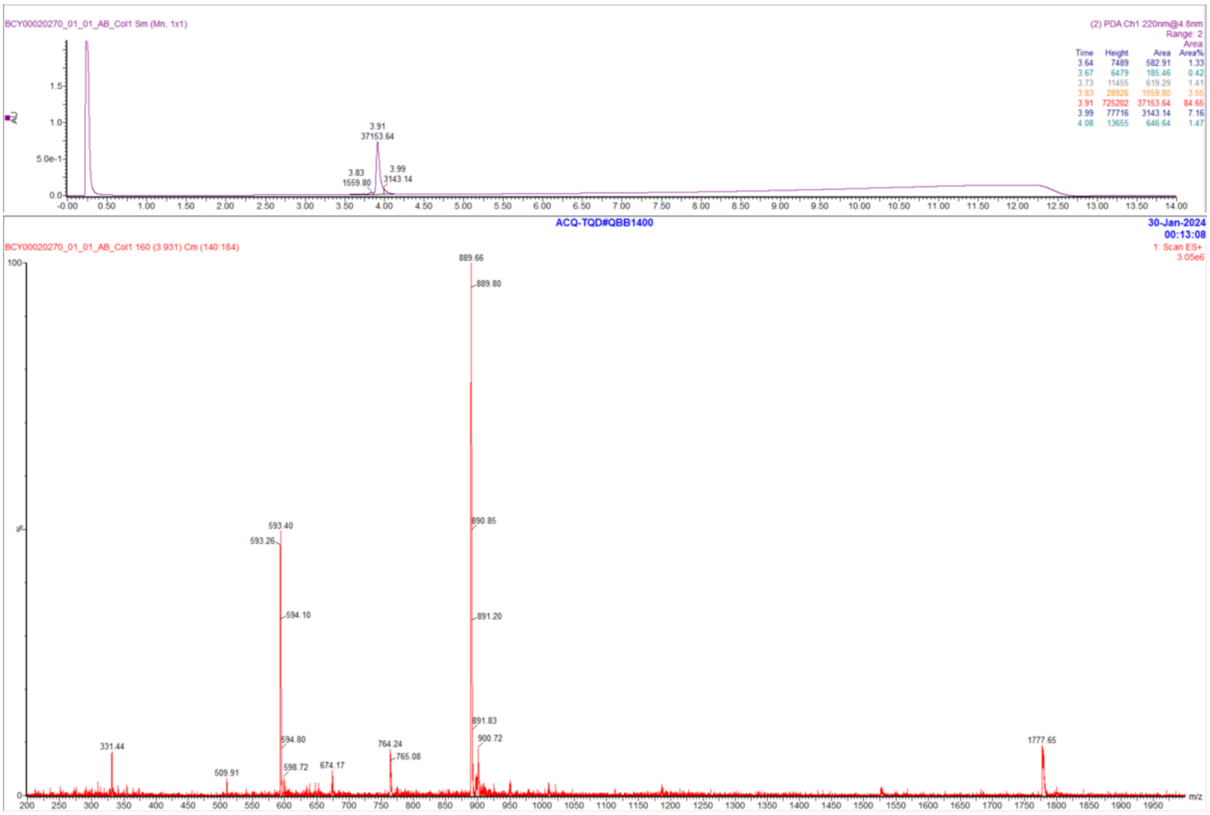

Time	Height	Area	Area%
3.64	7489	582.91	1.33
3.67	6479	185.46	0.42
3.73	11455	619.29	1.41
3.83	28926	1559.80	3.55
3.91	725202	37153.64	84.65
3.99	77716	3143.14	7.16
4.08	13655	646.64	1.47

Peptide 33

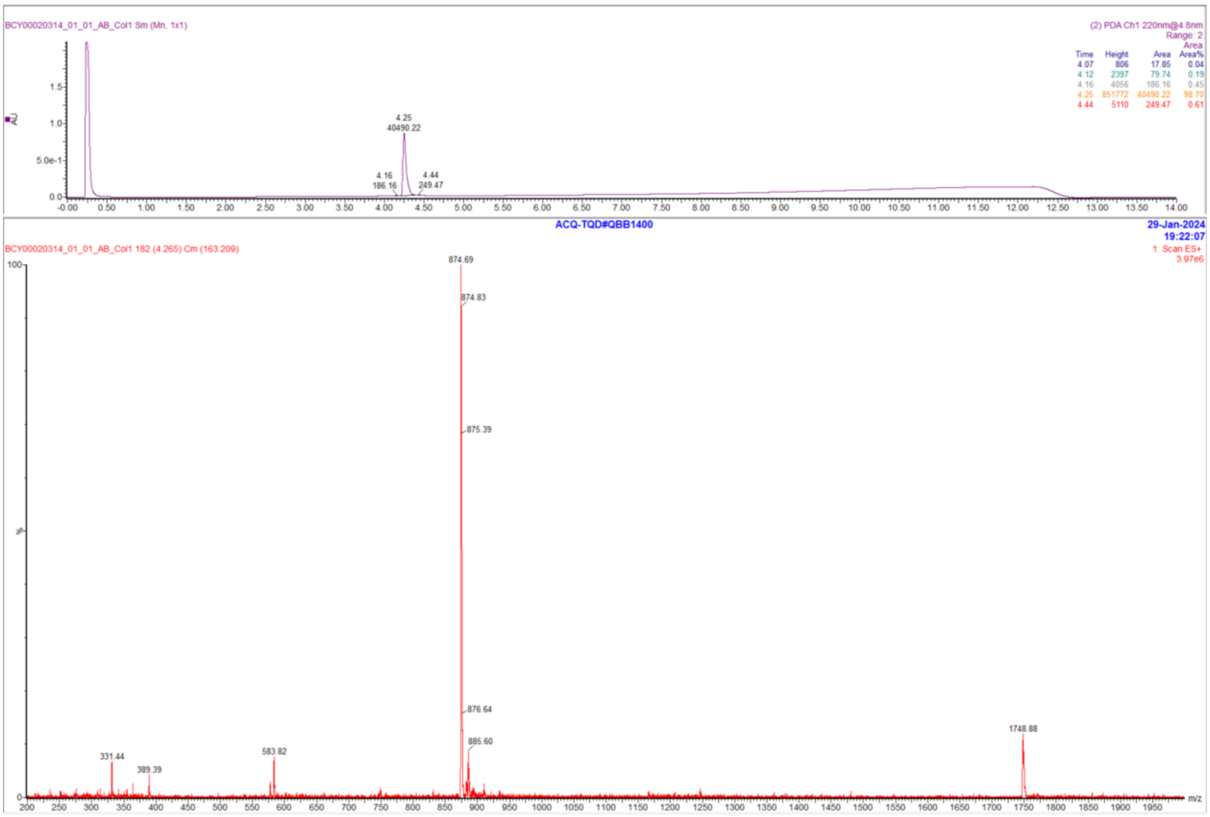

Time	Height	Area	Area%
4.07	806	17.85	0.04
4.12	2397	79.74	0.19
4.16	4056	186.16	0.45
4.25	851772	40490.22	98.70
4.44	5110	249.47	0.61

Peptide 34

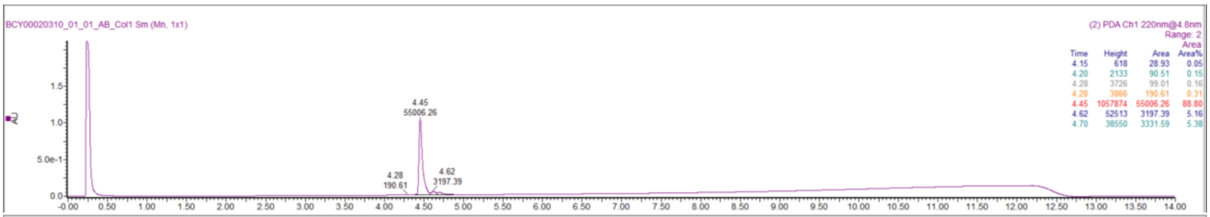

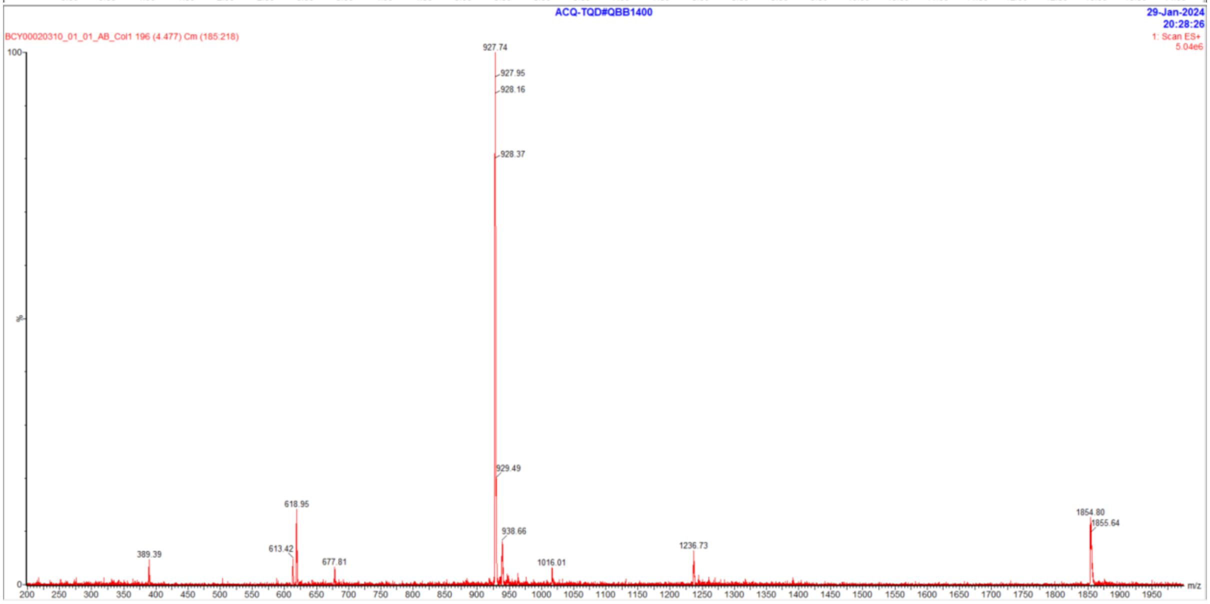

Peptide 35

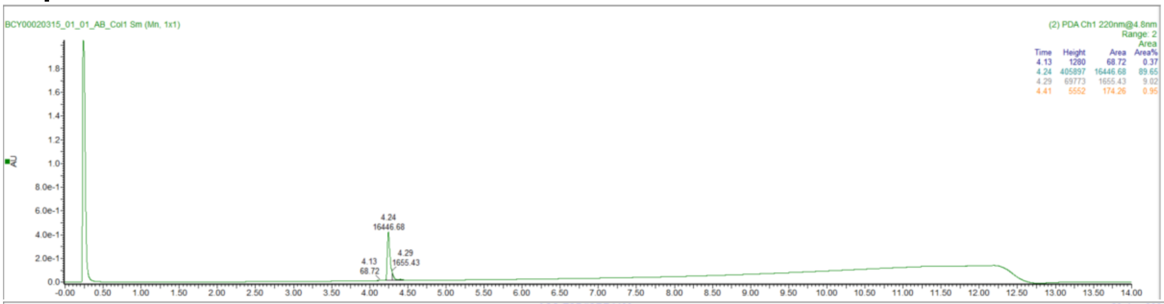

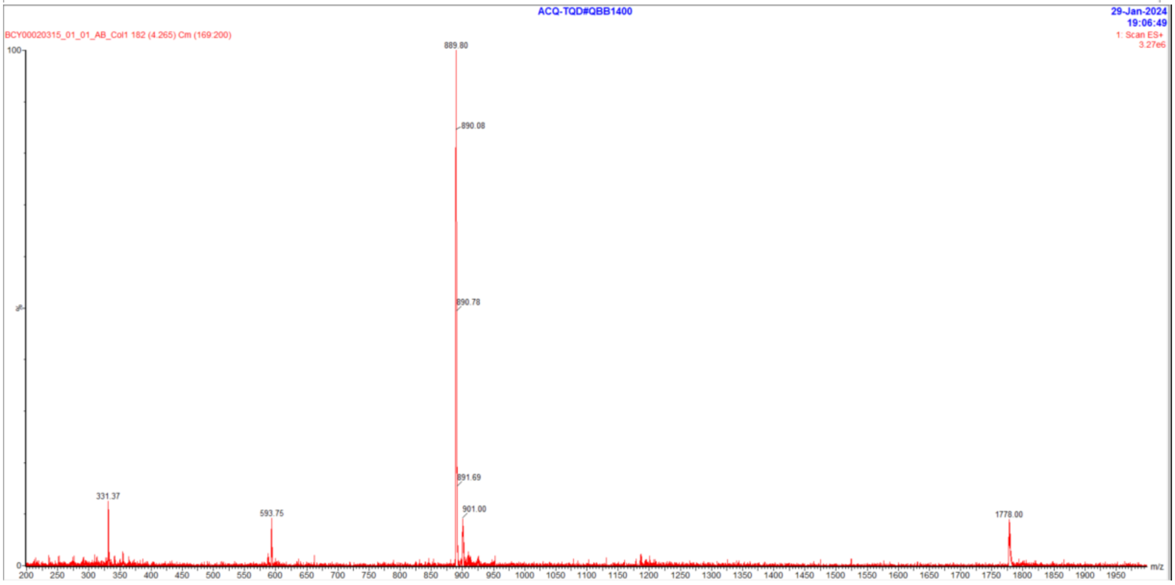

Peptide 36

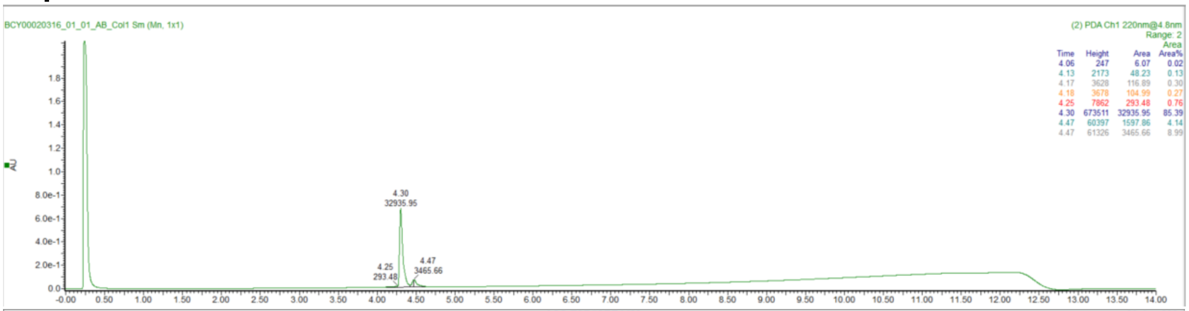

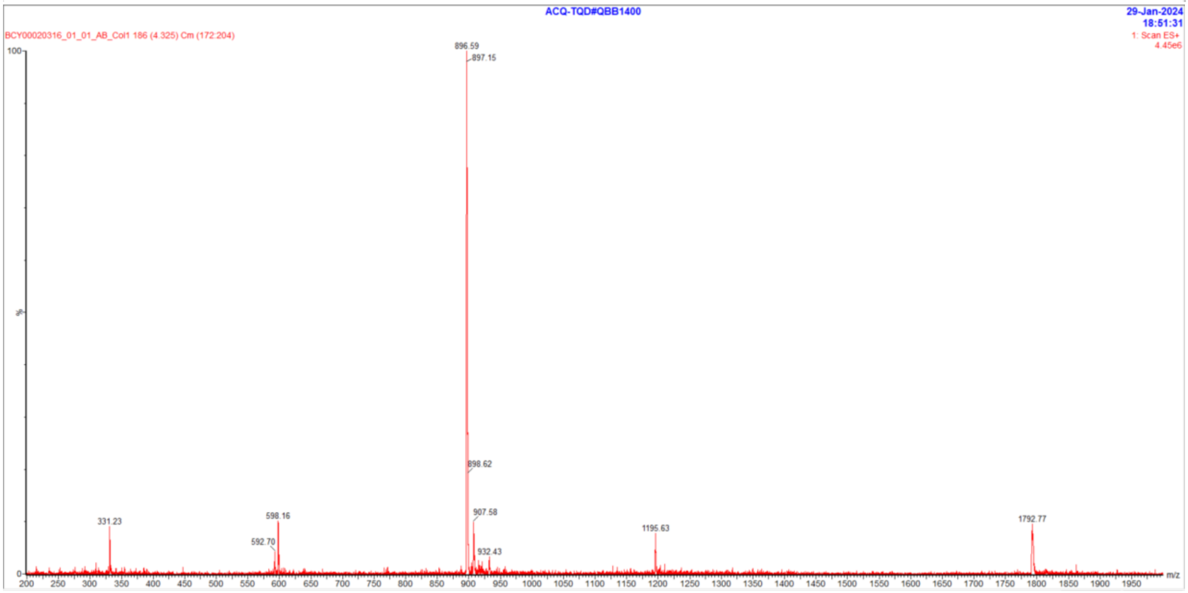

Peptide 37

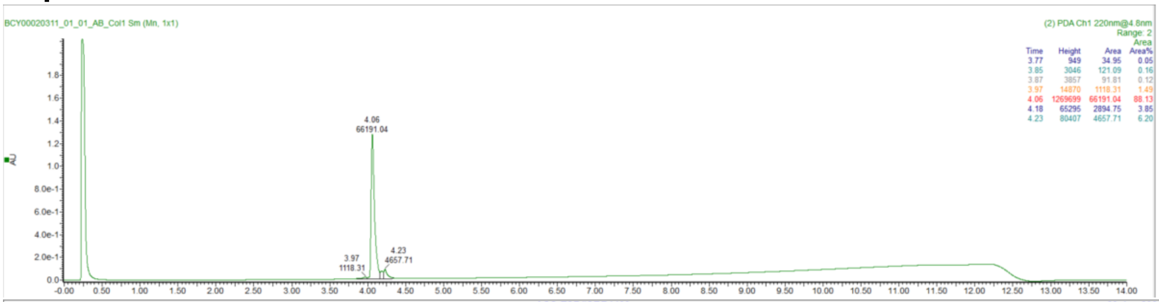

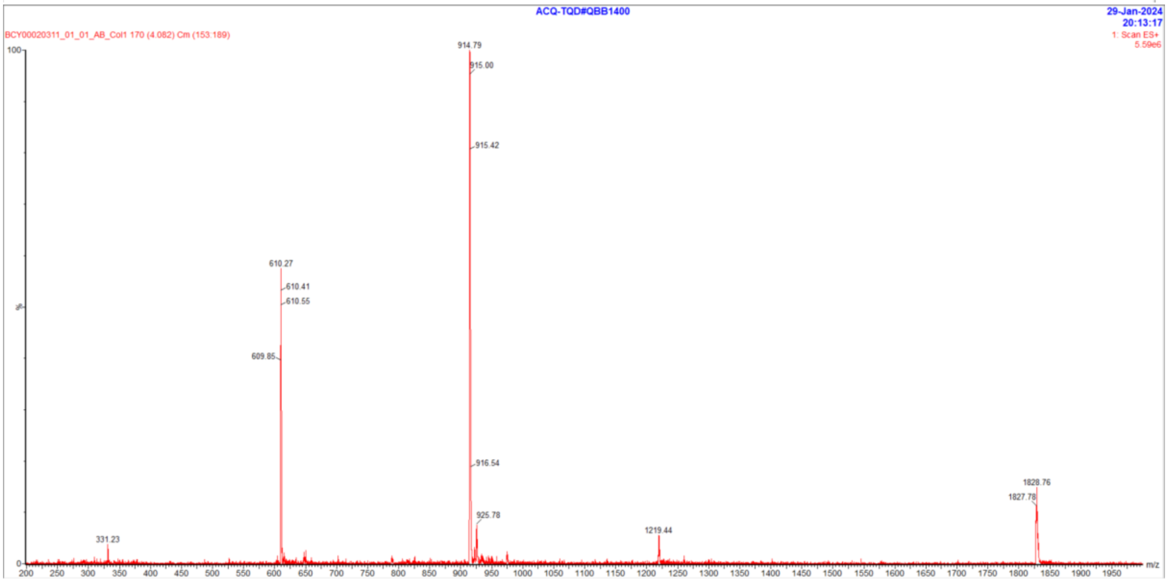

Peptide 38

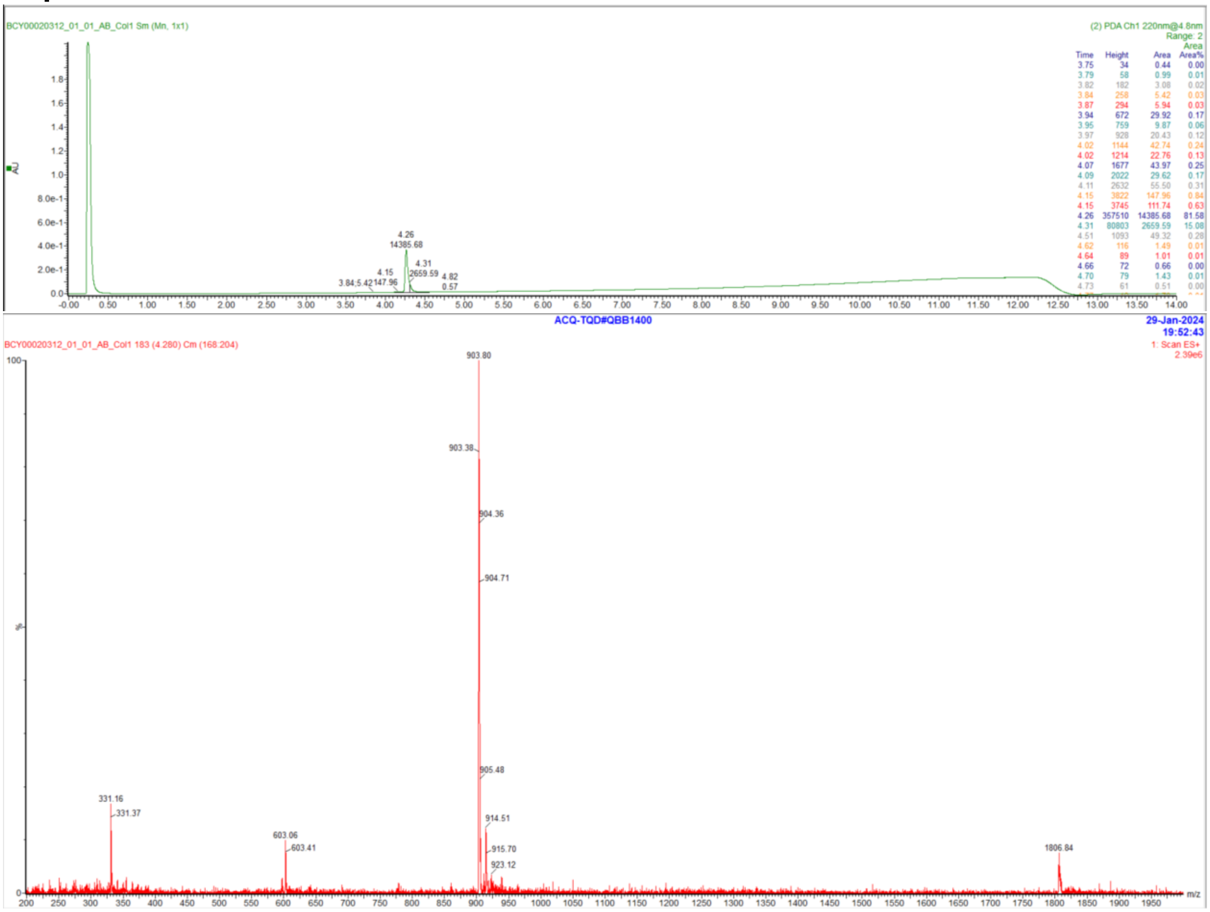

Peptide 39

Sample name: 101-08-26 (TATA) QC
Instrument: 1260_2
Injection date: 12/11/2019 2:17:40 PM
Acq. method: 0595B_AB_Poroshell120_15.5min.M

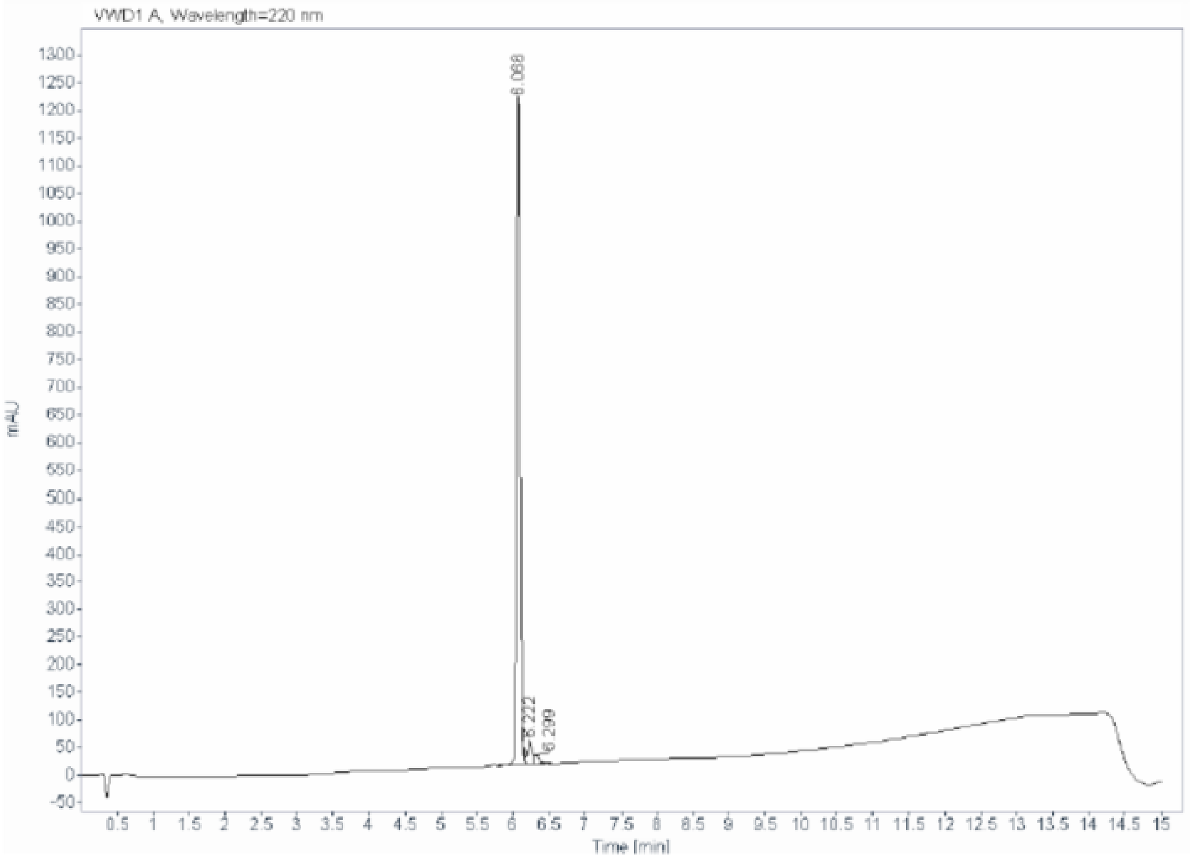

Signal: VWD1 A, Wavelength=220 nm

RT [min]	Type	Width [min]	Area	Height	Area%
6.068	BV R	0.0520	4026.8979	1203.2932	93.4396
6.222	VV E	0.0667	190.5921	41.4232	4.4225
6.299	VB E	0.0834	92.1372	16.9520	2.1379
		Sum	4309.6273		

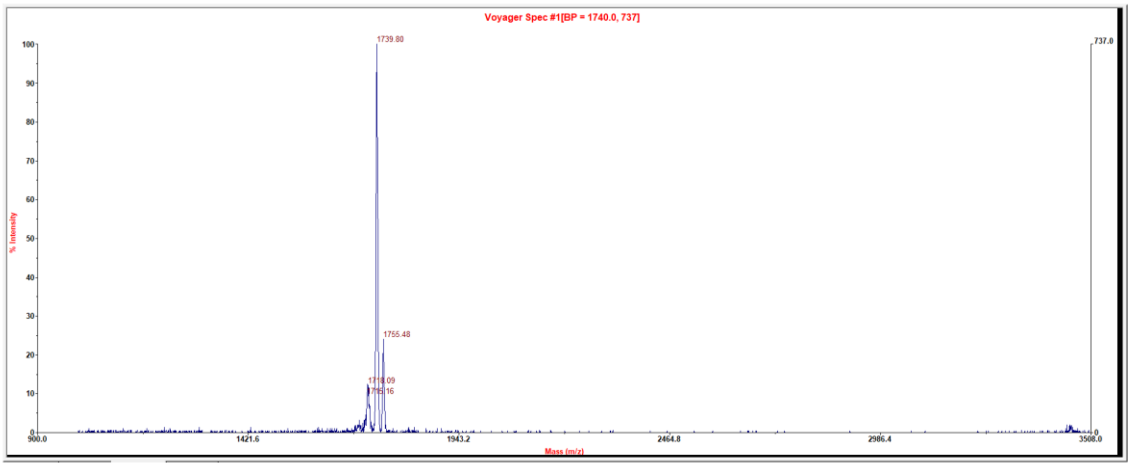

Peptide 40

Sample name:	101-08-29 (TATA) QC
Instrument:	1260_2
Injection date:	12/11/2019 3:21:52 PM
Acq. method:	0595B_AB_Poroshell120_15.5min.M

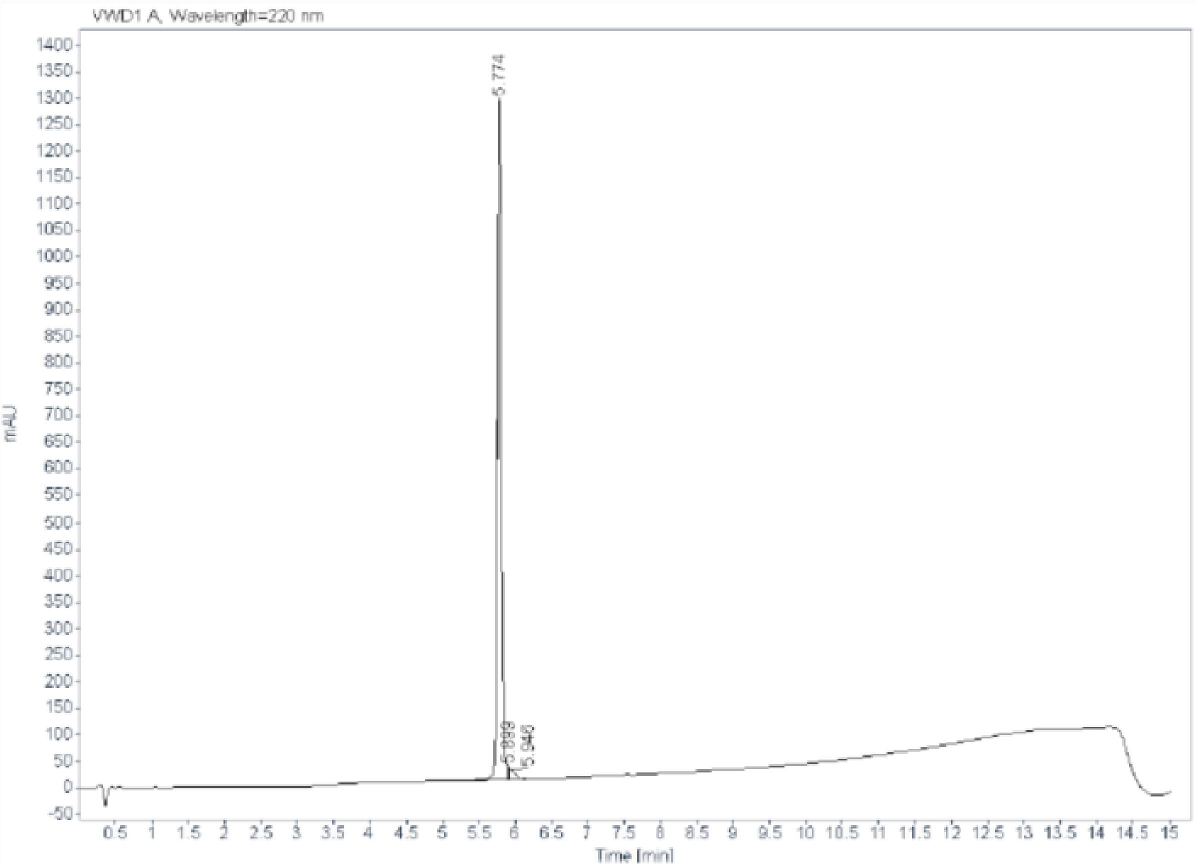

Signal: VWD1 A, Wavelength=220 nm

RT [min]	Type	Width [min]	Area	Height	Area%
5.774	VF R	0.0617	5143.9707	1284.1719	97.8242
5.899	VV	0.0157	22.5725	24.0308	0.4293
5.946	VV B	0.0747	91.8406	17.0967	1.7466
		Sum	5258.3838		

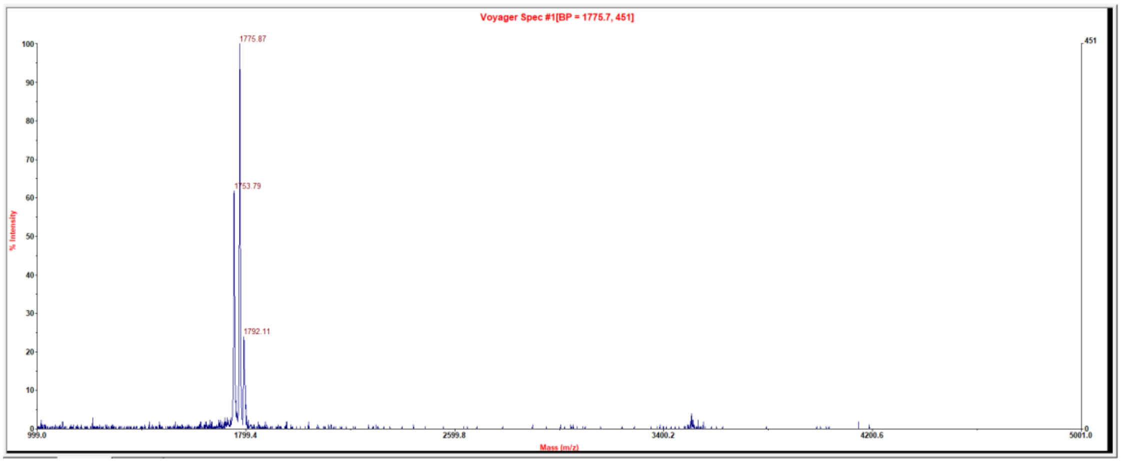

Peptide 41

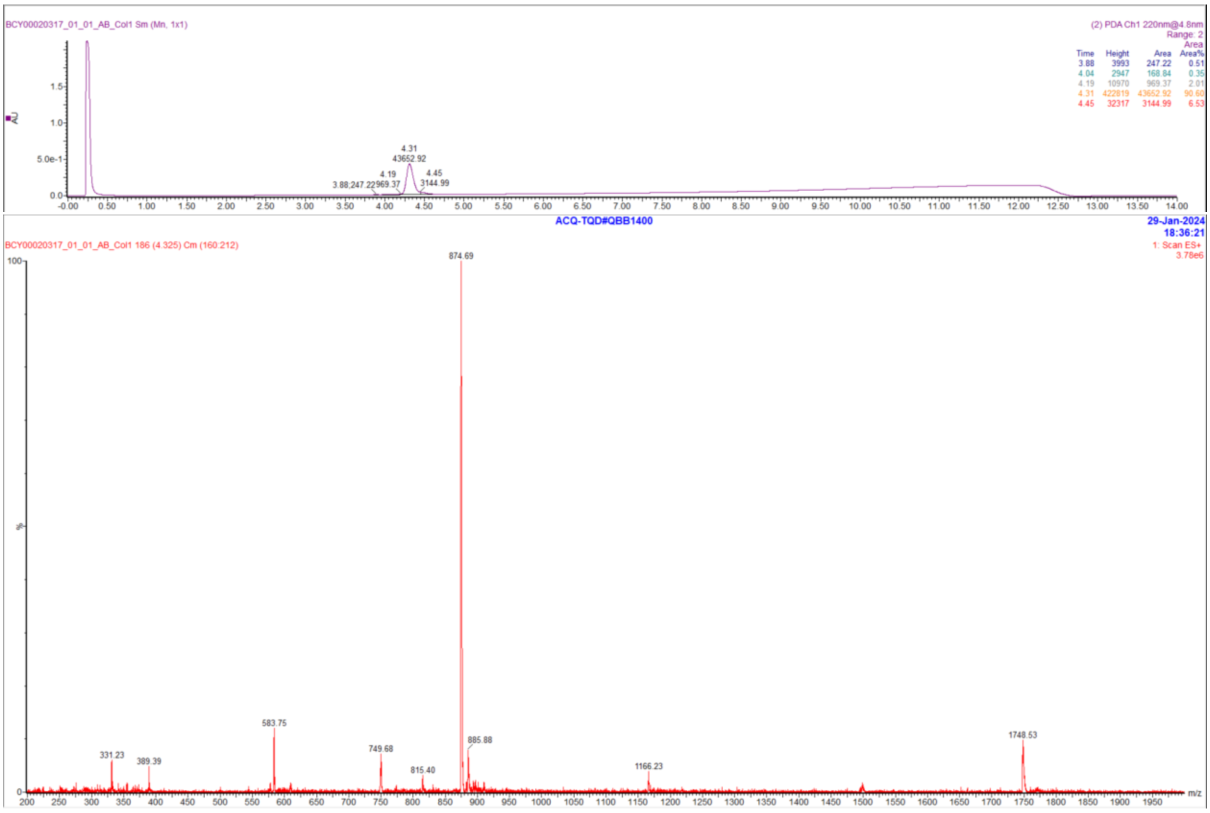

Peptide 42

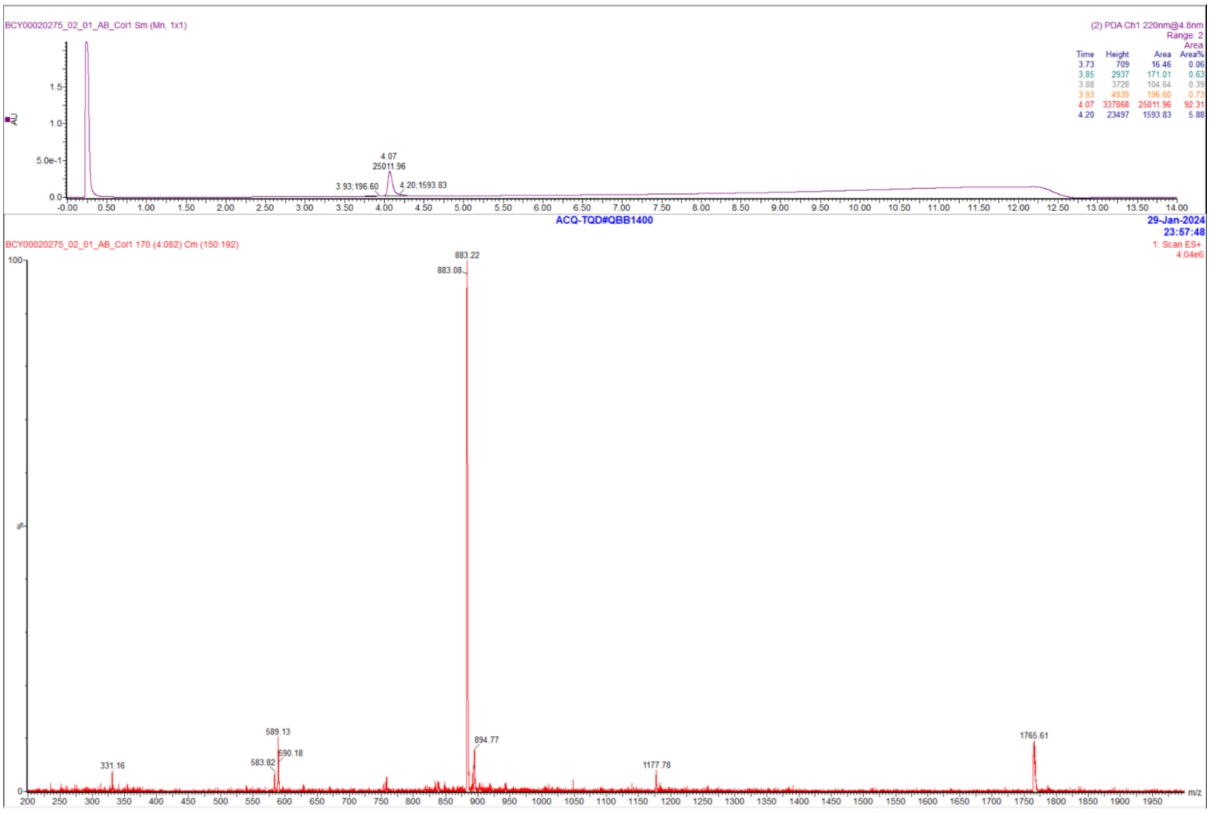

Peptide 43

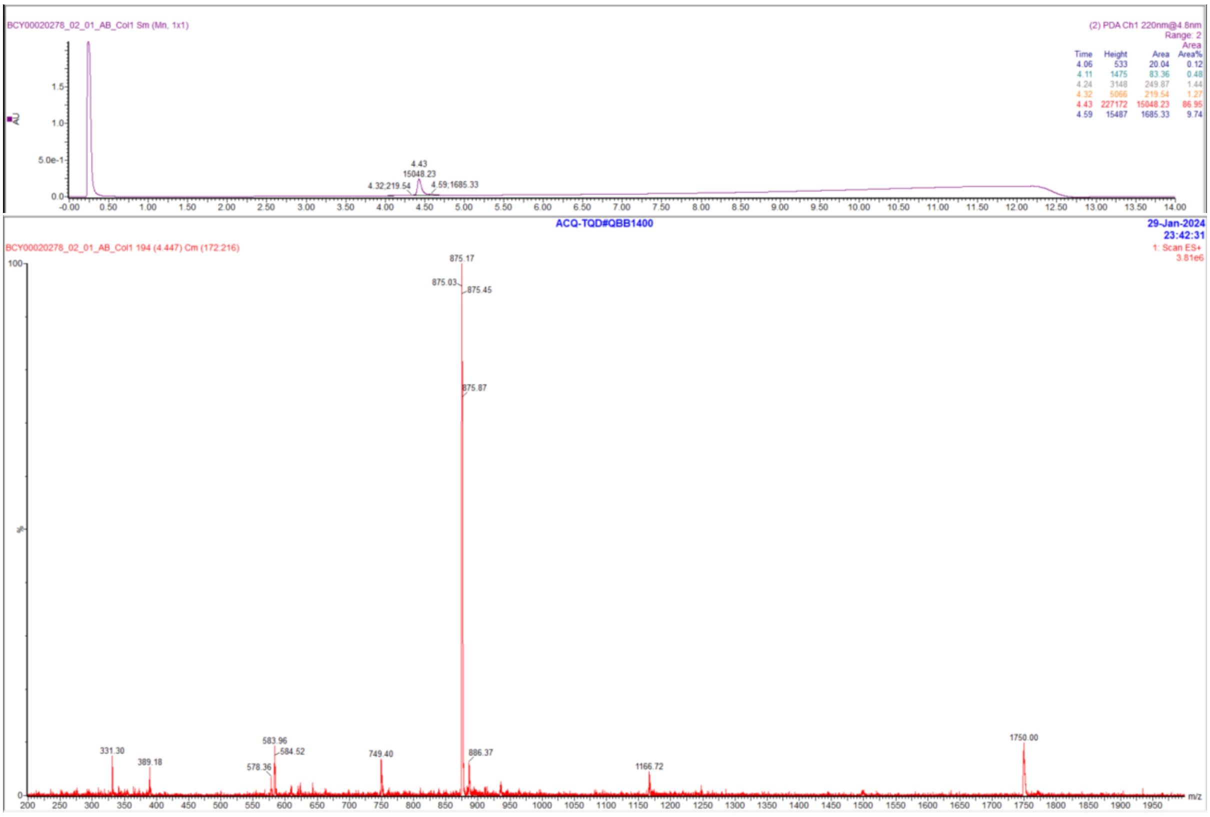

Peptide 44

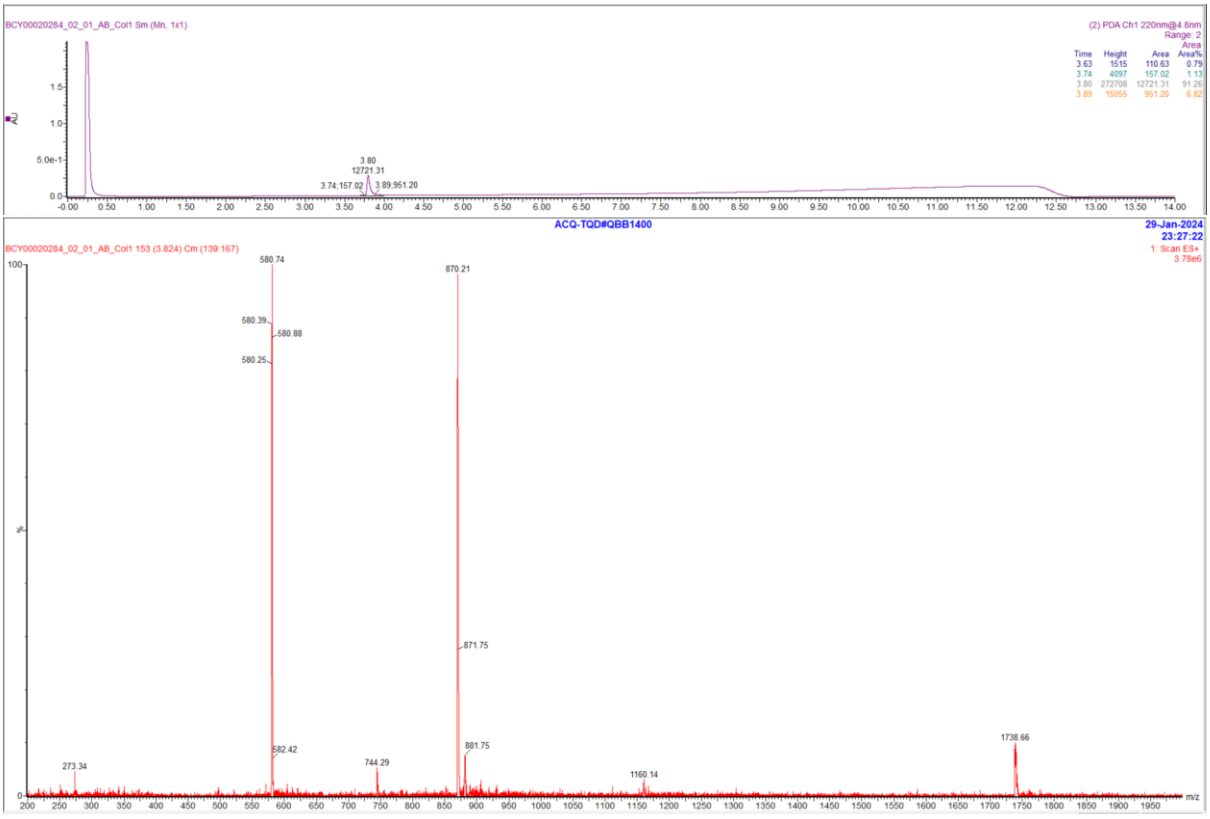

Peptide 45

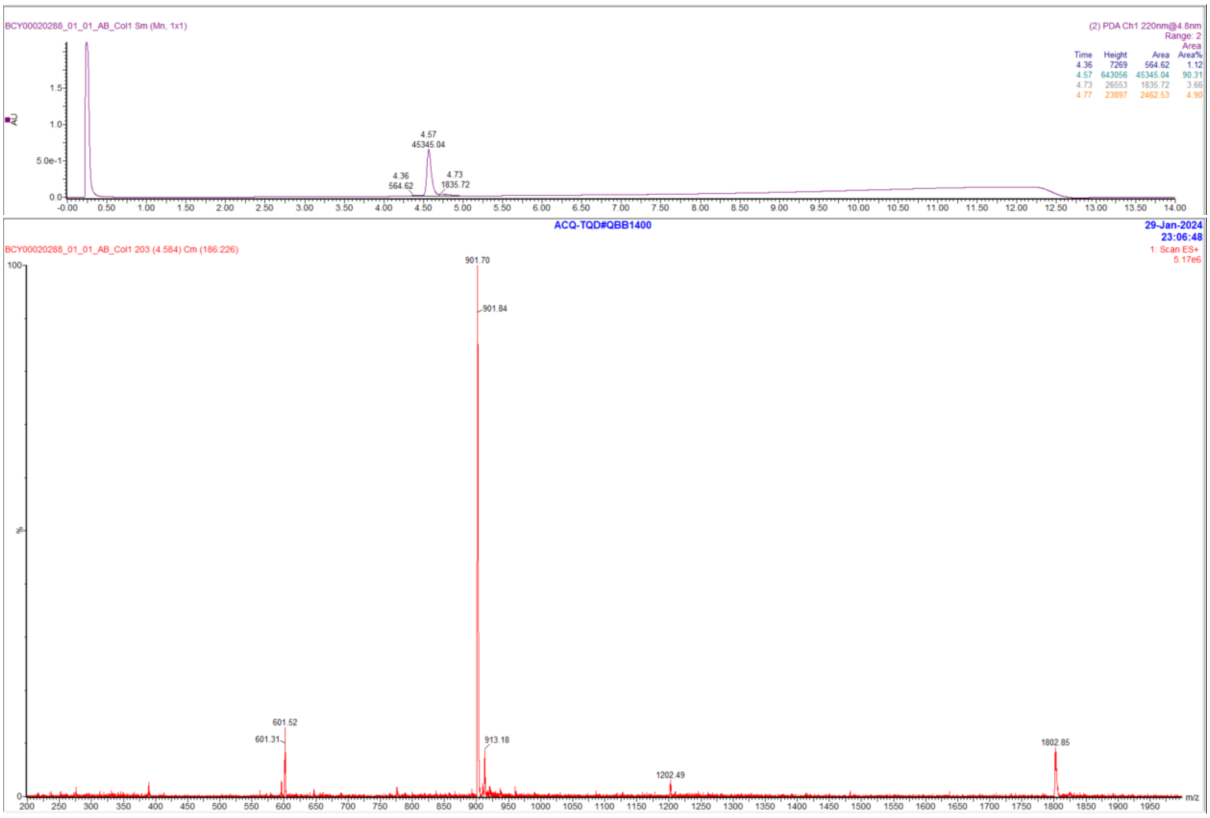

Peptide 46

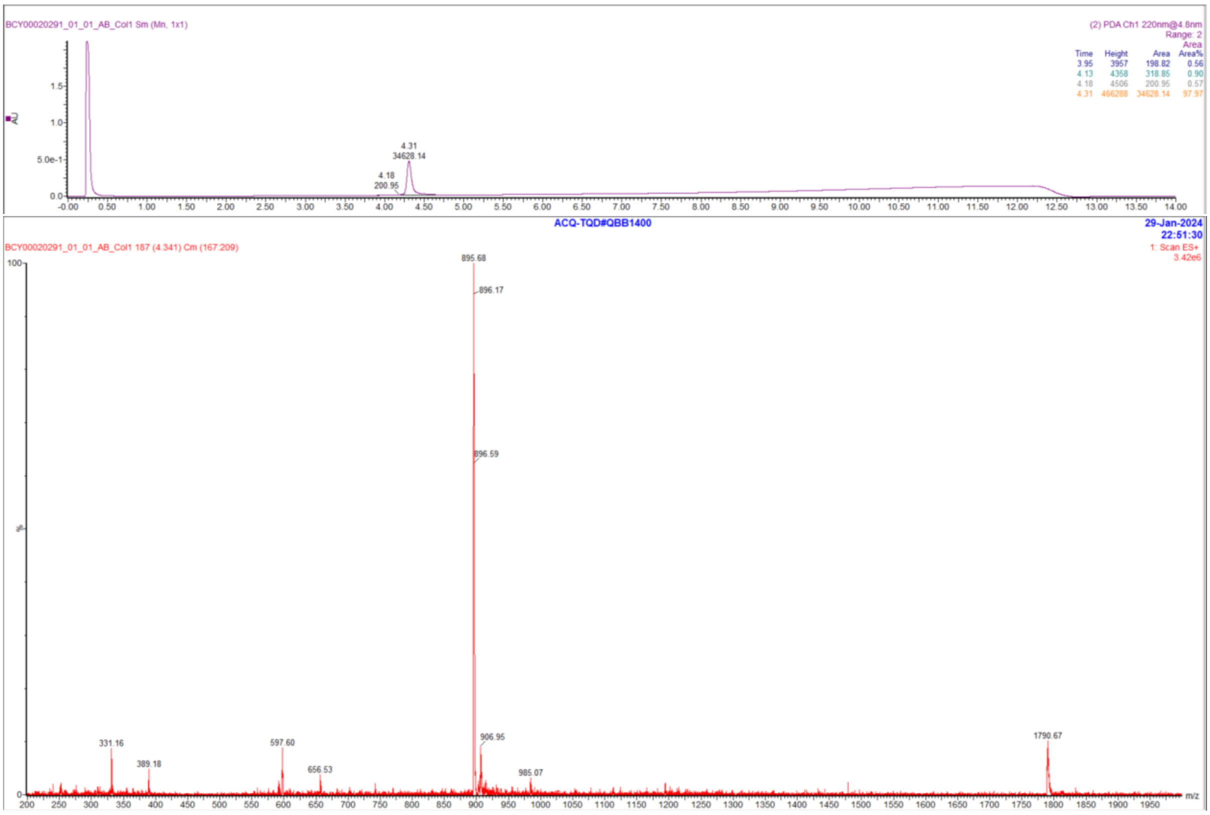

Peptide 47

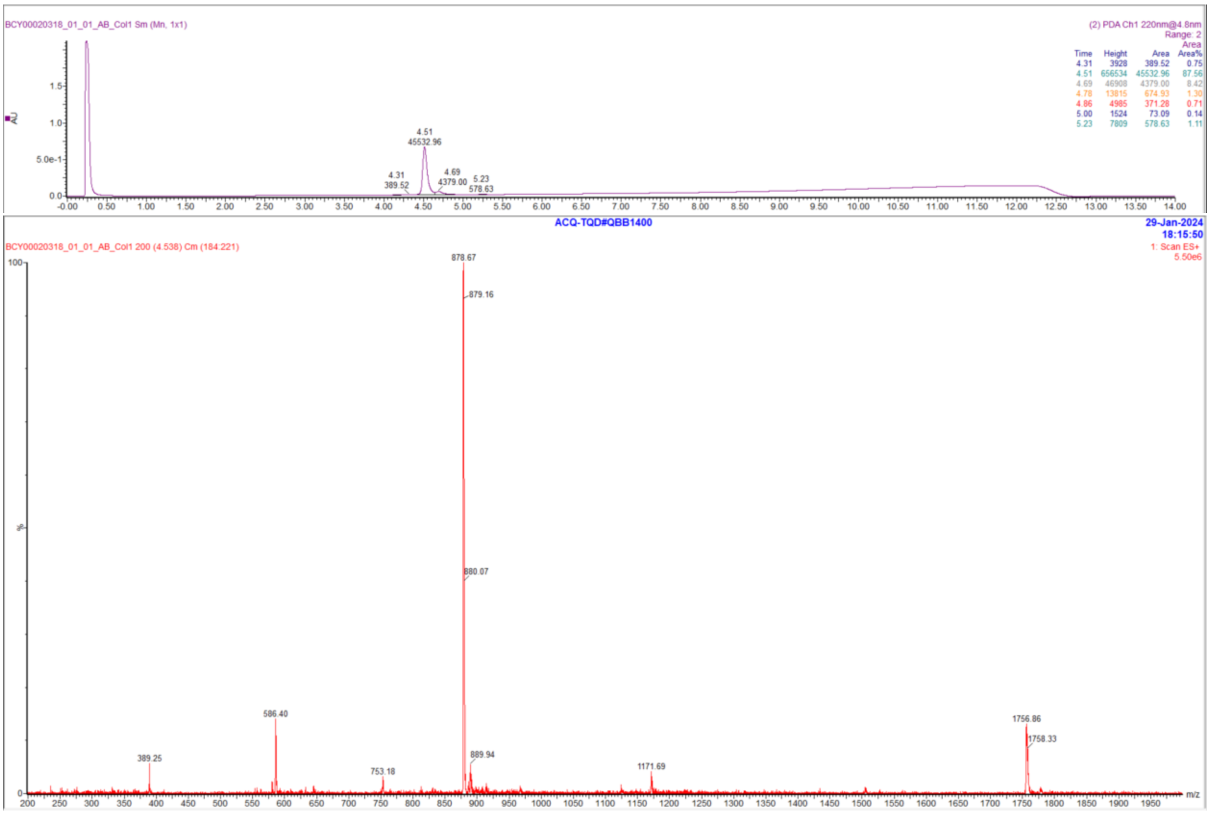

Peptide 48

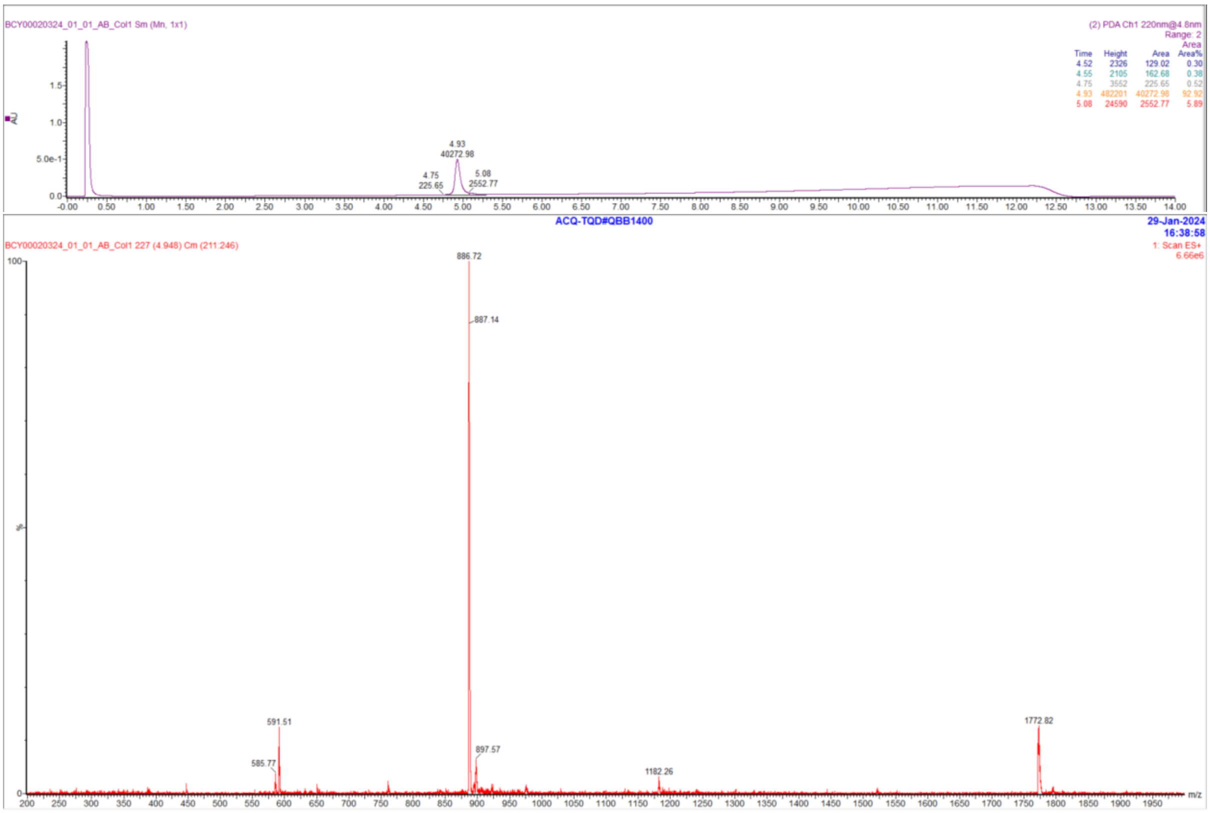

Peptide 49

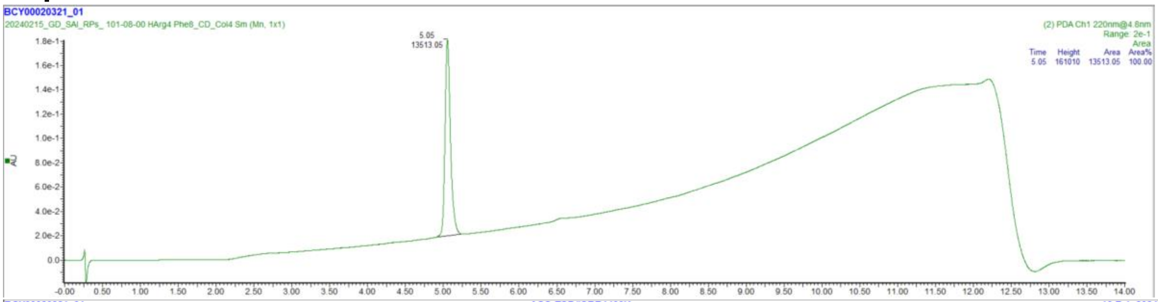

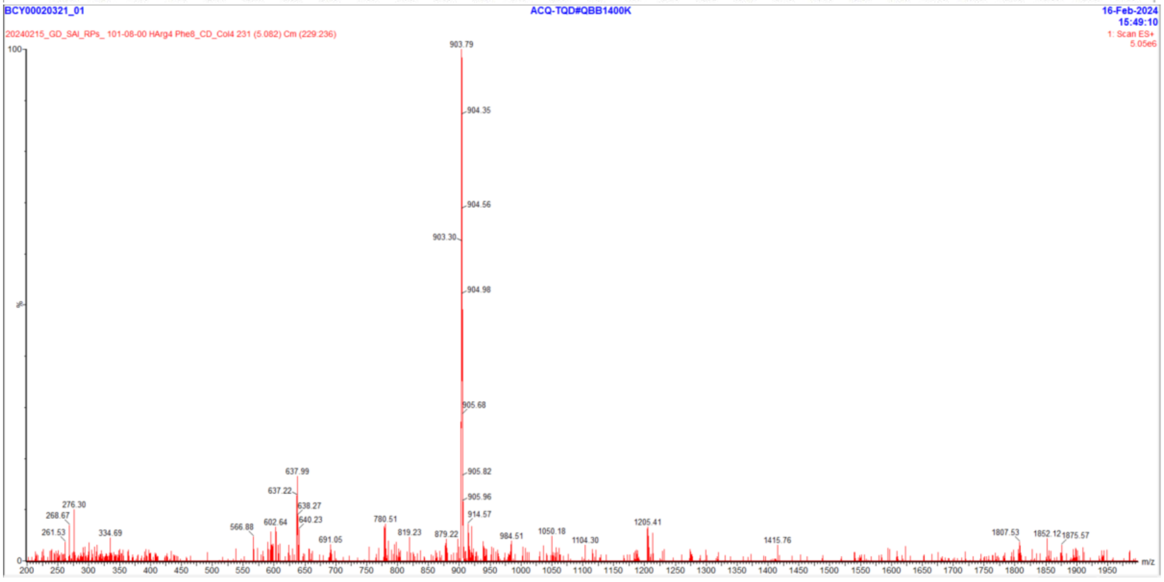

Peptide 50

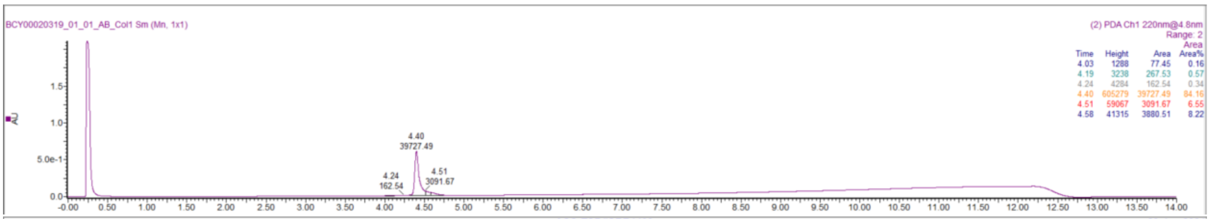

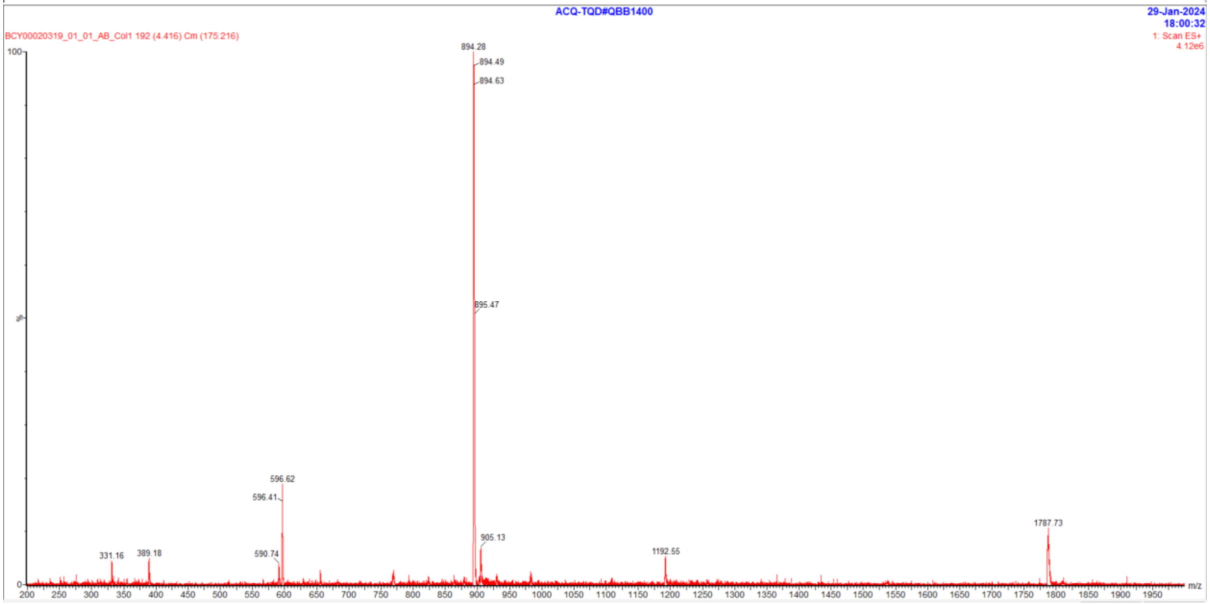

Peptide 51

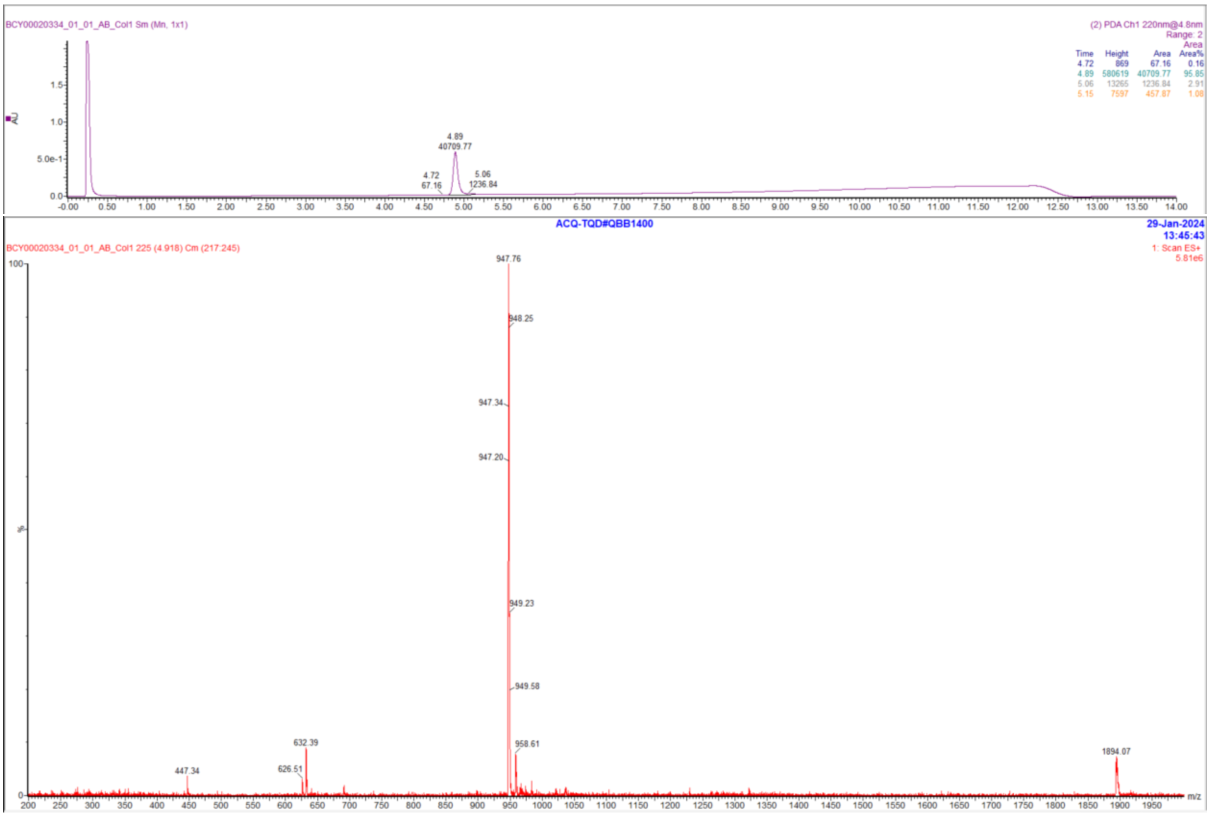

Peptide 52

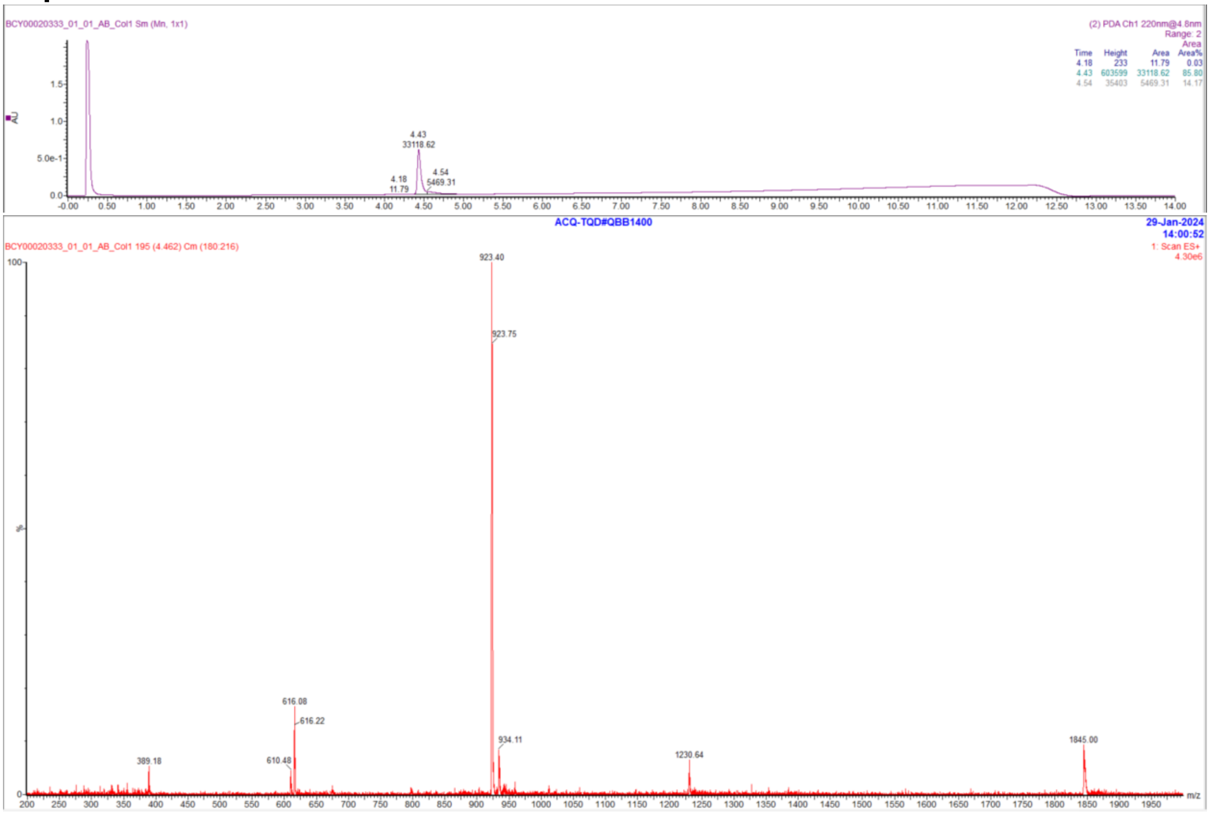

Peptide 53

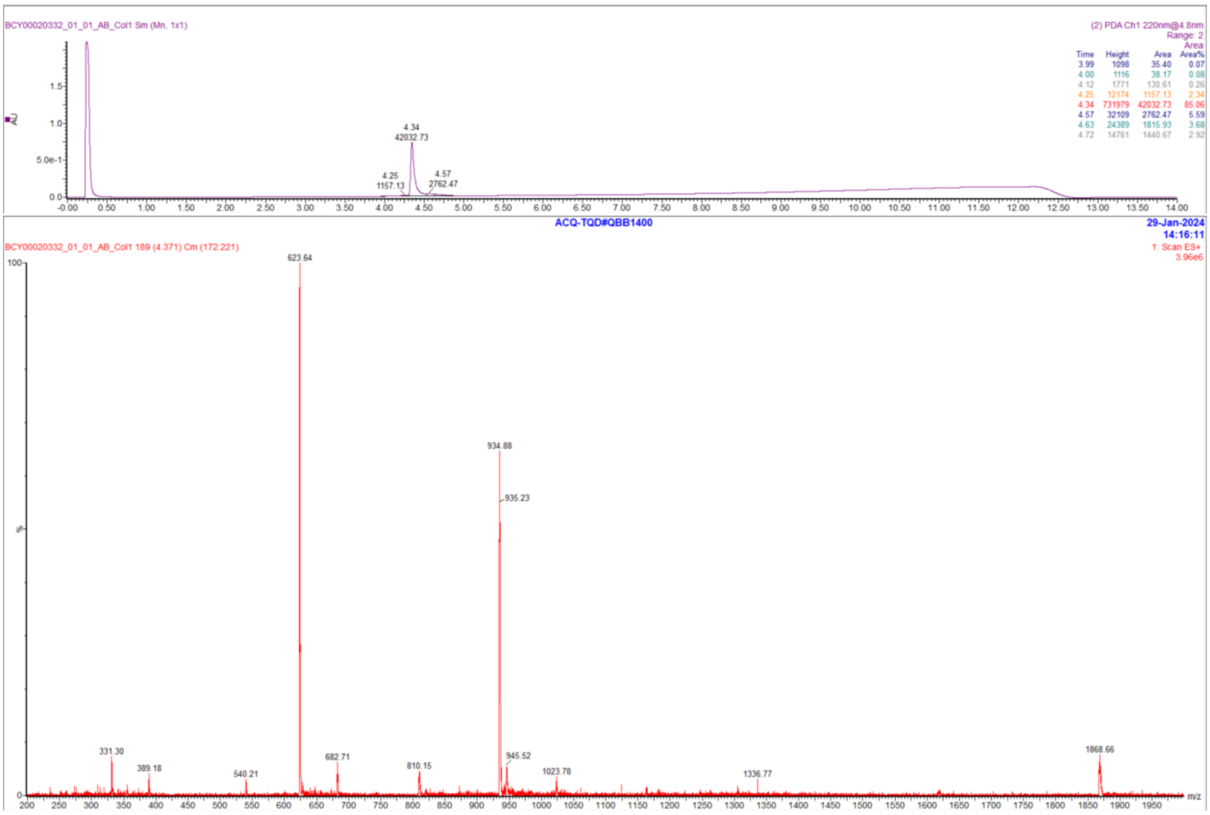

BCY00020332_01_01_AB_Col1 Sm (Mn, 1x1)
(2) PDA Ch1 220nm@4.8nm
Range: 2
Time Height Area Area%
3.99 1098 35.40 0.07
4.00 1116 38.17 0.08
4.12 1771 130.61 0.26
4.25 12174 1157.13 2.34
4.34 731979 42032.73 85.06
4.57 32109 2762.47 5.59
4.63 24389 1815.93 3.68
4.72 14761 1440.67 2.92
4.34
42032.73
4.25
1157.13
4.57
2762.47
AU
ACQ-TQD#QBB1400
29-Jan-2024
14:16:11
BCY00020332_01_01_AB_Col1 189 (4.371) Cm (172:221)
1: Scan ES+
3.96e6
623.64
934.88
935.23
331.30
389.18
540.21
682.71
810.15
945.52
1023.78
1336.77
1868.66
%
m/z

Peptide 54

Data file: D:\Chemstation\1\Data\Peptides_facon\7000-7999
_BCY00016387_01_7564_A_zHPLC 13-03-54.D

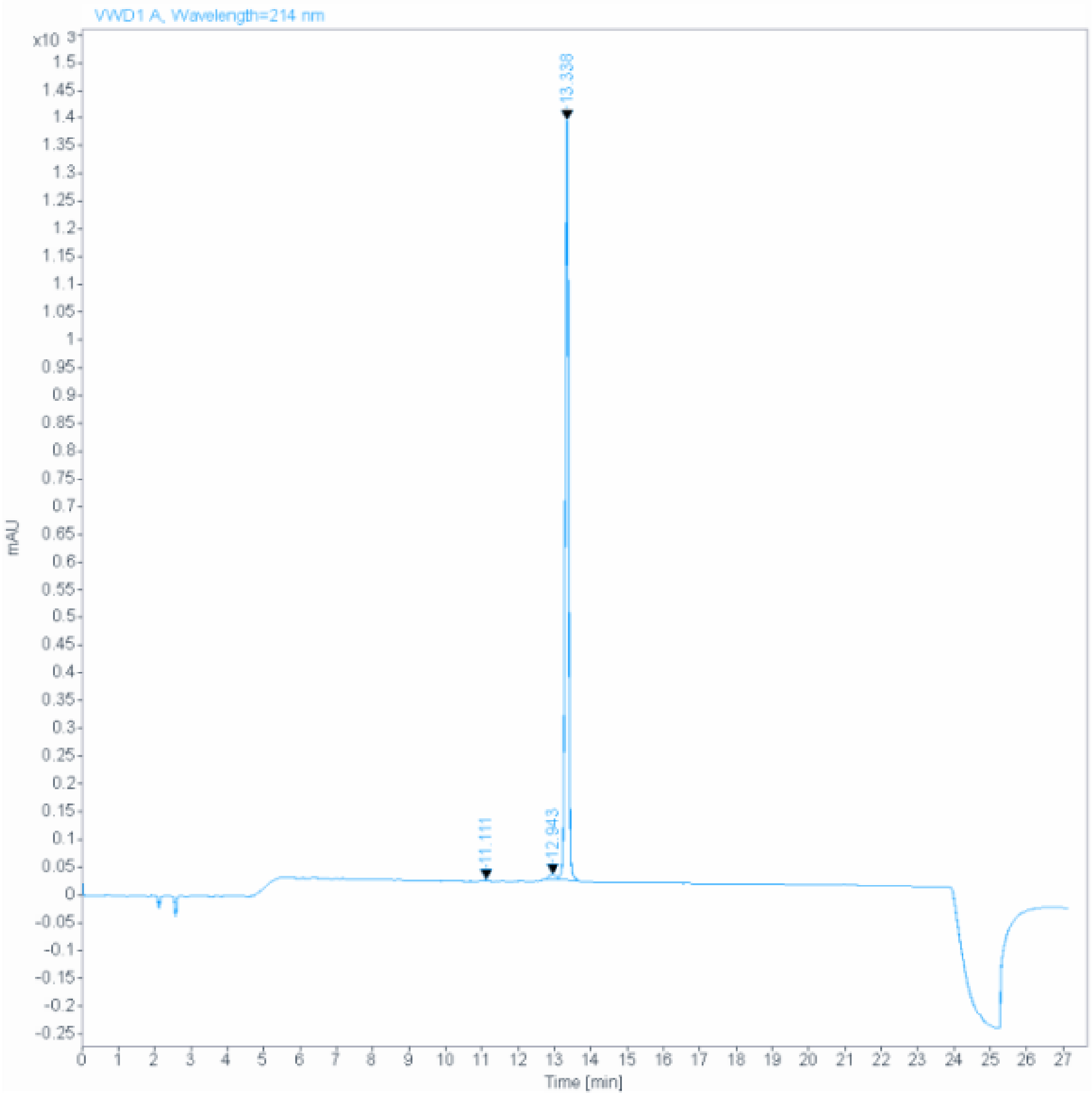

Signal: VWD1 A, Wavelength=214 nm

RT [min]	Type	Width [min]	Area	Height	Area%	Name
11.111	BB	0.0569	12.7126	3.2343	0.1433	
12.943	MF	0.2437	166.6561	11.3989	1.8791	
13.338	FM	0.1056	8689.7803	1371.4633	97.9776	
		Sum	8869.1490			

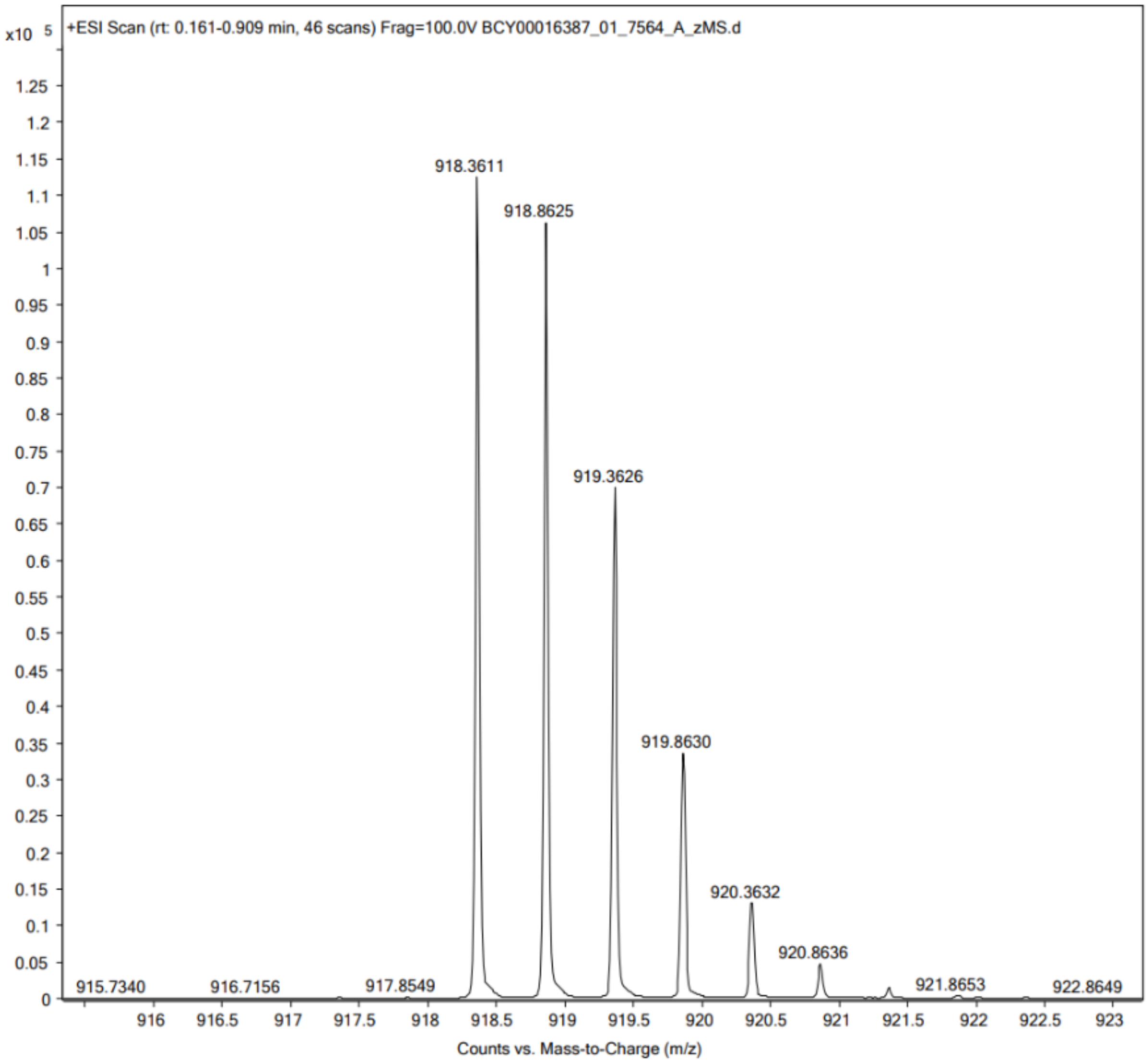
x10 5
+ESI Scan (rt: 0.161-0.909 min, 46 scans) Frag=100.0V BCY00016387_01_7564_A_zMS.d
918.3611
918.8625
919.3626
919.8630
920.3632
920.8636
915.7340
916.7156
917.8549
921.8653
922.8649
Counts vs. Mass-to-Charge (m/z)

Peptide 55

Data file: D:\Chemstation\1\Data\Peptides_facon\7000-7999_BCY00016385_01_7562_A_zHPLC 11-55-44.D

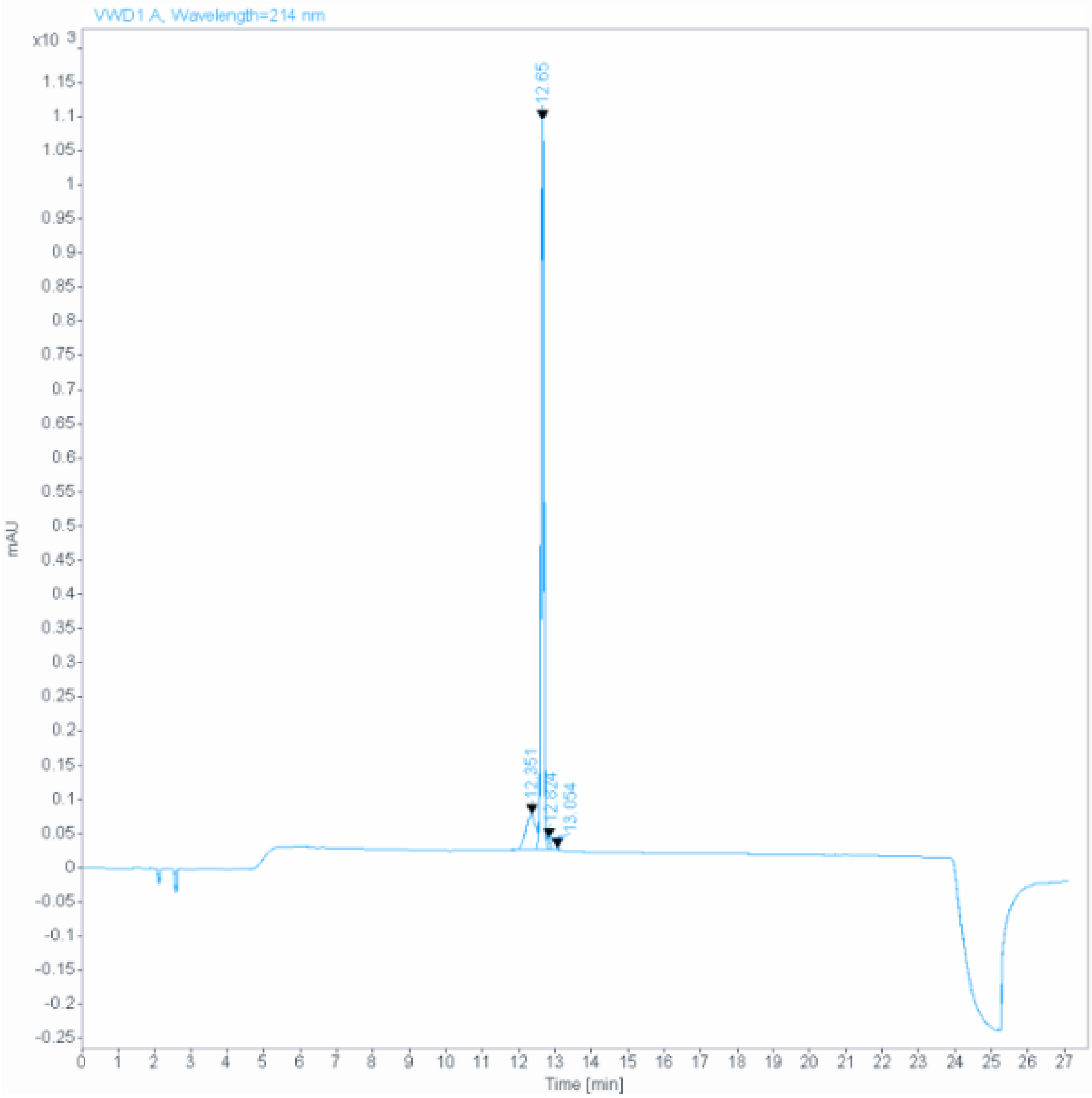

Signal: VWD1 A, Wavelength=214 nm

RT [min]	Type	Width [min]	Area	Height	Area%	Name
12.351	BV E	0.2492	1018.3990	51.9577	16.6186	
12.650	VV R	0.0716	5004.1411	1068.6205	81.6593	
12.824	VB E	0.0832	96.2061	16.3371	1.5699	
13.054	MM	0.0538	9.3291	2.8900	0.1522	
		Sum	6128.0753			

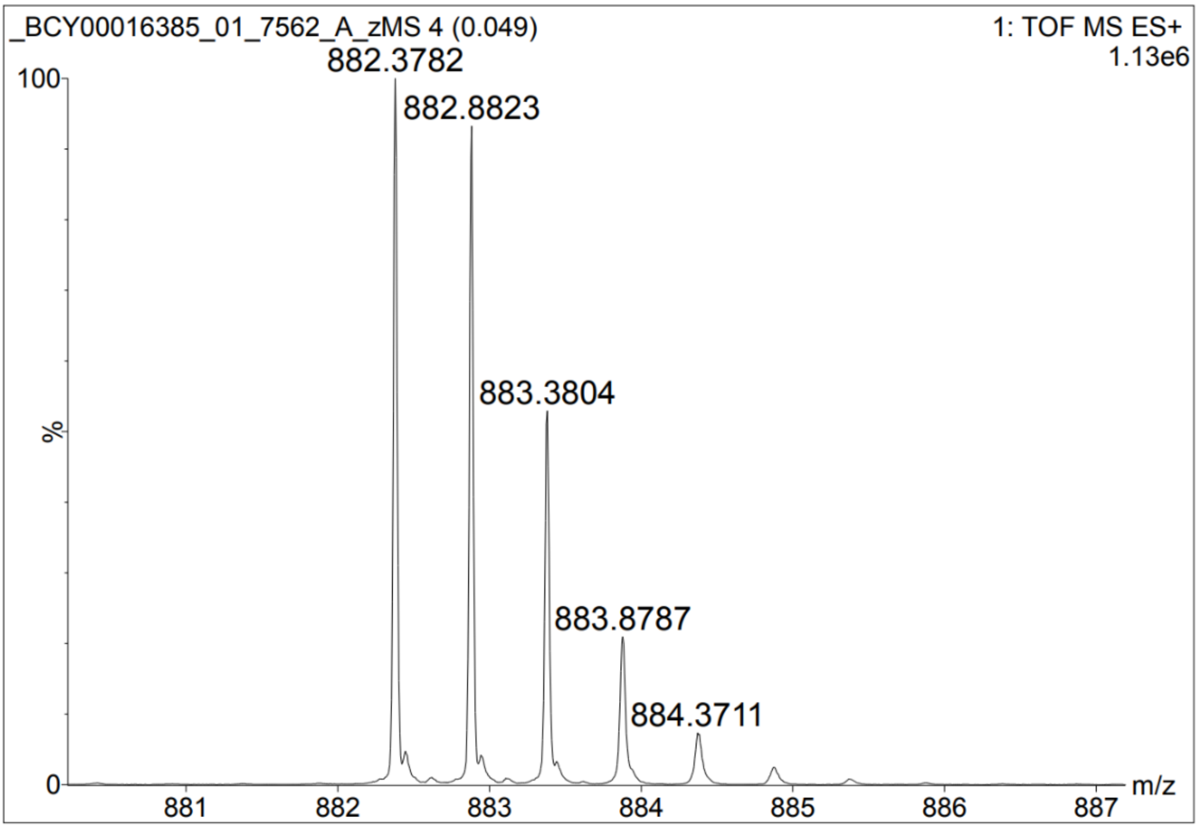

_BCY00016385_01_7562_A_zMS 4 (0.049)
1: TOF MS ES+
1.13e6
882.3782
882.8823
883.3804
883.8787
884.3711
100
%
0
881
882
883
884
885
886
887
m/z

Peptide 56

Data file: D:\Chemstation\1\Data\Peptides_facon\7000-7999_BCY00016384_01_7561_A_zHPLC 12-38-51.D

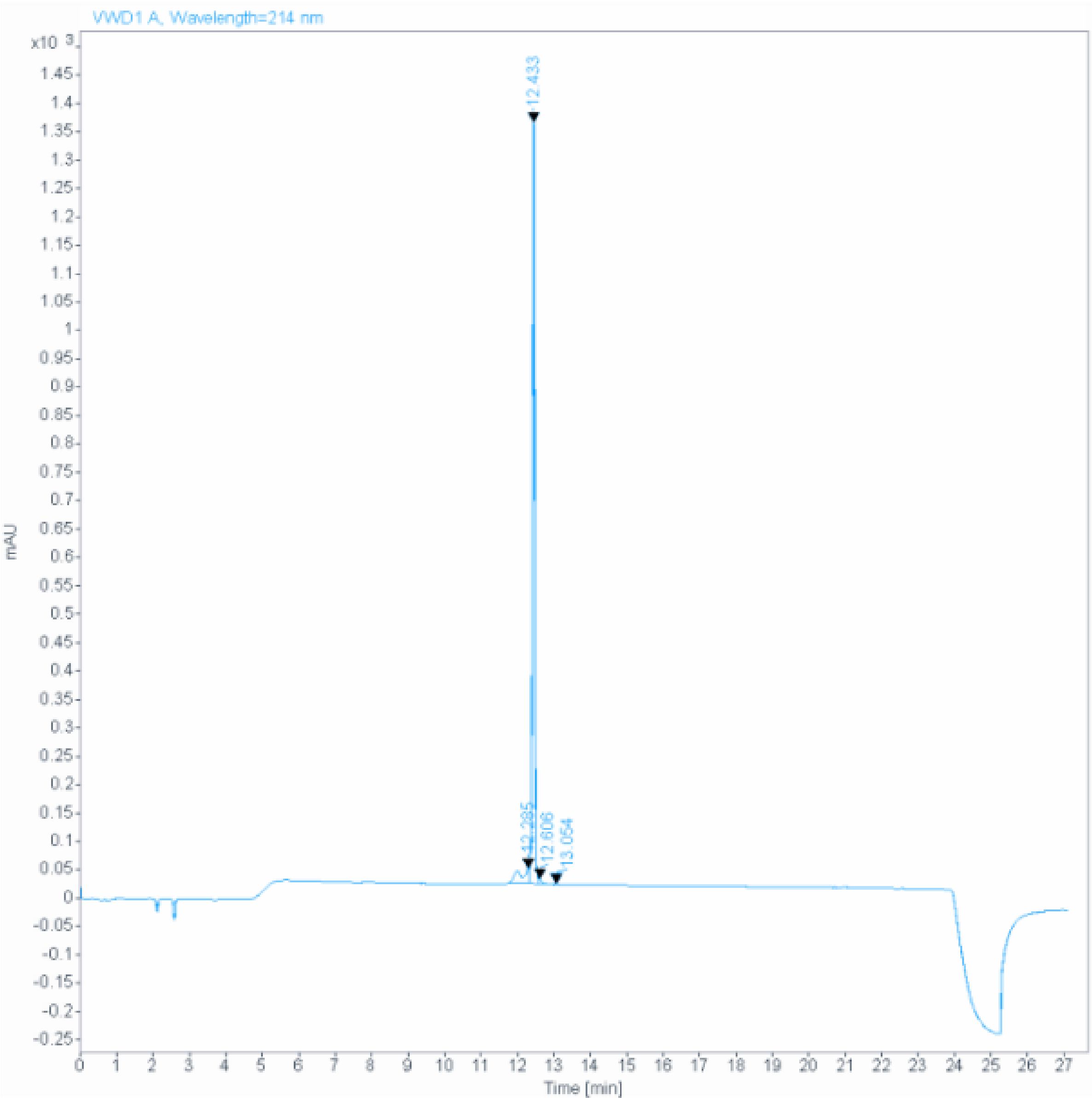

Signal: VWD1 A, Wavelength=214 nm

RT [min]	Type	Width [min]	Area	Height	Area%	Name
12.285	MF	0.3117	496.0348	26.5213	7.5037	
12.433	MF	0.0753	6061.4360	1341.2587	91.6936	
12.606	FM	0.0872	47.8876	9.1495	0.7244	
13.054	MM	0.0490	5.1769	1.7622	0.0783	
		Sum	6610.5353			

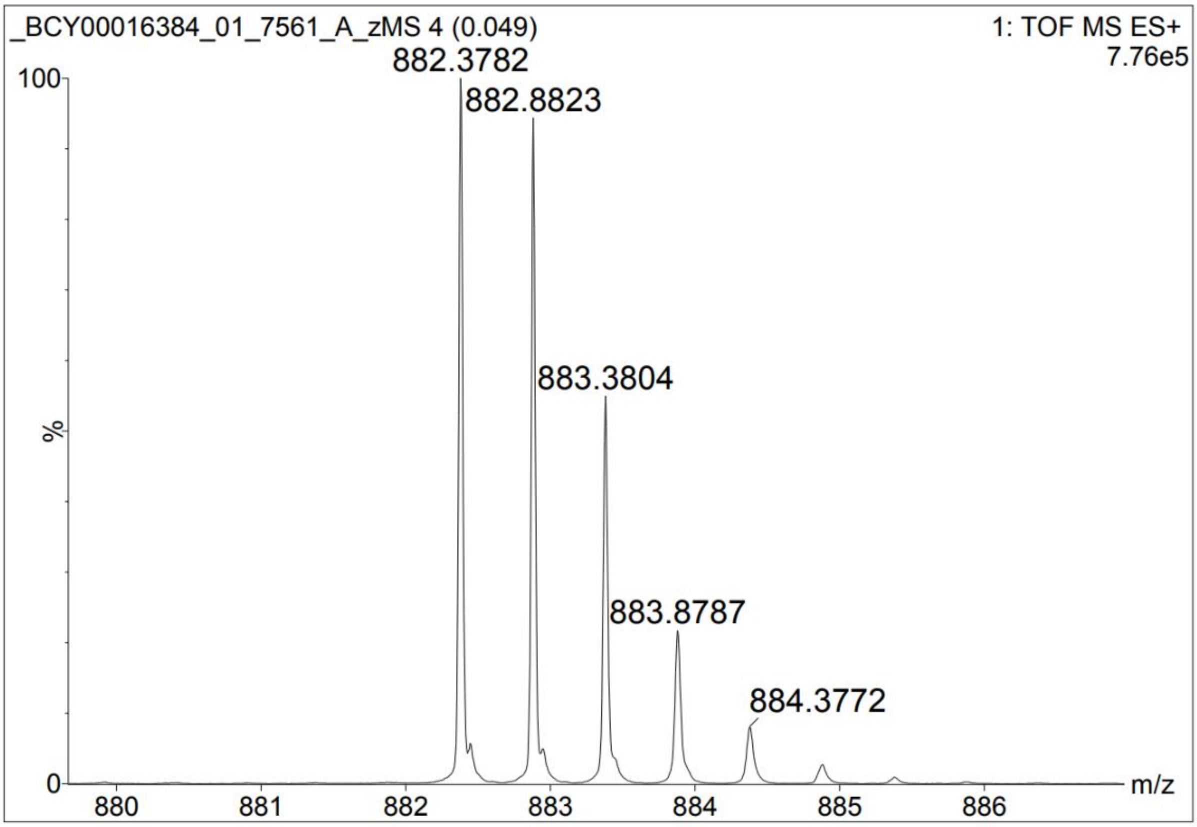

_BCY00016384_01_7561_A_zMS 4 (0.049)
1: TOF MS ES+
7.76e5
882.3782
882.8823
883.3804
883.8787
884.3772
100
%
0
880
881
882
883
884
885
886
m/z

Peptide 57

Data file: D:\Chemstation\1\Data\Peptides_facon\7000-7999_BCY00016381_01_7558_A_zHPLC 11-35-52.D

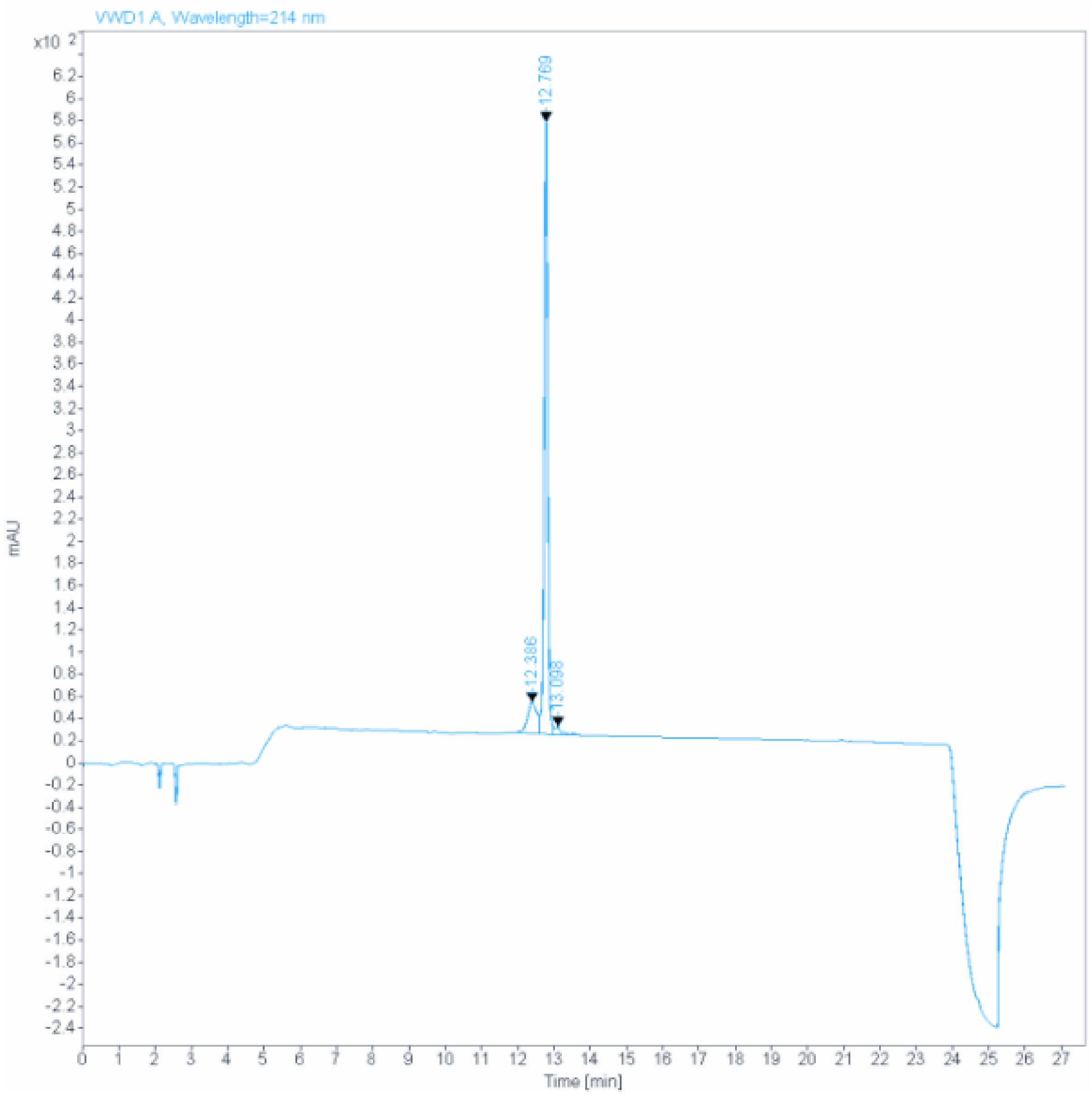

Signal: VWD1 A, Wavelength=214 nm

RT [min]	Type	Width [min]	Area	Height	Area%	Name
12.386	MF	0.3190	547.3634	28.5988	12.5099	
12.769	FM	0.1119	3717.5630	553.5273	84.9645	
13.098	FM	0.2575	110.5052	7.1529	2.5256	
		Sum	4375.4316			

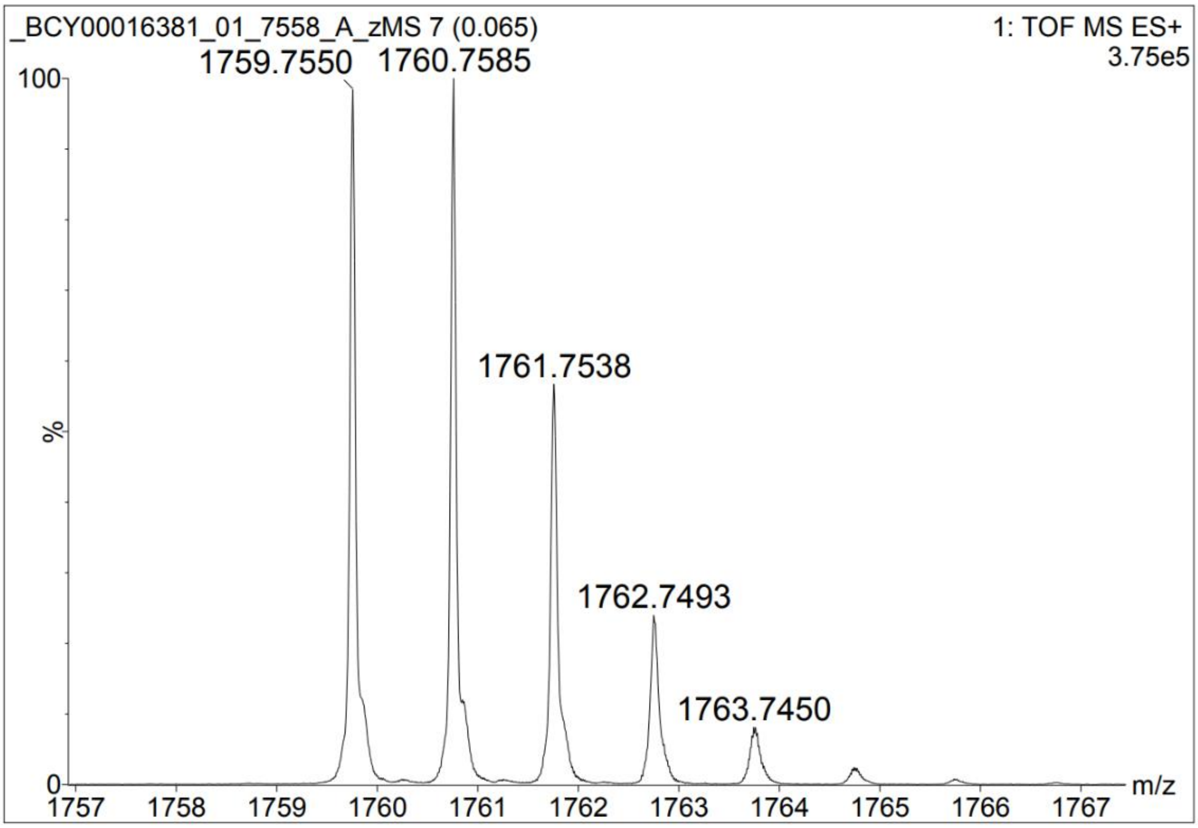
_BCY00016381_01_7558_A_zMS 7 (0.065)
1: TOF MS ES+
3.75e5
1759.7550
1760.7585
1761.7538
1762.7493
1763.7450
100
%
0
1757
1758
1759
1760
1761
1762
1763
1764
1765
1766
1767
m/z

Peptide 58

Data file: D:\Chemstation\1\Data\Peptides_facon\7000-7999_BCY00016383_01_7560_A_zHPLC 12-14-55.D

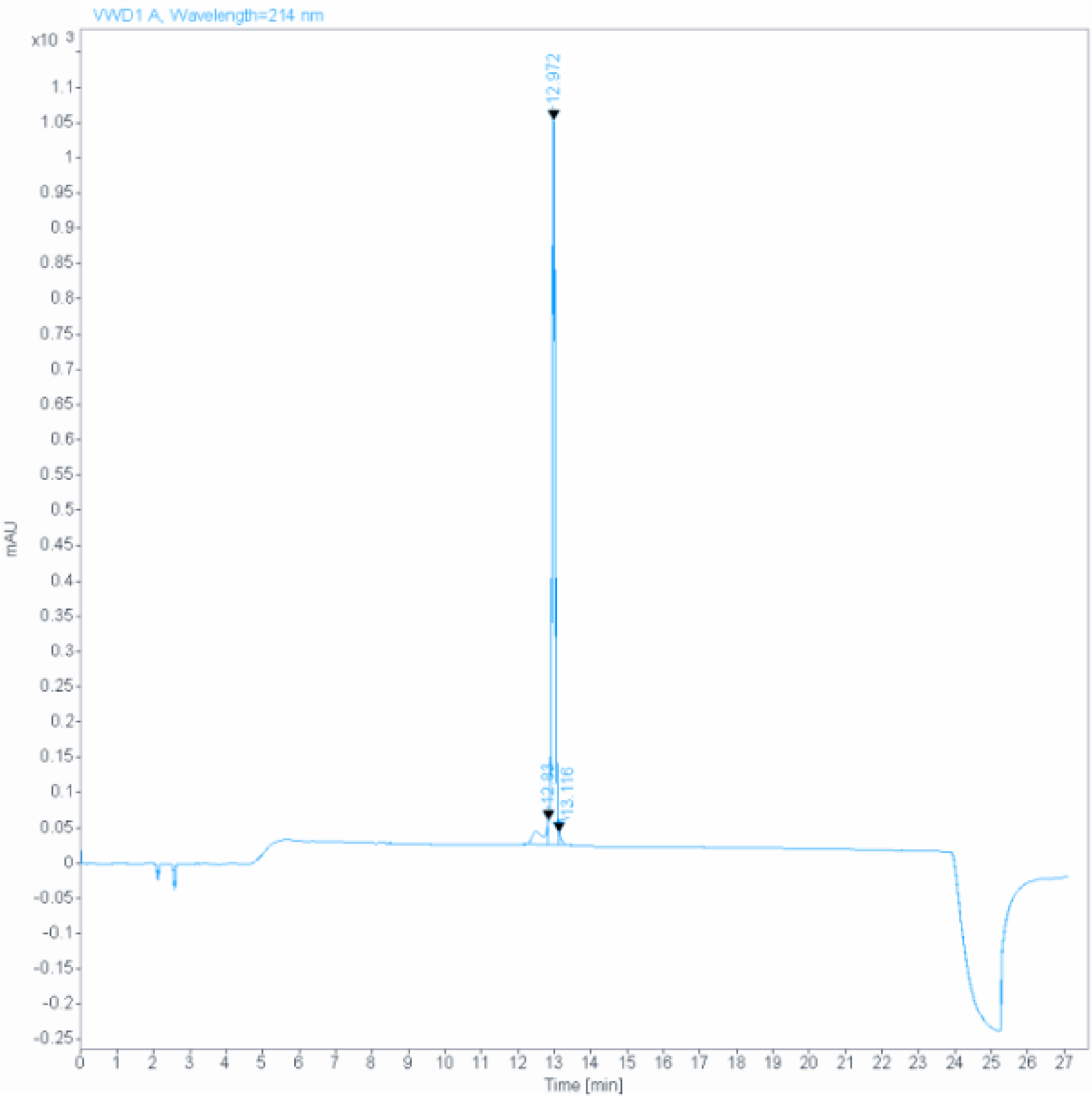

Signal: VWD1 A, Wavelength=214 nm

RT [min]	Type	Width [min]	Area	Height	Area%	Name
12.830	MF	0.2309	484.2571	34.9524	7.3136	
12.972	MF	0.0982	6056.5078	1028.3544	91.4700	
13.116	FM	0.0731	80.5382	18.3675	1.2163	
		Sum	6621.3031			

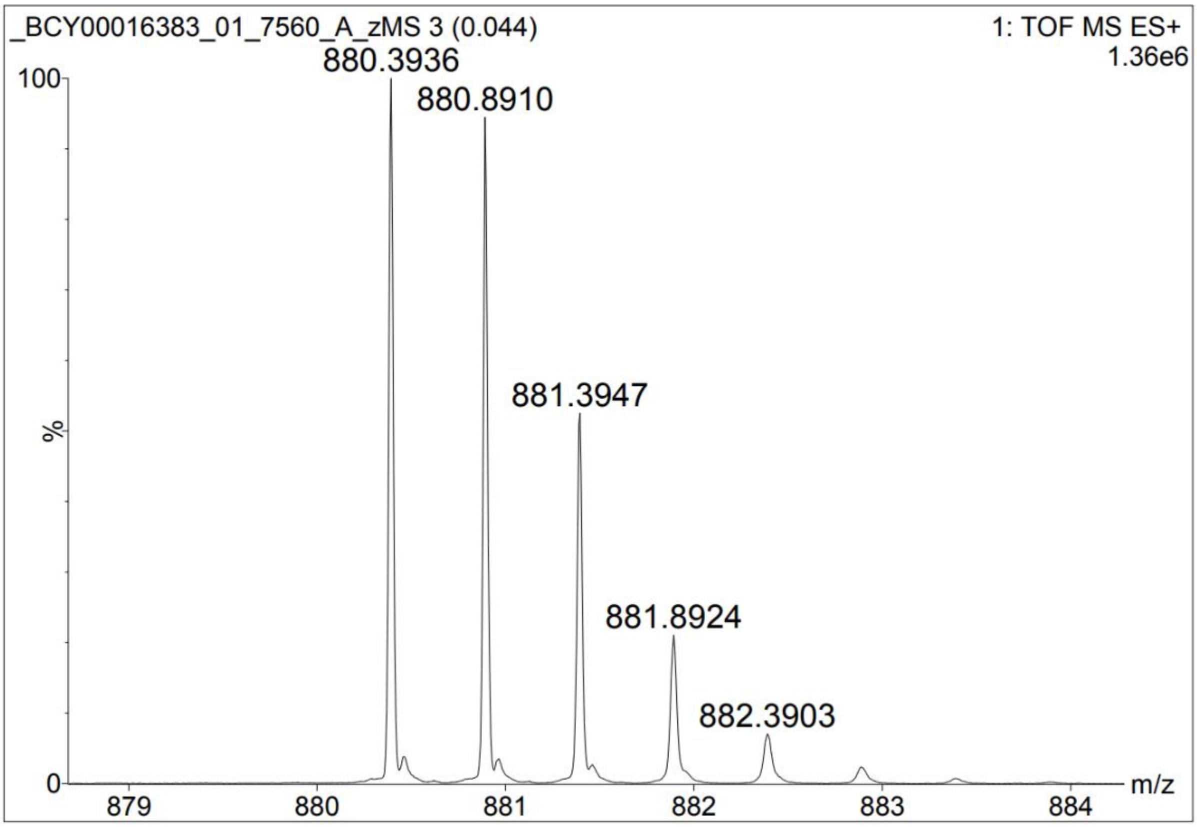

_BCY00016383_01_7560_A_zMS 3 (0.044)
1: TOF MS ES+
1.36e6
880.3936
880.8910
881.3947
881.8924
882.3903
100
%
0
879
880
881
882
883
884
m/z

Peptide 59

Data file: D:\Chemstation\1\Data\Peptides_facon\7000-7999_BCY00016392_01_7569_A_zHPLC 12-32-22.D

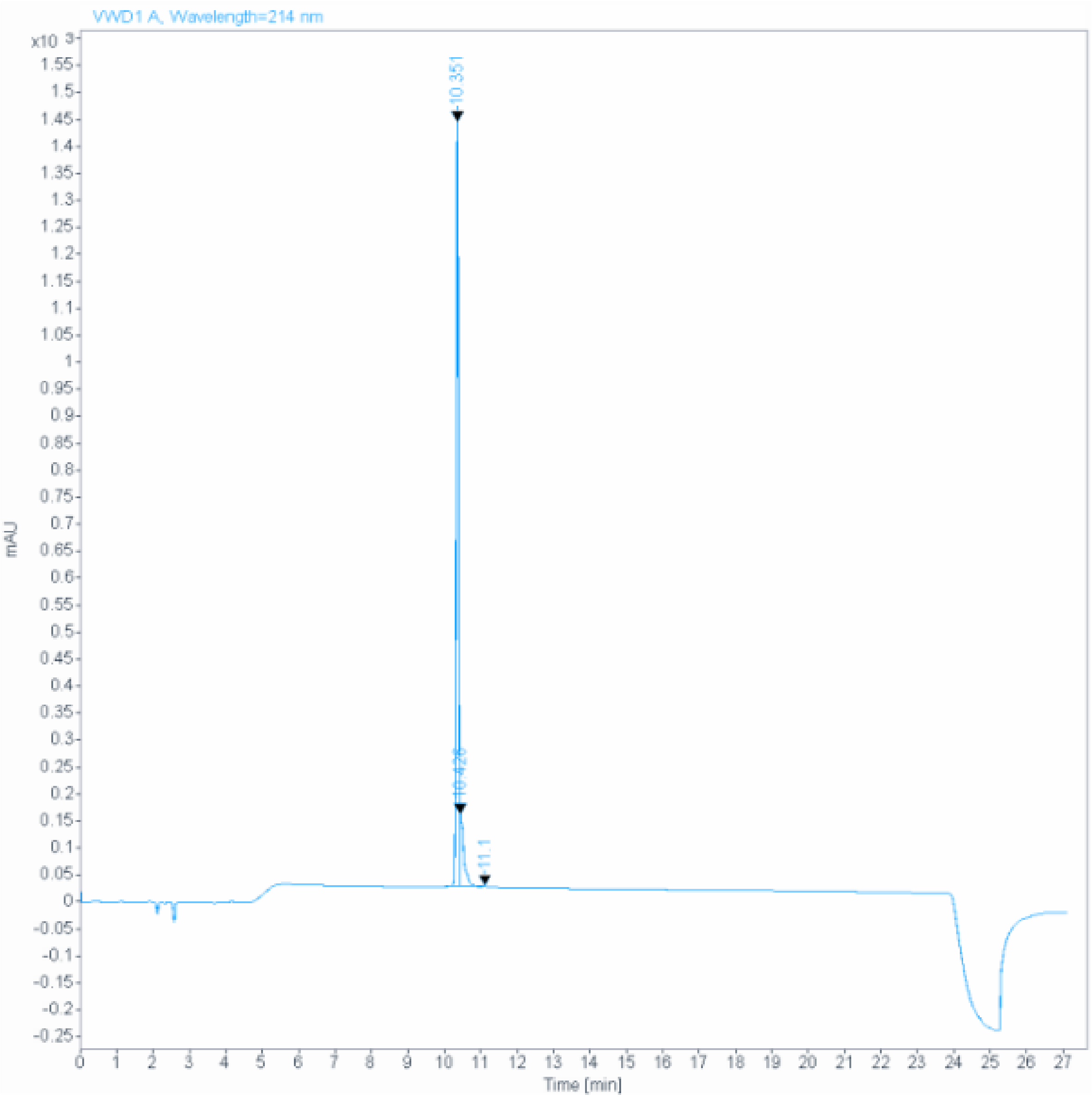

Signal: VWD1 A, Wavelength=214 nm

RT [min]	Type	Width [min]	Area	Height	Area%	Name
10.351	MF	0.0660	5622.1333	1419.0532	85.9266	
10.426	FM	0.1119	906.7505	135.0147	13.8584	
11.100	BB	0.0593	14.0652	3.5067	0.2150	
		Sum	6542.9490			

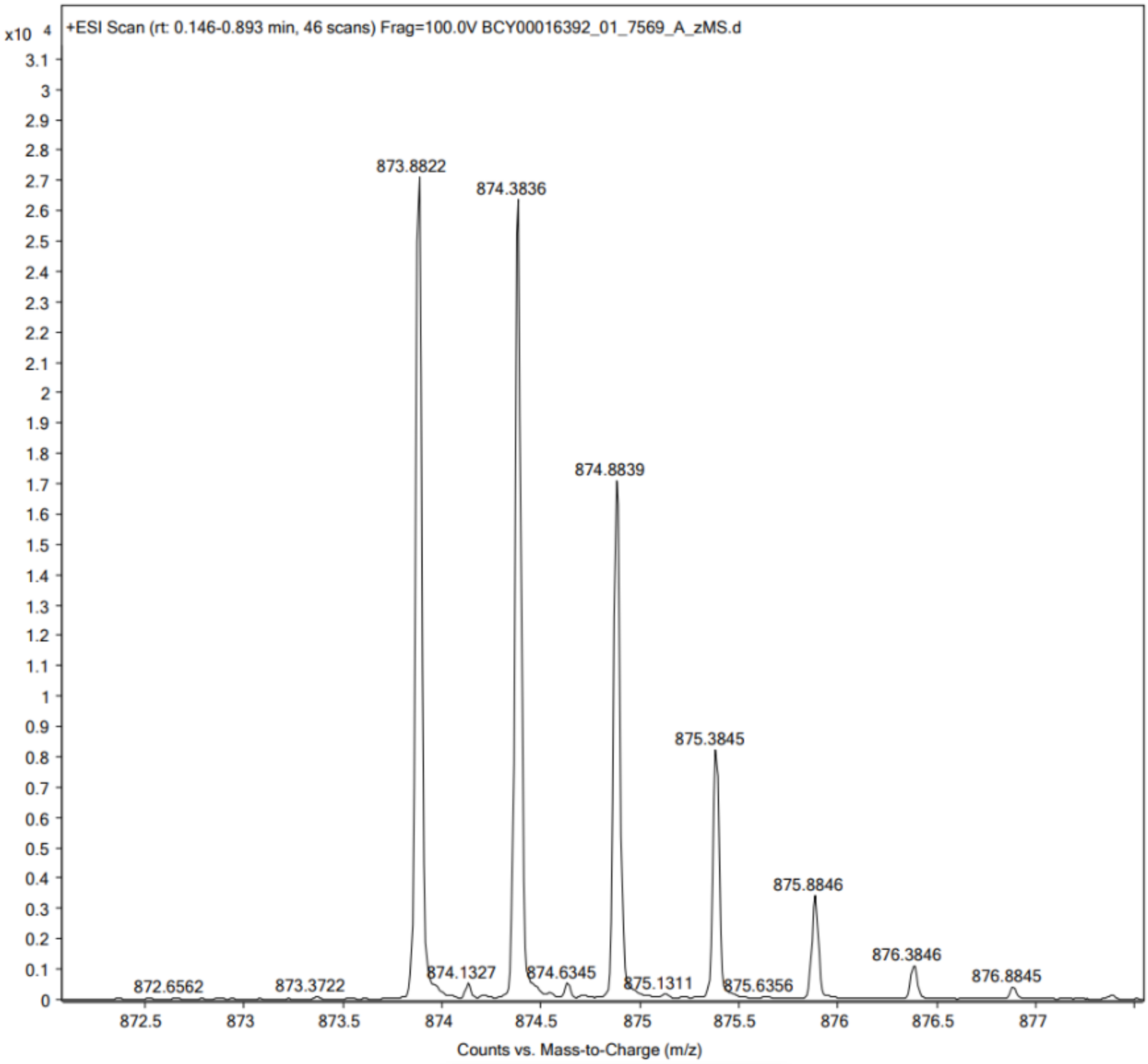
x10 4
+ESI Scan (rt: 0.146-0.893 min, 46 scans) Frag=100.0V BCY00016392_01_7569_A_zMS.d
873.8822
874.3836
874.8839
875.3845
875.8846
876.3846
876.8845
872.6562
873.3722
874.1327
874.6345
875.1311
875.6356
Counts vs. Mass-to-Charge (m/z)

Peptide 60

Data file: D:\Chemstation\1\Data\Peptides_facon\7000-7999
_BCY00016390_01_7567_A_zHPLC 17-49-38.D

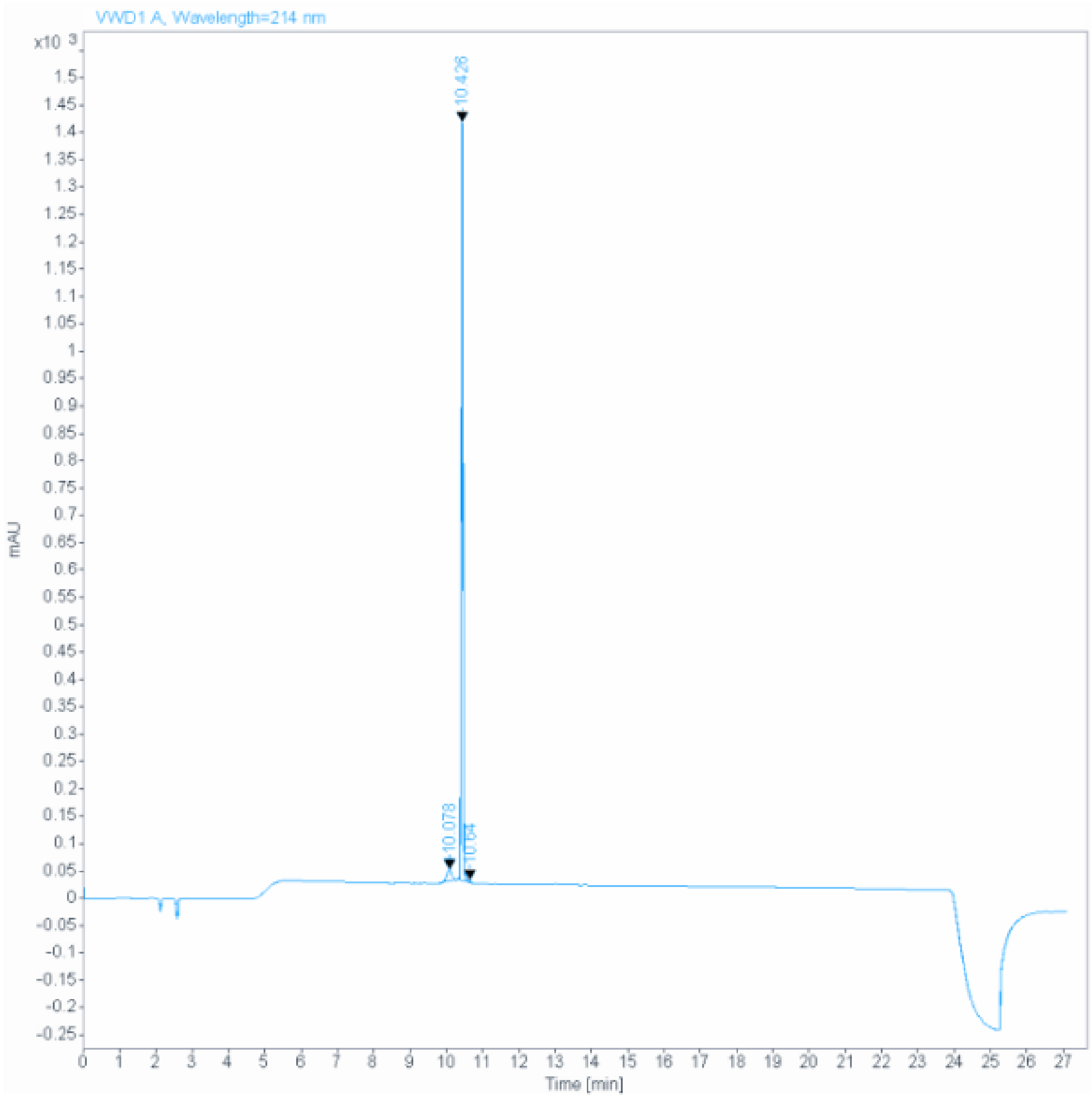

Signal: VWD1 A, Wavelength=214 nm

RT [min]	Type	Width [min]	Area	Height	Area%	Name
10.078	BB	0.1423	228.6930	23.2463	4.3871	
10.426	BV R	0.0558	4966.4780	1386.9453	95.2733	
10.640	VB E	0.0492	17.7032	5.5542	0.3396	
		Sum	5212.8743			

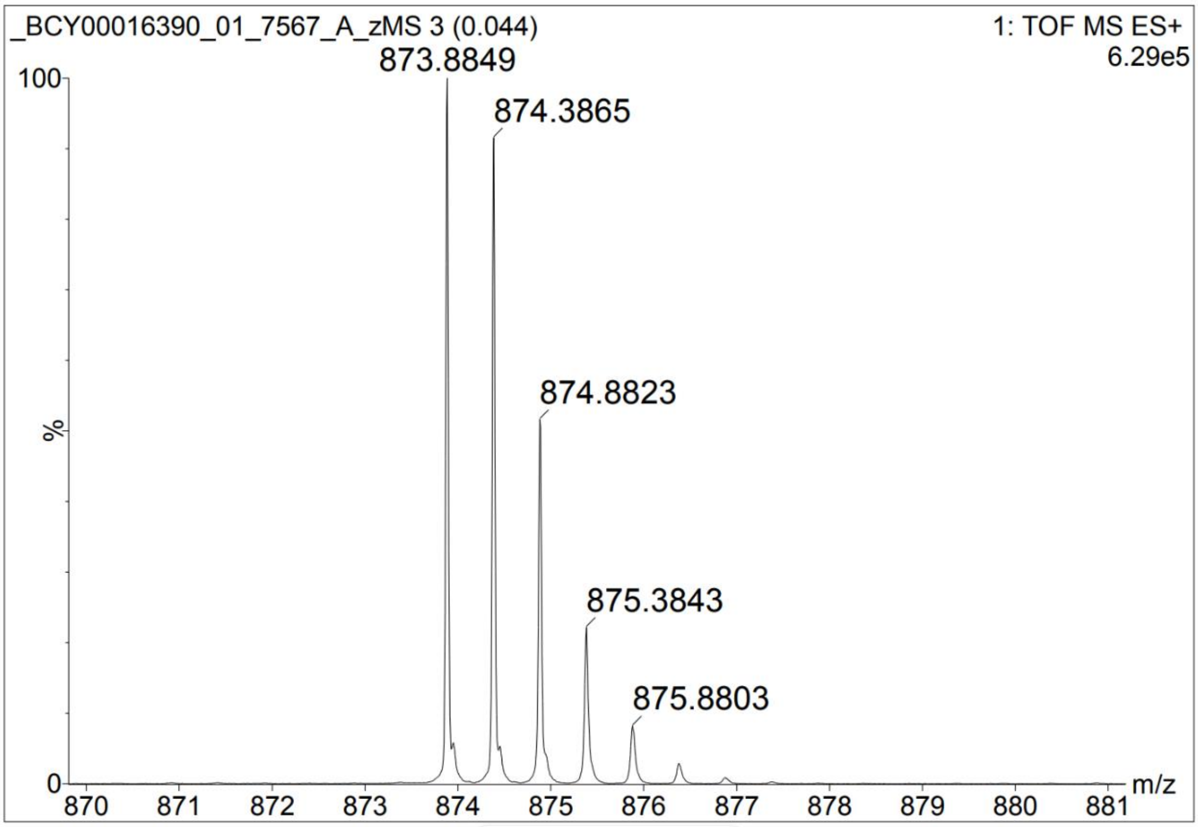

_BCY00016390_01_7567_A_zMS 3 (0.044)
1: TOF MS ES+
6.29e5
873.8849
874.3865
874.8823
875.3843
875.8803
100
%
0
870
871
872
873
874
875
876
877
878
879
880
881
m/z

Peptide 61

Data file: D:\Chemstation\1\Data\Peptides_facon\7000-7999_BCY00016420_01_7572_A_zHPLC 15-26-48.D

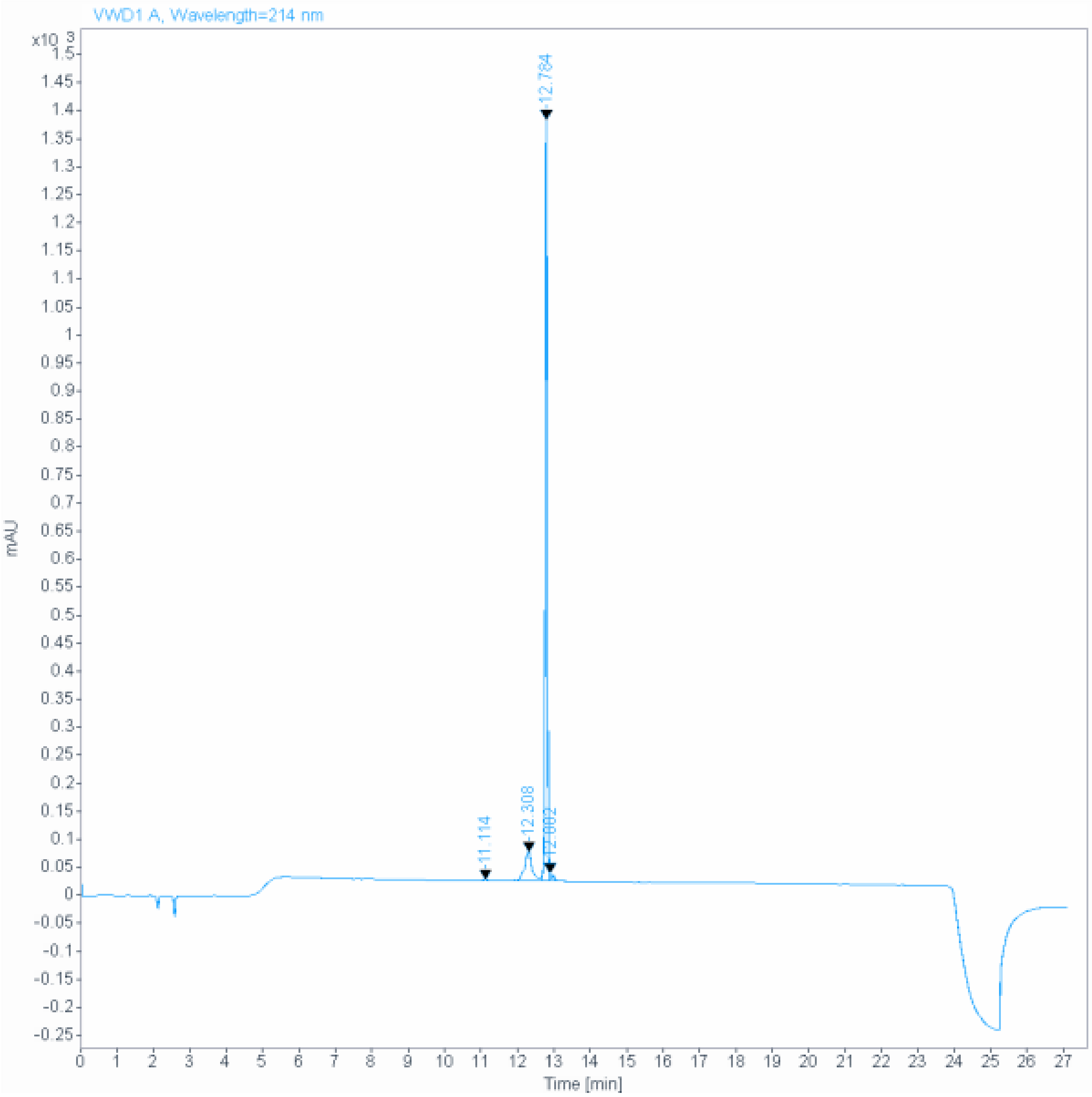

Signal: VWD1 A, Wavelength=214 nm

RT [min]	Type	Width [min]	Area	Height	Area%	Name
11.114	MM	0.0529	4.7925	1.5107	0.0821	
12.308	MF	0.2497	769.0922	51.3441	13.1703	
12.784	MF	0.0612	4988.9927	1358.3730	85.4340	
12.882	FM	0.0772	76.7119	14.2774	1.3137	
		Sum	5839.5893			

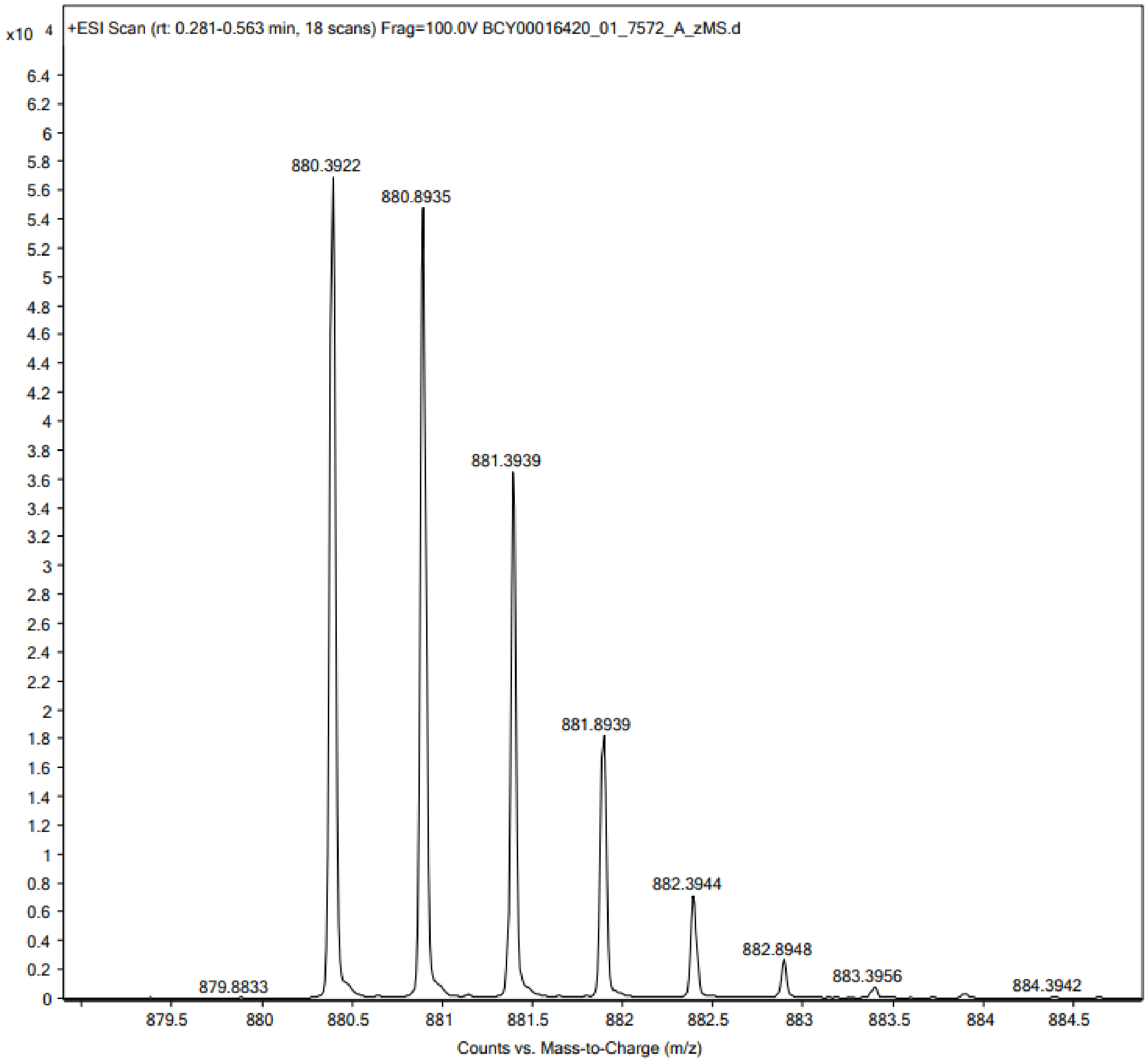

x10 4
+ESI Scan (rt: 0.281-0.563 min, 18 scans) Frag=100.0V BCY00016420_01_7572_A_zMS.d
880.3922
880.8935
881.3939
881.8939
882.3944
882.8948
883.3956
884.3942
879.8833
Counts vs. Mass-to-Charge (m/z)

Peptide 62

Data file: D:\Chemstation\1\Data\Peptides_facon\7000-7999_BCY00016414_01_7599_A_zHPLC 17-10-12.D

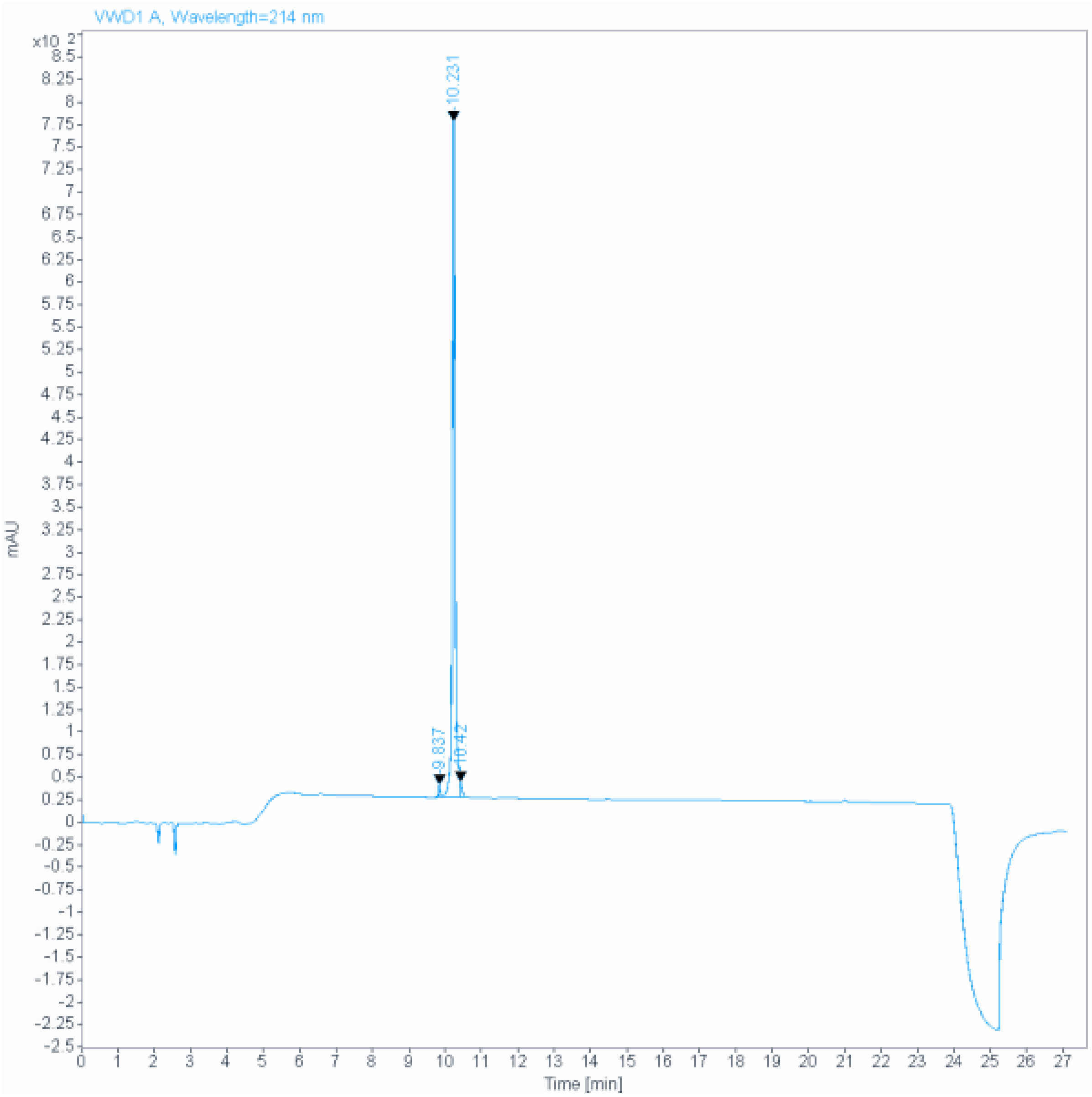

Signal: VWD1 A, Wavelength=214 nm

RT [min]	Type	Width [min]	Area	Height	Area%	Name
9.837	MF	0.0500	43.0186	14.3529	1.0656	
10.231	MF	0.0873	3936.6382	751.3096	97.5109	
10.420	FM	0.0543	57.4695	17.6266	1.4235	
		Sum	4037.1262			

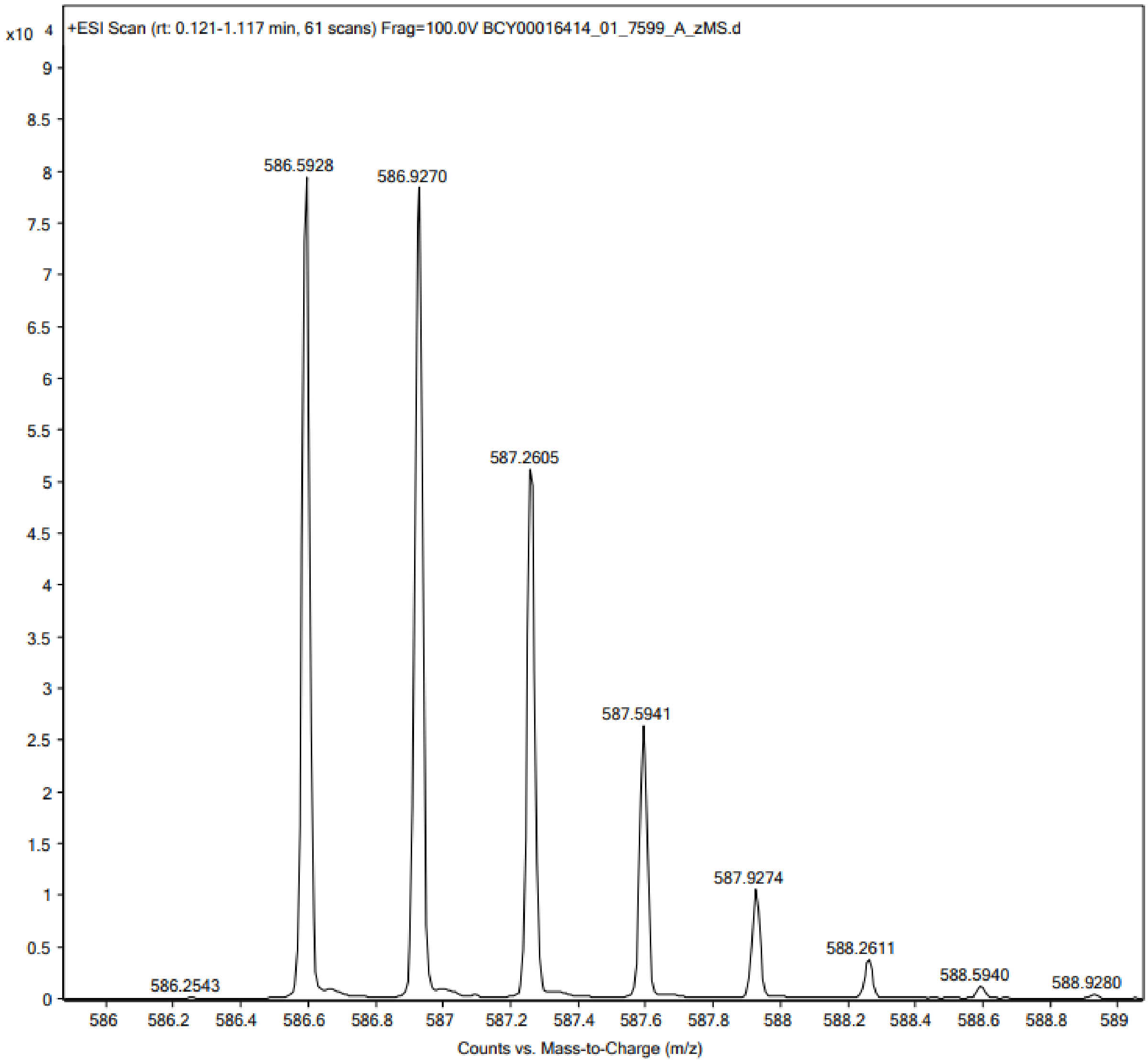
x10 4
+ESI Scan (rt: 0.121-1.117 min, 61 scans) Frag=100.0V BCY00016414_01_7599_A_zMS.d
586.5928
586.9270
587.2605
587.5941
587.9274
588.2611
588.5940
588.9280
586.2543
Counts vs. Mass-to-Charge (m/z)

Peptide 63

Data file: D:\Chemstation\1\Data\Peptides_facon\7000-7999_BCY00016424_01_7590_A_zHPLC 12-15-21.D

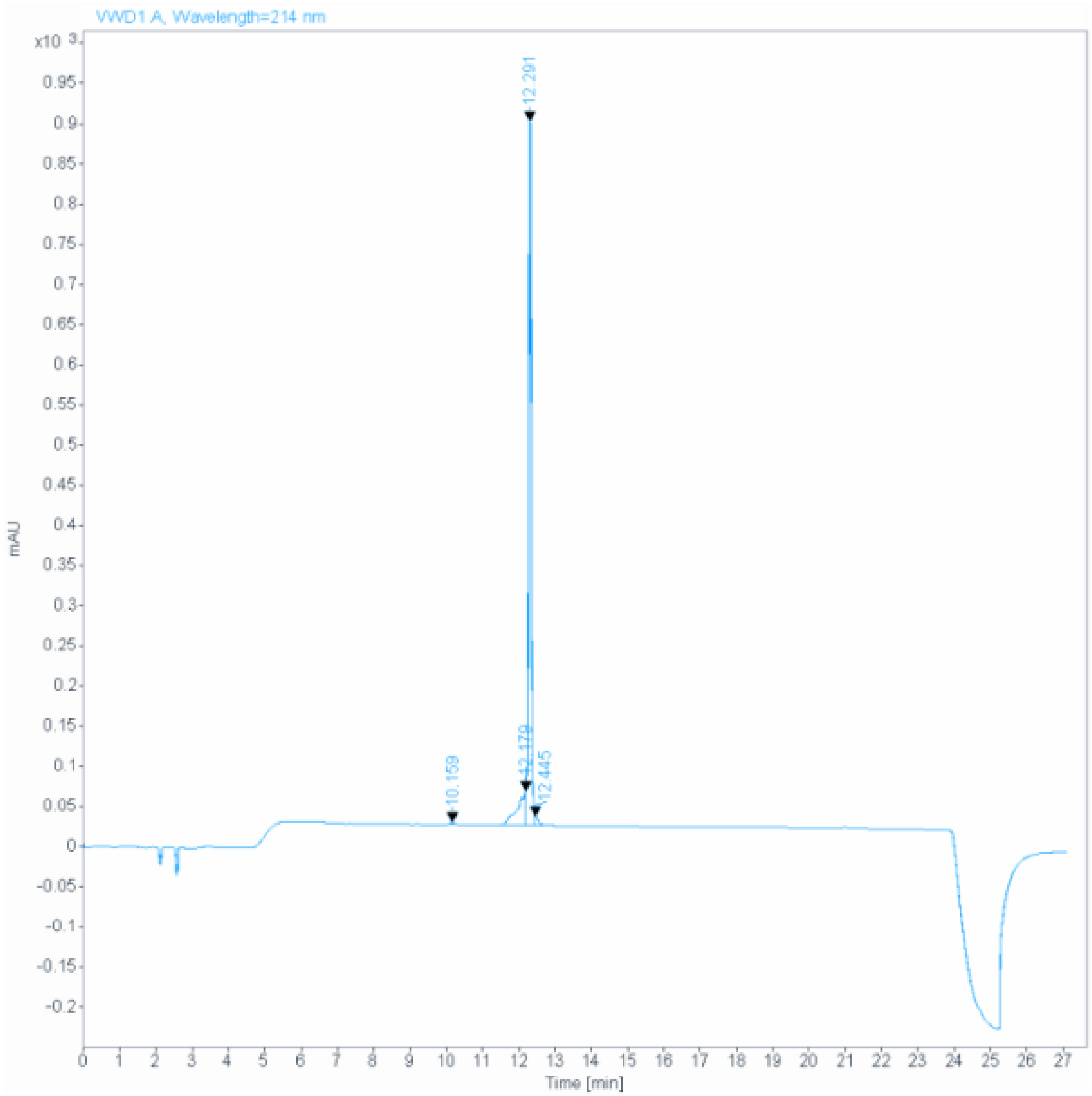

Signal: VWD1 A, Wavelength=214 nm

RT [min]	Type	Width [min]	Area	Height	Area%	Name
10.159	MM	0.1100	18.7838	2.8456	0.3526	
12.179	MF	0.2596	666.1787	42.7704	12.5038	
12.291	MF	0.0866	4556.5186	876.4865	85.5230	
12.445	FM	0.1265	86.3453	11.3775	1.6206	
		Sum	5327.8263			

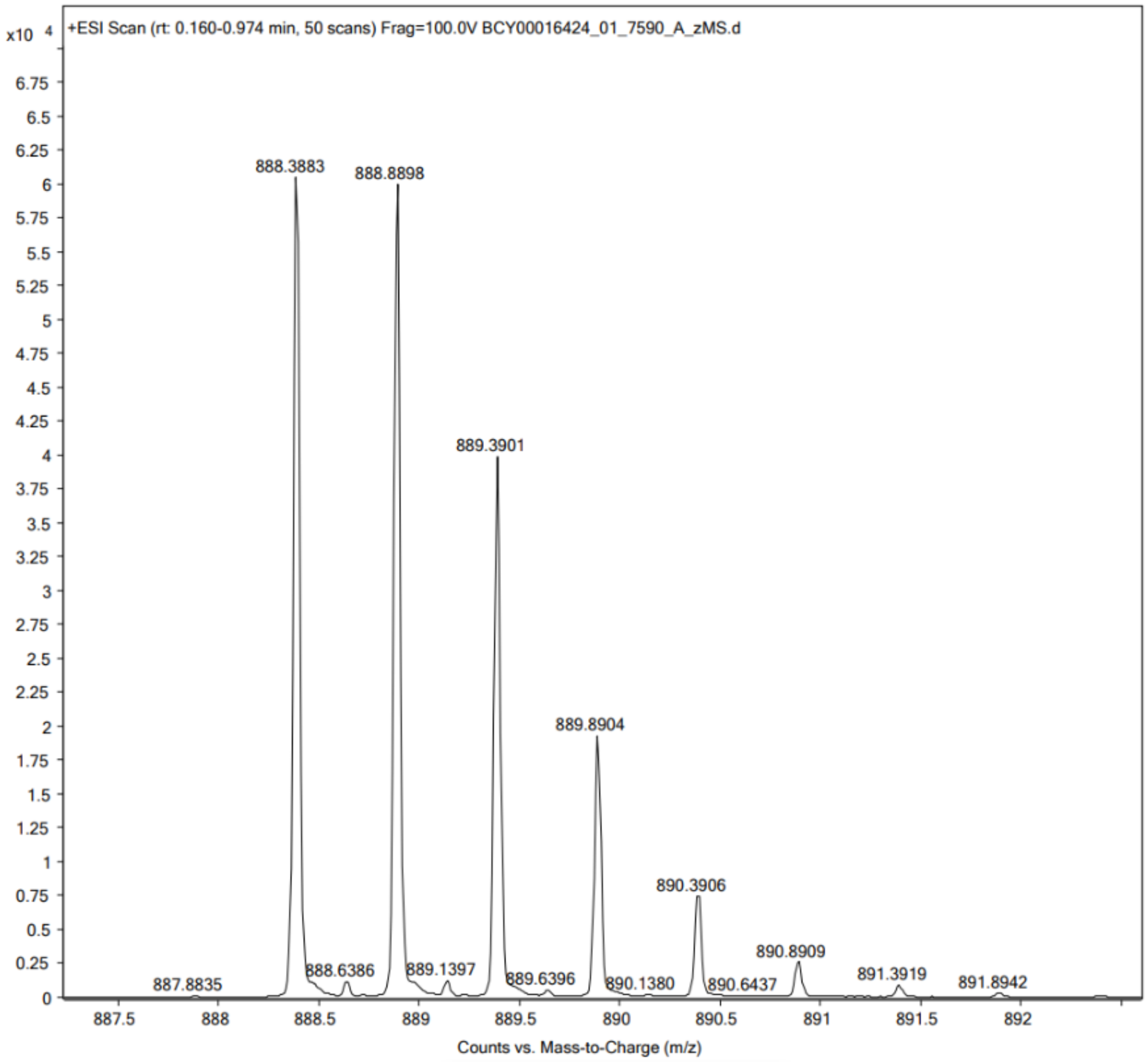

x10 4
+ESI Scan (rt: 0.160-0.974 min, 50 scans) Frag=100.0V BCY00016424_01_7590_A_zMS.d
888.3883
888.8898
889.3901
889.8904
890.3906
890.8909
891.3919
891.8942
887.8835
888.6386
889.1397
889.6396
890.1380
890.6437
Counts vs. Mass-to-Charge (m/z)

Peptide 64

Data file: D:\Chemstation\1\Data\Peptides_facon\7000-7999_BCY00016425_01_7591_A_zHPLC 12-51-13.D

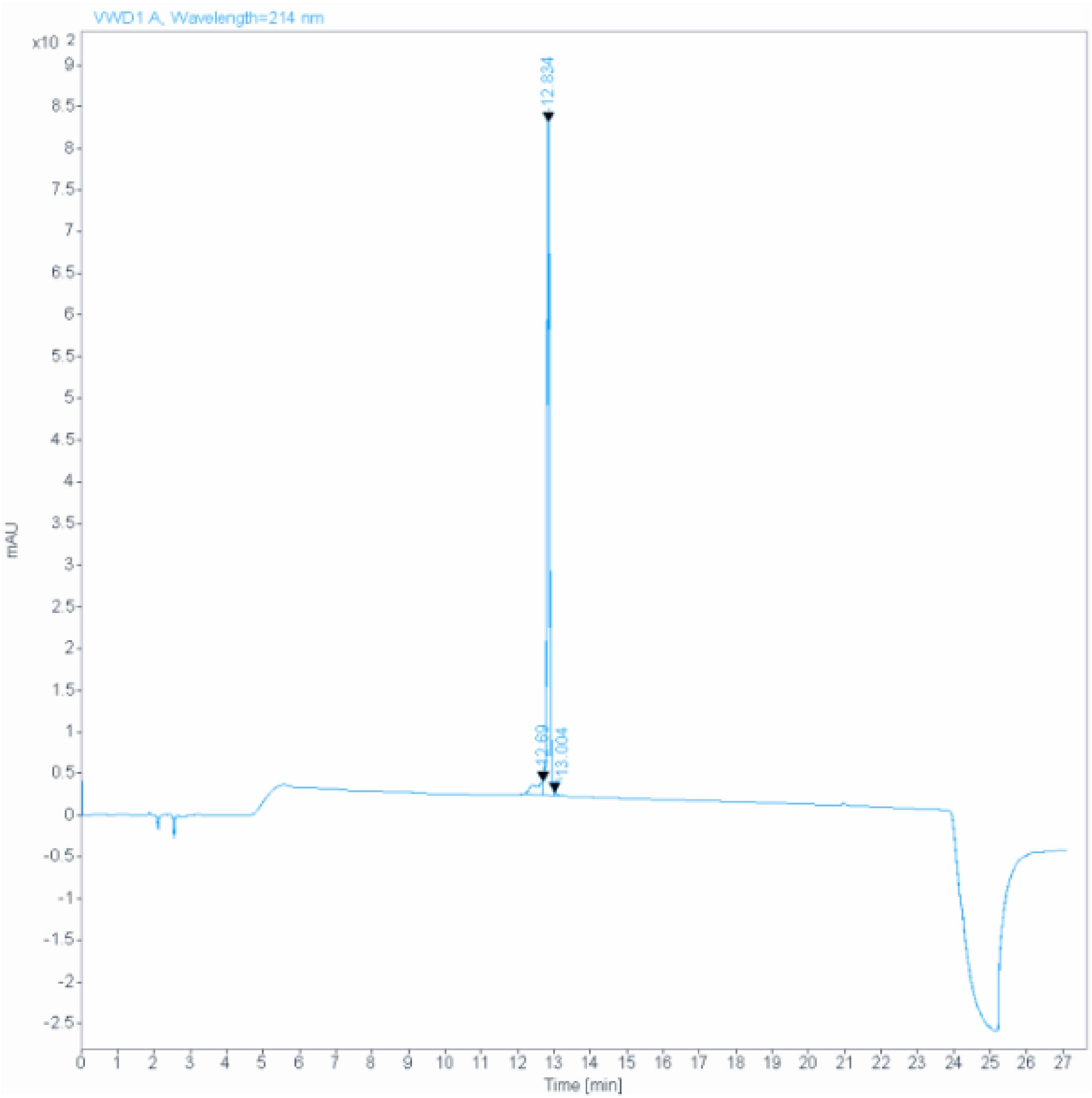

Signal: VWD1 A, Wavelength=214 nm

RT [min]	Type	Width [min]	Area	Height	Area%	Name
12.690	MF	0.3195	313.4438	16.3531	7.0477	
12.834	MF	0.0848	4111.5620	807.7789	92.4476	
13.004	FM	0.0878	22.4476	4.2594	0.5047	
		Sum	4447.4534			

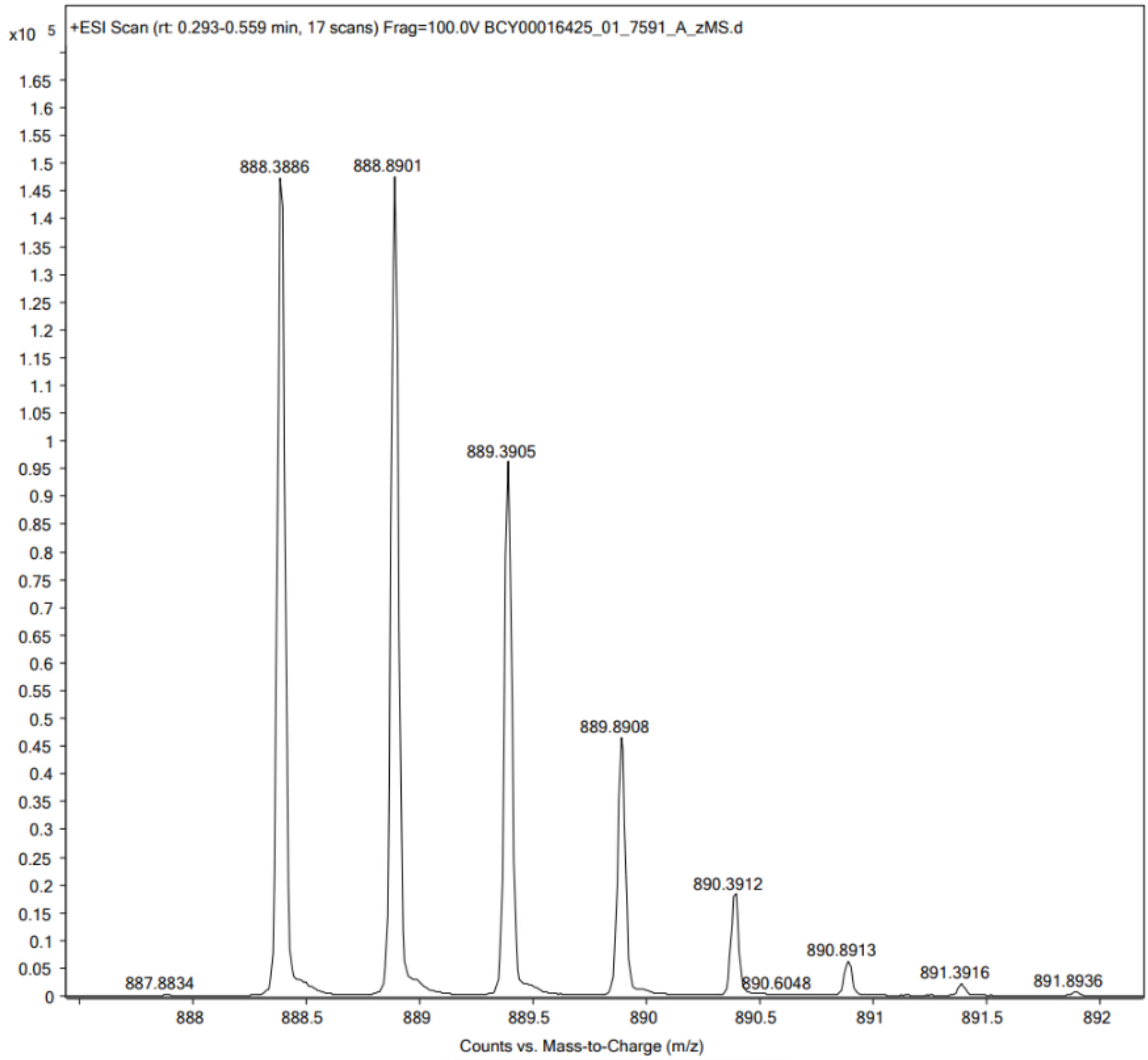
x10 5
+ESI Scan (rt: 0.293-0.559 min, 17 scans) Frag=100.0V BCY00016425_01_7591_A_zMS.d
888.3886
888.8901
889.3905
889.8908
890.3912
890.8913
891.3916
891.8936
887.8834
890.6048
Counts vs. Mass-to-Charge (m/z)

Peptide 65

Data file: D:\Chemstation\1\Data\Peptides_facon\7000-7999 _BCY00016423_01_7585_A_zHPLC 15-52-38.D

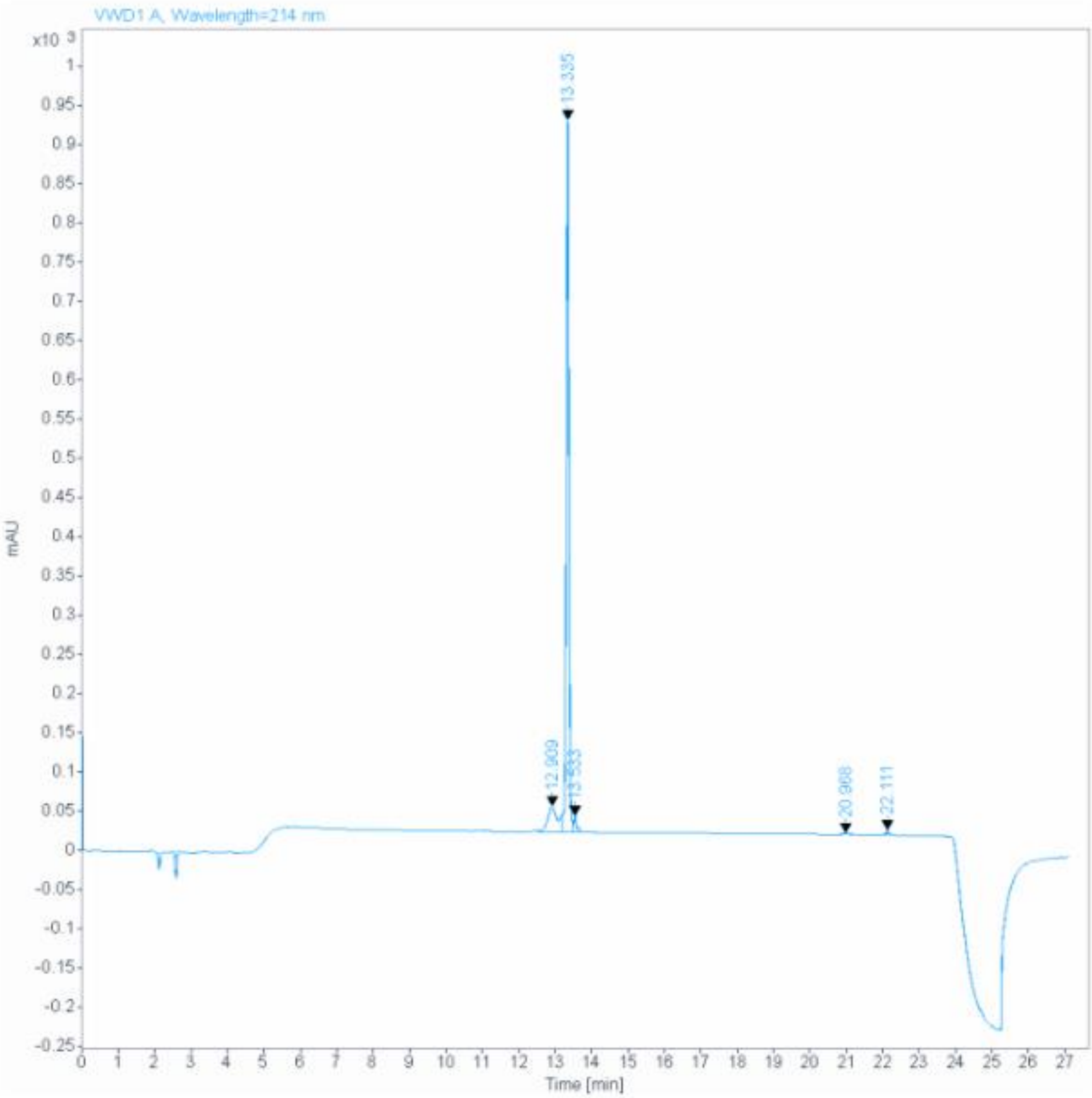

Signal: VWD1 A, Wavelength=214 nm

RT [min]	Type	Width [min]	Area	Height	Area%	Name
12.909	MF	0.3389	679.0752	33.3977	12.3935	
13.335	MF	0.0849	4632.4487	908.9108	84.5449	
13.533	FM	0.0988	127.4004	21.4998	2.3251	
20.968	MM	0.1232	17.8466	2.4138	0.3257	
22.111	MM	0.0615	22.5069	6.0992	0.4108	
		Sum	5479.2778			

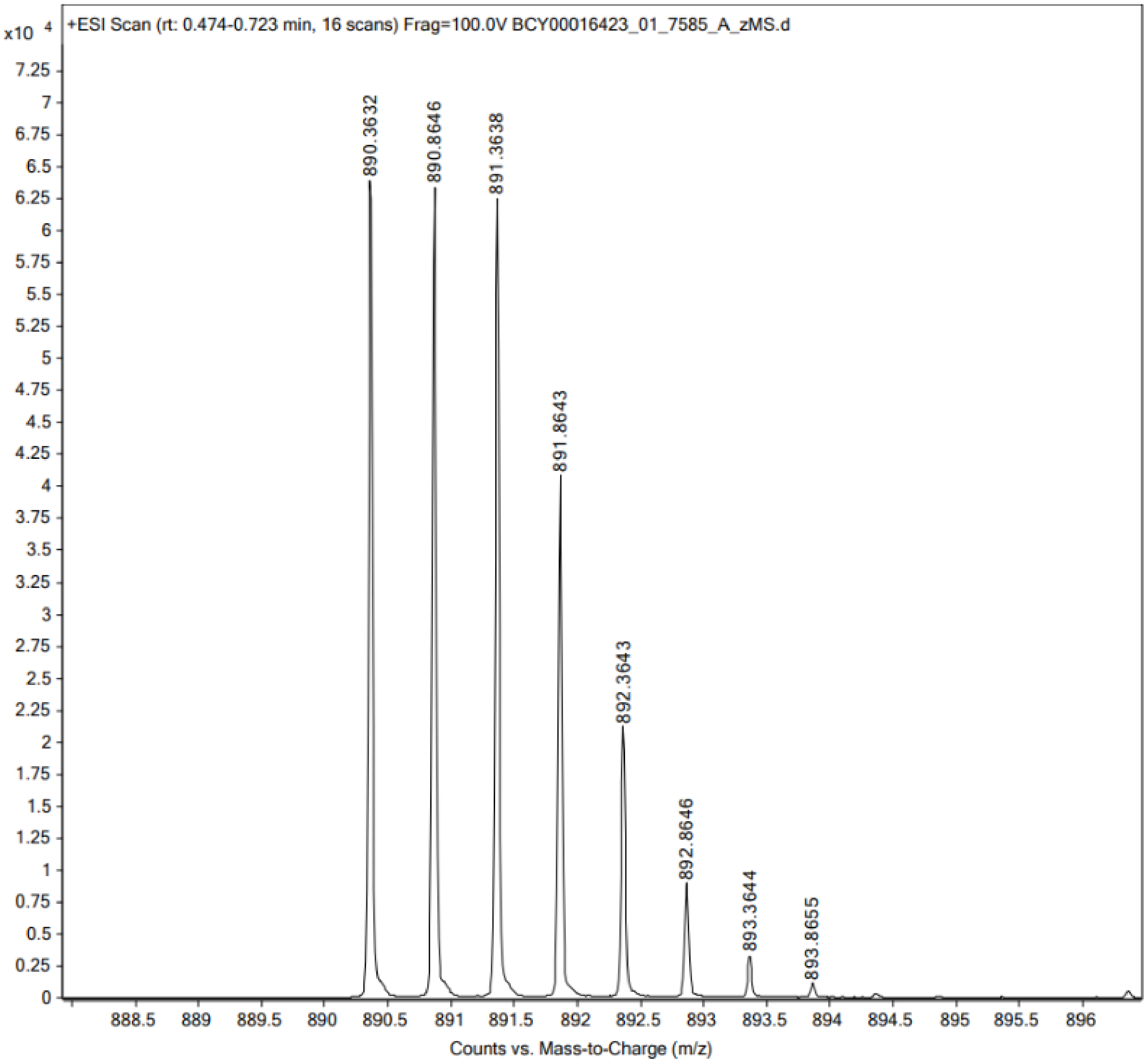
+ESI Scan (rt: 0.474-0.723 min, 16 scans) Frag=100.0V BCY00016423_01_7585_A_zMS.d
x10 4
890.3632
890.8646
891.3638
891.8643
892.3643
892.8646
893.3644
893.8655
Counts vs. Mass-to-Charge (m/z)

Peptide 66

Data file: D:\Chemstation\1\Data\Peptides_facon\7000-7999_BCY00016422_01_7584_A_zHPLC 15-26-42.D

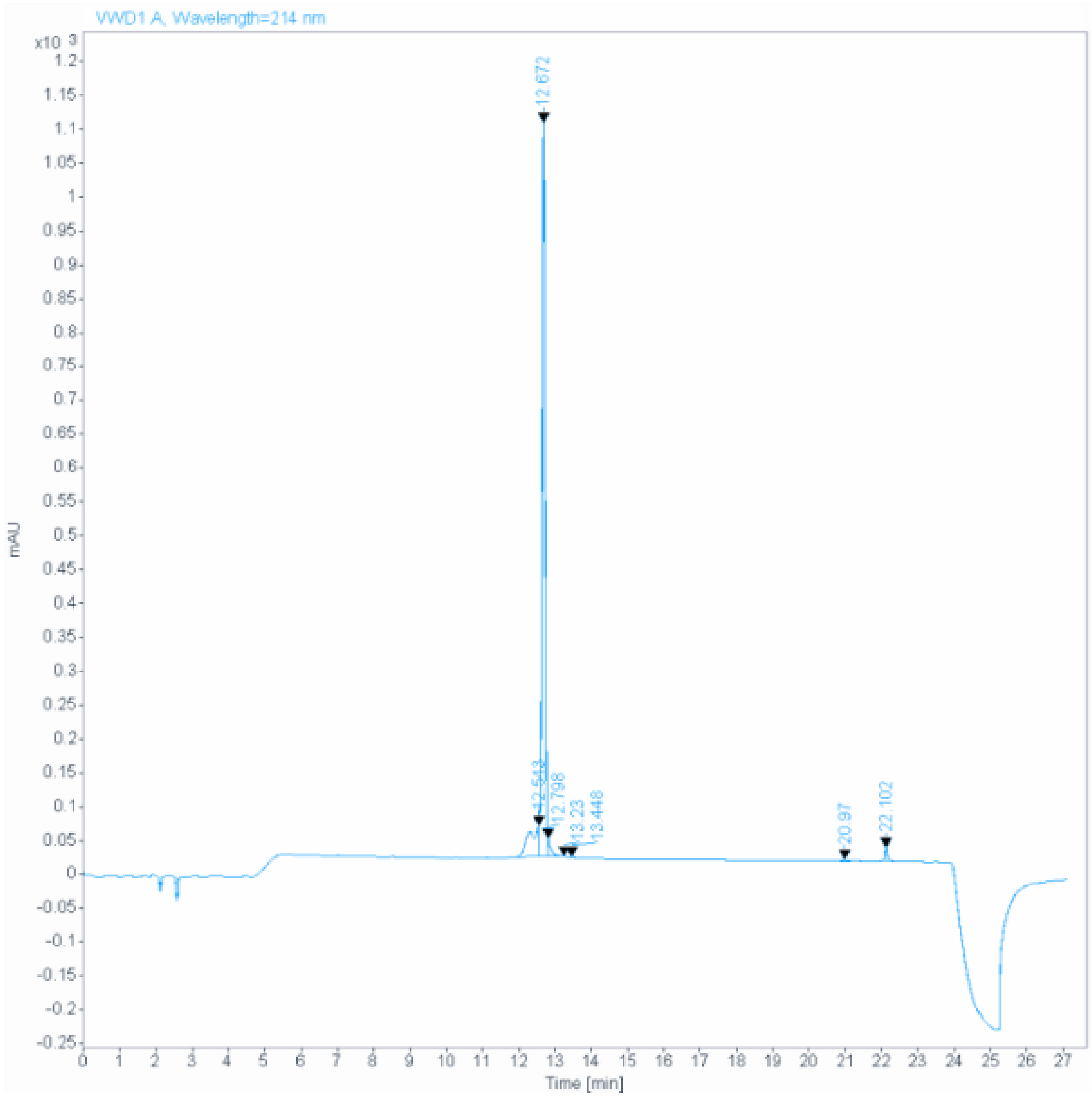

Signal: VWD1 A, Wavelength=214 nm

RT [min]	Type	Width [min]	Area	Height	Area%	Name
12.543	MF	0.3063	858.9743	46.7413	12.1752	
12.672	FM	0.0909	5917.3608	1084.9646	83.8735	
12.798	FM	0.0853	140.6630	27.4843	1.9938	
13.230	MM	0.1219	9.4092	1.2863	0.1334	
13.448	MM	0.0623	6.7239	1.8000	0.0953	
20.970	MM	0.2149	38.4804	2.9842	0.5454	
22.102	MM	0.0690	83.4887	20.1703	1.1834	
		Sum	7055.1002			

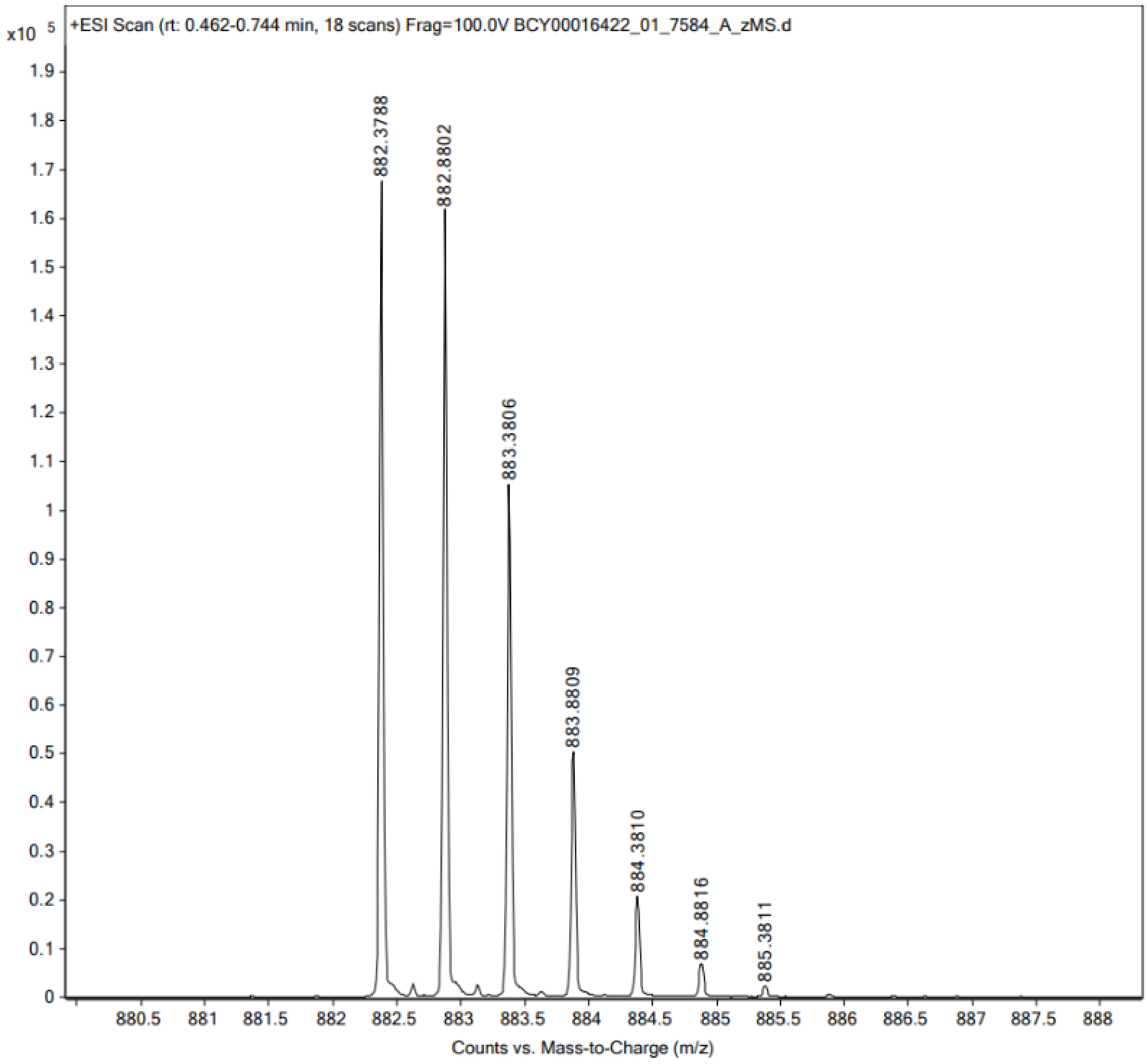

x10 5
+ESI Scan (rt: 0.462-0.744 min, 18 scans) Frag=100.0V BCY00016422_01_7584_A_zMS.d
1.9
1.8
1.7
1.6
1.5
1.4
1.3
1.2
1.1
1
0.9
0.8
0.7
0.6
0.5
0.4
0.3
0.2
0.1
0
882.3788
882.8802
883.3806
883.8809
884.3810
884.8816
885.3811
880.5
881
881.5
882
882.5
883
883.5
884
884.5
885
885.5
886
886.5
887
887.5
888
Counts vs. Mass-to-Charge (m/z)

Peptide 67

Data file: D:\Chemstation\1\Data\Peptides_facon\7000-7999 _BCY00016415_01_7600_A_zHPLC 15-12-56.D

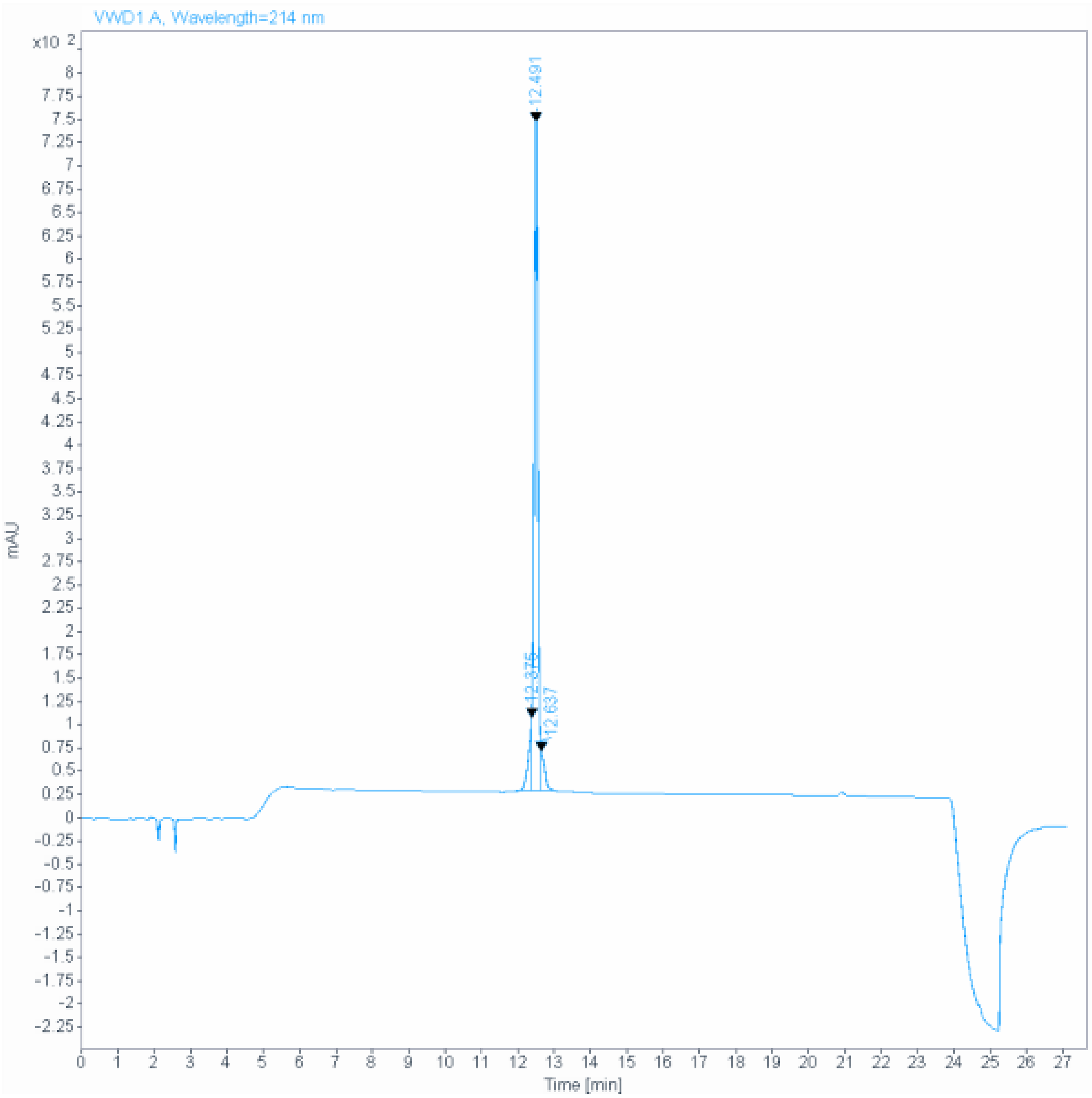

Signal: VWD1 A, Wavelength=214 nm

RT [min]	Type	Width [min]	Area	Height	Area%	Name
12.375	MF	0.1044	490.5663	78.2872	8.5694	
12.491	FM	0.1147	4946.5508	718.8433	86.4086	
12.637	FM	0.1135	287.4871	42.2170	5.0220	
		Sum	5724.6042			

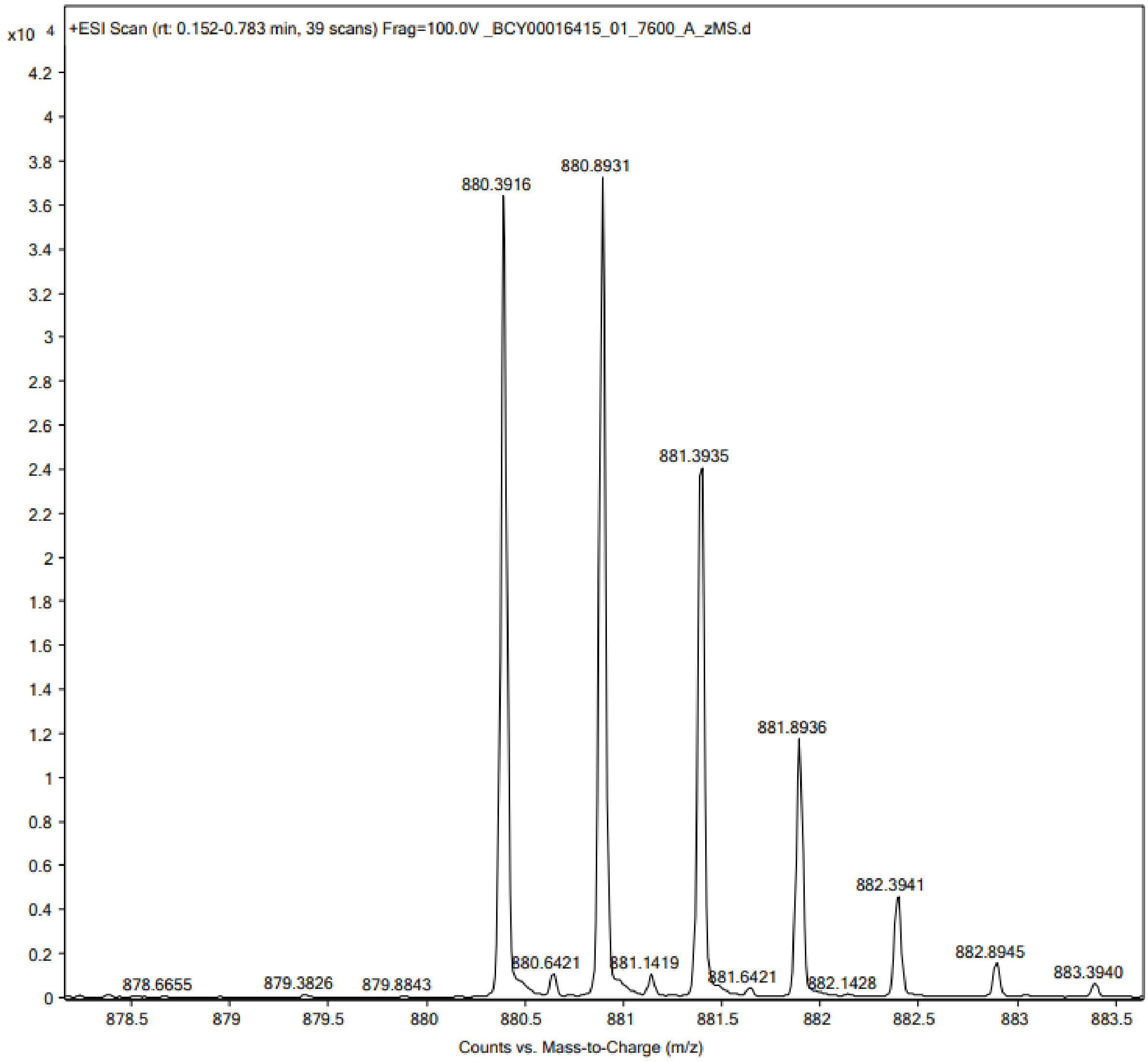

x10 4
+ESI Scan (rt: 0.152-0.783 min, 39 scans) Frag=100.0V _BCY00016415_01_7600_A_zMS.d
880.3916
880.8931
881.3935
881.8936
882.3941
882.8945
883.3940
880.6421
881.1419
881.6421
882.1428
878.6655
879.3826
879.8843
Counts vs. Mass-to-Charge (m/z)

Peptide 68

Data file: D:\Chemstation\1\Data\Peptides_facon\7000-7999_BCY00016416_01_7601_A_zHPLC 15-47-49.D

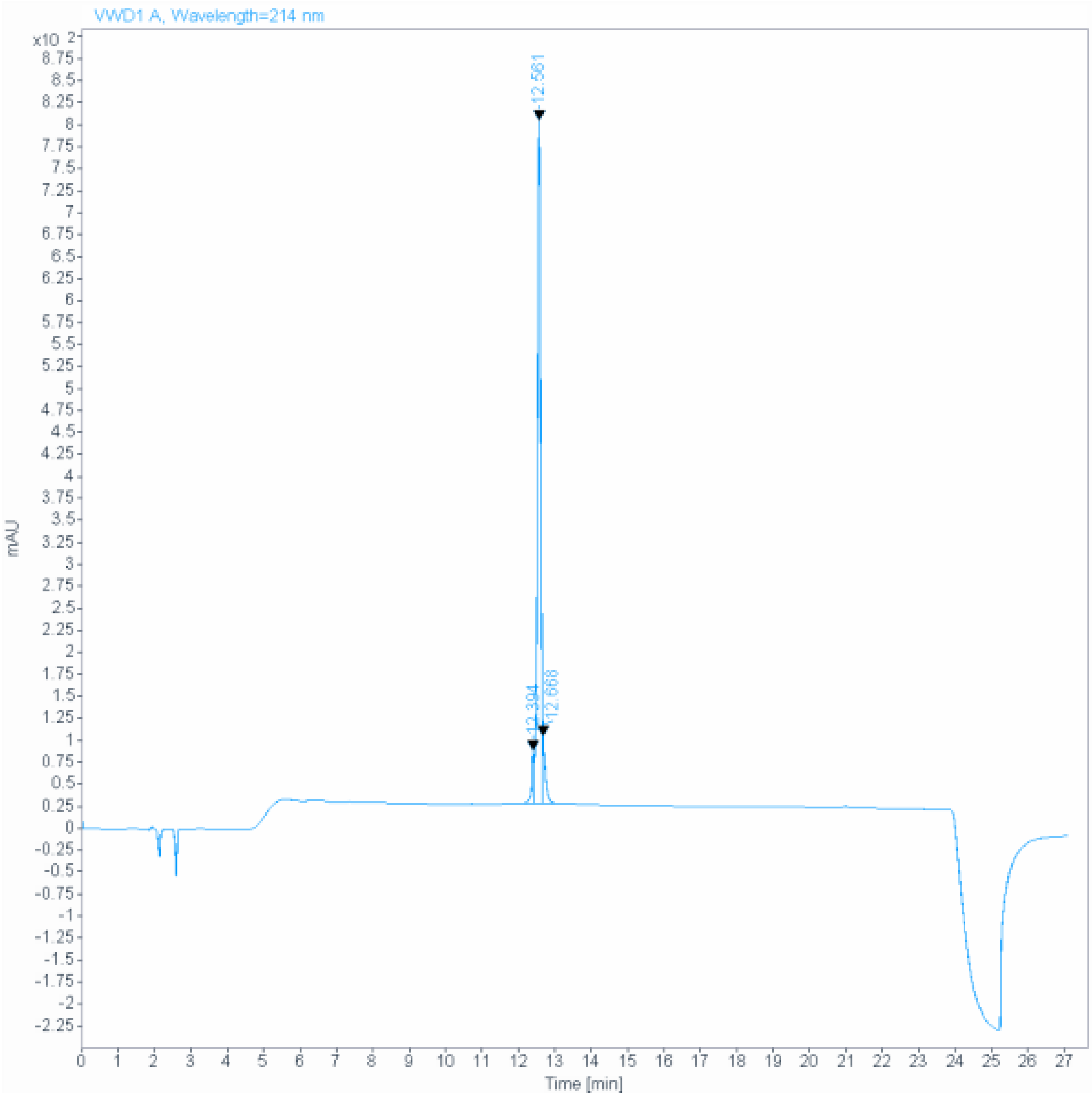

Signal: VWD1 A, Wavelength=214 nm

RT [min]	Type	Width [min]	Area	Height	Area%	Name
12.394	MF	0.0699	255.5601	60.8998	4.5475	
12.561	MF	0.1080	5041.1660	777.6755	89.7044	
12.668	FM	0.0696	323.0262	77.3246	5.7481	
		Sum	5619.7524			

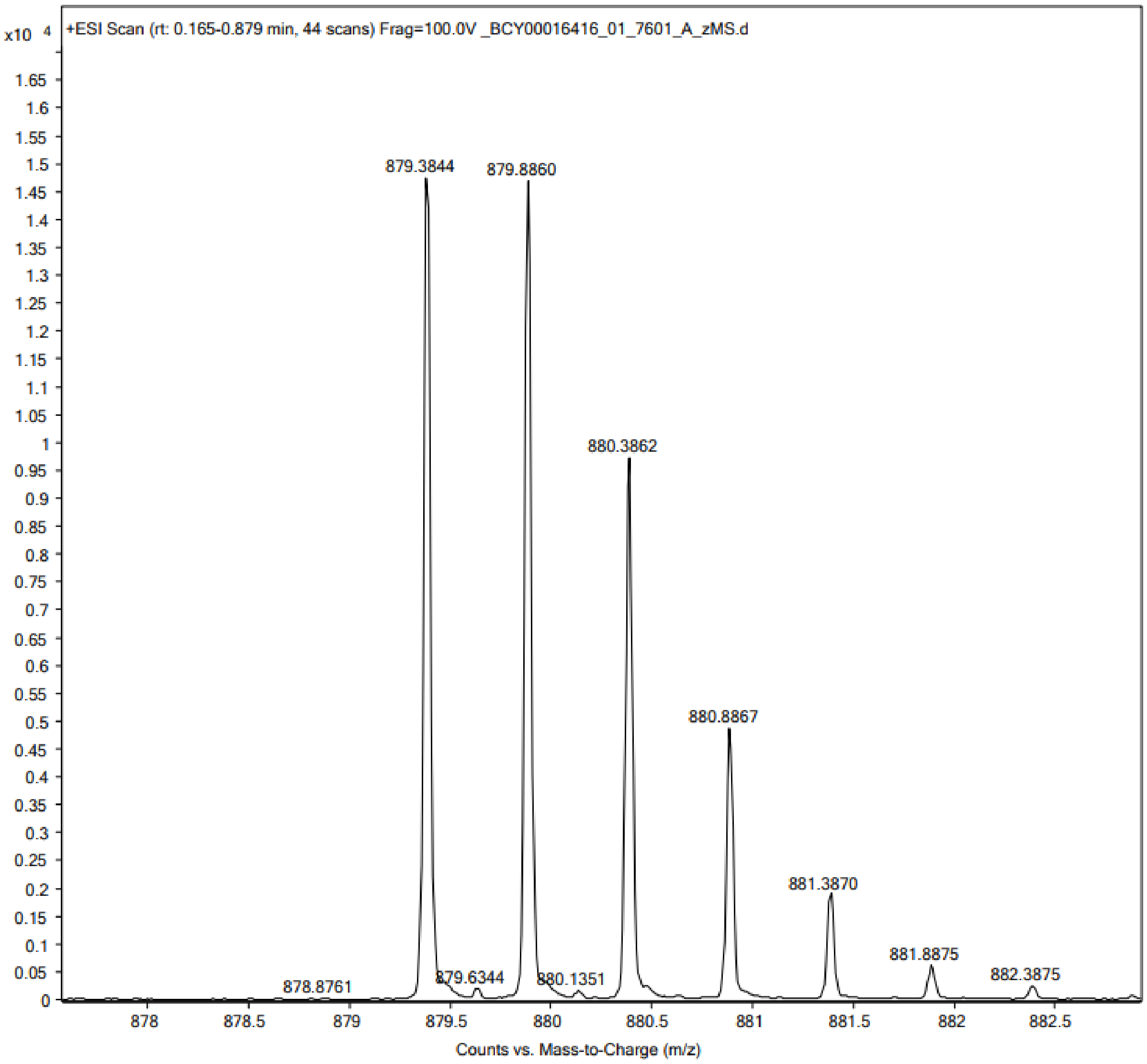
x10 4
+ESI Scan (rt: 0.165-0.879 min, 44 scans) Frag=100.0V _BCY00016416_01_7601_A_zMS.d
879.3844
879.8860
880.3862
880.8867
881.3870
881.8875
882.3875
878.8761
879.6344
880.1351
Counts vs. Mass-to-Charge (m/z)

Peptide 69

Sample name:	(101-08-00) D-Ala7 (TATA) QC
Instrument:	1260_2
Injection date:	12/12/2019 3:28:14 PM
Acq. method:	0595B_AB_POROSHELL120_15.5MIN.M

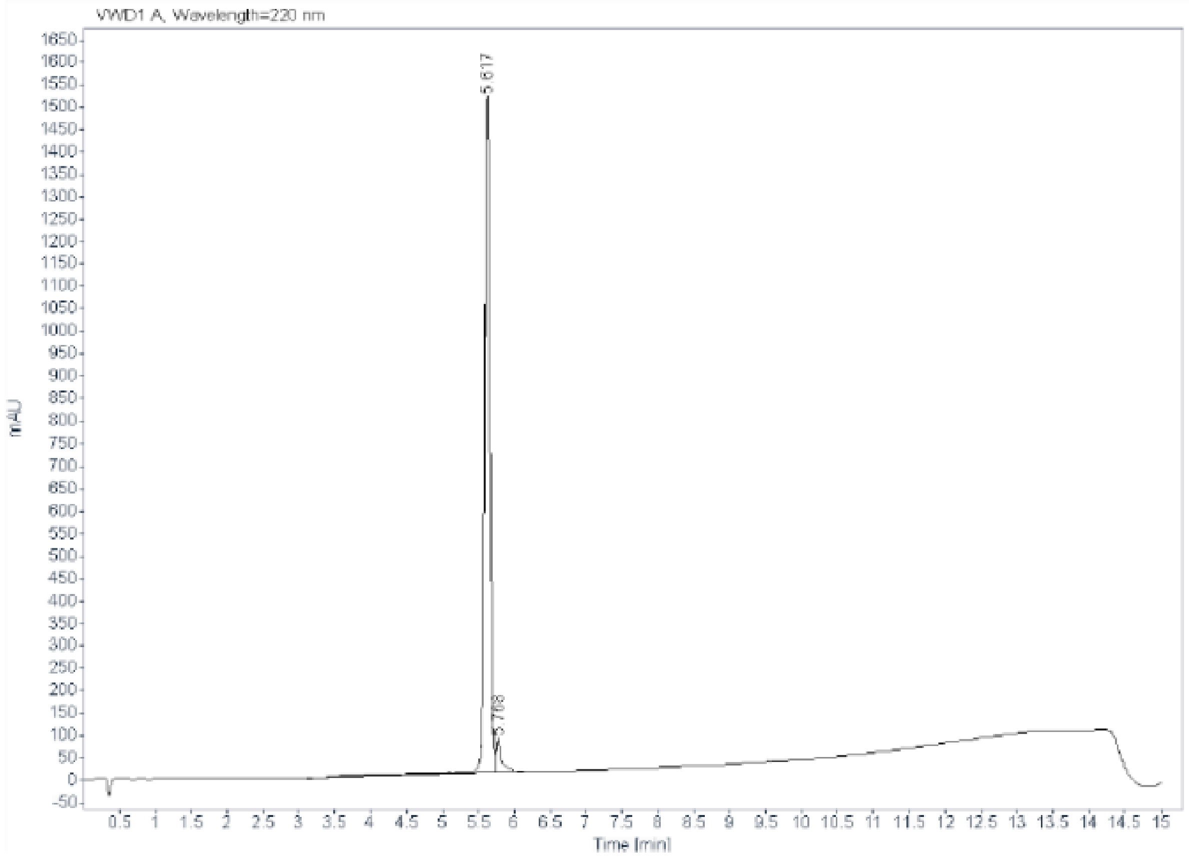

Signal: VWD1 A, Wavelength=220 nm

RT [min]	Type	Width [min]	Area	Height	Area%
5.617	VV R	0.0816	8202.7715	1505.3533	95.1574
5.768	VB	0.0800	417.4467	73.7483	4.8426
		Sum	8620.2182		

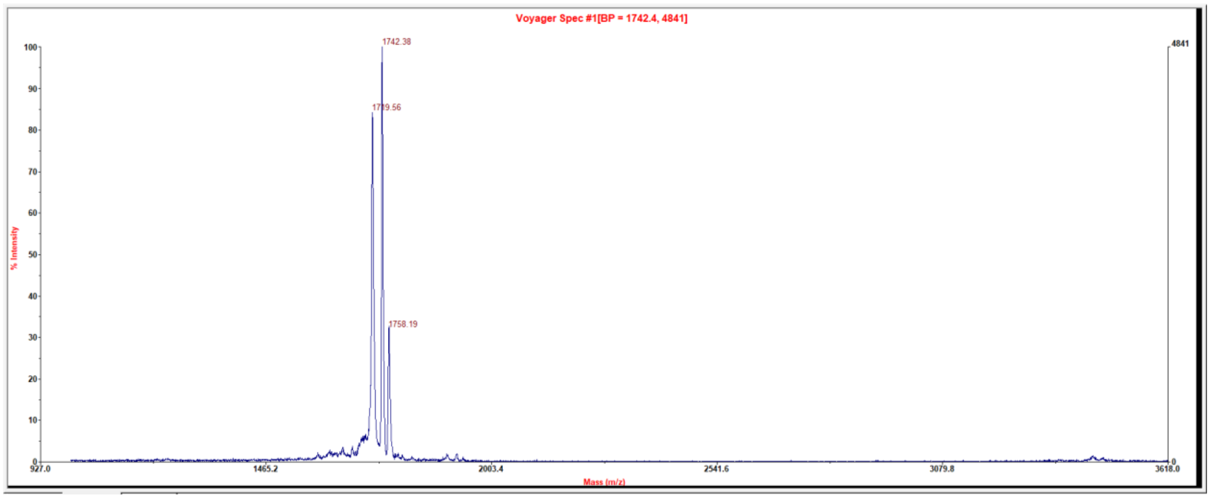

Peptide 70

Sample name:	A-(101-08-00) HArg4
Instrument:	1260_1
Injection date:	5/21/2021 1:13:27 PM
Acq. method:	0595B_AB_KxC18_QC _20min.M

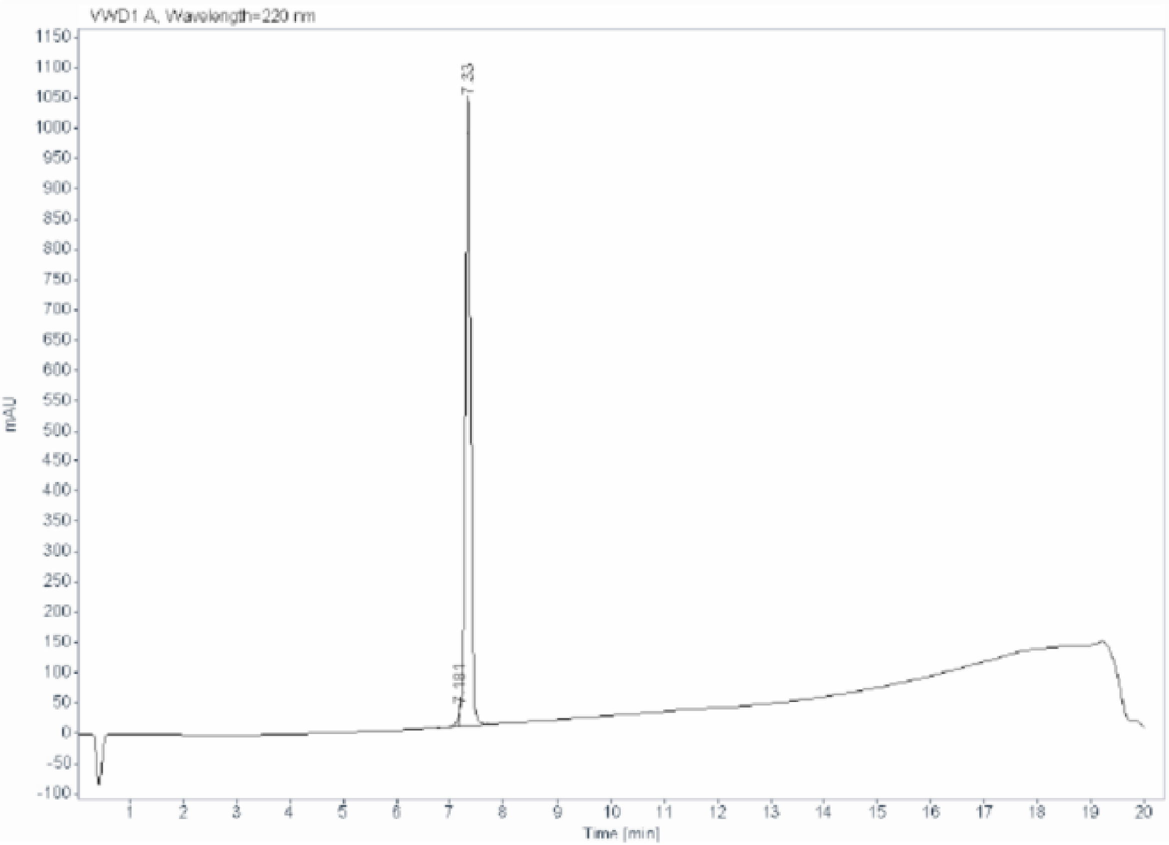

Signal: VWD1 A, Wavelength=220 nm

RT [min]	Type	Width [min]	Area	Height	Area%
7.181	MF	0.0577	105.6076	30.5107	1.4226
7.330	FM	0.1173	7317.7363	1039.9047	98.5774
		Sum	7423.3439		

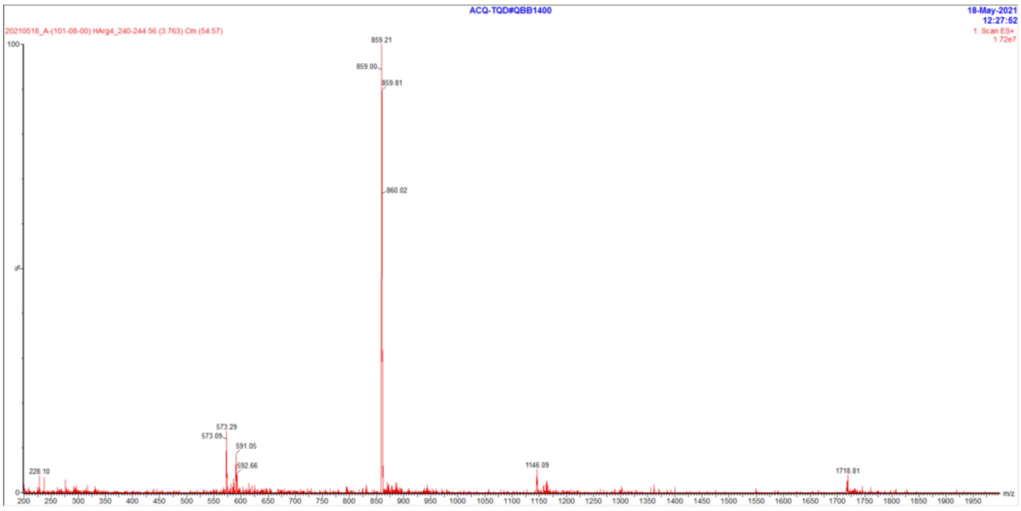

Peptide 71

**Sample name:**	(101-08-00)-A HArg4
**Instrument:**	1260_1
**Injection date:**	5/21/2021 10:00:45 AM
**Acq. method:**	0595B_AB_KxC18_QC _20min.M

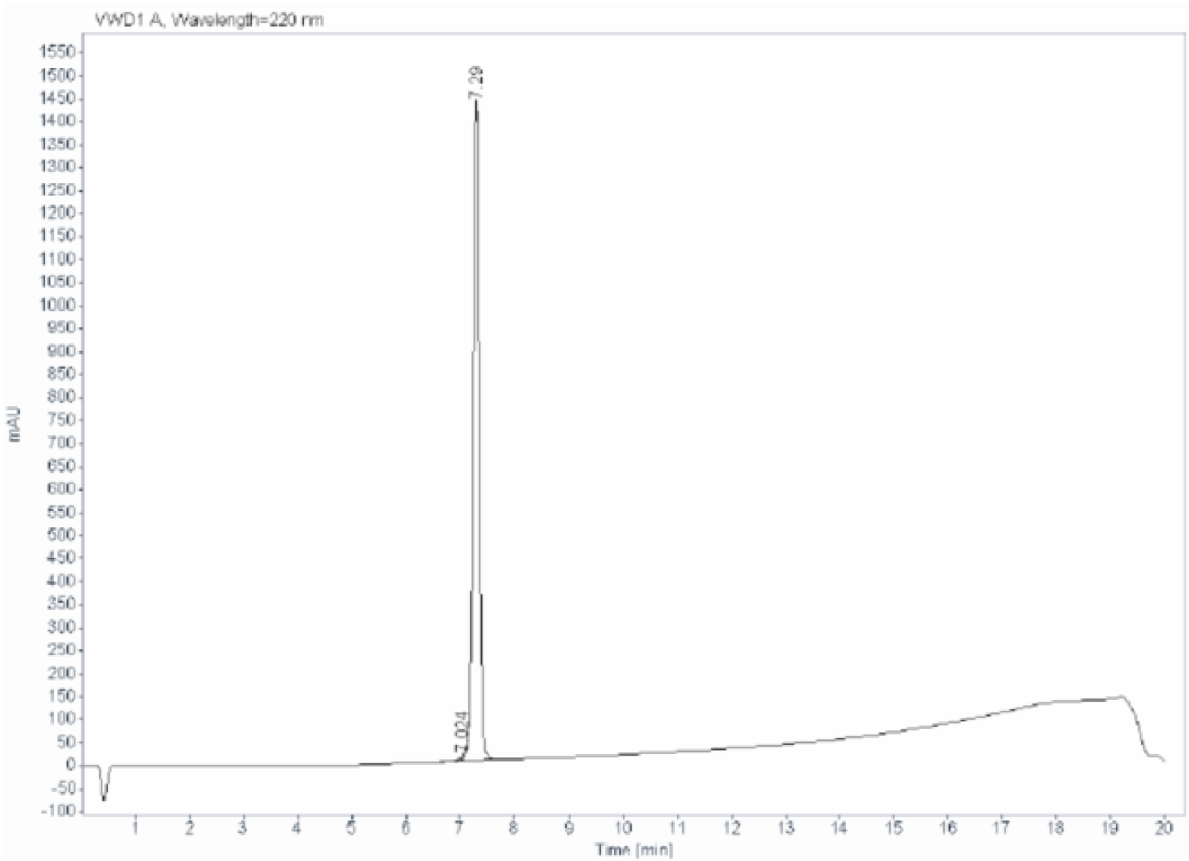

Signal: VWD1 A, Wavelength=220 nm

RT [min]	Type	Width [min]	Area	Height	Area%
7.024	BV E	0.1226	76.7479	8.5584	0.6741
7.290	VB R	0.1223	11309.2490	1430.0098	99.3259
		Sum	11385.9969		

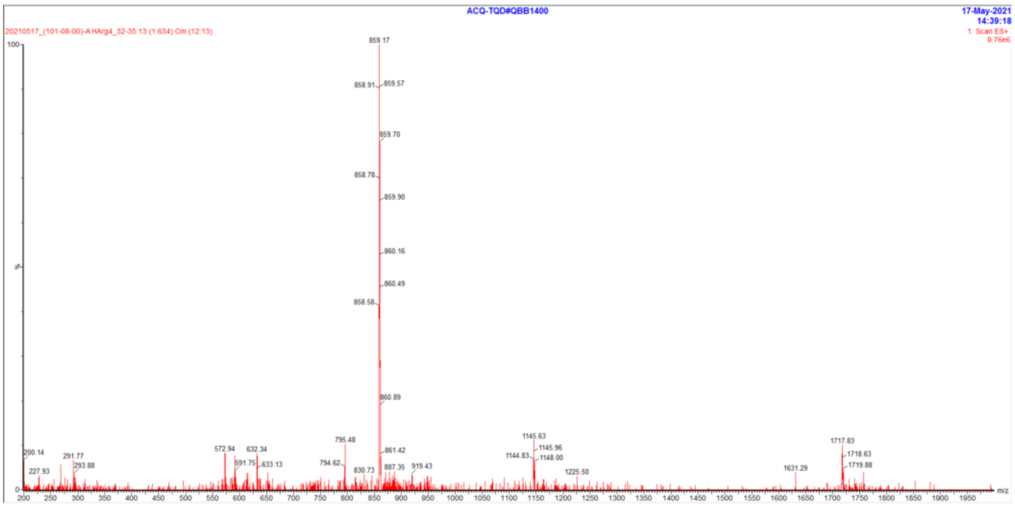

Peptide 72

Sample name: (101-08-00) HArg4
Instrument: 1260_1

Injection date: 5/21/2021 11:05:35 AM
Acq. method: 0595B_AB_KxC18_QC_20min.M

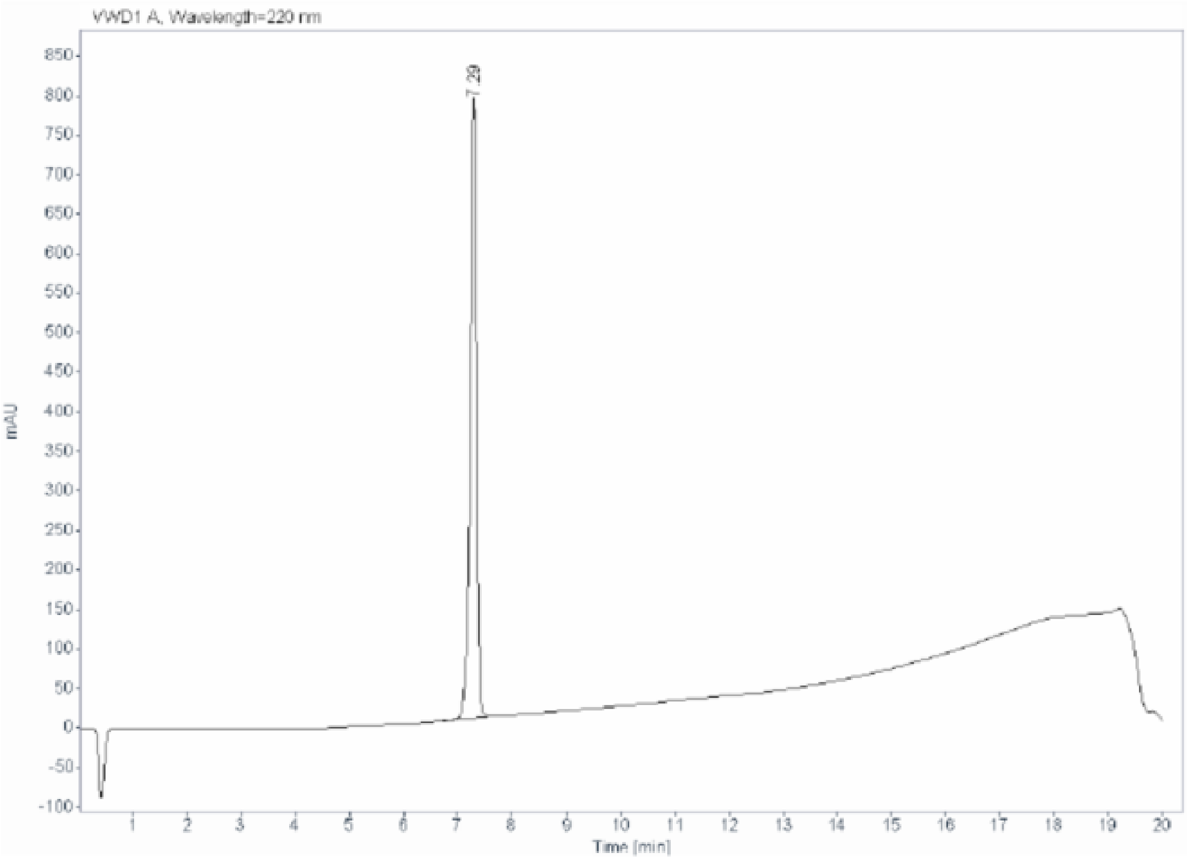

Signal: VWD1 A, Wavelength=220 nm

RT [min]	Type	Width [min]	Area	Height	Area%
7.290	BB	0.1211	6378.9600	782.3913	100.0000
		Sum	6378.9600		

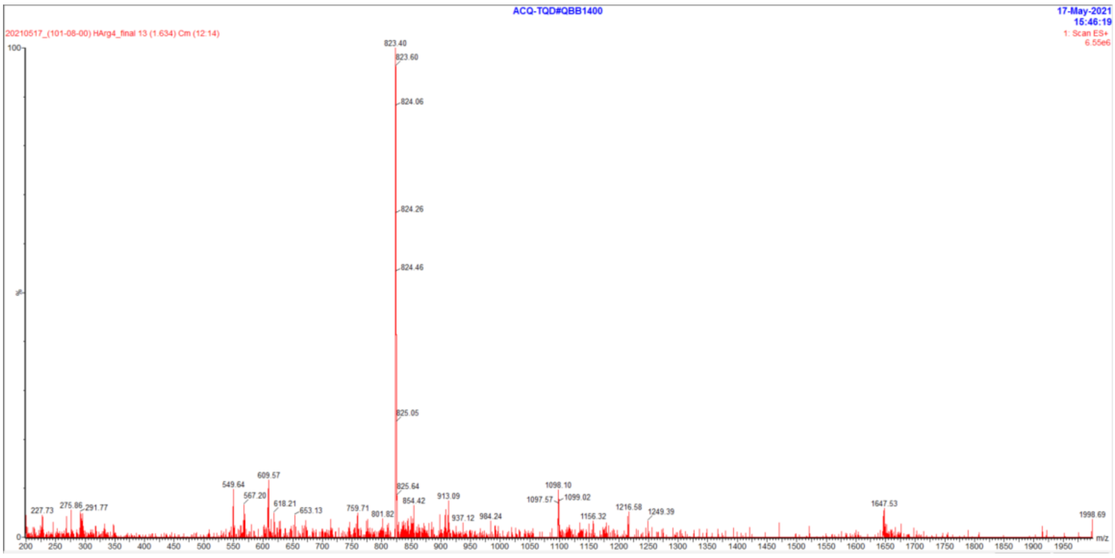

Peptide 73

Sample name:	Ac-A-(101-08-00)(TATA)-A HArg4
Instrument:	1260_2
Injection date:	2/23/2021 1:57:49 PM
Acq. method:	0595B_AB_KxC18_QC<br>_20min.M

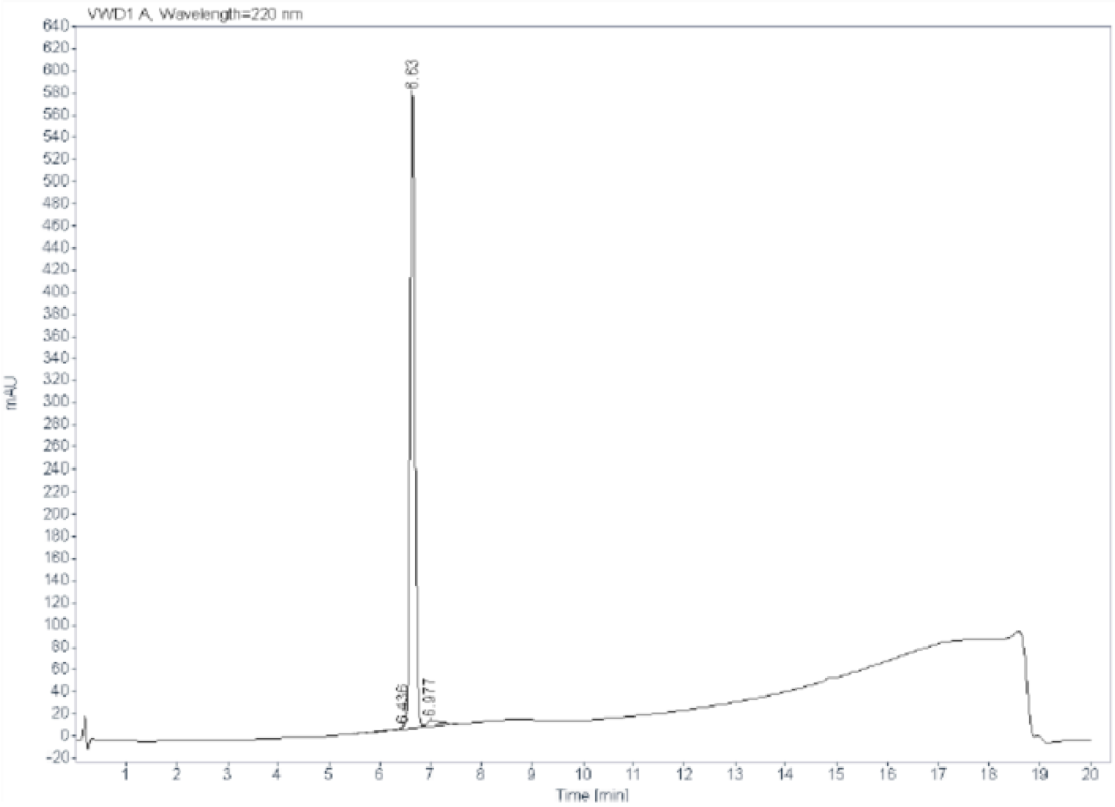

Signal: VWD1 A, Wavelength=220 nm

RT [min]	Type	Width [min]	Area	Height	Area%
6.436	MF	0.3005	33.3237	1.8482	0.8637
6.630	FM	0.1079	3719.9207	574.5839	96.4149
6.977	FM	0.3036	104.9992	5.7642	2.7214
		Sum	3858.2436		

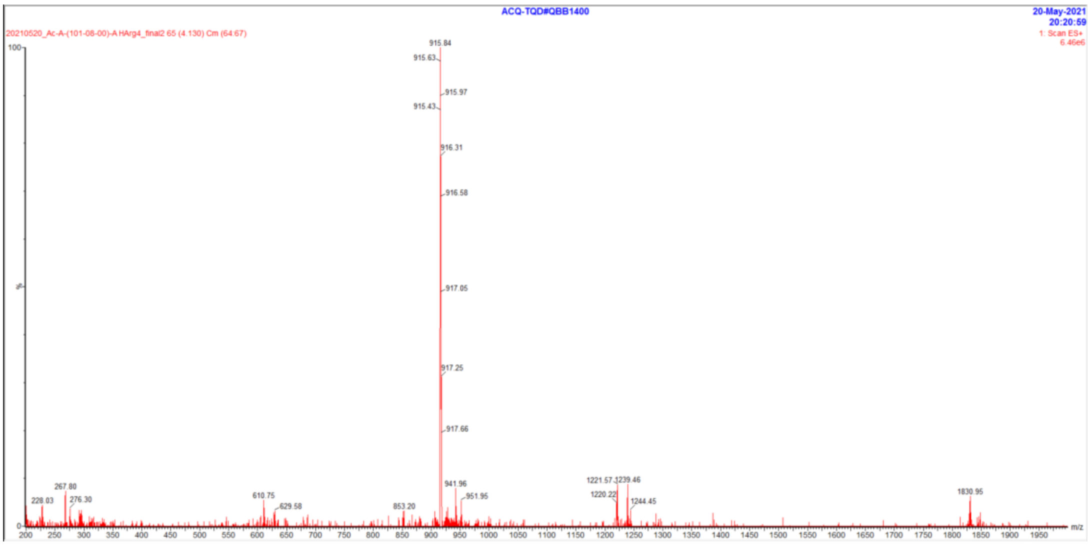

Peptide 74

Sample name:	Ac-(101-08-00)-A HArg4
Instrument:	1260_1
Injection date:	5/20/2021 10:39:30 AM
Acq. method:	0595B_AB_KxC18_QC_20min.M

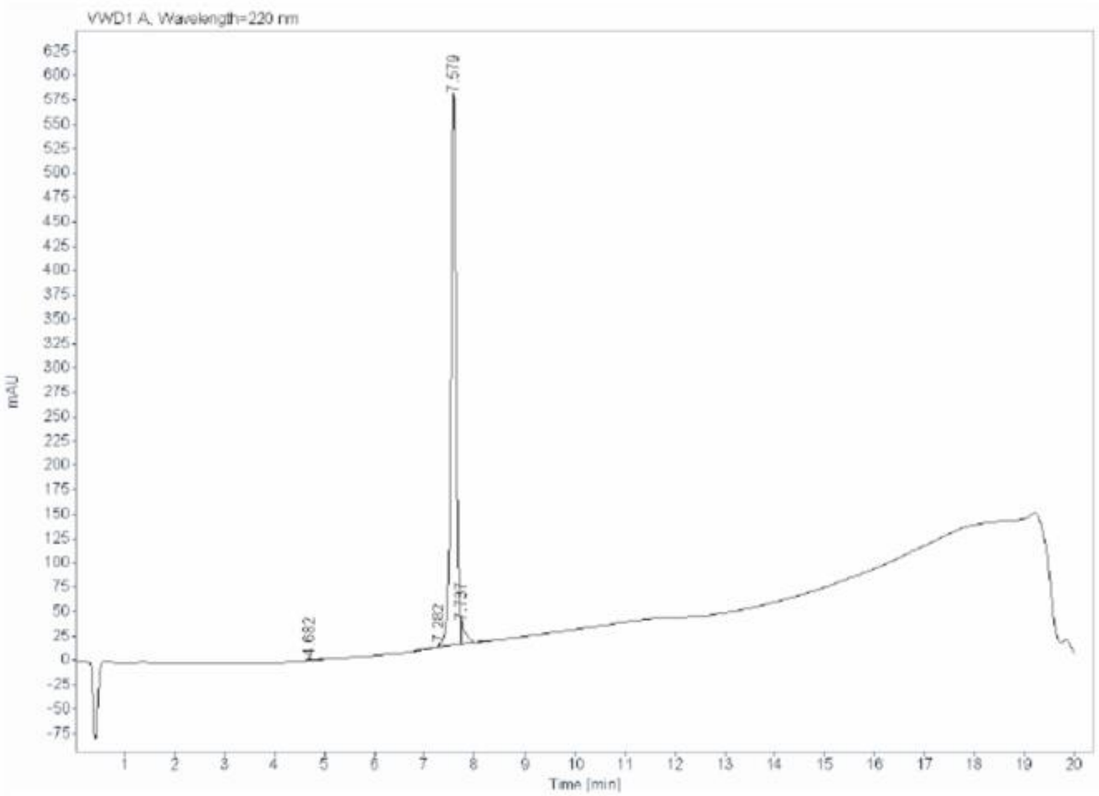

Signal: VWD1 A, Wavelength=220 nm

RT [min]	Type	Width [min]	Area	Height	Area%
4.682	BB	0.1267	12.5235	1.5108	0.2884
7.282	MF	0.1087	13.9476	2.1379	0.3212
7.579	FM	0.1236	4195.7515	565.9112	96.6260
7.737	FM	0.0961	120.0347	20.8225	2.7643
		Sum	4342.2573		

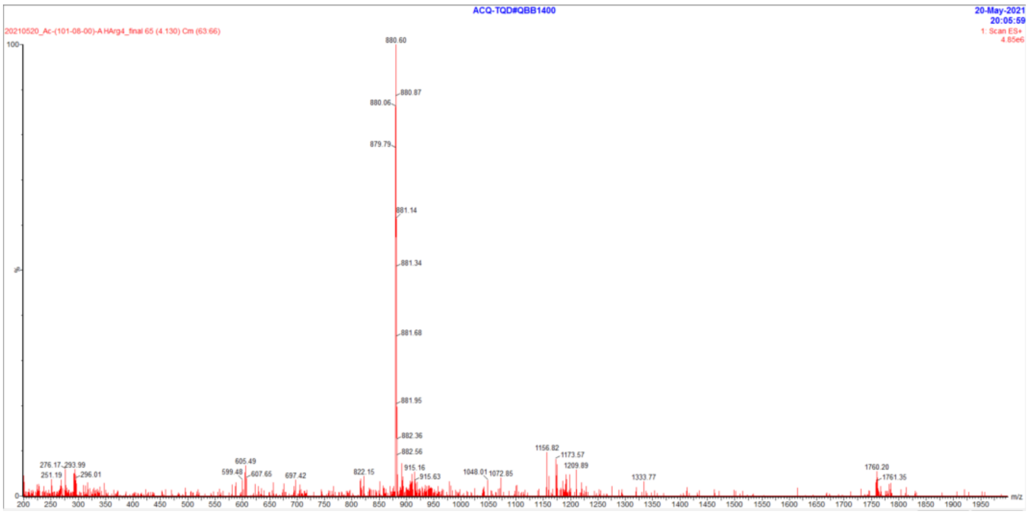

Peptide 75

Sample name: Ac-A-(101-08-00)(TATA)-A HArg4 6ClTrp
Instrument: 1260_2

Injection date: 2/23/2021 3:26:25 PM
Acq. method: 0595B_AB_KxC18_QC _20min.M

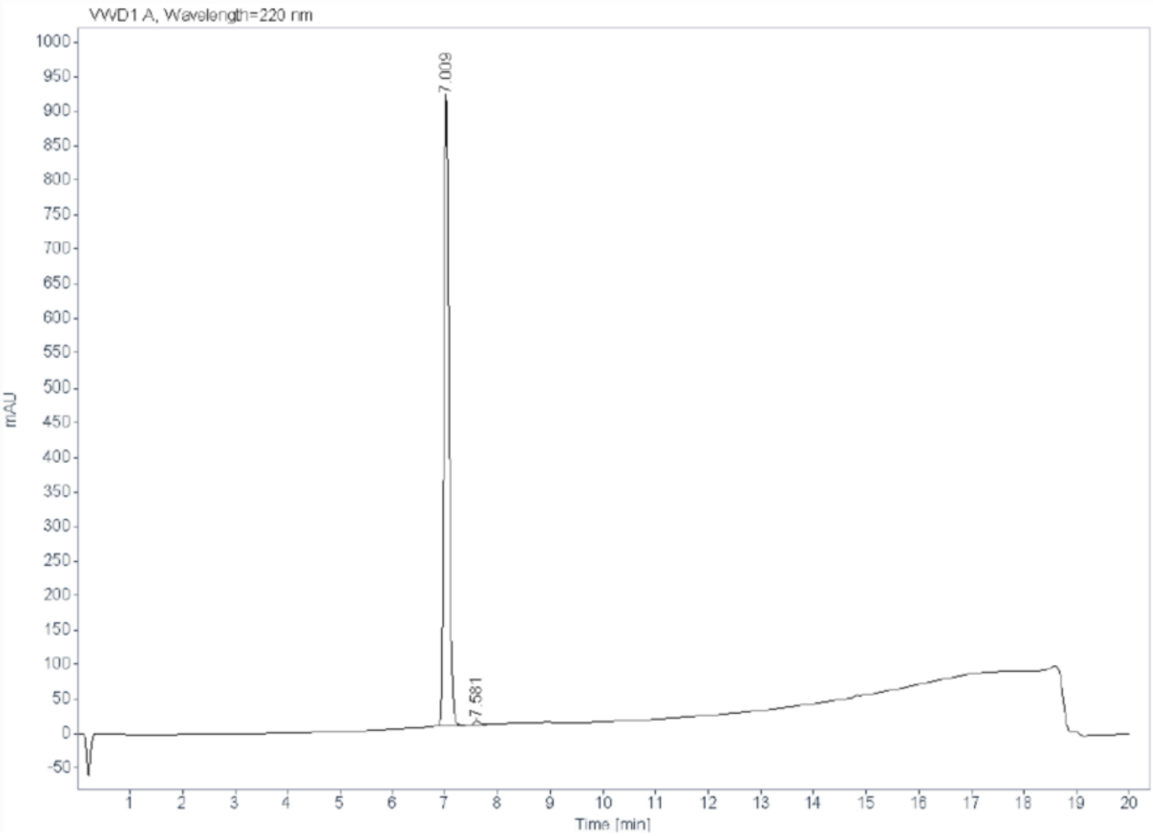

Signal: VWD1 A, Wavelength=220 nm

RT [min]	Type	Width [min]	Area	Height	Area%
7.009	FM	0.1086	5949.2568	912.7531	98.7811
7.581	FM	0.1850	73.4126	6.6154	1.2189
		Sum	6022.6694		

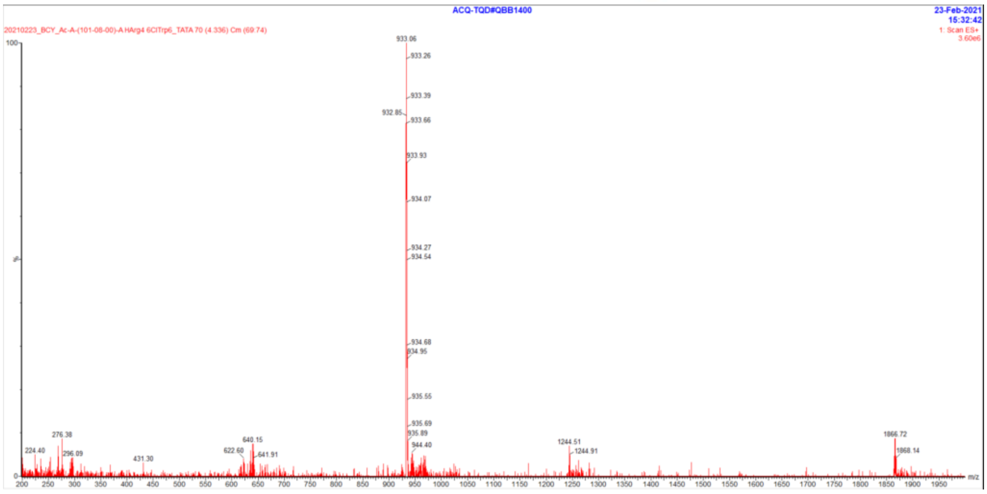

Peptide 76

**Sample name:**	(101-08-00)(TATA)[D-Form] QC
**Instrument:**	1260_2
**Injection date:**	11/11/2019 4:18:26 PM
**Acq. method:**	0595B_AB_Poroshell1<br>20_15.5min.M

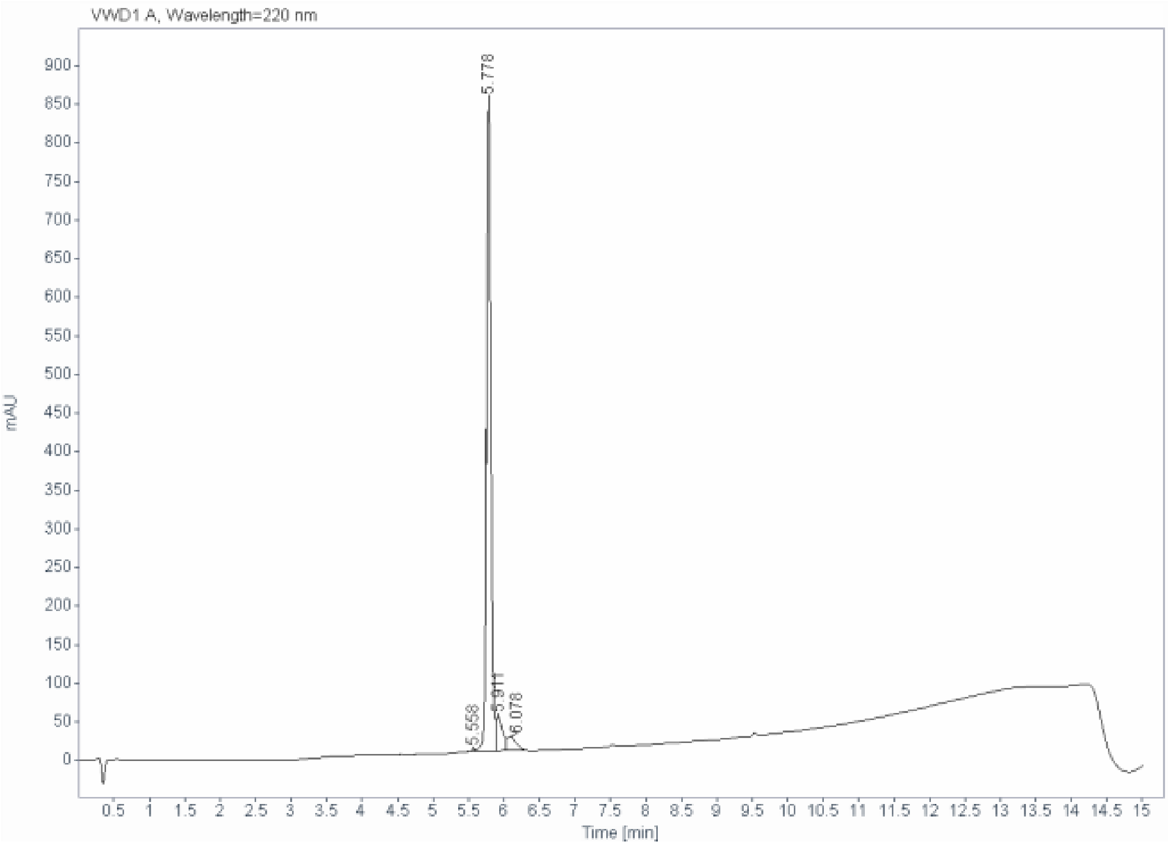

Signal: VWD1 A, Wavelength=220 nm

RT [min]	Type	Width [min]	Area	Height	Area%
5.558	BV	0.0504	10.5694	3.1286	0.2553
5.778	VV	0.0667	3681.5608	847.9104	88.9329
5.911	VV	0.0845	283.0704	45.5661	6.8379
6.078	VB	0.1286	164.5071	18.6974	3.9739
		Sum	4139.7077		

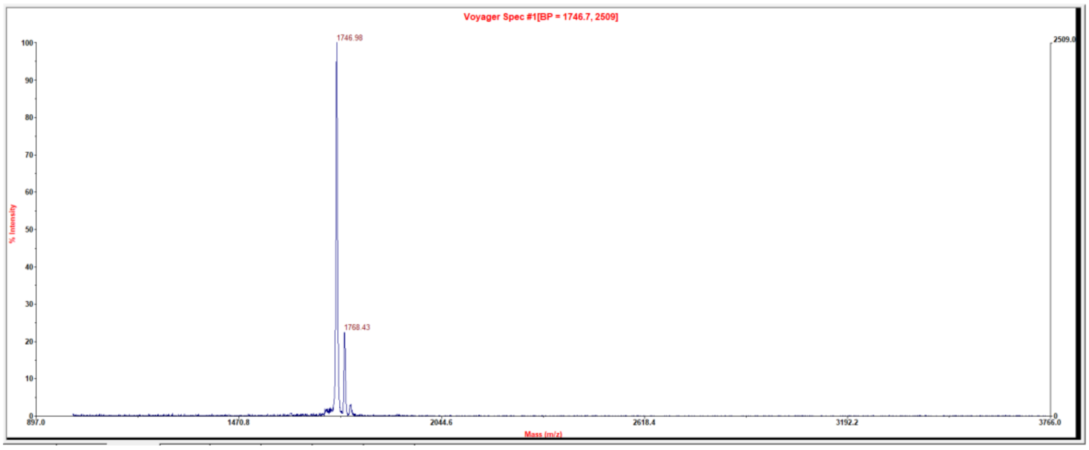

Conjugate 1

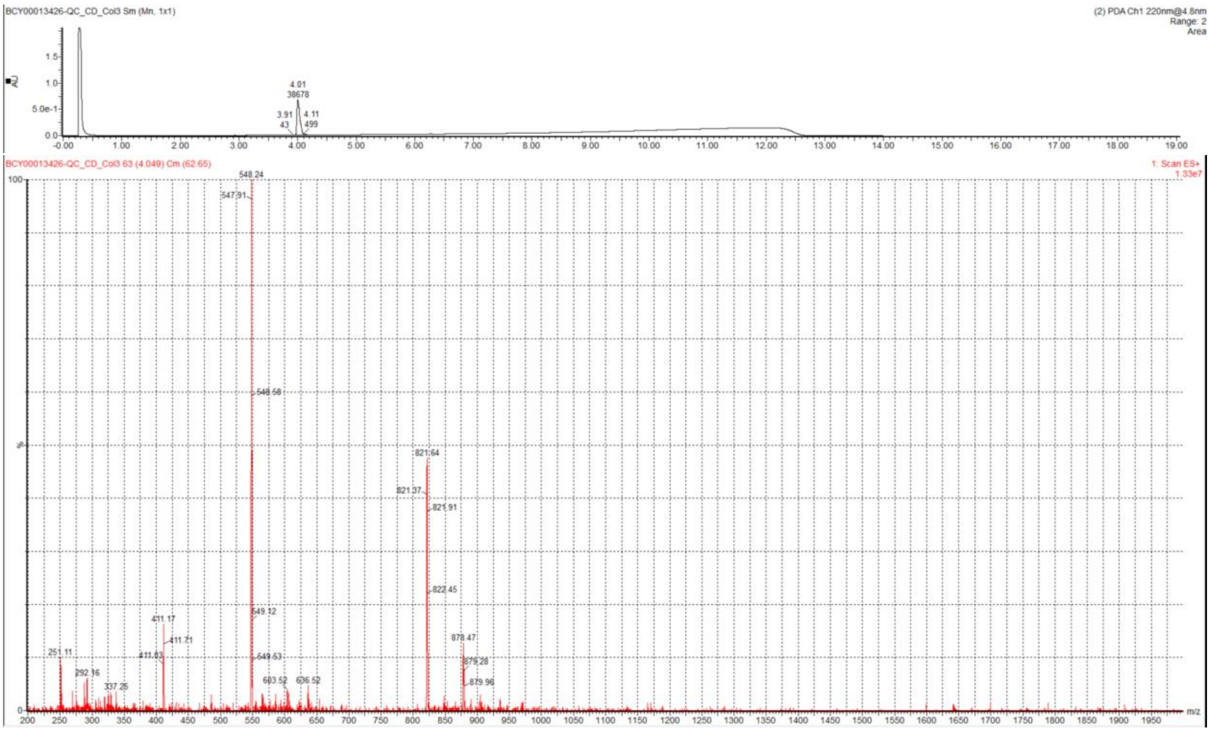

Conjugate 2

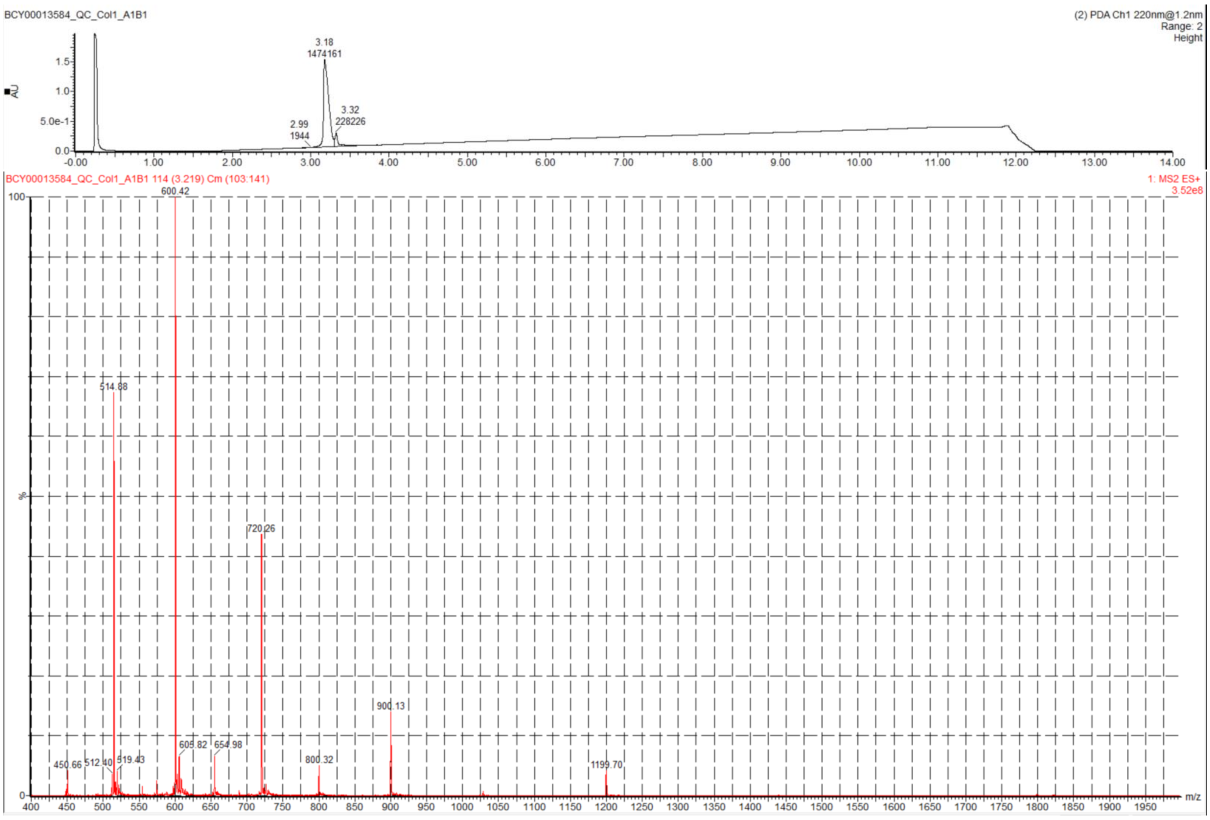

Conjugate 3

**Data file:**	ES19551-238-P1B.dx		
**Sequence Name:**	2021-10-15 14-26-12+08-00	**Project Name:**	Installation HPLC
**Sample name:**	ES19551-238-P1B	**Operator:**	SYSTEM
**Instrument:**	LCMS-QC	**Injection date:**	2021-10-15 23:41:29+08:00
**Inj. volume:**	8.000	**Location:**	P1-B9
**Acq. method:**	20-50-20min-P1waste.amx	**Colum:**	Gemini-NX C18 5um 110A 150mm4.6
**Processing method:**	*GC_LC Area Percent_DefaultMethod.pmx	**Mobile phase:**	A:0.1%TFAin H2O B:0.1%TFAin ACN

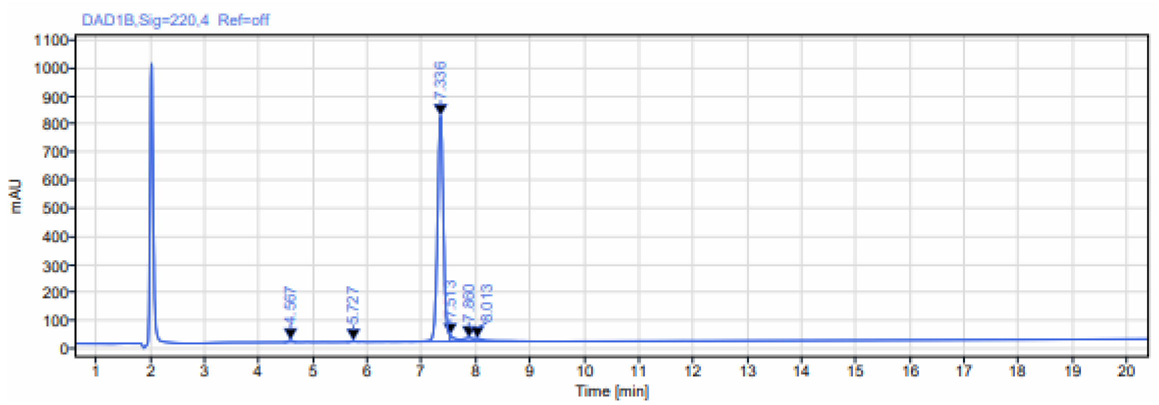

Signal: DAD1B,Sig=220,4 Ref=off

RT [min]	Type	Width [min]	Area	Height	Area%	Name
4.567	MM m	0.24	38.11	6.87	0.58	
5.727	MM m	0.26	17.01	2.99	0.26	
7.336	BM m	0.74	6107.46	802.41	92.86	
7.513	MV m	0.19	128.50	21.92	1.95	
7.860	VV	0.26	151.00	12.11	2.30	
8.013	VB	0.81	135.02	9.53	2.05	
		**Sum**	**6577.10**			

**Sequence Name:**	2021-10-15 11-53-51+08-00		
**Data file:**	ES19551-238-P1A.dx	**Project Name:**	Installation
**Sample name:**	ES19551-238-P1A	**Operator:**	SYSTEM
**Instrument:**	LCMS-QC	**Acquired on:**	2021-10-15 15:31:35+08:00
**Inj. volume:**	10.000	**Location:**	P1-B9
**Acq. method:**	10-80-3min-1.5.amx		
**Processing method:**	*MS_DefaultMethod.pmx		

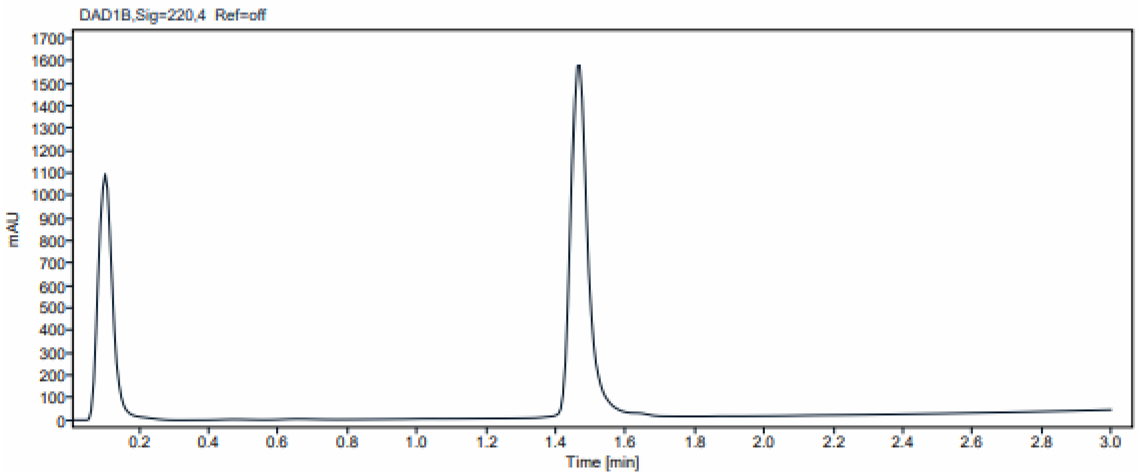

Peak Results (Area Percent at least 1%)

RT (min)	Signal Description	Width (min)	Area	Height	Area%
1.470	MS1 +TIC SCAN ESI Frag=135V Gain=1.0	0.148	1947137.1	623043.0	100.00
	**Sum MS1 +TIC SCAN ESI Frag=135V Gain=1.0**		1947137.1		

Signal Name MS1 +TIC SCAN ESI Frag=135V Gain=1.0

Peak Retention Time 1.470

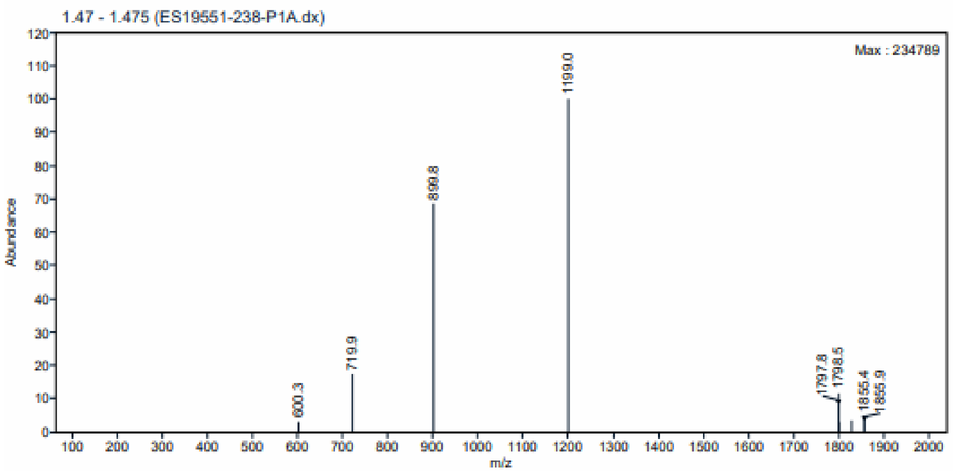

MS Peak Table

m/z	Abundance	Abundance %
1199.0	234789	100.00
899.8	160915	68.54
719.9	40389	17.20
1798.5	27198	11.58
1797.8	19702	8.39

Conjugate 4

Filename : C:\CHEM32\1\DATA\20210116\20210116 2021-01-16 17-07-34\ES12190-1331-P1B.D
Compound ID : ES12190-1331-P1B
Sample ID : ES12190-1331-P1B
Mobile Phase : A: 0.1%TFA in H2O B: 0.1%TFA in ACN
Flow : 1.0ml/min
Column : Gemini-NX C18 5um 110A 150*4.6mm
Instrument : Agilent 1200 HPLC-BE(1-614)

==

Injection Date : Sat, 16. Jan. 2021 Location : Vial 46
Injection Time : 7:21:43 PM Inj. Vol. : 10.0 ul
Acq Method : C:\Chem32\1\DATA\20210116\20210116 2021-01-16 17-07-34\G20-50_20+3MIN.M

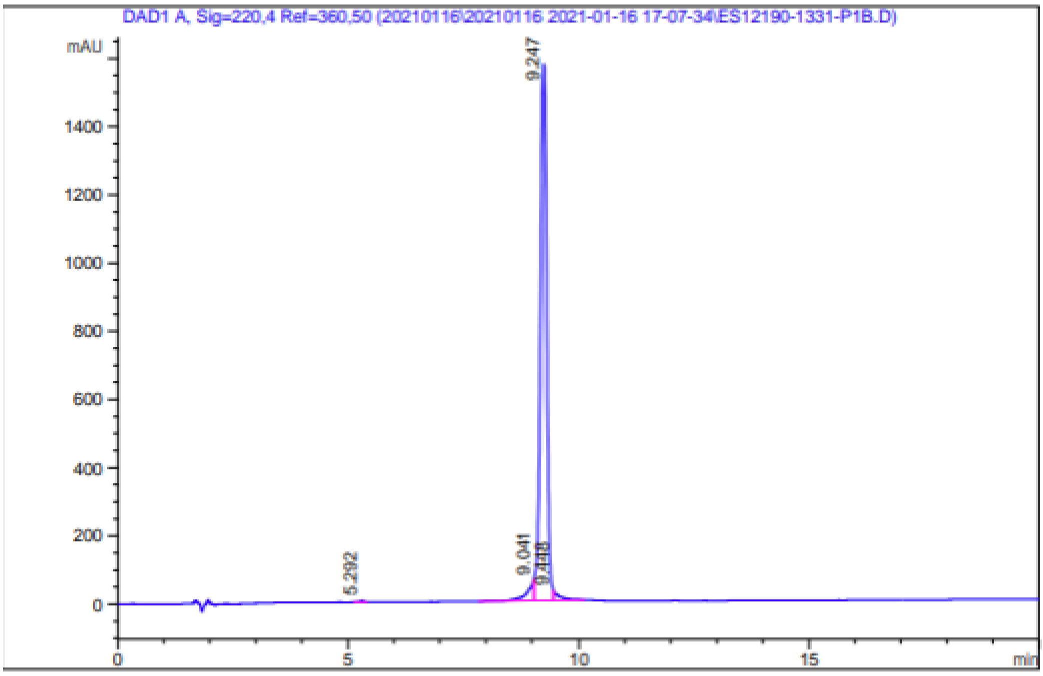

==

Area Percent Report

==

Signal->:DAD1 A, Sig=220,4 Ref=360,50

Peak #	RT [min]	Height	Height %	Width [min]	Area	Area %
1	5.292	2.924	0.175	0.091	16.865	0.119
2	9.041	62.039	3.719	0.163	605.734	4.260
3	9.247	1573.396	94.323	0.141	13329.167	93.742
4	9.448	29.742	1.783	0.150	267.210	1.879

**Data Filename**	ES12190-1331-P1A.d	**Sample Name**	ES12190-1331-P1A
**Sample Type**	Sample	**Position**	P1-B8
**Instrument Name**	Instrument 1	**User Name**	
**Acq Method**	10-80AB_2MIN-400.m	**Acquired Time**	1/15/2021 9:53:12 AM
**IRM Calibration Status**	Not Applicable	**DA Method**	Default.m
**Comment**			

Sample Group **Info.**
Acquisition SW Version 6400 Series Triple Quadrupole B.06.00 (B6025.4 [illegible]

User Chromatograms

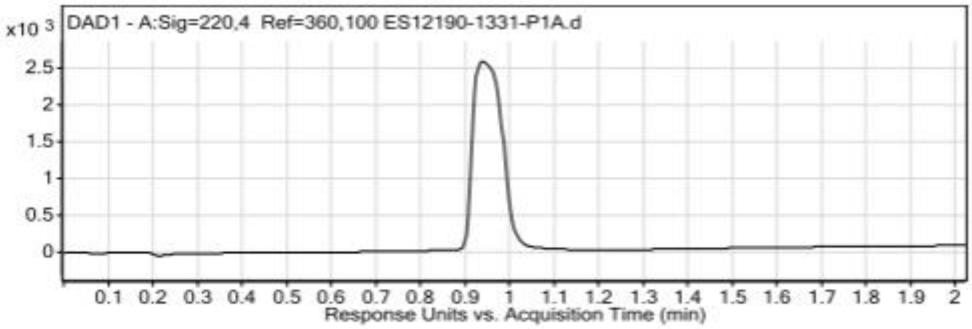

User Spectra

Fragmentor Voltage	Collision Energy	Ionization Mode
120	0	ESI

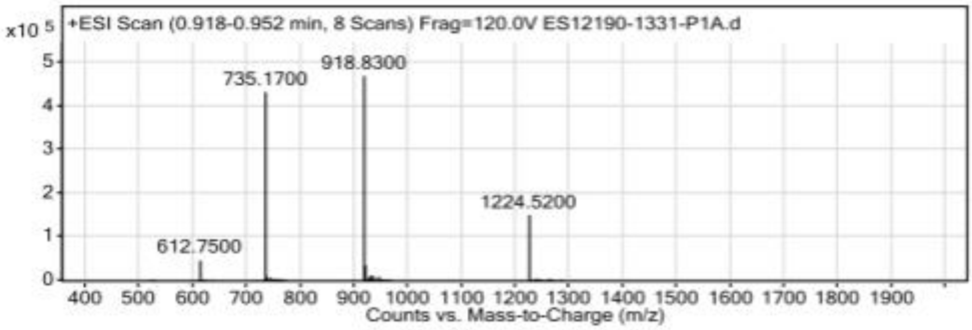

Peak List

m/z	z	Abund
735		53249.06
735.17		433253.16
735.32		98805.48
918.43		166771.44
918.67		331814
918.83		470390.44
1224.23	2	111092.84
1224.4	2	91019.02
1224.52		150640.5
1225.06		51635.01

Conjugate 5

**Data file:**	ES19551-443-P1B.dx		
**Sequence Name:**	2022-03-11 13-49-08+08-00	**Project Name:**	Installation HPLC
**Sample name:**	ES19551-443-P1B	**Operator:**	SYSTEM
**Instrument:**	LCMS-QC	**Injection date:**	2022-03-12 04:03:59+08:00
**Inj. volume:**	8.000	**Location:**	P1-F1
**Acq. method:**	20-50-20min-P1waste.amx	**Colum:**	Gemini-NX C18 5um 110A 150mm4.6
**Processing method:**	*GC_LC Area Percent_DefaultMethod.pmx	**Mobile phase:**	A:0.1%TFAin H2O B:0.1%TFAin ACN

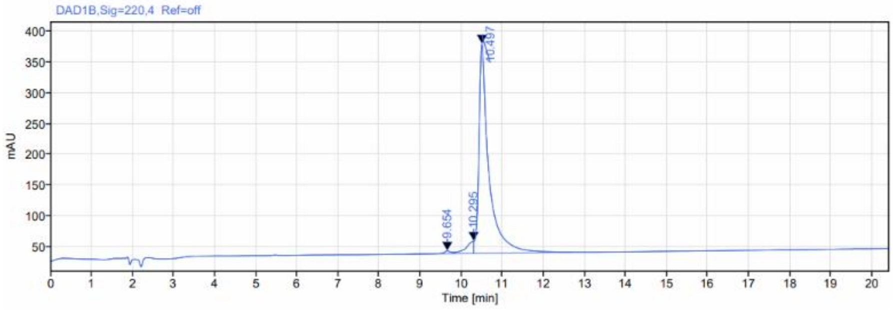

Signal: DAD1B,Sig=220,4 Ref=off

RT [min]	Type	Width [min]	Area	Height	Area%	Name
9.654	BV	1.12	61.56	4.50	1.02	
10.295	VM m	0.49	250.04	19.89	4.13	
10.497	MB m	2.41	5737.41	339.94	94.85	
		**Sum**	**6049.01**			

**Sequence Name:**	2022-03-11 11-38-37+08-00		
**Data file:**	ES19551-443-P1A.dx	**Project Name:**	Installation
**Sample name:**	ES19551-443-P1A	**Operator:**	SYSTEM
**Instrument:**	LCMS-QC	**Acquired on:**	2022-03-11 14:55:49+08:00
**Inj. volume:**	10.000	**Location:**	P1-F1
**Acq. method:**	10-80-2min-1.5_P2.amx		
**Processing method:**	*MS_DefaultMethod.pmx		

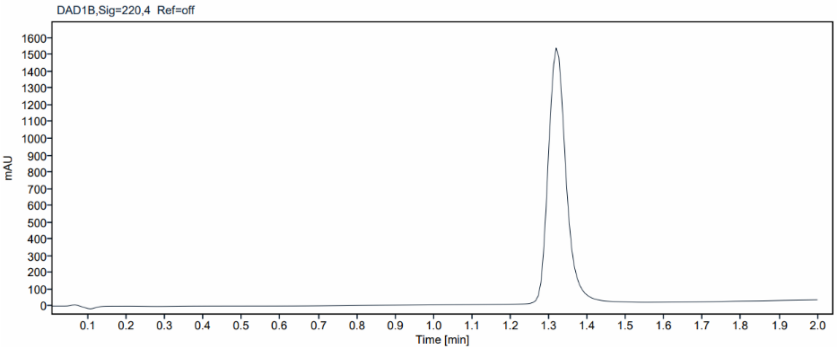

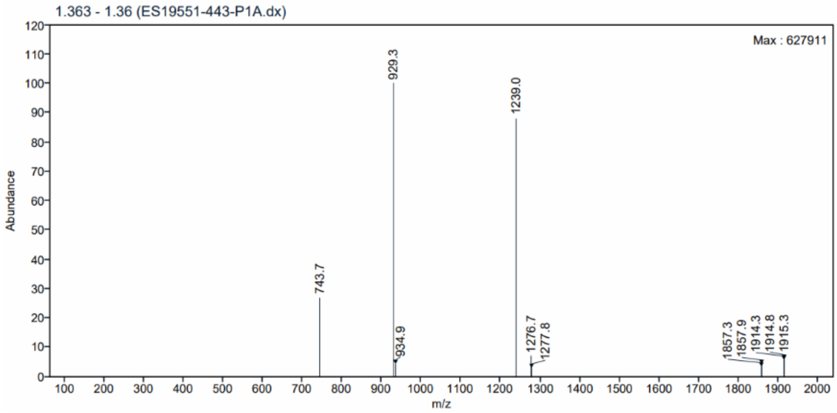

Conjugate 6

**Data file:**	ES19551-187-P1B.dx		
**Sequence Name:**	2021-09-15 16-15-13+08-00	**Project Name:**	Installation HPLC
**Sample name:**	ES19551-187-P1B	**Operator:**	SYSTEM
**Instrument:**	LCMS-QC	**Injection date:**	2021-09-16 07:40:14+08:00
**Inj. volume:**	3.000	**Location:**	P2-D4
**Acq. method:**	20-50-20min-P1waste.amx	**Colum:**	Gemini-NX C18 5um 110A 150mm4.6
**Processing method:**	*GC_LC Area Percent_DefaultMethod.pmx	**Mobile phase:**	A:0.1%TFAin H2O B:0.1%TFAin ACN

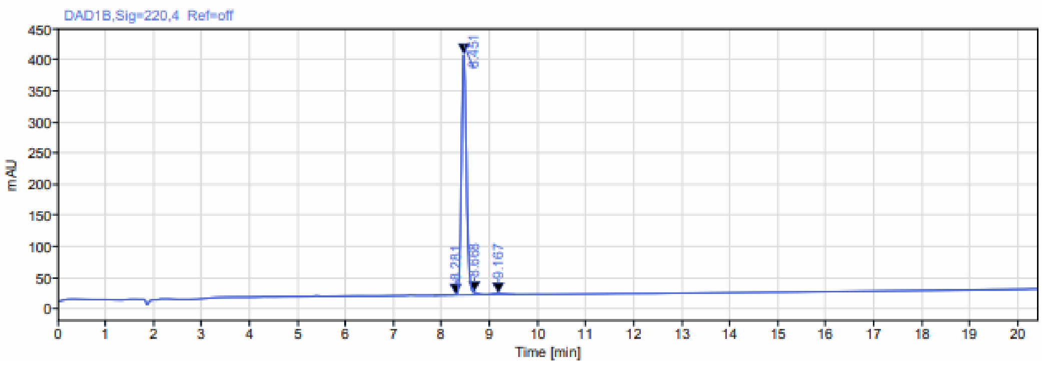

Signal: DAD1B,Sig=220,4 Ref=off

RT [min]	Type	Width [min]	Area	Height	Area%	Name
8.281	BM m	0.46	7.99	0.72	0.28	
8.451	MM m	0.38	2778.23	386.87	97.53	
8.668	MV m	0.25	26.34	4.44	0.92	
9.167	VB	0.61	35.99	1.96	1.26	
		**Sum**	**2848.54**			

Signal Name MS1 +TIC SCAN ESI Frag=135V Gain=1.0

Peak Retention Time 1.556

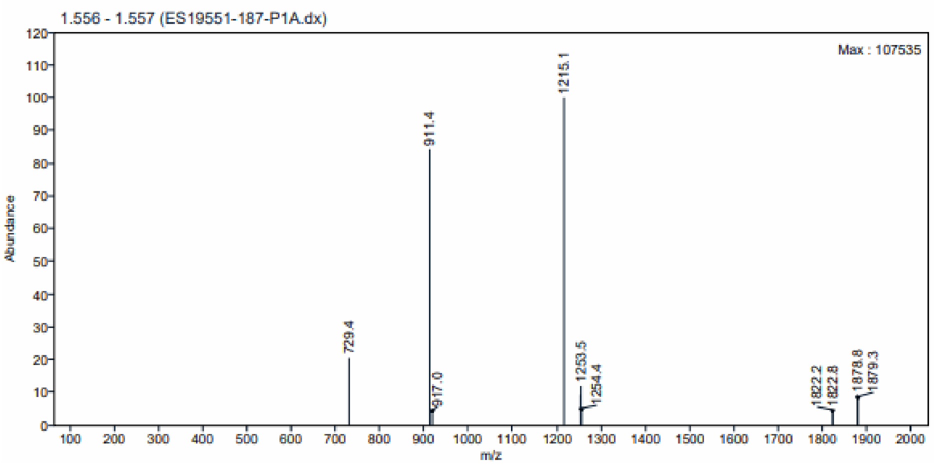

MS Peak Table

m/z	Abundance	Abundance %
1215.1	107535	100.00
911.4	90406	84.07
729.4	21925	20.39
1253.5	12725	11.83
1252.7	9832	9.14
1878.8	9693	9.01
1879.3	8156	7.58

Conjugate 7

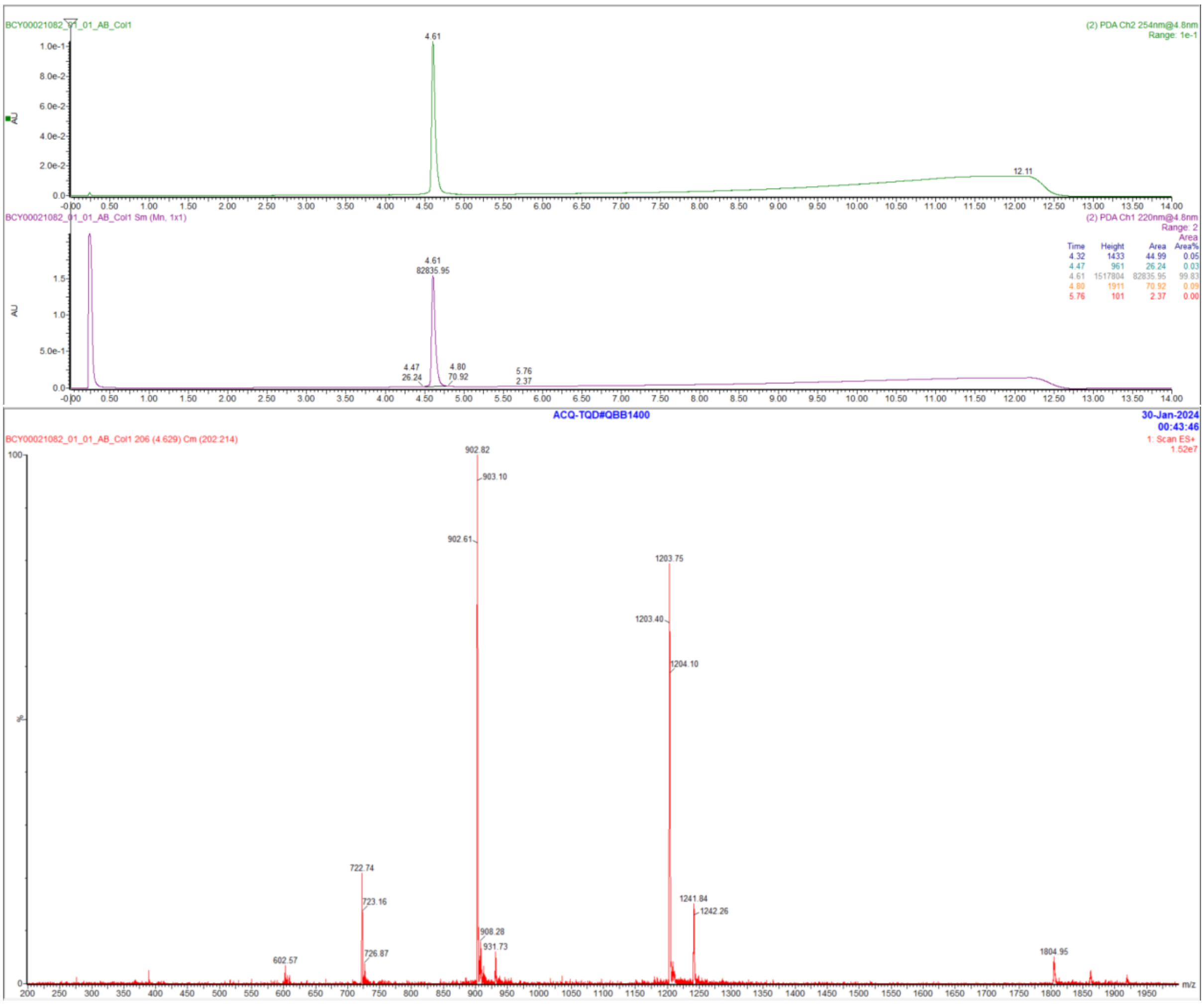
BCY00021082_01_01_AB_Col1
(2) PDA Ch2 254nm@4.8nm
Range: 1e-1
4.61
12.11
AU
BCY00021082_01_01_AB_Col1 Sm (Mn, 1x1)
(2) PDA Ch1 220nm@4.8nm
Range: 2
Time Height Area Area%
4.32 1433 44.99 0.05
4.47 961 26.24 0.03
4.61 1517804 82835.95 99.83
4.80 1911 70.92 0.09
5.76 101 2.37 0.00
4.61
82835.95
4.47
26.24
4.80
70.92
5.76
2.37
ACQ-TQD#QBB1400
30-Jan-2024
00:43:46
BCY00021082_01_01_AB_Col1 206 (4.629) Cm (202:214)
1: Scan ES+
1.52e7
902.82
903.10
902.61
1203.75
1203.40
1204.10
722.74
723.16
726.87
602.57
908.28
931.73
1241.84
1242.26
1804.95
m/z

Conjugate 8

**Sample name:**	(101-08-00)-K[N3]K-DF-18563 RP QC
**Instrument:**	1260_1
**Injection date:**	11/4/2019 12:27:21 PM
**Acq. method:**	0595B_AB_Poroshell1<br>20_15.5min.M

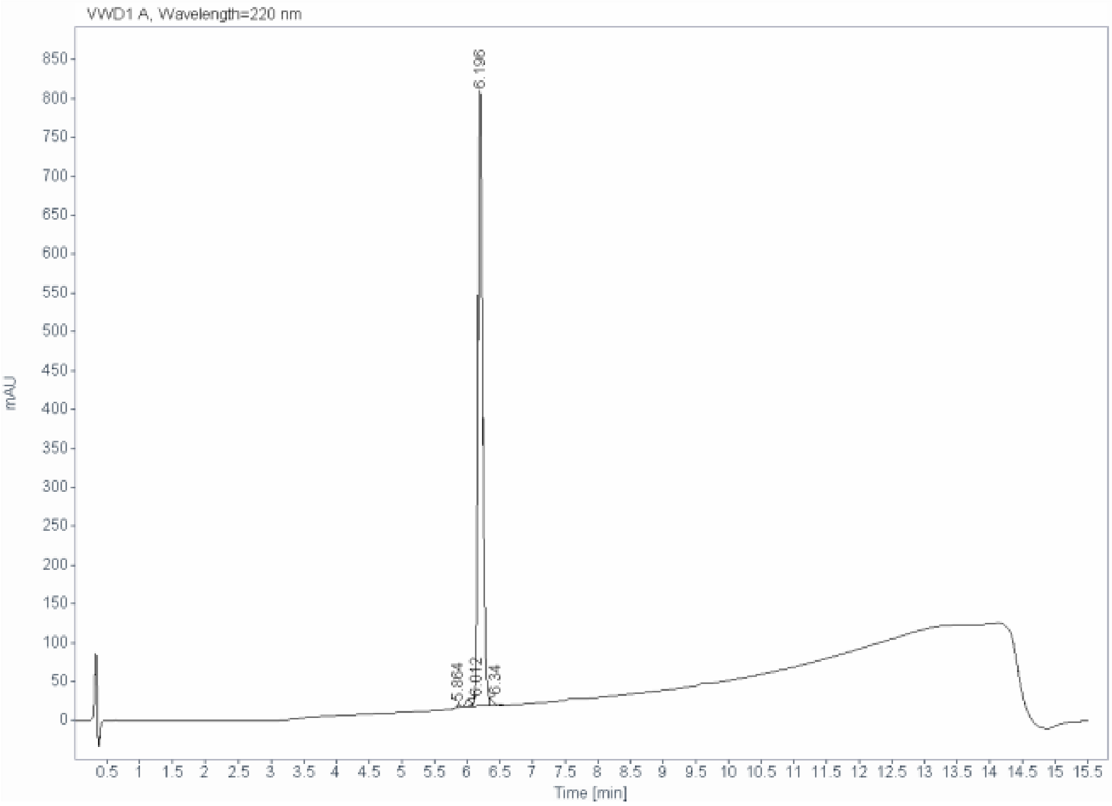

Signal: VWD1 A, Wavelength=220 nm

RT [min]	Type	Width [min]	Area	Height	Area%
5.864	BB	0.0605	17.6040	4.6144	0.4390
6.012	BV E	0.0828	51.9744	9.3539	1.2962
6.196	VF R	0.0756	3902.7305	790.5502	97.3337
6.340	VB	0.0601	37.3299	10.3479	0.9310
		Sum	4009.6388		

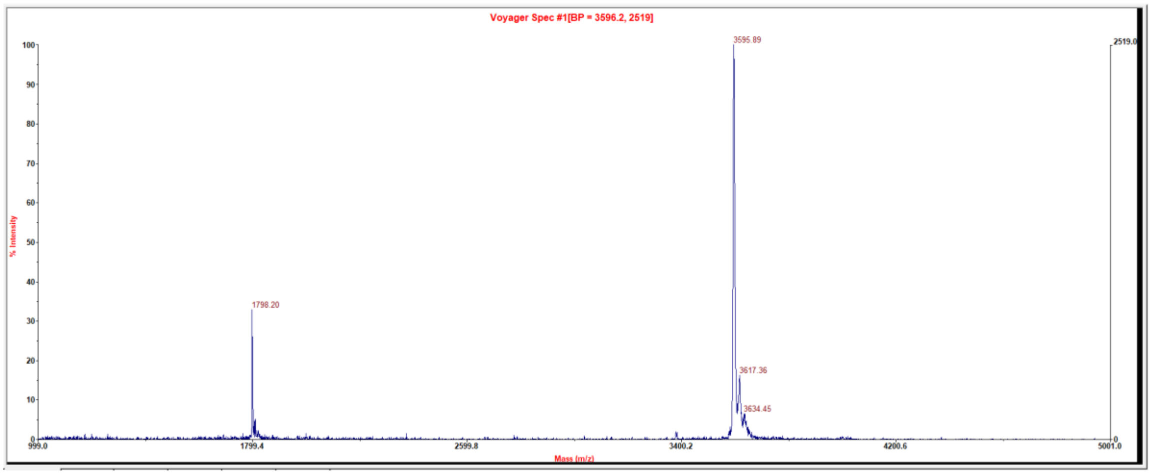

Tracer 1

Sample name:	Fl-G-Sar5-(101-08-00) #2
Instrument:	1260_2
Injection date:	10/26/2020 4:48:46 PM
Acq. method:	0595B_AB_KxC18_QC _20min.M

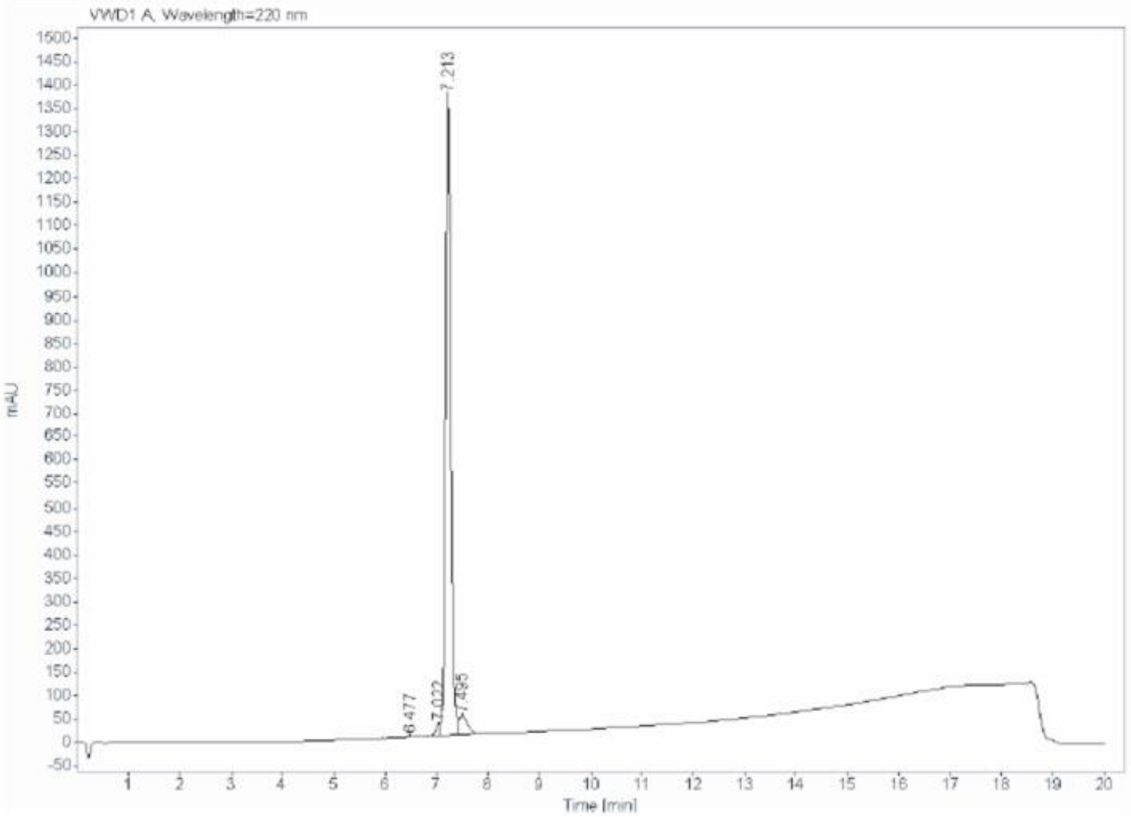

Signal: VWD1 A, Wavelength=220 nm

RT [min]	Type	Width [min]	Area	Height	Area%
6.477	VV	0.0752	7.8869	1.6659	0.0784
7.022	VV F	0.1011	170.6320	24.5470	1.6972
7.213	VV	0.1043	9408.2314	1367.5016	93.5769
7.495	VB	0.1672	467.2570	40.3145	4.6475
		Sum	10054.0073		

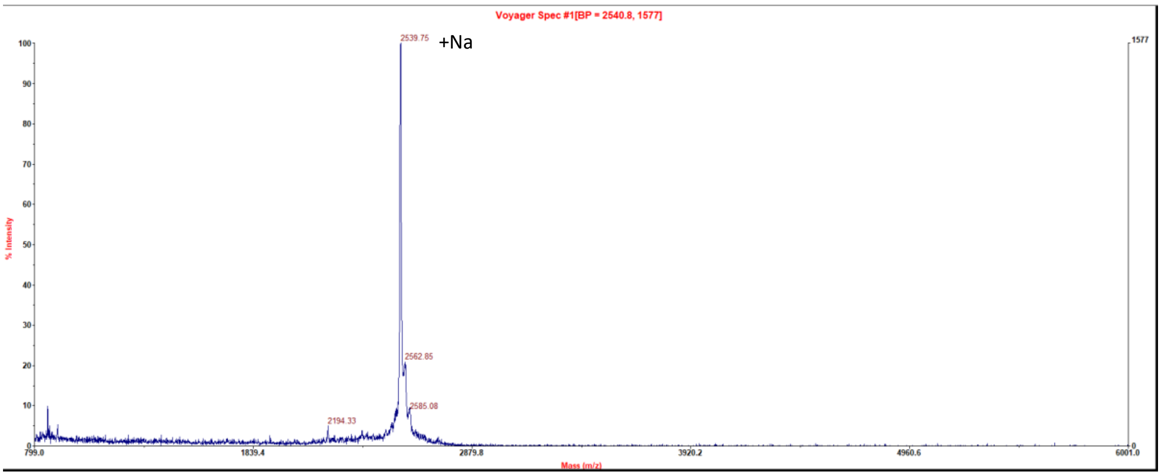

Tracer 2

Sample name:	101-10-00-Sar6-K(Fl)(TATA) QC
Instrument:	1260_2
Injection date:	6/27/2019 3:00:40 PM
Acq. method:	0595B_15.5min.M

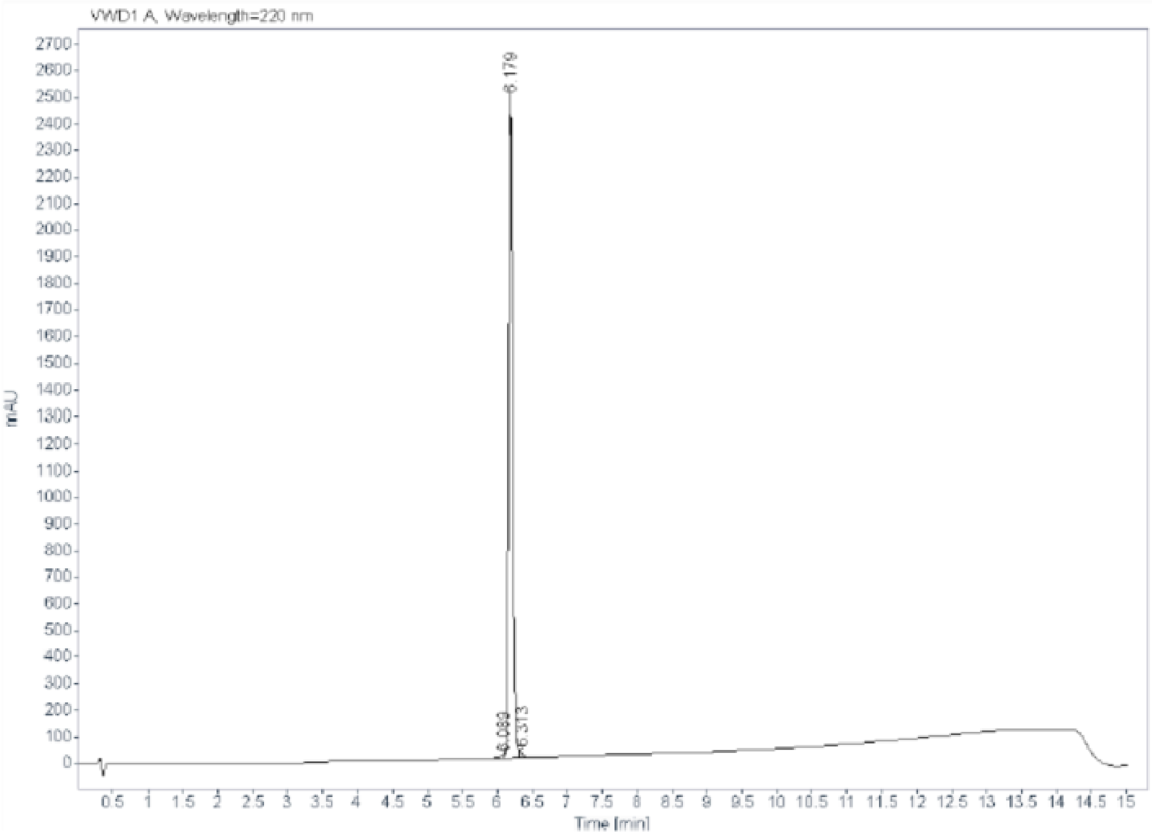

Signal: VWD1 A, Wavelength=220 nm

RT [min]	Type	Width [min]	Area	Height	Area%
6.089	MF	0.1085	69.8961	10.7321	0.6991
6.179	FM	0.0660	9854.9658	2488.1157	98.5732
6.313	FM	0.0434	72.7495	27.9667	0.7277
		Sum	9997.6114		

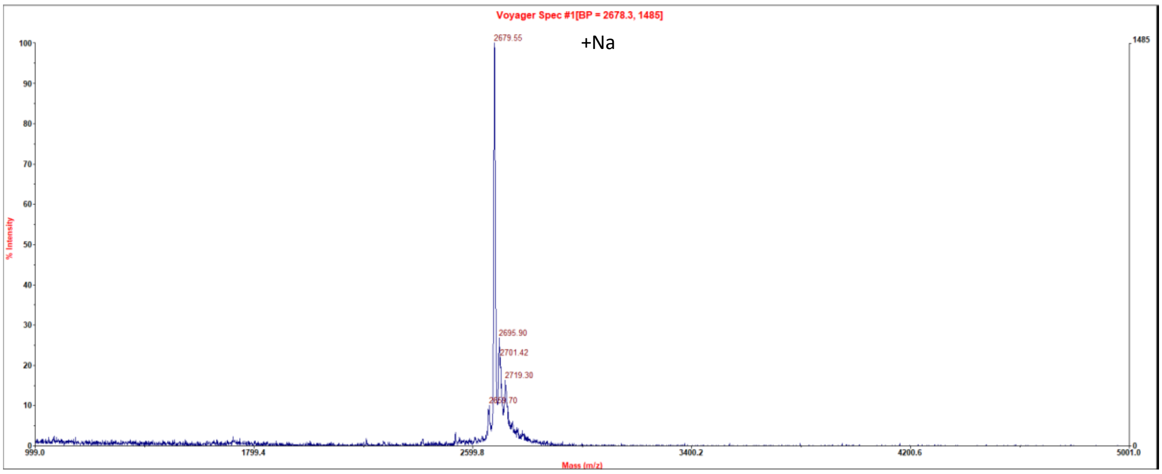

Supplement: Supplementary file 1 — Supplementary Materials [file 42003_2025_8246_MOESM1_ESM.pdf]
